# Proteomic Analysis of the Action of the *Mycobacterium ulcerans* Toxin Mycolactone: Targeting Host Cells Cytoskeleton and Collagen

**DOI:** 10.1371/journal.pntd.0003066

**Published:** 2014-08-07

**Authors:** José B. Gama, Steffen Ohlmeier, Teresa G. Martins, Alexandra G. Fraga, Belém Sampaio-Marques, Maria A. Carvalho, Fernanda Proença, Manuel T. Silva, Jorge Pedrosa, Paula Ludovico

**Affiliations:** 1 Life and Health Sciences Research Institute, School of Health Sciences, University of Minho, Braga, Portugal; 2 ICVS/3B's - PT Government Associate Laboratory, Braga/Guimarães, Portugal; 3 Proteomics Core Facility, Biocenter Oulu, Department of Biochemistry, University of Oulu, Oulu, Finland; 4 Chemistry Research Center, School of Sciences, University of Minho, Braga, Portugal; 5 Institute for Molecular and Cell Biology, Porto, Portugal; University of Tennessee, United States of America

## Abstract

Buruli ulcer (BU) is a neglected tropical disease caused by *Mycobacterium ulcerans*. The tissue damage characteristic of BU lesions is known to be driven by the secretion of the potent lipidic exotoxin mycolactone. However, the molecular action of mycolactone on host cell biology mediating cytopathogenesis is not fully understood. Here we applied two-dimensional electrophoresis (2-DE) to identify the mechanisms of mycolactone's cellular action in the L929 mouse fibroblast proteome. This revealed 20 changed spots corresponding to 18 proteins which were clustered mainly into cytoskeleton-related proteins (Dync1i2, Cfl1, Crmp2, Actg1, Stmn1) and collagen biosynthesis enzymes (Plod1, Plod3, P4ha1). In line with cytoskeleton conformational disarrangements that are observed by immunofluorescence, we found several regulators and constituents of both actin- and tubulin-cytoskeleton affected upon exposure to the toxin, providing a novel molecular basis for the effect of mycolactone. Consistent with these cytoskeleton-related alterations, accumulation of autophagosomes as well as an increased protein ubiquitination were observed in mycolactone-treated cells. *In vivo* analyses in a BU mouse model revealed mycolactone-dependent structural changes in collagen upon infection with *M. ulcerans*, associated with the reduction of dermal collagen content, which is in line with our proteomic finding of mycolactone-induced down-regulation of several collagen biosynthesis enzymes. Our results unveil the mechanisms of mycolactone-induced molecular cytopathogenesis on exposed host cells, with the toxin compromising cell structure and homeostasis by inducing cytoskeleton alterations, as well as disrupting tissue structure, by impairing the extracellular matrix biosynthesis.

## Author Summary

Buruli Ulcer (BU) is a neglected tropical disease caused by *Mycobacterium ulcerans* infection. It has been recognized for many years that BU pathogenesis is mediated by the potent exotoxin mycolactone; however, the molecular action of this toxin on the host cell biology that drives its pathogenesis is not fully understood. Here we present a proteomic-based study that explores the molecular action of mycolactone on host cells biology. Our results provide further molecular evidence for the cytoskeleton-disarrangement induced by mycolactone, and unveil its impact on cytoskeleton-dependent cellular functions. Moreover, we extend the field of action of this toxin to the biosynthesis of collagen, implicating mycolactone on the decrease of dermal collagen found on BU lesions. Given the dependence of *M. ulcerans* virulence on its toxin, these findings on mycolactone's molecular action on host cells and tissues are of major importance for the understanding of BU pathogenesis.

## Introduction

Buruli ulcer (BU) is a neglected tropical disease caused by *Mycobacterium ulcerans* infection [1]. Infection usually starts in the subcutaneous tissue and initially gives rise to non-ulcerative lesions. Histologically, increasing areas of necrosis contrast with the smaller central zone, in which acid-fast bacilli concentrate [2] during both an intracellular phase as well as extracellularly [3], [4]. With disease progression, necrosis advances, radiating from the focus of infection and involving all cells and structures in its path [5]. If left untreated, necrosis extends to the corium and the lesion breaks down into a severe ulcer. In the ulcerative stage of the disease bacteria disseminate and become predominantly extracellular [3], [4], being found throughout the necrotic tissue [5]. The treatment of BU consists primarily in a lingering antibiotic protocol with a combination of rifampicin and streptomycin [6], however surgical resection of infected skin is still necessary for advanced stages [7]. Moreover, the frequent delay in treatment seeking hampers disease management and increases morbidity [8], with serious long-term sequelae [9]. Prevention is also difficult as little is known about disease transmission [10], [11], [12], [13], [14] and no vaccine is currently available [15], [16].

*M. ulcerans* pathogenicity and the tissue damage characteristic of BU are mediated by its toxin mycolactone, a potent cytotoxic and immunosuppressive polyketide-derived macrolide [2], [17], [18], [19], [20], [21], [22]. Mycolactone is produced as a mixture of congeners, with one major form, which is conserved within a given geographical area [23]. Mycolactone A/B is the main variant produced by African isolates; Australian isolates produce mycolactone C [23] and the Chinese isolate MU98912 used in this study produces mycolactone D [24]. Regarding mycolactone's action, *in vitro* studies mainly performed in the mouse fibroblast L929 cell line have shown that the toxin diffuses passively through the plasma membrane [25]. Further studies also show that cells incubated with the toxin display a distinctive cytopathicity, characterized by early actin cytoskeleton rearrangement, cell round-up and detachment from the bottom of the well, and an arrest in the G_0_/G_1_-phase [17], [26], culminating in an apoptotic cell death [19]. Recently, Guenin-Macé et al. unveiled that the toxin targets the actin-cytoskeleton regulator Wiskott-Aldrich syndrome protein (WASP), inducing its hyperactivation [27], and Hall et al. described that mycolactone inhibits co-translational translocation of proteins into the endoplasmic reticulum (ER), thus inhibiting the production of nearly all proteins that transit through the ER [28]. However, despite these advances, the molecular action of this toxin on the host cell biology that drives its pathogenesis is not fully understood.

This work had the purpose of conducting a characterization of the proteome of mycolactone-treated cells, in order to better understand the effects of this toxin on host cell biology. At first, we performed a kinetic characterization of mycolactone's cytopathic, cytostatic and cytotoxic effects on L929 cells. Based on this, specific incubation times and toxin doses were chosen for the proteomic study by two-dimensional electrophoresis (2-DE). Functional studies were performed in both *in vitro* and *in vivo* models to verify our findings in mycolactone-exposed cells and investigate their role in BU pathogenesis. The data obtained showed that cytoskeleton and collagen biosynthesis are severely affected by mycolactone, supporting the involvement of cytoskeleton on mycolactone-induced cytopathogenicity and identifying a new activity of the toxin on the decrease of the collagen content in *M. ulcerans*-infected tissues.

## Results

### Kinetic characterization of mycolactone cytopathic, cytostatic and cytotoxic effects

The time- and dose-dependent kinetics of the cytostatic and cytotoxic activities of mycolactone were investigated by an integrated analysis of cell cycle and cell death in L929 cells. Doses of mycolactone were selected through a pre-screening MTS assay based on the concentrations reported for human ulcer exudates (0–300 ng/mL) [29]. MTS assay showed a threshold around 15 ng/mL, above which mycolactone is progressively cytotoxic reaching a plateau at 50 ng/mL (data not shown). Therefore, the range of mycolactone concentrations tested in this study was narrowed to 12.5–50 ng/mL.

**Figure 1:**
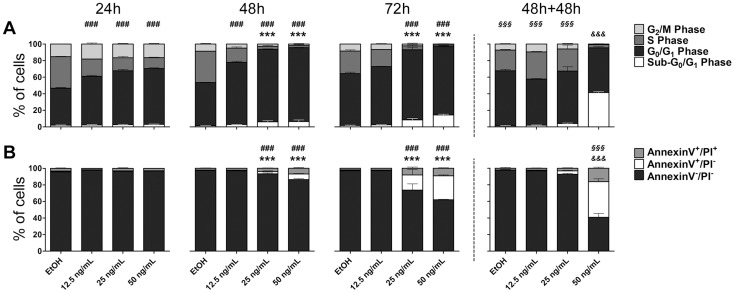
Kinetics of mycolactone cytostatic and cytotoxic effects. Mouse fibroblasts L929 cells were incubated for 24, 48 or 72(referred to as 48 h+48 h). Cell cycle analysis (A) and annexin-V/PI assay (B) were performed for each-time point. Bars represent the mean + SD (n = 3) from one out of, at least, two independent experiments. Each condition was compared to EtOH-treated samples throughout each time-point (24 h, 48 h and 72 h) by Two-way ANOVA with Bonferroni posttest; statistical differences were represented by *** (*P*<0.001) for sub-G_0_/G_1_ and Annexin-V^+^/PI^−^ , and by ^###^ (*P*<0.001) for G_0_/G_1_ Phase and Annexin-V^+^/PI^+^. Each condition at 48 h+48 h time-point was compared with the same condition at the 48 h by Two-way ANOVA with Bonferroni posttest; statistical differences were represented by *^&&&^* (*P*<0.001) for sub-G_0_/G_1_ and Annexin-V^+^/PI^−^, and by *^§§§^* (*P*<0.001) for G_0_/G_1_ Phase and Annexin-V^+^/PI^+^.

Results presented in figure 1 show that the ethanol (vehicle) equivalent (<0.002%), as well as the mycolactone concentration below the threshold (12.5 ng/mL), had no detectable cytopathic (rounding and detachment) or cytotoxic effects. Cytotoxic doses of mycolactone (>12.5 ng/mL) induced detachment, cell cycle arrest in G_0_/G_1_ phase at 48 h and 72 h of treatment and the appearance of a sub-G_0_/G_1_ population, compatible to apoptotic cells, more evident at 72 h (figure 1A). Consistent with cell cycle data, annexin-V/PI assays revealed an annexin-V^+^/PI^−^ population for the highest mycolactone concentrations (25 and 50 ng/ml) at 72 h (figures 1B), indicative of apoptotic cells.

It was previously reported that cells incubated with mycolactone re-grow when mycolactone is removed from the medium, indicating that mycolactone's effect might be reversible [17]. To further investigate the reversibility of mycolactone's effect, cells were incubated with different concentrations of mycolactone for 48 h and afterwards washed and incubated in fresh media for an extra 48 h period (figure 1, 48 h+48 h). We found that cells that had previously been incubated with the lowest cytotoxic concentration of the toxin (25 ng/mL) re-adhere, recover the normal cell cycle progression (figure 1A, 48 h+48 h) and appeared to overcome the cytotoxic stimulus, since no increase in the sub-G_0_/G_1_ or annexin-V^+^/PI^−^ populations was observed (figures 1A and 1B, 48 h+48 h). On the other hand, cells that had been previously incubated with the highest cytotoxic dose (50 ng/mL), while remaining in suspension, were not able to overcome the initial stress induced by mycolactone and became committed to death (figures 1A and 1B, 48 h+48 h). These data demonstrate that the reversibility of mycolactone's effect occurs within a window of concentrations around 25 ng/mL.

Overall, within the range of studied mycolactone concentrations, we found doses that did not induce observable cytotoxic effects, doses that induced a reversible stress, and doses that irreversibly triggered an apoptotic cell death.

**Figure 2:**
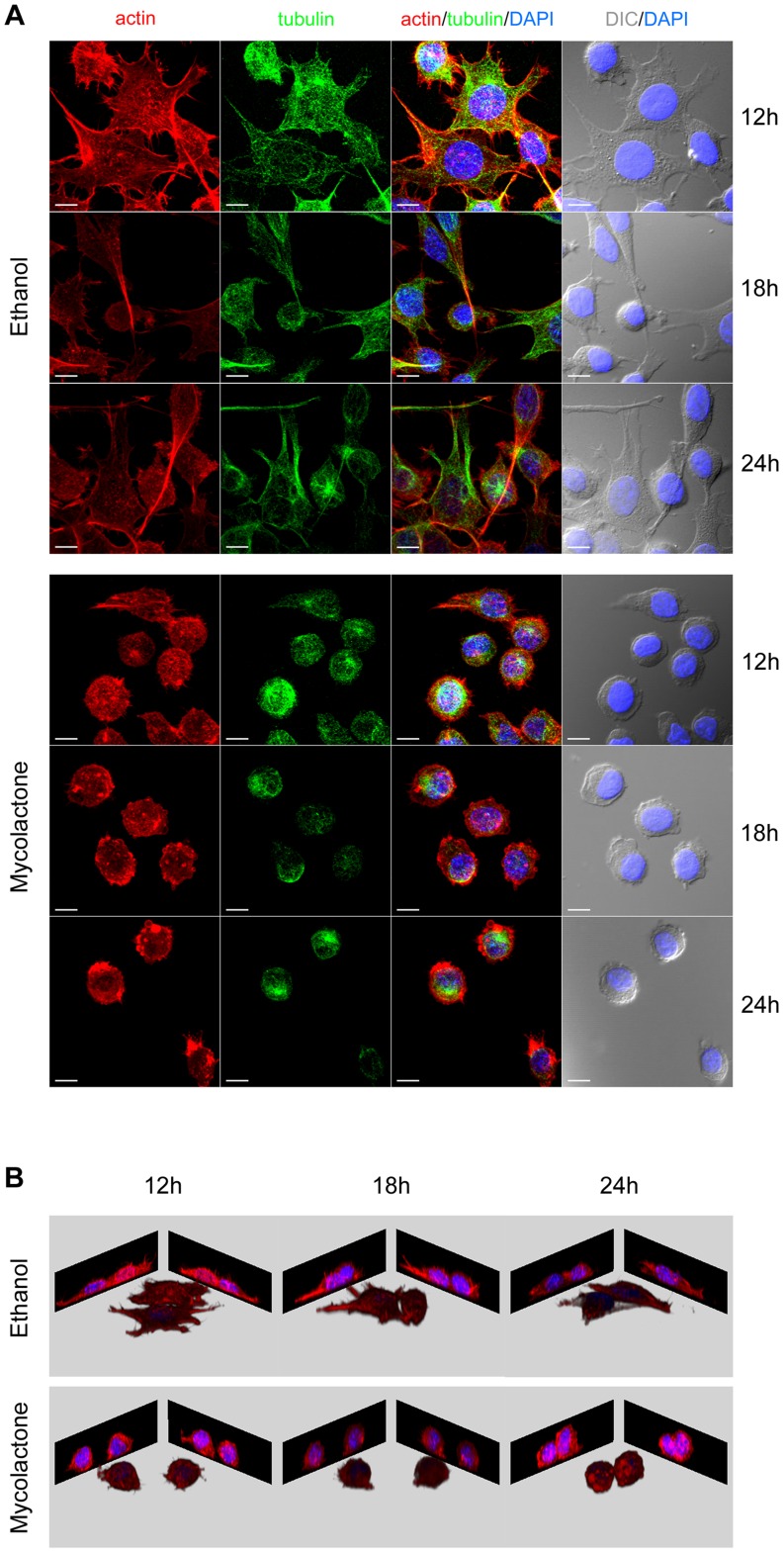
Mycolactone induces cytoskeletal alteration, cell round up and detachment. Mouse fibroblasts L929 cells seeded on coverslips were incubated for 12, 18 or 24/mL of mycolactone. Cytoskeletal changes were visualized by immunofluorescence microscopy using rhodamine-phalloidin conjugate (red) and a tubulin-specific antibody (green). Nuclei were stained with 4′,6-diamidino-2-phenylindole (DAPI). The cellular shape was visualized by differential interference contrast (DIC) microscopy. White horizontal bar represents a 10 µm scale (A). Blue (nucleus) and red (actin) channels were used for 3D remodeling of the respective confocal z stacks (B).

To further characterize our model, the kinetics of the cytopathic effects, namely cytoskeleton alterations and cell round-up and detachment, were also assessed. In cells incubated with mycolactone, we observed not only the previously described alterations for actin [26], but also changes in the tubulin cytoskeleton, which appeared bended into a microtubule hank (figure 2A). Within 12–18 h of exposure to the toxin, actin ultrastructures (stress fibers and lamellipodia) were lost, and, although still attached, most of the cells were completely round-up by 18–24 h (figures 2A and 2B). At 24 h, half of the cells were already in suspension, while the remaining cells eventually detached in the following 12 h [26]: detachment being probably a consequence of cell round-up and loss of adhesion structures.

### Mycolactone causes time-dependent proteomic changes related to cytoskeleton and collagen biosynthesis

To study the effects of mycolactone on host cell biology, the total cellular proteome from mouse fibroblast L929 cells incubated with 50 ng/mL of mycolactone (dose that triggers a commitment to apoptotic cell death) or the ethanol equivalent (control), was separated by 2-DE. To establish a temporal perspective of mycolactone's action, three incubation times were chosen: 24 h (when cells are detaching, but viable and mounting a response to the mycolactone-induced stress, associating with the first detectable consequences of mycolactone on the fibroblast proteome); 48 h (when cells become committed to death, coinciding with the onset of an apoptotic population); and 48 h+48 h, (when most of the cells are in the process of apoptotic cell death, with only 40% of viability) (figure 1).

**Figure 3:**
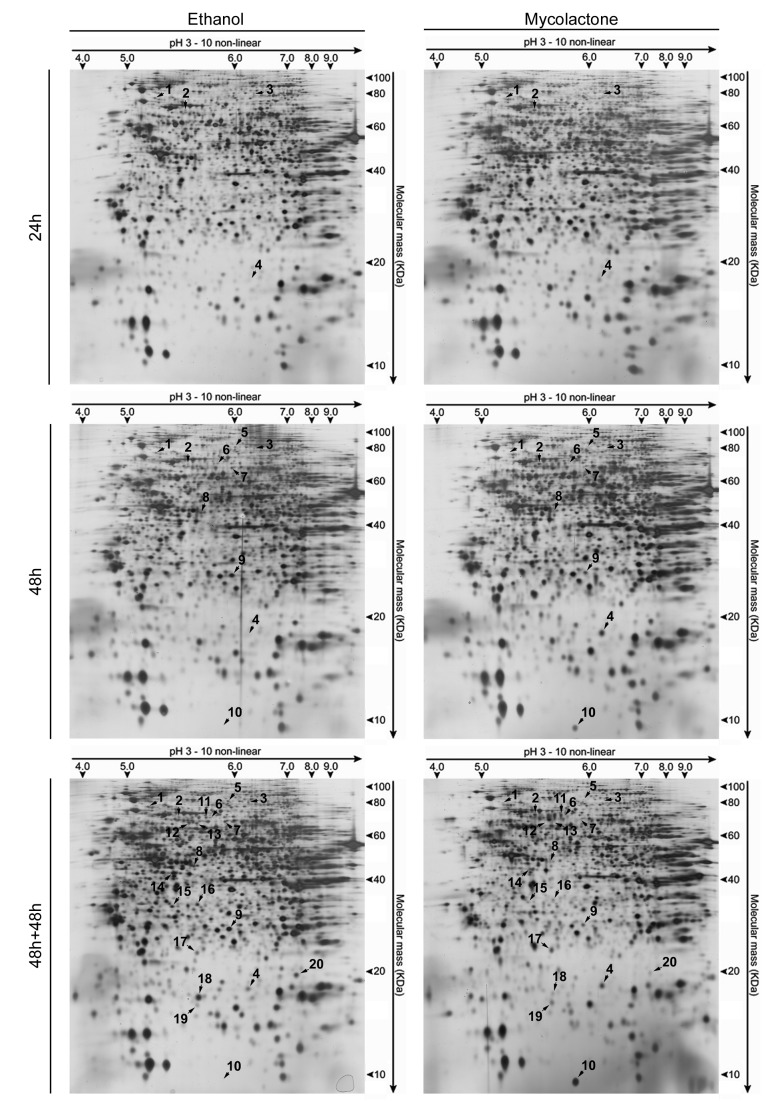
Mycolactone causes time-dependent proteomic changes. Mouse fibroblasts L929 cells were incubated for 24 or 48(50 ng/mL). Additionally, an assay was performed where cells were incubated for 48 hours in the same conditions followed by a 48 hour incubation period in fresh medium (referred to as 48 h+48 h). Representative silver-stained 2-D gels of total protein extracts (100 µg) are shown for the different exposure times (all analyzed gels are in Supplementary Information). Spots altered upon treatment are represented by numbers (1–20).

**Figure 4:**
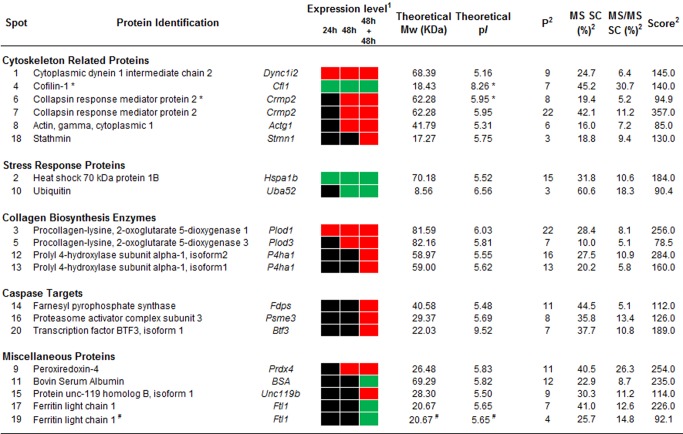
Altered proteins upon mycolactone treatment. Figure shows the proteins identified for each spot (numbers according to figure 3 and figure S1) clustered into five categories. (1) Expression levels show decreased (red), increased (green) or no changes (black) in spot intensities upon mycolactone treatment. (2) MS and MS/MS parameters are presented, including the number of specific matched peptides (P), the sequence coverage (SC) in percentage, as well as the MS scores. Phosphoproteins (*) and putative protein fragments (#) are indicated. Full MS and MS/MS data in Supplementary Information.

The comparison of control cells proteome at different time-points did not reveal any significantly changed spots, showing that ethanol (vehicle) represents a suitable control. In contrast, the comparison between the proteome of control and mycolactone-treated cells at each time-point revealed a time-dependent increase in the number of changed spots, with 4 spots changed at 24 h, 10 at 48 h and 20 at 48 h+48 h. All 20 spots were identified by mass spectrometry and found to correspond to 18 proteins comprising 5 up- and 13 down-regulated (figure 3 and figure 4).

To reveal the cellular processes altered by mycolactone, the proteins were clustered into functional groups according to UniProt database (www.uniprot.org). The major groups comprised (i) cytoskeleton-related proteins (Dync1i2, Cfl1, Crmp2, Actg1, Stmn1); (ii) stress response proteins (Hspa1b, Uba52); and (iii) collagen biosynthesis enzymes (Plod1, Plod3, P4ha1) (figure 4 and figure S1). This reveals that mycolactone results in an alteration of cytoskeleton-related proteins and down-regulation collagen biosynthesis enzymes. On the other hand, stress response proteins were up-regulated. An additional group (caspase targets) was created clustering proteins identified as caspase substrates (Fdps, Psme3, Btf3) through the web caspase substrates database CASBAH (www.casbah.ie) [30]. Consistent with being caspase substrates, these proteins were only decreased in the last time point (48 h+48 h) when most of the cells were undergoing an apoptotic death process (figure 1). Four additional proteins were classified as miscellaneous proteins (Prdx4, BSA, Unc119b, Ftl1) (figure 4 and figure S1).

Overall, the proteome of mycolactone-treated cells revealed that the intracellular structure (cytoskeleton) and the extracellular matrix (collagen) are severely affected by the *M. ulcerans* toxin.

### Intracellular structure: Mycolactone impairs cytoskeleton dynamics and functions

The proteomic study revealed several regulators and structural components of both microfilaments and microtubules affected by mycolactone treatment after 24 h (Dync1i2, Cfl1), 48 h (Crmp2, Actg1) and 48 h+48 h (Stmn1) (figure 4 and figure S1).

Cytoplasmic dynein 1 intermediate chain 2 (Dync1i2) is a non-catalytic subunit of the microtubule-associated molecular motor dynein, which is involved in the transport of elements of the Golgi apparatus, endosomes and lysosomes [31]. The here detected early (24 h) down-regulation of Dync1i2 (spot 1) suggests that this transport may be compromised in mycolactone-treated cells.

Three other proteins altered in cells treated with the toxin are cytoskeleton regulators (Cfl1, Crmp2, Stmn1). Cofilin 1 (Cfl1, spot 4), a well-established regulator of actin dynamics, promotes microfilament assembly or disassembly depending upon the concentration of Cfl1 relative to actin and other actin-binding proteins, as well as upon its phosphorylation status [32]. Collapsin response mediator protein 2 (Crmp2), which was identified in two spots (spot 6 and 7), is a multifunctional adaptor protein which can induce microtubule assembly by binding to αβ-tubulin heterodimers [33], whereas stathmin (Stmn1, spot 18) has been described as a microtubule-destabilizing oncoprotein [34]. Interestingly, the isoelectric points (p*I*s) of spots 4 (Cfl1) and 6 (Crmp2) differed in the gel from their expected theoretical values (figure 3 and figure 4), suggesting posttranslational modification such as phosphorylation. Given that the regulatory activity of Cfl1 [32] and Crmp2 [33] can be modulated by phosphorylation, we studied the phospho-status of these proteins with the Pro Q Diamond phosphostaining. Indeed, the analysis of the phosphoprotein stained gel revealed that both spots were phosphorylated (figure S2). Thus, mycolactone increases the phospho-Cfl1 at 24 h, decreases both phosphorylated and non-phosphorylated forms of Crmp2 at 48 h, and decreases Stmn1 in the latest time point (48 h+48 h).

In addition to the alteration of cytoskeleton regulators, the proteomic study revealed that the cytoskeleton is also altered on its structural components. Actin gamma (Actg1), a component of microfilaments, is down-regulated at 48 h in mycolactone-treated cells.

Overall, these results show that cells exposed to mycolactone undergo a process of cytoskeleton remodeling involving regulators and structural components, providing a novel molecular basis for the effect of mycolactone on this organelle.

### Mycolactone impairs autophagy, a cytoskeleton-dependent cellular function

**Figure 5:**
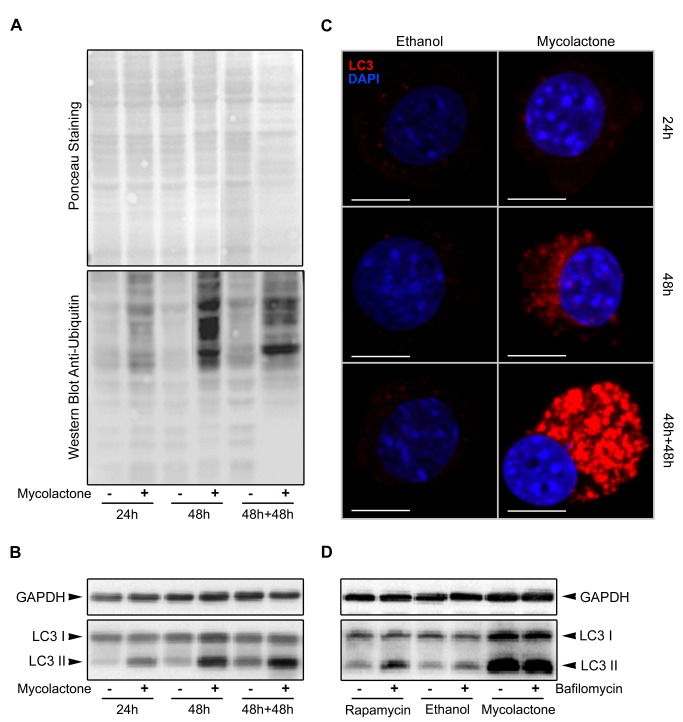
Mycolactone causes an up-regulation of the ubiquitin-proteasome pathway and an accumulation of autophagosomes. In A, B and C, mouse fibroblasts L929 cells were incubated for 24 or 48(−) or mycolactone (50 ng/mL, +). Additionally, an assay was performed where cells were incubated for 48 hours in the same conditions followed by a 48 hour incubation period in fresh medium (referred to as 48 h+48 h). At each time-point, total protein was extracted and Western blot was performed to assess ubiquitinated proteins (A) or LC3 processing (B). Additionally, cytospins were made to assess immunofluorescence LC3 (red). White horizontal bars represent a 10 µm scale (C). In D, mouse fibroblasts L929 cells were incubated for 48 hours either with ethanol or mycolactone (50 ng/mL) and bafilomycin A1 (10 nM, +) or DMSO (vehicle control, −) was added 2 hours prior the end of the assay. Additionally, mouse fibroblast L929 cells were incubated for 48 hours without any stimuli until 2 hours prior the end of the assay, when autophagy was induced with rapamycin (1 µM), and bafilomycin A1 (10 nM, +) or DMSO (vehicle control, −) was added. At the end of the assay, total protein was extracted and Western blot was performed to assess LC3 processing (D).

Two stress response proteins (Hspa1b, Uba52) were up-regulated upon treatment with mycolactone. Spot 10 (figure 4 and figure S1) was identified as a fusion protein (Uba52) consisting of N-terminal ubiquitin and C-terminal 60S ribosomal protein L40. The detected spot position in the gel (figure 3) in comparison with the theoretical positions for the fusion protein (p*I* 9.87/14.7 kDa), ubiquitin (p*I* 6.56/8.6 kDa) and the ribosomal protein (p*I* 10.32/6.2 kDa) suggest the presence of ubiquitin. Indeed, all three spot-specific peptides covered amino acids 13–55 revealing that ubiquitin is present. The here detected increase of free ubiquitin after 48 h of mycolactone treatment could result from an inhibition of ubiquitin ligases or from an up-regulation of its expression. To investigate this in more detail, protein ubiquitination was studied by western blot, which revealed that mycolactone exposure results in an increase of ubiquitinated proteins, more evident at 48 h and 48 h+48 h (figure 5A). Therefore, these data show that rather than an inhibition of ubiquitin ligases, mycolactone induces an up-regulation of the ubiquitin/proteasome system (UPS).

The UPS and the lysosomal degradation system (autophagy) are the two main cellular degradative pathways. These systems crosstalk each other and the up-regulation of one may occur in response to a down-regulation/dysfunction of the other. Autophagy is known to be dependent on microtubule cytoskeleton [35] and dynein-driven transport [36] with dynein playing a role in the delivery of autophagosome contents to lysosomes during autophagosome-lysosome fusion [36]. Since microtubules (Figure 2) and dynein (figure 4 and figure S1) were found to be affected by mycolactone, we hypothesized that the mycolactone-induced cytoskeleton-related changes might impair the autophagic process leading to the up-regulation of the UPS. Therefore the role of mycolactone in autophagy was further investigated.

During autophagy the cytosolic form of LC3 (LC3-I) is conjugated to phosphatidylethanolamine (PE) to form LC3-PE (LC3-II), which is recruited to autophagosomal membranes. As the autophagosomes fuse with lysosomes to form autolysosomes, LC3-II is degraded together with the intra-autophagosomal components by lysosomal hydrolases. Thus, lysosomal turnover of the autophagosomal marker LC3-II reflects autophagic activity [37]. Processing of this marker was analyzed by western blot and immunofluorescence. Western blot revealed an increase of the autophagosome marker LC3-II in cells treated with mycolactone (figure 5B) compatible with an inhibition of autolysomes formation. In agreement, an increase of LC3-positive cytoplasmic vesicles upon toxin exposure was also detected with the immunofluorescence assay (figure 5C, red-stained). To further understand these results, we treated L929 cells with different stimuli and added bafilomycin A1 to inhibit the autolysosomal degradation step [38] 2 hours before protein extraction (figure 5D). The increase of LC3-II induced by mycolactone (figure 5B and 5C), together with the lack of difference in LC3-II in cells treated with mycolactone in the presence or absence of bafilomycin A1 (figure 5C), suggests a block of autophagy at the terminal stages [39]. Furthermore, the higher LC3-I levels observed in cells exposed to mycolactone, when compared with cells where autophagy was induced by rapamycin (figure 5C), suggests that most probably autophagy is being induced due to cell detachment [40] or as a feedback response to the blockage of the autophagic terminal stage. These data indicate that mycolactone inhibits autophagosome-lysosome fusion and in turn impairs autophagy.

Taken together, this reveals that mycolactone mediates up-regulation of the UPS and inhibition of autophagy. Since autophagy counteracts several stresses, including infection by intracellular pathogens [41], [42], [43], mycolactone-induced impairment of autophagy may have implications for the progress of *M. ulcerans* infection.

### Extracellular matrix: Mycolactone impairs collagen biosynthesis

Proteomics identified several enzymes of collagen biosynthesis progressively down-regulated in mycolactone-treated cells: Plod1 (24 h), Plod3 (48 h) and two isoforms of P4ha1 (48 h+48 h). Further studies showed that these proteins were transcriptionally down-regulated after 24 h of mycolactone exposure (figure S3), thus the differential down-regulation of the different proteins probably reflects different protein stability. These enzymes catalyze the hydroxylation of lysine (Plod1 and Plod3) and proline (P4ha1) residues, which is essential for the formation and stabilization of collagen fibers [44], [45]. The here detected down-regulation of these enzymes suggests that collagen fibers stability may be compromised in mycolactone exposed cells. Interestingly, histopathological studies from the 1960's reported a collagen decrease in human BU lesions [5], [46], [47]; however this feature has been overlooked and it was never subject to studies to determine its cause or its relevance for BU.

**Figure 6:**
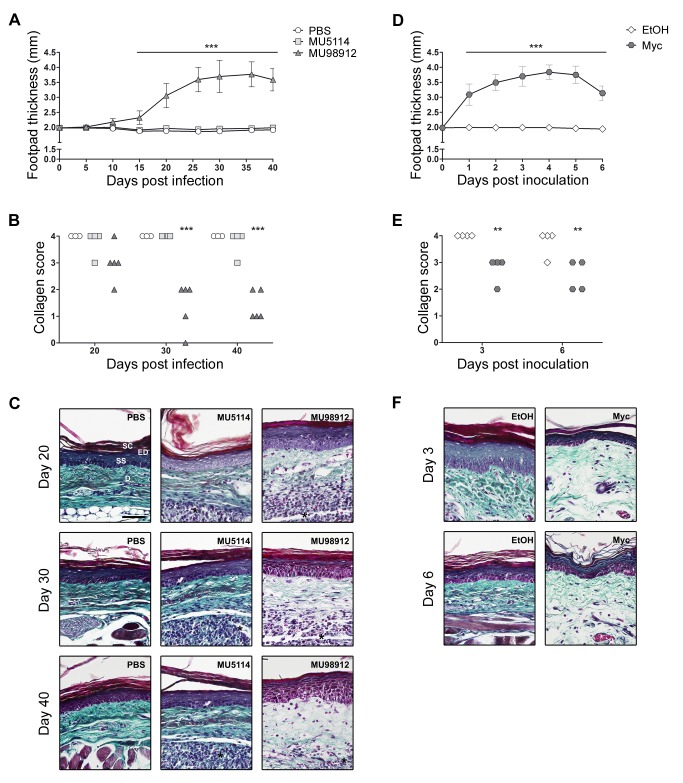
Mycolactone induces a decrease in dermal collagen fibers. Mice were infected with virulent mycolactone-secreting (MU98912: dark gray triangles) (n = 5) or avirulent mycolactone-negative (MU5114: light gray squares) (n = 4) strains of *M. ulcerans*, or injected with PBS (vehicle: white circles) (n = 3) as a control (A, B and C). Alternatively, mice were inoculated with 5 µg of mycolactone (dark gray hexagons) (n = 4) or ethanol (vehicle: white diamonds) (n = 4) as a control (D, E, F). Lesion progression was assessed by measurement of footpad swelling (A and D). Collagen content was assessed by a qualitative blind scoring of the amount of dermal collagen fibers (score 0 = lowest, 4 = highest) visualized in HE-stained sections by polarized light (B and E). The different groups were compared to the control groups (PBS in A and B; ethanol in C and D) by Two-way ANOVA with Bonferroni posttest; statistical differences were represented by ** (P<0.01) or *** (P<0.001). Additionally, mouse footpads exposed to the different stimuli were stained with Masson's trichrome to highlight collagen fibers (green) (C and F). On panel are depicted representative histological image each group. Black horizontal bars represent a 50 µm scale. Histological structures like dermis (D) and the stratum corneum (SC) and stratum spinosum (SS) of the epidermis (ED) are indicated on the upper-left image. Inflammatory infiltrate is indicated by an asterisk.

To investigate if mycolactone was responsible for a decrease in tissue collagen, an experimental model of BU disease, the mouse footpad infection with *M. ulcerans*, was used. Mice were challenged with virulent mycolactone-secreting (MU98912), avirulent mycolactone-negative (MU5114) strains of *M. ulcerans* or PBS as a control. Pathology progression was assessed by measuring footpad swelling (figure 6A) and, at different time-points, footpads were collected for histological processing and collagen scoring (figure 6B). The results showed that, in footpads infected with mycolactone-secreting *M. ulcerans* (MU98912), the progressive increase of pathology (figure 6A) was associated with a decrease of the collagen score (figure 6B), preceding the breakdown of the lesion into an ulcer (by day 40 post-infection). In contrast, infection with the mycolactone-negative strain (MU5114) did not induce pathology nor did it alter the collagen content in infected footpads, similar to what was observed for the PBS-injected control group (figure 6A and 6B). These results suggest that the decrease in collagen content is not a consequence of the infection or the elicited immune response, but rather caused by mycolactone. To verify this, mice were challenged with purified toxin or ethanol equivalent as control. The results showed that mycolactone induced footpad swelling associated with a decrease of the collagen score, while the vehicle did not (figure 6D and 6E). Histological samples stained with Masson's trichrome showed a decay of collagen fibers in MU98912-infected or mycolactone-treated footpads, characterized by the disorganization and thinness of collagen fibers (figure 6C and 6F).

These results in the mouse model of infection show that the earlier described degeneration of collagen in BU lesions [5], [46], [47], [48] is a consequence of the secretion of mycolactone by the infecting strain.

## Discussion

Even though, the methodology used has some limitations, since it excludes the analysis of transmembrane and secretory proteins, which were found to be severely affected by mycolactone [28], this work is the first proteomic study on the effect of mycolactone on cells, unveiling important information about the toxin action.

It has been known for years that the actin-cytoskeleton of mycolactone-treated cells suffers early structural rearrangements [26]. Recently, it was also shown that these changes were mediated by the mycolactone-induced hyperactivation of the actin-cytoskeleton regulator WASP [27]. In this study we show that mycolactone causes structural changes in microtubules and we identify several regulators and structural components of both microfilaments and microtubules affected by the *M. ulcerans* toxin. These data confirm the cytoskeleton as a major target of mycolactone and further specifies the mechanisms of the toxin's cellular action. Moreover, given the cytoskeleton's dynamic nature, with constant remodeling, it remained unclear how these changes contribute to the tissue damage characteristic of BU lesions. Since the proteomic data pointed likewise to an involvement of the UPS further experiments were performed confirming its mycolactone-dependent up-regulation. UPS and autophagy constitute the main intracellular processes of protein degradation taking part in the cellular protein quality control system. Thus, UPS and autophagy are critical in the maintenance of cellular homeostasis and their activities are strictly orchestrated. Moreover, perturbations in the flux through either pathway have been reported to affect the activity of the other system, and a number of mechanisms have been proposed to rationalize the link between the UPS and autophagy [49]. Therefore, it was investigated if the detected mycolactone-dependent changes affect autophagy. The here obtained data indicate that autophagosome-lysosome fusion is impaired in mycolactone-treated cells. Given that the delivery of autophagosome contents to lysosomes is dependent on microtubule cytoskeleton [35] and on dynein-driven transport [36], the mycolactone-induced impairment of autophagy appears to occur secondarily to mycolactone-induced cytoskeleton alterations. Further evidence of a dysfunctional vesicle-lysosome fusion is given by another altered protein in mycolactone-treated cells. Proteomics revealed that the cell culture medium constituent BSA (clustered on miscellaneous proteins) was increased in cells treated with mycolactone. As degradation of extracellular proteins occurs in lysosomes [50], the observed BSA accumulation suggests likewise that the endosomes-lysosome fusion may be compromised. Since the delivery of autophagosome to lysosomes [36] and retrograde transport of endosomes [31] is mediated by dynein the here observed down-regulation of one of its components (Dync1i2), suggest an impairment of dynein-driven transport upon mycolactone exposure. Further work is needed to explore the effect of mycolactone on cytoskeletal motors-mediated transport, however the down-regulation of a molecular motor subunit (Dync1i2) together with the microfilaments' and microtubules' architectural changes, induced by the toxin, hint at a dysfunctional cytoskeletal motors-mediated transport within mycolactone-treated cells. Other cytoskeleton dependent functions, like phagocytosis [51], [52], cell motility [27] and cell shape [17] are also described to be impaired in mycolactone treated cells. Thus, these evidences imply that mycolactone induces a nonfunctional cytoskeletal-architecture, affecting cytoskeleton-dependent functions, with consequences for cellular homeostasis. Moreover, our proteomic study revealed several regulators and structural constituents of both actin- and tubulin-cytoskeleton (Cfl1, Crmp2, Stmn1 and Actg1) affected by mycolactone. These alterations may reflect a cell feedback response to the abnormal cytoskeletal architecture as an attempt to restore the physiological cytoskeletal conformation and dynamics. In particular, the early alterations found on cofilin, a well-known regulator of actin dynamics [32], and on dynein, recently found to play a role in the production of normal bundled stress fibers [53], may represent an immediate cellular response to actin polymerization mediated by mycolactone-induced WASP hyperactivation [27]. Thus, a growing body of evidences supports a model in which the cytoskeletal disarrangement induced by mycolactone impairs multiple cytoskeleton-dependent cellular functions with cytotoxic consequences for the host cells.

These cytoskeletal changes might have also implications early on infection, during the *M. ulcerans* intracellular phase, when the pathogen has to survive and proliferate inside the host cell [3], [4]. Autophagy is being increasingly recognized as an important component of immunity, playing specific roles in shaping the immune system development, fuelling host innate and adaptive immune responses, and directly controlling intracellular microbes as a cell-autonomous innate defense mechanism. As an evolutionary counterpoint, intracellular pathogens have evolved to block autophagic microbicidal defenses and subvert host autophagic responses for their survival or growth [41], [42]. Importantly, studies have implicated autophagy in the control of many pathogenic bacteria [43], from which *M. tuberculosis* [54] should be highlighted here due to its genetic proximity to *M. ulcerans*. Thus, the mycolactone-induced impairment of autophagy, mediated by its action over the cytoskeleton, might represent a virulence mechanism of *M. ulcerans* to impair host cell immunity against intracellular pathogens.

One of the main findings of this work is the identification of a novel activity of mycolactone, with the demonstration of its role in the decrease of collagen content in *M. ulcerans*-infected tissues. Collagen decrease in human BU lesions was described in the 1960's, in the first histopathological studies of this disease [5], [46], [47], however, this phenomenon has been overlooked, even when, more recently, Guarner et al. described this feature as one of the most reliable criteria for the histopathological diagnosis of BU [48]. This previously unappreciated feature of the disease was never subject to studies to determine its cause or its relevance for BU progression and associated sequelae.

Here we link, for the first time, the activity of mycolactone with the collagen reduction in *M. ulcerans*-infected tissues. Our results from the *in vivo* model show that collagen decrease is not a consequence of the infection or the immune response, but of the presence of mycolactone. In fact the inoculation of the purified toxin shows an association between decrease of collagen and the presence of mycolactone. *In vitro*, we showed that in L929 cells mycolactone transcriptionally down-regulated several ER-resident collagen-modifying enzymes (Plod1, Plod3 and two isoforms of P4ha1). Additionally, Hall et al. described a post-transcriptional mechanism in which mycolactone blocks co-translational translocation of proteins into the ER, thus inhibiting the synthesis of the majority of ER-resident (like the collagen-modifying enzymes) and secretory proteins (like extracellular matrix proteins) [28]. Although these mechanisms have not been verified *in vivo*, altogether, these data suggest that mycolactone inflicts a transcriptional and post-transcriptional inhibition of the collagen biosynthesis pathway, which translate into a degeneration of collagen fibers in mycolactone-exposed tissues.

Our data also show that the mycolactone-induced collagen degeneration precedes the breakdown of the lesion into an ulcer, suggesting that collagen decrease may be involved, together with cell death, in the tissue destructuration that culminates in the emergence of an *M. ulcerans*-induced ulcerative lesion. Moreover, it may be a mechanism of pathogen dissemination, given that in early lesions bacteria concentrate in a smaller central zone, while in advanced lesions bacilli are dispersed throughout the necrotic area. Finally, this collagen decay in BU lesions may also be implicated in the development of the sequelae characteristic of this devastating skin disease. BU has a very high morbidity rate associated with contractures [9]. Wound contraction is a natural mechanism by which open wounds close during the healing process, but also results in significant tissue distortion with loss of joint mobility and cosmetic disfigurement. Although the mechanism of wound contraction is not fully understood, it is associated with the abnormal generation of thicker collagen fibers [55], [56], [57]. Therefore, it is conceivable that during the healing process, fibroblasts and myofibroblasts repopulating the lesion overcome the collagen-deficiency through abnormal- or over-production of collagen leading to the extreme contractures characteristic of BU [58]. In fact, a recent paper by Andreoli et al. described an increase in activated myofibroblasts and an abundant production of extracellular matrix proteins in antibiotic-treated BU lesions [59]. Further studies are needed to test this hypothesis, but if proven correct the use of collagen-based materials as a bed for the skin graft, or even as a replacement in smaller legions, may decrease the contracture and thus the morbidity in BU patients.

Overall, our results provide molecular and functional evidence of the impact of mycolactone on the cytoskeleton and cytoskeleton-dependent cellular functions, and extend our knowledge on the action of the *M. ulcerans* toxin to collagen biosynthesis, providing new perspectives on BU pathogenesis and paving the way for future therapeutic approaches.

## Materials and Methods

### Bacteria

*M. ulcerans* strains were selected from the Institute of Tropical Medicine collection in Antwerp, Belgium. MU5114 is a mycolactone-negative strain due to repeated subculturing, leading to the spontaneous loss of genes involved in mycolactone synthesis [23], [60]. MU98912 is highly virulent for mice [22] and produces mycolactone type D [24]. The isolates were grown on Middlebrook 7H9 medium (Becton, Dickinson and Company) with 1.5% of agar at 32°C for approximately 6–8 weeks. For the preparation of the inoculum, *M. ulcerans* was recovered, vortexed using glass beads and diluted in phosphate-buffered saline pH 7.4 (PBS) to a final concentration of 1 mg/ml.

### Mycolactone extraction/purification

Protocol for mycolactone extraction/purification was adapted from the one previously described [17]. Briefly, MU98912 was cultured in Dubos medium supplemented with 10% oleic acid-albumin-dextrose complex, at 32°C. At late exponential growth phase, bacteria were harvested and lipids were extracted with chloroform and methanol (2∶1) for 4 hours. The organic phase was separated from bacterial debris and hydrophilic components by addition of a 0.2 volume of water, followed by centrifugation. The organic phase was dried and resuspended in ice-cold acetone. The individual lipid components of the acetone-soluble lipid fraction were separated by chromatography using the CycloGraph system (Analtech). The separated fractions were analyzed by thin layer chromatography, and the fractions corresponding to mycolactone were pooled, dried down, weighed, resuspended in absolute ethanol, and stored at −80°C under nitrogen atmosphere in the dark [61]. Purified mycolactone was analyzed by mass spectrometry (MS detector Thermo LxQ linear ion trap) and the presence of mycolactone D confirmed. Under these conditions, mycolactone was stable for at least three years.

### *In vitro* experimental design

Mouse fibroblasts L929 cell line was cultured in Dulbecco's Modified Eagle Medium (DMEM) (Gibco) supplemented with 10% fetal bovine serum (Gibco), 2 mM L-glutamine (Gibco), 10 mM HEPES (Gibco), 1 mM sodium pyruvate (Gibco) and antibiotic-antimycotic (Gibco). Cells were expanded in 175 cm^2^ flasks (Nunc) until 90% confluence. Then, cells were plated in 12-wells plates (Nunc) at a density of 2.5×10^5^ cells/well, with increasing concentrations of mycolactone or with ethanol equivalent (<0.002%), as a control. Rapamycin (Calbiochen) and Bafilomycin A1 (Sigma) was used to induce autophagy and to block autolysosome degradation, respectively.

### Cell cycle analysis

At each time-point, cells were collected and a pool of adherent and suspended cells was made. Cells were rinsed and resuspended in PBS. Absolute ethanol was gently added until 70% final concentration. Cells were stored in this fixing solution at 4°C. When all time-points had been collected, cells were rinsed in PBS and incubated with staining solution (0.1% triton-X-100, 20 µg/mL of propidium iodide, 250 µg/mL of RNase in PBS) for one hour in a bath at 50°C, in the dark. Samples were analyzed by flow cytometry (LSRII, BD).

### Annexin-V/PI assay

The protocol was done according to the manufacturer's instructions (BD Pharmingen). At each time-point cells were collected and a pool of adherent and suspended cells was made. Cells were rinsed, stained and analyzed by flow cytometry (LSRII, BD).

### Cytoskeleton imaging

L929 cells were allowed to adhere to coverslips (Nunc) overnight following the incubation in different conditions. Cells were rinsed and fixed in paraformaldehyde, for 1 hour, at room temperature. Cells were rinsed and stored in PBS at 4°C. When all time-points had been collected, cells were blocked with blocking solution (5% BSA, 0.1% triton-X-100, 0.1% tween-20 in PBS) and incubated overnight at 4°C with the mouse anti-tubulin antibody (AA4.3, developed by C. Walsh and obtained from the Developmental Studies Hybridoma Bank, developed under the auspices of the National Institute of Child Health and Human Development and maintained by The University of Iowa, Department of Biology). Cells were rinsed and incubated with secondary AF488 goat anti-mouse antibody (Invitrogen) and rhodamine-phalloidin conjugate (Invitrogen) for 1 hour, at room temperature, in the dark. Cells were visualized using a confocal microscope (FV1000, Olympus) with ×60 objective. 3D remodeling was performed using Fluoview software (Olympus).

### Protein extraction

At each time-point, cells were collected and a pool of adherent and suspended cells was made. Cells were rinsed with PBS, resuspended in Lysis Buffer (50 mM Tris-HCl pH7.2, 250 mM NaCl, 2 mM EDTA, 1% NP-40, 10% Glycerol, protease inhibitor (Roche #11873580001) and phosphatase inhibitor (Roche #04906837001)) and stored at −80°C, until protein was extracted at the end of the experiment. When all time-points had been collected, samples were thawed, incubated for 30 minutes at 4°C with agitation, sonicated in a ultrasonic ice cold bath for 1 minute until no agglomerate was seen and centrifuged (30 minutes, 14000 rpm, 4°C). The supernatant was considered the total protein extract. For Western Blot analysis, protein concentration was determined (Thermo Scientific #23227) and aliquots stored at −80°C.

### Two-dimensional gel electrophoresis (2-DE)

Protein was precipitated in 80% (v/v) acetone and the protein pellet resuspended in urea buffer (7 M urea, 2 M thiourea, 4% (w/v) CHAPS, 0.15% (w/v) DTT, 0.5% [v/v] carrier ampholytes and Complete Mini protease inhibitor cocktail). The protein separation was done as previously described [62]. Briefly, 100 µg protein extract was diluted with urea buffer to a final volume of 420 µL and in-gel rehydration was performed overnight. IEF was carried out in IPG strips (pH 3–10, non-linear, 18 cm; GE Healthcare, Uppsala, Sweden) with the Multiphor II system (GE Healthcare) under paraffin oil for 55 kVh. SDS-PAGE was done overnight in polyacrylamide gels (12.5% T, 2.6% C) with the Ettan DALT II system (GE Healthcare) at 1–2 W per gel and 12°C. The gels were silver stained and analyzed with the 2-DE image analysis software Melanie 3.0 (Gene-Bio, Geneva, Switzerland). To verify the reproducibility three biological replicates for each time point and condition as well as three technical replicates were analyzed (all analyzed gels are in Supplementary Information). An expression change was considered significant if the intensity of the corresponding single spot differed reproducibly more than twofold and was reproducible for all three experiments. The expected spot position in the 2D-gel according to the known protein sequence was calculated with the Compute p*I*/Mw tool (http://ca.expasy.org/tools/pi_tool.html). For the detection of phosphorylated proteins 400 µg of protein were separated by 2-DE, stained with Pro-Q Diamond Phosphoprotein Gel Stain (Molecular Probes), according to the manufacturer's instructions, scanned to detect the phosphorylation signals, silver stained and rescanned. Images of both scans were matched with the 2-DE image analysis software Melanie 3.0 (Gene-Bio).

### Mass spectrometry (MS)

For the protein identification, 400 µg of protein were separated by 2-DE. Selected spots were excised, digested with trypsin (recombinant; Roche), and prepared as described earlier [62]. In brief, the extracted and dried peptides were dissolved in 5 µl alpha-Cyano-3-hydroxycinnamic acid (98%, recrystallized from ethanol-water, 5 mg/ml in 50% acetonitrile and 0.1% TFA) and 0.5 µl applied onto the sample plate using the dried-droplet method. Peptide masses were measured with a UltrafleXtreme MALDI-TOF/TOF (Bruker, Billerica, MA, USA). Proteins were identified according to their spot-specific peptide mass fingerprint and/or peptide sequence with the bioinformatic tool BioTools Version 3.2 (Bruker) with the following search parameters (tolerance: MS = 10–50 ppm, MS/MS = 0.5–0.9 Da, enzyme: Trypsin, engine: Mascot, database: NCBInr, modifications: Oxidation (M)). A protein identification was accepted if at least three major peaks matched to the protein with the highest score (full MS and MS/MS data in Supplementary Information).

If the protein spot was detected at a lower molecular mass than expected, suggesting processing or fragmentation, the spot-specific peptides in the mass spectrum were also analyzed to confirm which parts of the corresponding protein sequence matched with these peptides. If the mass spectrum of the spot lacked peptides observed for the complete protein and had a different position in the 2D gel than expected it was indicated as a protein fragment. Therefore, both the spot position observed by 2-DE and the specific peptides in the corresponding mass spectrum were analyzed to indicate a putative protein fragment.

### Western blot analysis

40 µg of protein were resolved in a 12% SDS-PAGE and transferred to the 0.2 µm Nitrocellulose membranes (Bio-Rad #170-4159) with the semi-dry Trans-Blot Turbo system (Bio-Rad). Membranes were blocked and subjected to immunoblotting with GAPDH antibody (CellSignaling #2118), LC3A/B antibody (CellSignaling #4108) or mono- and polyubiquitinylated conjugates antibody (Enzo Life Sciences #PW8810), followed by incubation with horseradish peroxidase linked secondary antibodies (Southern Biotech). Bands were detected with SuperSignal (Thermo Scientific #34095) in a Universal Hood II (Bio-Rad) and quantified with QuantityOne (Bio-Rad). GAPDH was used as loading control.

### LC3 Immunofluorescence

At each time-point, cells were collected and a pool of adherent and suspended cells was made. Cytospins were made (Cytospin III, Shandon) and cells were fixed in paraformaldehyde for 20 minutes at room temperature and stored in ethanol 96% at 4°C. When all time-points had been collected, cells were blocked with blocking solution (5% BSA, 0.1% triton-X-100, 0.1% tween-20 in PBS) and incubated overnight at 4°C with the LC3A/B antibody (CellSignaling #4108). Normal Rabbit IgG Control (R&D Systems AB-105-C) was used as isotype control. Cells were rinsed and incubated with secondary AF568 goat anti-rabbit antibody (Invitrogen) for 1 hour, at room temperature, in the dark. Cells were visualized using a confocal microscope (FV1000, Olympus) with ×60 objective.

### *In vivo* experimental design

Eight-weeks-old female BALB/c mice were obtained from Charles River (Barcelona, Spain) and housed under specific-pathogen-free conditions with food and water *ad libitum*. Mice were infected in the left hind footpad with 30 µL of *M. ulcerans* suspensions with 4.8 log_10_ AFBs, or 30 µL PBS as control. Footpad thickness was evaluated every 2–3 days. Mice were sacrificed weekly and footpads were harvested for histological studies.

### Ethics statement

The *in vivo* studies were approved by the Portuguese national authority for animal experimentation *Direção Geral de Veterinária* (ID: DGV 594 from 1^st^ June 2010). Animals were kept and handled in accordance with the guidelines for the care and handling of laboratory animals in the Directive 2010/63/EU of the European Parliament and of the Council.

### Collagen assessment

Footpads were harvested, fixed in 10% phosphate-buffered formalin and embedded in paraffin. Tissue sections were stained with hematoxylin and eosin (H&E), analyzed by light microscopy with polarized light and the amount of dermal collagen fibers was blindly scored from 0 (lowest) to 4 (highest) independently by two persons in two independent experiments. Additionally, tissue sections were stained with Masson's trichrome and pictures were taken in a light microscopy.

### RT-qPCR

At each time-point, cells were collected and a pool of adherent and suspended cells was made. Cells were rinsed, resuspended in TRIzol Reagent (Ambion) and stored at −80°C, until total RNA was extracted, at the end of the experiment, according to the manufacturer's protocol.

Reverse transcription was done with whole RNA using RevertAid H Minus First Strand cDNA Synthesis Kit (Fermentas) according to the manufacturer's instructions. qPCR was perform on the C1000TM Thermo Cycler (Bio-Rad) using TaqMan Gene Expression Assay (AB Applied Biosystems) (*Plod1*: Mm01255769_m1; *Plod3*: Mm00478798_m1; *P4ha1*: Mm00803137_m1; *B2m*: Mm00437762_m1; *Gapdh*: Mm99999915_g1; *Hprt*: Mm00446968_m1). Relative quantification was determined with CFX Manager Software (Bio-Rad) using *B2m*, *Gapdh* and *Hprt* as reference genes.

### Statistical analysis

Differences between the means of experimental groups were analyzed using the Prism version 5.0 software (GraphPad). Percentage and fraction values were transformed to and analyzed as arcsin values. Differences were considered significant only with a *P* value<0.001, in the *in vitro* studies; or with a *P* value<0.01, in the *in vivo* study.

## Supporting Information

Figure S1 **Mycolactone treatment affects mainly cytoskeleton related proteins and collagen biosynthesis enzymes.** Enlarged parts of silver-stained 2-D gel of total protein extracts (100 µg) of L929 cells treated either with ethanol or mycolactone (50 ng/mL), showing the identified altered spots clustered into five categories. Arrows and circles represent conditions where the spot intensity is increased or decreased, respectively. Phosphoproteins are indicated by an asterisk (*) and putative protein fragments are indicated by a number sign (#). For P4ha1 the number of the specific isoform is indicted in parentheses.
(TIF)

Figure S2 **Spots 4 and 6 are phosphoproteins.** Enlarged parts of silver- and phospho-stained 2-D gel showing the spot 4 (Cfl1) and the spot 6 (Crmp2) in both stainings.
(TIF)

Figure S3 **Mycolactone treatment induces a transcriptional down-regulation of collagen biosynthesis enzymes.** Mouse fibroblasts L929 cells were incubated for 24 or 48 hours either with ethanol (white) or mycolactone (50 ng/mL, gray). Additionally, an assay was performed where cells were incubated for 48 hours in the same conditions followed by a 48 hour incubation period in fresh medium (referred to as 48 h+48 h). At each time-point, total RNA was extracted and *Plod1*, *Plod3*, *P4ha1* mRNA levels assessed. Bars represent the mean + SD from two independent experiments with three technical replicas (n = 6). Mycolactone-treated was compared to EtOH-treated samples throughout each time-point (24 h and 48 h) by Two-way ANOVA with Bonferroni posttest; statistical differences were represented by *** (*P*<0.001). Each condition at 48 h+48 h time-point was compared with the same condition at the 48 h by Two-way ANOVA with Bonferroni posttest; statistical differences were represented by ^&&&^ (*P*<0.001).
(TIF)

Dataset S1 **2D gels from L929 cells incubated for 24 hours with ethanol.**
(ZIP)

Dataset S2 **2D gels from L929 cells incubated for 48 hours with ethanol.**
(ZIP)

Dataset S3 **2D gels from L929 cells incubated for 48 hours with ethanol followed by a 48 hour incubation period in fresh medium (referred to as 48 h+48 h).**
(ZIP)

Dataset S4 **2D gels from L929 cells incubated for 24 hours with mycolactone.**
(ZIP)

Dataset S5 **2D gels from L929 cells incubated for 48 hours with mycolactone.**
(ZIP)

Dataset S6 **2D gels from L929 cells incubated for 48 hours with mycolactone followed by a 48 hour incubation period in fresh medium (referred to as 48 h+48 h).**
(ZIP)

Dataset S7 **MS and MS/MS data.**
(ZIP)
