# Supplementary figures and images for: Proteomic Analysis of the Action of the Mycobacterium ulcerans Toxin Mycolactone: Targeting Host Cells Cytoskeleton and Collagen
Source: PLoS Negl Trop Dis. 2014 Aug 7;8(8):e3066. doi: 10.1371/journal.pntd.0003066 (PMC4125307; doi:10.1371/journal.pntd.0003066)

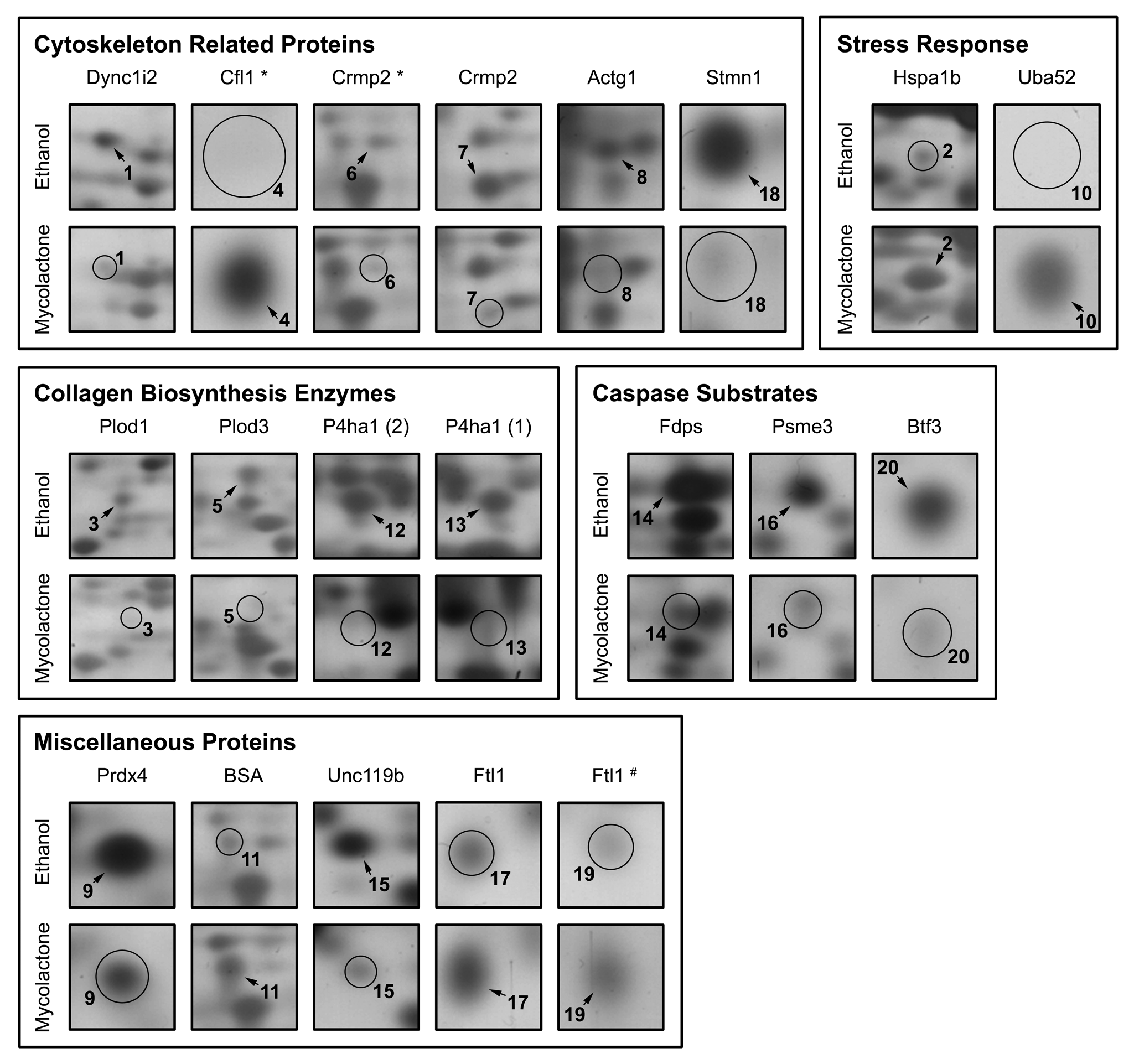

Supplement: Figure S1 — Mycolactone treatment affects mainly cytoskeleton related proteins and collagen biosynthesis enzymes. Enlarged parts of silver-stained 2-D gel of total protein extracts (100 µg) of L929 cells treated either with ethanol or mycolactone (50 ng/mL), showing the identified altered spots clustered into five categories. Arrows and circles represent conditions where the spot intensity is increased or decreased, respectively. Phosphoproteins are indicated by an asterisk (*) and putative protein fragments are indicated by a number sign (#). For P4ha1 the number of the specific isoform is indicted in parentheses. (TIF) [file pntd.0003066.s001.tif]

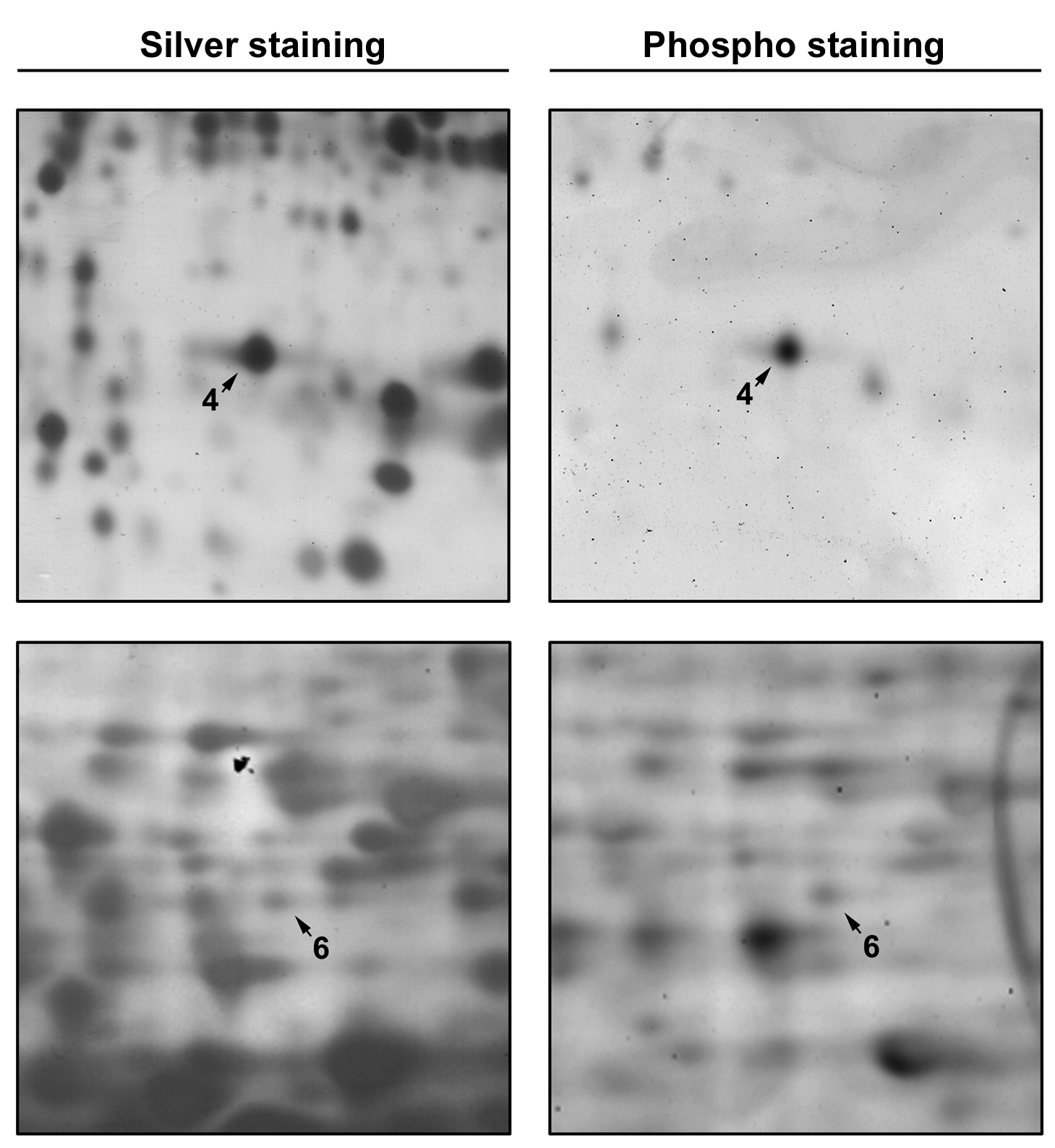

Supplement: Figure S2 — Spots 4 and 6 are phosphoproteins. Enlarged parts of silver- and phospho-stained 2-D gel showing the spot 4 (Cfl1) and the spot 6 (Crmp2) in both stainings. (TIF) [file pntd.0003066.s002.tif]

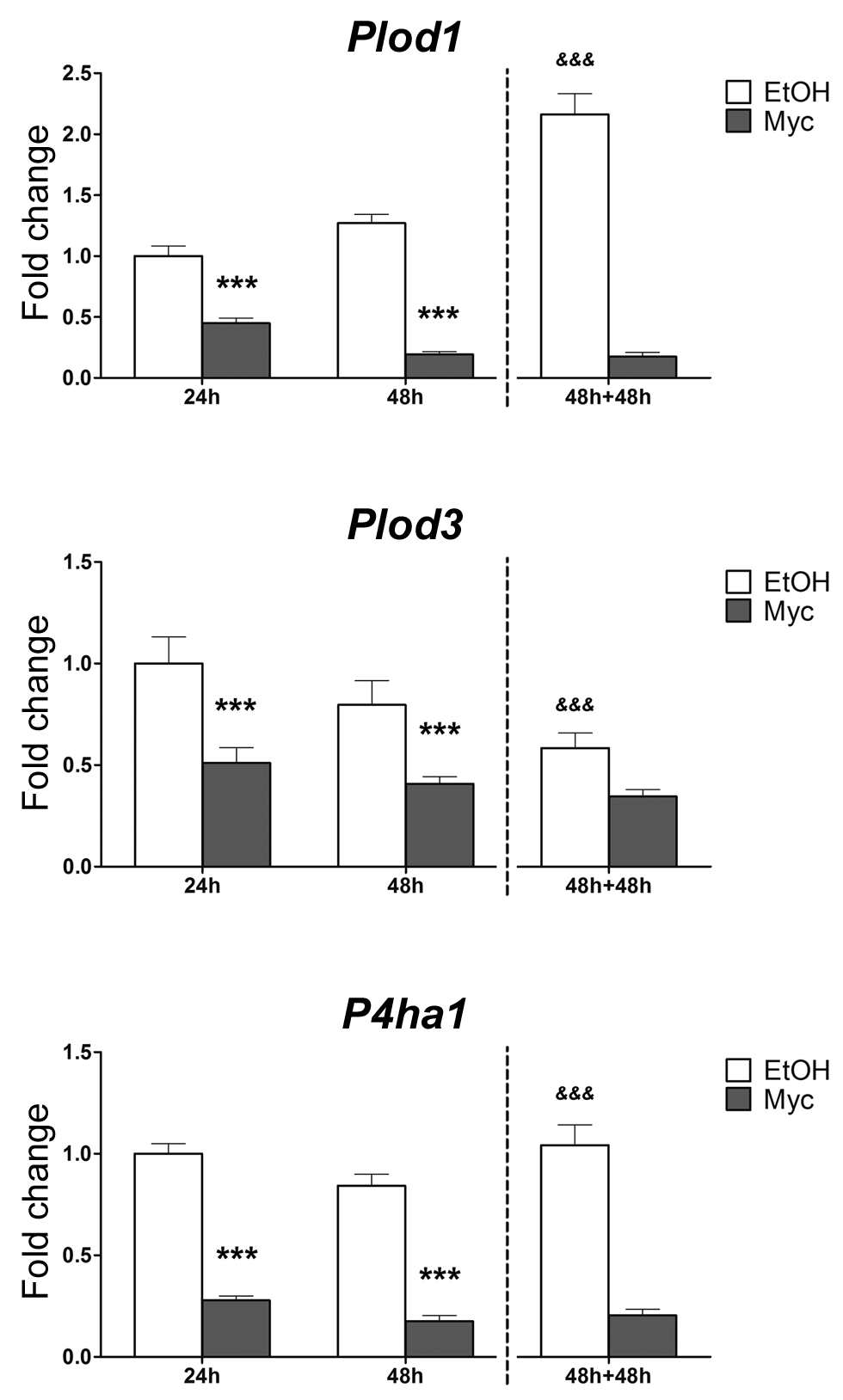

Supplement: Figure S3 — Mycolactone treatment induces a transcriptional down-regulation of collagen biosynthesis enzymes. Mouse fibroblasts L929 cells were incubated for 24 or 48 hours either with ethanol (white) or mycolactone (50 ng/mL, gray). Additionally, an assay was performed where cells were incubated for 48 hours in the same conditions followed by a 48 hour incubation period in fresh medium (referred to as 48 h+48 h). At each time-point, total RNA was extracted and Plod1, Plod3, P4ha1 mRNA levels assessed. Bars represent the mean + SD from two independent experiments with three technical replicas (n = 6). Mycolactone-treated was compared to EtOH-treated samples throughout each time-point (24 h and 48 h) by Two-way ANOVA with Bonferroni posttest; statistical differences were represented by *** (P<0.001). Each condition at 48 h+48 h time-point was compared with the same condition at the 48 h by Two-way ANOVA with Bonferroni posttest; statistical differences were represented by &&& (P<0.001). (TIF) [file pntd.0003066.s003.tif]
