# Supplementary material for: Proteomic Analysis of the Action of the Mycobacterium ulcerans Toxin Mycolactone: Targeting Host Cells Cytoskeleton and Collagen
Source: PLoS Negl Trop Dis. 2014 Aug 7;8(8):e3066. doi: 10.1371/journal.pntd.0003066 (PMC4125307; doi:10.1371/journal.pntd.0003066)
Supplement: Dataset S7 — MS and MS/MS data. (ZIP) [file pntd.0003066.s010.zip › MS Data/Spot 01 - Dync1i2.pdf]

D:\Data\Bernardo\2011_07_25\C5_2_29\0_M5\1\1SRef

Comment 1

Comment 2

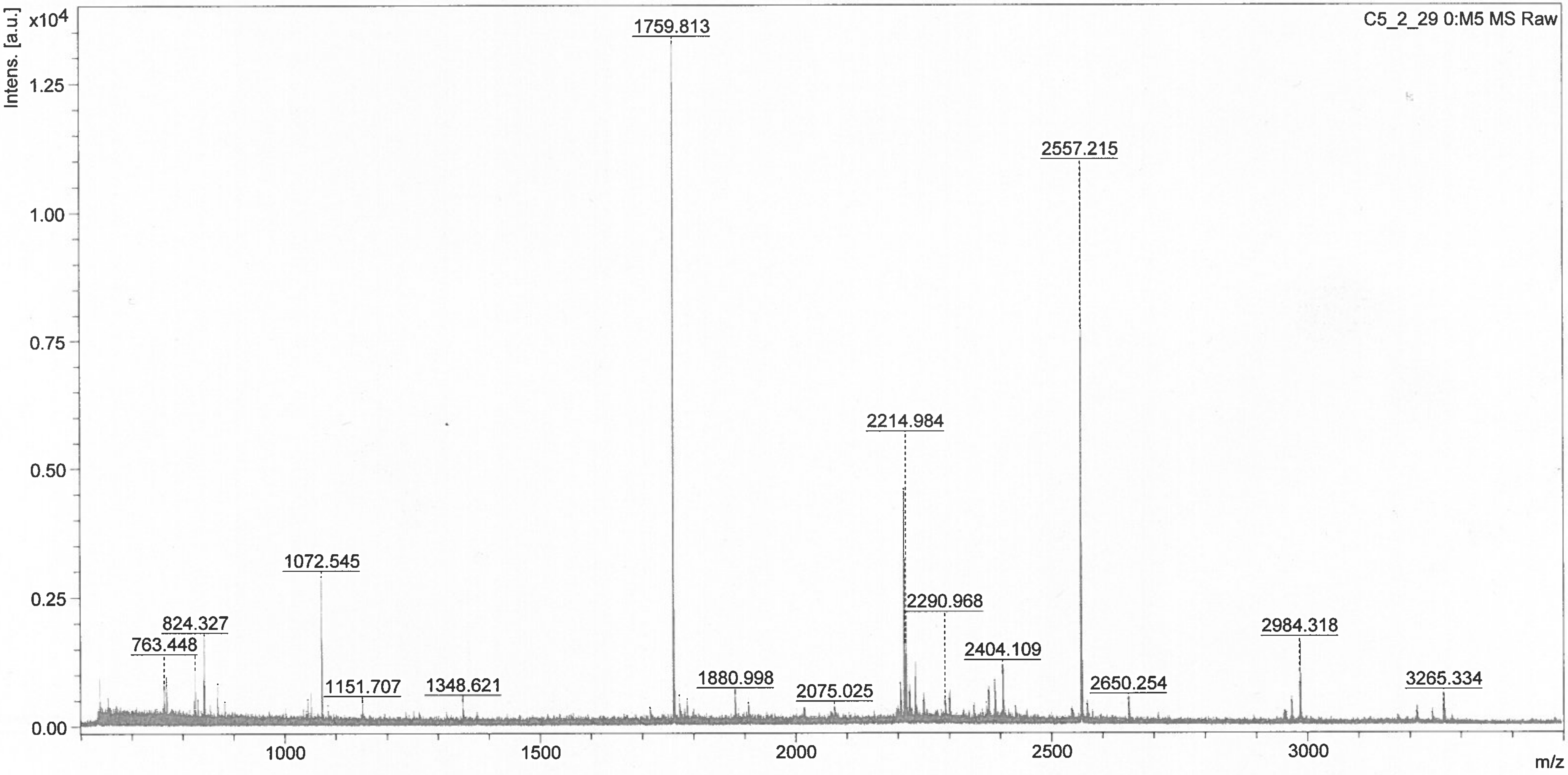

Bruker Daltonics flexAnalysis

printed: 7/26/2011 9:09:40 AM

Spectrum Analysis Report

Date: 07/29/2011 Time: 06:44

FileName: D:\Data\Bernardo\2011_07_25\C5_2_29\0_M5\1\1SRef\pdata\1\PMF_LIFT.xml

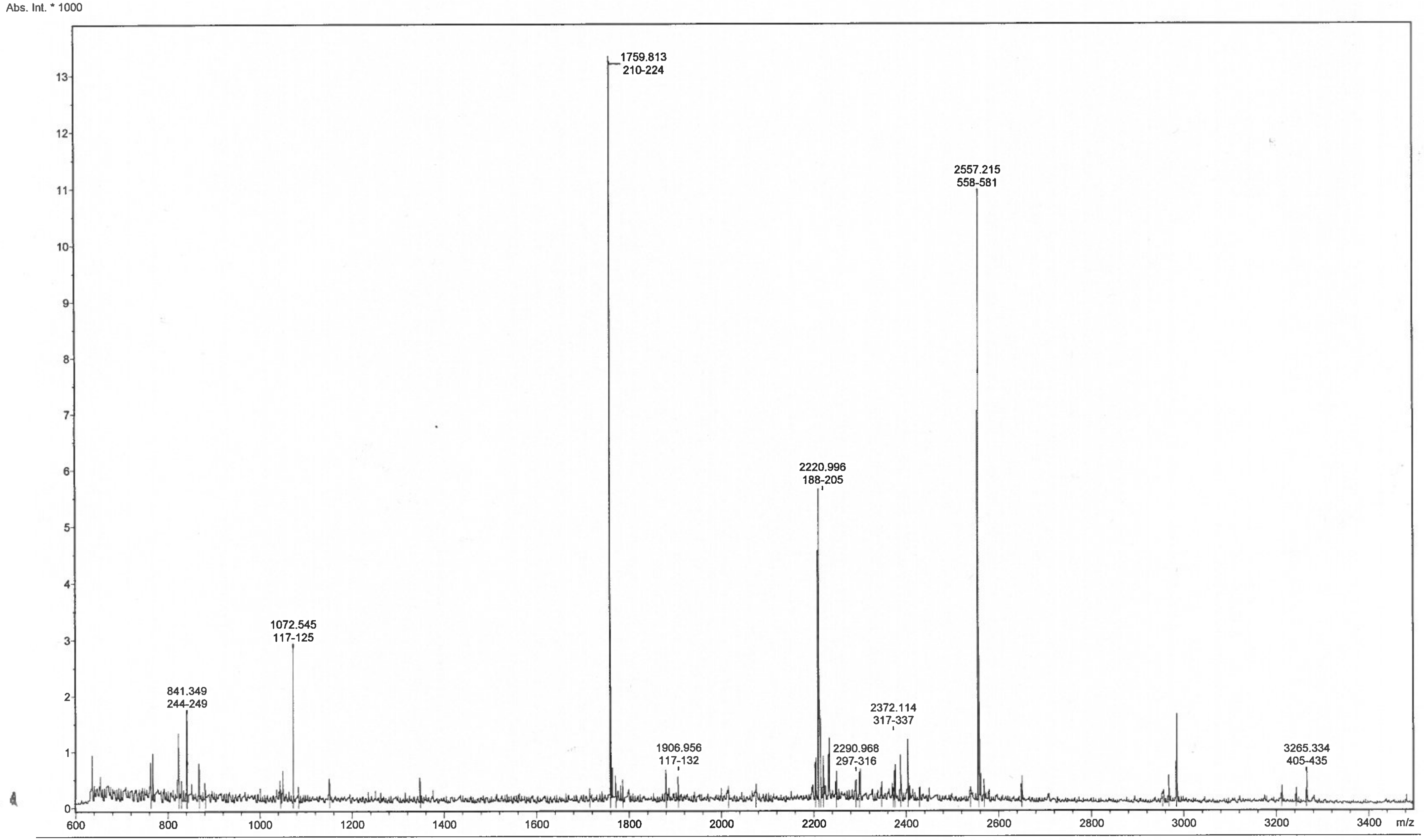

Copyright © 1999-2008 Bruker Daltonik GmbH

Spectrum Analysis Report

Date: 07/29/2011 Time: 06:44

FileName: D:\Data\Bernardo\2011_07_25\C5_2_29\0_M5\1\1SRef\pdata\1\PMF_LIFT.xml

Sequence data:

cytoplasmic dynein 1 intermediate chain 2 isoform 5 [Mus musculus] gi|6753658

Intensity Coverage:	55.0 % (26718 cnts)	Sequence Coverage MS:	24.7%
Sequence Coverage MS/MS:	6.4%	pI (isoelectric point):	5.0

10	20	30	40	50	60	70	80	90	100	110
MSDKSDLKAE	LERKKQRLAQ	IREEKKRKEE	ERKKKETDQK	KEAAVSVQEE	SDLEKKRREA	EALLQSMGLT	TDSPIVPPPM	SPSSKSVSTP	SEAGSQDSGD	GAVGSRRGPI
120	130	140	150	160	170	180	190	200	210	220
KLGMAKITQV	DFPPREIVTY	TKETQTPVTA	QPKEDEEEED	DVATPKPPVE	PEEEKTLKKD	EENDSKAPPH	ELTEEEKQQI	LHSEEFLSFF	DHSTRIVERA	LSEQINIFFD
230	240	250	260	270	280	290	300	310	320	330
YSGRDLEDKE	GEIQAGAKLS	LNRQFFDERW	SKHRVVSCLD	WSSQYPELLV	ASYNNNEEAP	HEPDGVALVW	NMKYKKTTPE	YVFHCQSAVM	SATFAKFHPN	LVVGGTYSGQ
340	350	360	370	380	390	400	410	420	430	440
IVLWDNRSNK	RTPVQRTPLS	AAAHTHPVYC	VNVVGTQNAH	NLISISTDGK	ICSWSLDMLS	HPQDSMELVH	KQSKAVAVTS	MSFPVGDVNN	FVVGSEEGSV	YTACRHGSKA
450	460	470	480	490	500	510	520	530	540	550
GISEMFEGHQ	GPITGIHCHA	AVGAVDFSHL	FVTSSFDWTV	KLWTTKNNKP	LYSFEDNSDY	VYDVMWSPTH	PALFACVDGM	GRLDLWNLNN	DTEVPTASIS	VEGNPALNRV
560	570	580	590	600	610	620				
RWTHSGREIA	VGDSEGQIVI	YDVGEQIAVP	RNDEWARFGR	TLAEINANRA	DAEEEAATRI	PA				

Acquisition Parameter:

Matched Sequences:

Unmatched

Peaks/MSMS Spectra

Tree hierarchy	Meas. M/z	Calc. MH+	Meas. Mr	Calc. Mr	Int.	z	Dev.(Da)	Dev.(ppm)	Score	MascotScore	Rt(min)	Range	P	Sequence
peak 1	763.448	-	762.440	-	709.880	1+	-	-	-	-	-		-	
peak 2	824.327	-	823.319	-	1074.434	1+	-	-	-	-	-		-	
peak 3	829.492	-	828.485	-	378.438	1+	-	-	-	-	-		-	
peak 5	842.477	-	841.469	-	1572.337	1+	-	-	-	-	-		-	
peak 6	868.525	-	867.518	-	690.984	1+	-	-	-	-	-		-	
peak 7	882.542	-	881.535	-	324.257	1+	-	-	-	-	-		-	
peak 8	1045.526	-	1044.518	-	329.652	1+	-	-	-	-	-		-	
peak 10	1084.543	-	1083.536	-	304.761	1+	-	-	-	-	-		-	
peak 11	1151.707	-	1150.700	-	446.598	1+	-	-	-	-	-		-	
peak 12	1348.621	-	1347.614	-	468.102	1+	-	-	-	-	-		-	
peak 13	1714.852	-	1713.844	-	298.237	1+	-	-	-	-	-		-	
peak 15	1771.799	-	1770.791	-	424.493	1+	-	-	-	-	-		-	
peak 16	1786.902	-	1785.895	-	366.196	1+	-	-	-	-	-		-	
peak 17	1880.998	-	1879.990	-	604.016	1+	-	-	-	-	-		-	
peak 19	2015.975	-	2014.968	-	279.537	1+	-	-	-	-	-		-	
peak 20	2075.025	-	2074.017	-	293.699	1+	-	-	-	-	-		-	
peak 21	2203.964	-	2202.957	-	711.500	1+	-	-	-	-	-		-	
peak 22	2211.029	-	2210.021	-	4438.473	1+	-	-	-	-	-		-	
peak 23	2214.984	-	2213.976	-	1593.891	1+	-	-	-	-	-		-	
peak 25	2248.982	-	2247.975	-	420.451	1+	-	-	-	-	-		-	
peak 27	2299.082	-	2298.074	-	524.193	1+	-	-	-	-	-		-	
peak 28	2347.206	-	2346.199	-	300.728	1+	-	-	-	-	-		-	
peak 30	2376.113	-	2375.106	-	563.461	1+	-	-	-	-	-		-	
peak 31	2388.109	-	2387.101	-	737.638	1+	-	-	-	-	-		-	
peak 32	2404.109	-	2403.102	-	991.934	1+	-	-	-	-	-		-	
peak 33	2429.139	-	2428.132	-	239.575	1+	-	-	-	-	-		-	
peak 34	2539.200	-	2538.192	-	250.399	1+	-	-	-	-	-		-	
peak 36	2569.216	-	2568.209	-	319.909	1+	-	-	-	-	-		-	
peak 37	2650.254	-	2649.247	-	359.983	1+	-	-	-	-	-		-	
peak 38	2954.343	-	2953.336	-	178.284	1+	-	-	-	-	-		-	
peak 39	2968.309	-	2967.302	-	301.481	1+	-	-	-	-	-		-	
peak 40	2984.318	-	2983.311	-	1033.410	1+	-	-	-	-	-		-	
peak 41	3212.394	-	3211.387	-	150.281	1+	-	-	-	-	-		-	
peak 42	3243.405	-	3242.398	-	160.579	1+	-	-	-	-	-		-	

Global peptide results

Copyright © 1999-2008 Bruker Daltonik GmbH

unnamed protein product [Mus musculus] gi|74213854
MW:59774.100

MGLTTDSPIVPPPMSPSSKSVSTPSEAGSQDSGDGAVGSRRGPIKLGMAKITQVDFPPREIVTYTKETQTPVTAQPKEDEEEEDDVATPKPPVEPEEEKTLKKDEENDSKAPPHELTEEEKQQILHSEEFLSFFDHSTRIVERALSEQINIFFDYSGRDLEDKEGEIQAGAKLSLNRQFFDERWSKHRVVSCLDWSSQYPELLVASYNNNE
EAPHEPDGVALVWNMKYKKTTPEYVFHCQSAVMSATFAKFHPNLVVGGTYSGQIVLWDNRSNKRTPVQRTPLSAAAHTHPVYCVNVVGTQNAHNLISISTDGKICSWSLDMLSHPQDSMELVHKQSKAVAVTSMSFPVGDVNNFVVGSEEGSVYTACRHGSKAGISEMFEGHQGPITGIHCHAAVGAVDFSHLFVTSSFDWTVKLWTTKNN
KPLYSFEDNSDYVYDVMWSPTHPALFACVDGVGRLDLWNLNNDTEVPTASISVEGNPALNRVRWTHSGREIAVGDSEGQIVIYDVGEQIAVPRNNEWARFGRTLAEINANRADA

Digest Matches (Score: 150.00)
Score = 150.000000, Rank = 1, Database = NCBInr, Accesskey = gi|74213854
Search Parameters: MS Tol.:100.00 ppm, MSMS Tol.: 0.600000 Da, Enz.Trypsin, Engine: Mascot Version: 2.3.01.241 , DB: NCBInr , DB Version: NCBInr_20110715.fasta
Modifications: Optional: Oxidation (M)

Tree hierarchy	Meas. M/z	Calc. MH+	Meas. Mr	Calc. Mr	Int.	z	Dev.(Da)	Dev.(ppm)	Score	MascotScore	Rt(min)	Range	P	Sequence
peak 4	841.349	841.384	840.342	840.377	763.525	1+	-0.035	-41.318	-	-	-	178 - 183	0	QFFDER
peak 9	1072.545	1072.579	1071.538	1071.571	2939.736	1+	-0.033	-30.974	-	-	-	51 - 59	0	ITQVDFPPR
MSMS 14	1759.813	1759.865	1758.805	1758.858	13234.713	1+	-0.052	-29.676	20	-	-	144 - 158	0	ALSEQINIFFDYSGR
peak 18	1906.956	1907.027	1905.949	1906.020	412.740	1+	-0.071	-37.358	-	-	-	51 - 66	1	ITQVDFPPREIVTYTK
peak 24	2220.996	2221.067	2219.989	2220.060	651.559	1+	-0.071	-32.090	-	-	-	122 - 139	0	QQILHSEEFLSFFDHSTR
peak 26	2290.968	2291.047	2289.961	2290.040	394.153	1+	-0.079	-34.413	-	-	-	231 - 250	0	TTPEYVFHCQSAVMSATFAK 14: Oxidation (M) 9: Carbamidomethyl (C)
peak 29	2372.114	2372.215	2371.106	2371.207	280.231	1+	-0.101	-42.574	-	-	-	251 - 271	0	FHPNLVVGGTYSGQIVLWDNR
MSMS 35	2557.215	2557.314	2556.208	2556.307	7714.664	1+	-0.100	-38.975	1043	80	-	492 - 515	0	EIAVGDSEGQIVIYDVGEQIAVPR
peak 43	3265.334	3265.514	3264.327	3264.507	326.645	1+	-0.180	-55.012	-	-	-	339 - 369	0	AVAVTSMSFPVGDVNNFVVGSEEGSVYTACR 7: Oxidation (M) 30: Carbamidomethyl (C)

cytoplasmic dynein 1 intermediate chain 2 isoform 5 [Mus musculus] gi|6753658
MW:68750.810

MSDKSDLKAELERKKQRLAQIREEKKRKEEERKKKETDQKKEAAVSVQEESDLEKKRREAEALLQSMGLTTDSPIVPPPMSPSSKSVSTPSEAGSQDSGDGAVGSRRGPIKLGMAKITQVDFPPREIVTYTKETQTPVTAQPKEDEEEEDDVATPKPPVEPEEEKTLKKDEENDSKAPPHELTEEEKQQILHSEEFLSFFDHSTRIVERAL
SEQINIFFDYSGRDLEDKEGEIQAGAKLSLNRQFFDERWSKHRVVSCLDWSSQYPELLVASYNNNEEAPHEPDGVALVWNMKYKKTTPEYVFHCQSAVMSATFAKFHPNLVVGGTYSGQIVLWDNRSNKRTPVQRTPLSAAAHTHPVYCVNVVGTQNAHNLISISTDGKICSWSLDMLSHPQDSMELVHKQSKAVAVTSMSFPVGDVNNFV
VGSEEGSVYTACRHGSKAGISEMFEGHQGPITGIHCHAAVGAVDFSHLFVTSSFDWTVKLWTTKNNKPLYSFEDNSDYVYDVMWSPTHPALFACVDGMGRLDLWNLNNDTEVPTASISVEGNPALNRVRWTHSGREIAVGDSEGQIVIYDVGEQIAVPRNDEWARFGRTLAEINANRADAEEEAATRIPA

Digest Matches (Score: 145.00)
Score = 145.000000, Rank = 1, Database = NCBInr, Accesskey = gi|6753658
Search Parameters: MS Tol.:100.00 ppm, MSMS Tol.: 0.600000 Da, Enz.Trypsin, Engine: Mascot Version: 2.3.01.241 , DB: NCBInr , DB Version: NCBInr_20110715.fasta
Modifications: Optional: Oxidation (M)

Tree hierarchy	Meas. M/z	Calc. MH+	Meas. Mr	Calc. Mr	Int.	z	Dev.(Da)	Dev.(ppm)	Score	MascotScore	Rt(min)	Range	P	Sequence
peak 4	841.349	841.384	840.342	840.377	763.525	1+	-0.035	-41.318	-	-	-	244 - 249	0	QFFDER
peak 9	1072.545	1072.579	1071.538	1071.571	2939.736	1+	-0.033	-30.974	-	-	-	117 - 125	0	ITQVDFPPR
MSMS 14	1759.813	1759.865	1758.805	1758.858	13234.713	1+	-0.052	-29.676	20	-	-	210 - 224	0	ALSEQINIFFDYSGR
peak 18	1906.956	1907.027	1905.949	1906.020	412.740	1+	-0.071	-37.358	-	-	-	117 - 132	1	ITQVDFPPREIVTYTK
peak 24	2220.996	2221.067	2219.989	2220.060	651.559	1+	-0.071	-32.090	-	-	-	188 - 205	0	QQILHSEEFLSFFDHSTR
peak 26	2290.968	2291.047	2289.961	2290.040	394.153	1+	-0.079	-34.413	-	-	-	297 - 316	0	TTPEYVFHCQSAVMSATFAK 14: Oxidation (M) 9: Carbamidomethyl (C)
peak 29	2372.114	2372.215	2371.106	2371.207	280.231	1+	-0.101	-42.574	-	-	-	317 - 337	0	FHPNLVVGGTYSGQIVLWDNR
MSMS 35	2557.215	2557.314	2556.208	2556.307	7714.664	1+	-0.100	-38.975	1043	80	-	558 - 581	0	EIAVGDSEGQIVIYDVGEQIAVPR
peak 43	3265.334	3265.514	3264.327	3264.507	326.645	1+	-0.180	-55.012	-	-	-	405 - 435	0	AVAVTSMSFPVGDVNNFVVGSEEGSVYTACR 7: Oxidation (M) 30: Carbamidomethyl (C)

Copyright © 1999-2008 Bruker Daltonik GmbH
