# Supplementary material for: Proteomic Analysis of the Action of the Mycobacterium ulcerans Toxin Mycolactone: Targeting Host Cells Cytoskeleton and Collagen
Source: PLoS Negl Trop Dis. 2014 Aug 7;8(8):e3066. doi: 10.1371/journal.pntd.0003066 (PMC4125307; doi:10.1371/journal.pntd.0003066)
Supplement: Dataset S7 — MS and MS/MS data. (ZIP) [file pntd.0003066.s010.zip › MS Data/Spot 02 - Hspa1b.pdf]

D:\Data\Bernardo\2011_07_27\M23_28\0_J18\1\1SRef

Comment 1

Comment 2

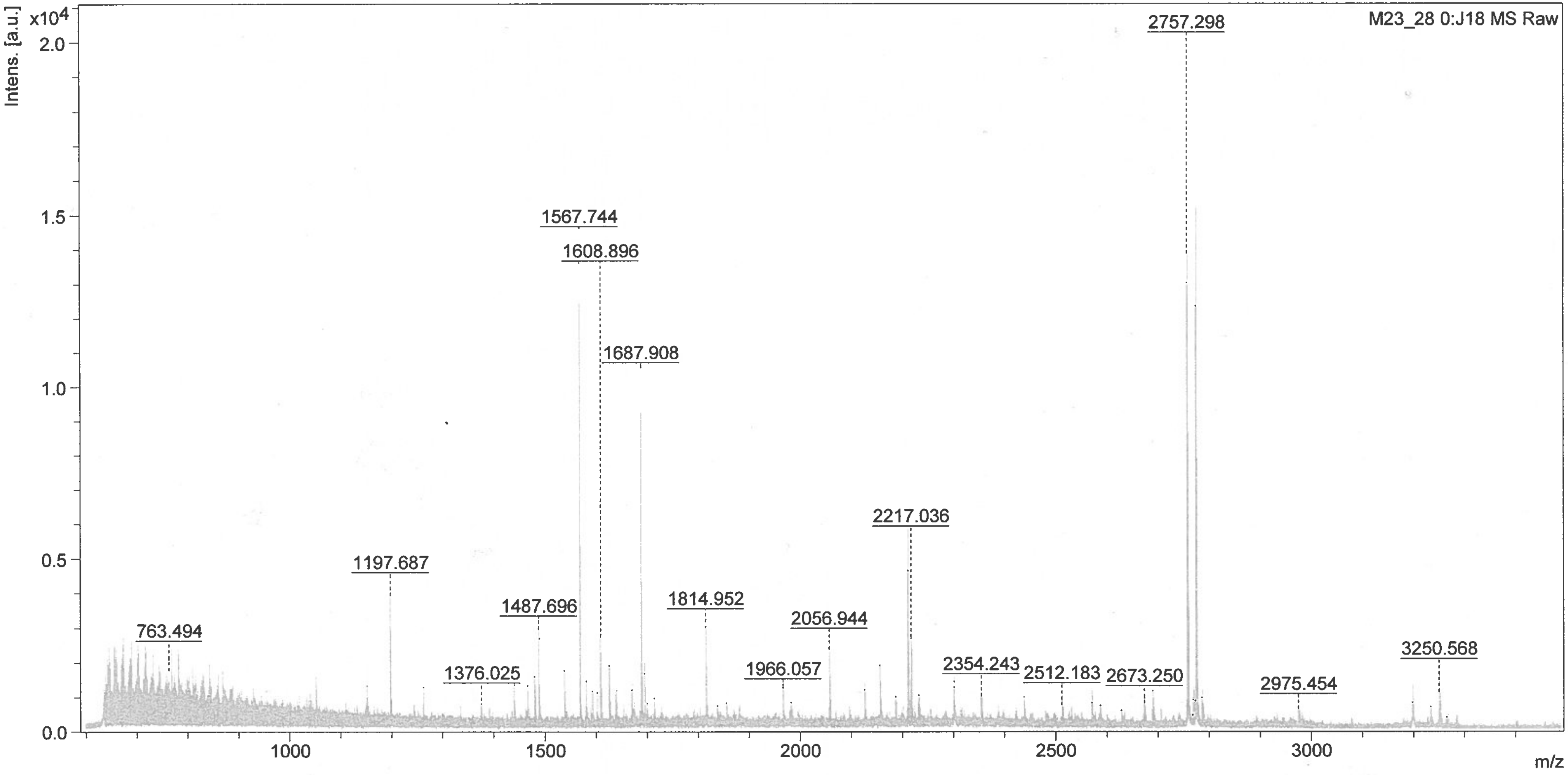

Spectrum Analysis Report

Date: 07/29/2011 Time: 06:56

FileName: D:\Data\Bernardo\2011_07_27\M23_28\0_J18\1\1SRef\pdata\1\PMF_LIFT.xml

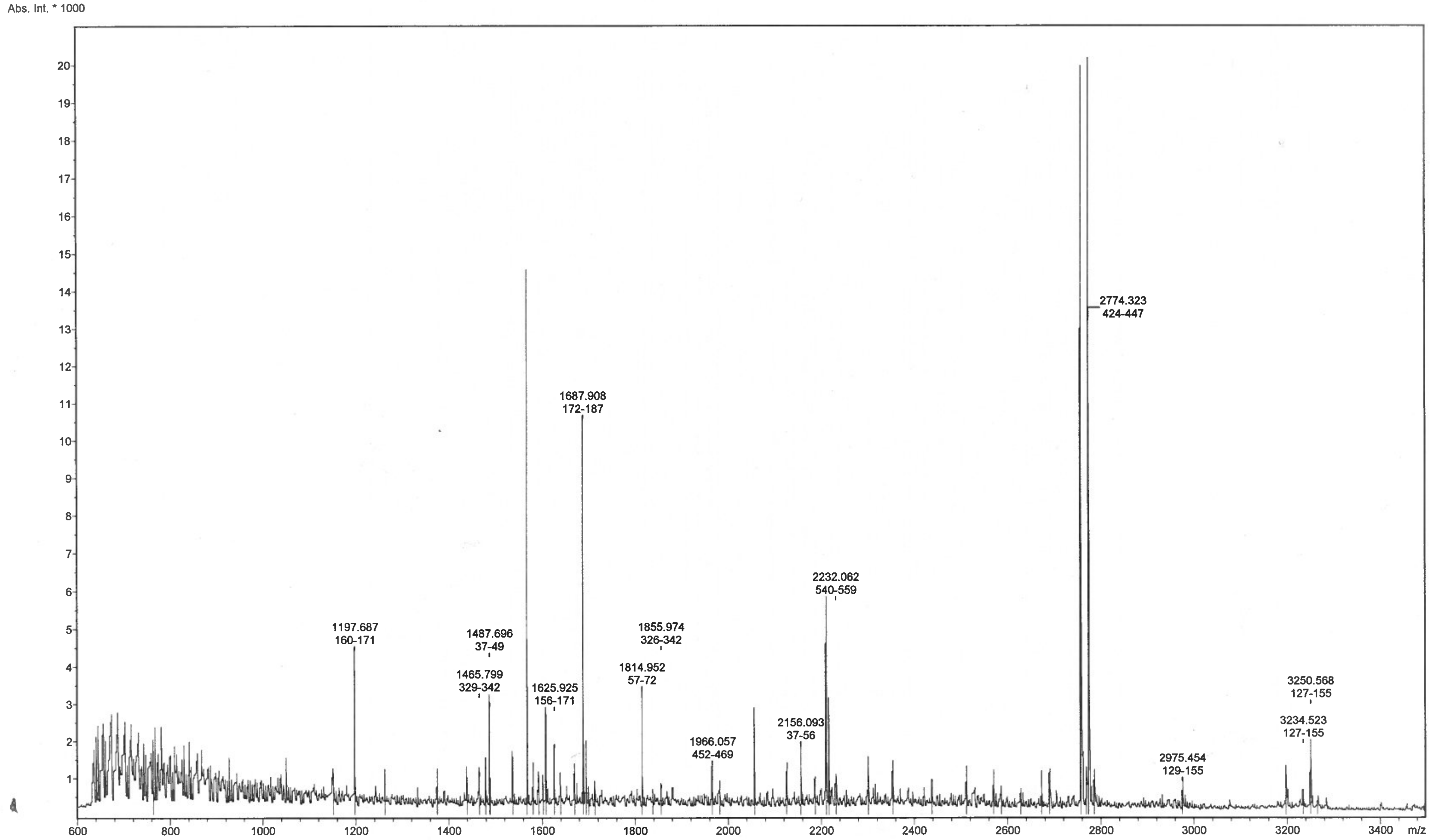

Copyright © 1999-2008 Bruker Daltonik GmbH

Spectrum Analysis Report

Date: 07/29/2011 Time: 06:56

FileName: D:\Data\Bernardo\2011_07_27\M23_28\0_J18\1\1SRef\pdata\1\PMF_LIFT.xml

Sequence data:

Heat shock 70 kDa protein 1A OS=Mus musculus GN=Hspa1a PE=1 SV=2 HS71A_MOUSE

Intensity Coverage:	26.7 % (31475 cnts)	Sequence Coverage MS:	27.5%
Sequence Coverage MS/MS:	6.2%	pI (isoelectric point):	5.4

10	20	30	40	50	60	70	80	90	100	110
MAKNTAIGID	LGTTYSCVGV	FQHGKVEIIA	NDQGNRTTPS	YVAFTDTERL	IGDAAKNQVA	LNPQNTVFDA	KRLIGRKFGD	AVVQSDMKHW	PFQVVNDGDK	PKVQVNYKGE
120	130	140	150	160	170	180	190	200	210	220
SRSFFPEEIS	SMVLTKMKEI	AEAYLGHPVT	NAVITVPAYF	NDSQRQATKD	AGVIAGLNVL	RIINEPTAAA	IAYGLDRTGK	GERNVLIFDL	GGGTFDVSIL	TIDDGIFEVK
230	240	250	260	270	280	290	300	310	320	330
ATAGDTHLGG	EDFDNRLVSH	FVEEFKRKHK	KDISQNKRAV	RRLRTACERA	KRTLSSSTQA	SLEIDSLFEG	IDFYTSITRA	RFEELCSDLF	RGTLEPVEKA	LRDAKMDKAQ
340	350	360	370	380	390	400	410	420	430	440
IHDLVLVGGS	TRIPKVQKLL	QDFFNGRDLN	KSINPDEAVA	YGAAVQAAIL	MGDKSENVQD	LLLLDVAPLS	LGLETAGGVM	TALIKRNSTI	PTKQTQTFTT	YSDNQPGVLI
450	460	470	480	490	500	510	520	530	540	550
QVYEGERAMT	RDNNLLGRFE	LSGIPPAPRG	VPQIEVTFDI	DANGILNVTA	TDKSTGKANK	ITITNDKGRL	SKEEIERMVQ	EAERYKAEDE	VQRDRVAAKN	ALESYAFNMK
560	570	580	590	600	610	620	630	640	650	
SAVEDEGLKG	KLSEADKKKV	LDKCQEVISW	LDSNTLADKE	EFVHKREELE	RVCSPIISGL	YQGAGAPGAG	GFGAQAPKGA	SGSGPTIEEV	D	

Acquisition Parameter:

Matched Sequences:

Unmatched

Peaks/MSMS Spectra

Tree hierarchy	Meas. M/z	Calc. MH+	Meas. Mr	Calc. Mr	Int.	z	Dev.(Da)	Dev.(ppm)	Score	MascotScore	Rt(min)	Range	P	Sequence
peak 1	763.494	-	762.487	-	1743.574	1+	-	-	-	-	-		-	
peak 2	1151.751	-	1150.744	-	1144.118	1+	-	-	-	-	-		-	
peak 4	1262.933	-	1261.925	-	1085.210	1+	-	-	-	-	-		-	
peak 5	1375.038	-	1374.031	-	629.760	1+	-	-	-	-	-		-	
peak 6	1376.025	-	1375.018	-	877.642	1+	-	-	-	-	-		-	
peak 7	1439.805	-	1438.798	-	1023.699	1+	-	-	-	-	-		-	
peak 9	1479.791	-	1478.784	-	1551.588	1+	-	-	-	-	-		-	
peak 11	1489.109	-	1488.101	-	1013.426	1+	-	-	-	-	-		-	
peak 12	1537.798	-	1536.791	-	1582.007	1+	-	-	-	-	-		-	
peak 13	1567.744	-	1566.737	-	14582.872	1+	-	-	-	-	-		-	
peak 14	1580.805	-	1579.797	-	1206.651	1+	-	-	-	-	-		-	
peak 15	1592.938	-	1591.931	-	1066.031	1+	-	-	-	-	-		-	
peak 16	1602.193	-	1601.186	-	837.830	1+	-	-	-	-	-		-	
peak 17	1608.896	-	1607.889	-	2722.383	1+	-	-	-	-	-		-	
peak 19	1639.881	-	1638.874	-	765.532	1+	-	-	-	-	-		-	
peak 20	1669.888	-	1668.881	-	1267.133	1+	-	-	-	-	-		-	
peak 22	1694.845	-	1693.838	-	1869.411	1+	-	-	-	-	-		-	
peak 23	1699.889	-	1698.882	-	613.055	1+	-	-	-	-	-		-	
peak 24	1713.802	-	1712.795	-	694.456	1+	-	-	-	-	-		-	
peak 26	1837.997	-	1836.989	-	637.686	1+	-	-	-	-	-		-	
peak 29	1982.017	-	1981.010	-	738.803	1+	-	-	-	-	-		-	
peak 30	2056.944	-	2055.937	-	2366.470	1+	-	-	-	-	-		-	
peak 31	2126.018	-	2125.010	-	1108.173	1+	-	-	-	-	-		-	
peak 33	2187.022	-	2186.014	-	772.835	1+	-	-	-	-	-		-	
peak 34	2211.116	-	2210.109	-	4799.838	1+	-	-	-	-	-		-	
peak 35	2217.036	-	2216.029	-	2718.134	1+	-	-	-	-	-		-	
peak 37	2301.093	-	2300.085	-	870.579	1+	-	-	-	-	-		-	
peak 38	2301.589	-	2300.582	-	643.832	1+	-	-	-	-	-		-	
peak 39	2354.243	-	2353.236	-	1000.830	1+	-	-	-	-	-		-	
peak 40	2438.189	-	2437.182	-	684.918	1+	-	-	-	-	-		-	
peak 41	2512.183	-	2511.176	-	733.013	1+	-	-	-	-	-		-	
peak 42	2570.189	-	2569.182	-	788.999	1+	-	-	-	-	-		-	

Copyright © 1999-2008 Bruker Daltonik GmbH

peak 43	2587.205	-	2586.198	-	536.306	1+	-	-	-	-	-	-	
peak 45	2673.250	-	2672.242	-	797.804	1+	-	-	-	-	-	-	
peak 46	2690.258	-	2689.251	-	841.712	1+	-	-	-	-	-	-	
peak 47	2757.298	-	2756.290	-	13861.741	1+	-	-	-	-	-	-	
peak 48	2768.288	-	2767.281	-	662.070	1+	-	-	-	-	-	-	
peak 49	2773.203	-	2772.196	-	1371.327	1+	-	-	-	-	-	-	
peak 51	2786.306	-	2785.299	-	819.739	1+	-	-	-	-	-	-	
peak 53	3197.636	-	3196.628	-	643.011	1+	-	-	-	-	-	-	
peak 56	3265.541	-	3264.534	-	326.147	1+	-	-	-	-	-	-	

Global peptide results

Heat shock 70 kDa protein 1B OS=Mus musculus GN=Hspa1b PE=1 SV=3 HS71B_MOUSE
MW:70418.110

MAKNTAIGIDLGTTYSCVGVFQHGKVEIIANDQGNRTTPSYVAFTDTERLIGDAAKNQVALNPQNTVFDAKRLIGRKFGDAVVQSDMKHWPFQVVNDGDKPKVQVNYKGESRSFFPEEISSMVLTKMKEIAEAYLGHPVTNAVITVPAYFNDSQRQATKDAGVIAGLNVLRIINEPTAAAIAYGLDRTGKGERNVLIFDLGGGTFDVSILT
IDDGIFEVKATAGDTHLGGEDFDNRLVSHFVEEFKRKHKKDISQNKRAVRRLRTACERAKRTLSSSTQASLEIDSLFEGIDFYTSITRARFEELCSDLFRGTLEPVEKALRDAKMDKAQIHDLVLVGGSTRIPKVQKLLQDFFNGRDLNKSINPDEAVAYGAAVQAAILMGDKSENVQDLLLLDVAPLSLGLETAGGVMTALIKRNSTIPT
KQTQTFTTYSDNQPGVLIQVYEGERAMTRDNNLLGRFELSGIPPAPRGVPQIEVTFDIDANGILNVTATDKSTGKANKITITNDKGRLSKEEIERMVQEAERYKAEDEVQRDRVAAKNALESYAFNMKSAVEDEGLKGKLSEADKKKVLDKCQEVISWLDSNTLADKEEFVHKREELERVCSPIISGLYQGAGAPGAGGFGAQAPPKGASG
SGPTIEEVD

Digest Matches (Score: 184.00)
Score = 184.000000, Rank = 1, Database = SwissProt, Accesskey = HS71B_MOUSE
Search Parameters: MS Tol.:100.00 ppm, MSMS Tol.: 0.600000 Da, Enz.Trypsin, Engine: Mascot Version: 2.3.01.241 , DB: NCBInr NCBInr , DB Version: NCBInr_20110715.fasta NCBInr_20110715.fasta
Modifications: Optional: Oxidation (M)

Tree hierarchy	Meas. M/z	Calc. MH+	Meas. Mr	Calc. Mr	Int.	z	Dev.(Da)	Dev.(ppm)	Score	MascotScore	Rt(min)	Range	P	Sequence
peak 3	1197.687	1197.695	1196.680	1196.688	3907.883	1+	-0.008	-6.317	-	-	-	160 - 171	0	DAGVIAGLNVLR
peak 8	1465.799	1465.812	1464.792	1464.805	1056.510	1+	-0.013	-8.803	-	-	-	329 - 342	0	AQIHDLVLVGGSTR
peak 10	1487.696	1487.701	1486.689	1486.694	2941.208	1+	-0.005	-3.551	-	-	-	37 - 49	0	TTPSYVAFTDTER
peak 18	1625.925	1625.933	1624.918	1624.926	1819.810	1+	-0.008	-5.008	-	-	-	156 - 171	1	QATKDAGVIAGLNVLR
MSMS 21	1687.908	1687.901	1686.901	1686.894	10530.833	1+	0.007	3.889	43	69	-	172 - 187	0	IINEPTAAAIAYGLDR
peak 25	1814.952	1814.951	1813.945	1813.943	3157.968	1+	0.002	0.864	-	-	-	57 - 72	1	NQVALNPQNTVFDAKR
peak 27	1855.974	1855.969	1854.967	1854.962	727.909	1+	0.004	2.422	-	-	-	326 - 342	1	MDKAQIHDLVLVGGSTR 1: Oxidation (M)
peak 28	1966.057	1966.050	1965.050	1965.043	1234.222	1+	0.007	3.440	-	-	-	452 - 469	1	DNNLLGRFELSGIPPAPR
peak 32	2156.093	2156.087	2155.086	2155.080	1829.326	1+	0.006	2.950	-	-	-	37 - 56	1	TTPSYVAFTDTERLIGDAAK
peak 36	2232.062	2232.049	2231.055	2231.042	856.783	1+	0.013	5.805	-	-	-	540 - 559	1	NALESYAFNMKSAVEDEGLK 10: Oxidation (M)
MSMS 44 X	2628.303	2628.324	2627.296	2627.317	436.361	1+	-0.021	-7.854	1	-	-	602 - 629	0	VCSPIISGLYQGAGAPGAGGFGAQAPPK 2: Carbamidomethyl (C)
MSMS 50	2774.323	2774.327	2773.316	2773.320	13223.342	1+	-0.004	-1.300	41	11	-	424 - 447	0	QTQTFTTYSDNQPGVLIQVYEGER
peak 52	2975.454	2975.490	2974.447	2974.482	629.392	1+	-0.036	-12.031	-	-	-	129 - 155	0	EIAEAYLGHPVTNAVITVPAYFNDSQR
peak 54	3234.523	3234.625	3233.516	3233.618	293.994	1+	-0.102	-31.567	-	-	-	127 - 155	1	MKEIAEAYLGHPVTNAVITVPAYFNDSQR
peak 55	3250.568	3250.620	3249.560	3249.613	1118.240	1+	-0.052	-16.131	-	-	-	127 - 155	1	MKEIAEAYLGHPVTNAVITVPAYFNDSQR 1: Oxidation (M)

Heat shock 70 kDa protein 1A OS=Mus musculus GN=Hspa1a PE=1 SV=2 HS71A_MOUSE
MW:70321.060

MAKNTAIGIDLGTTYSCVGVFQHGKVEIIANDQGNRTTPSYVAFTDTERLIGDAAKNQVALNPQNTVFDAKRLIGRKFGDAVVQSDMKHWPFQVVNDGDKPKVQVNYKGESRSFFPEEISSMVLTKMKEIAEAYLGHPVTNAVITVPAYFNDSQRQATKDAGVIAGLNVLRIINEPTAAAIAYGLDRTGKGERNVLIFDLGGGTFDVSILT
IDDGIFEVKATAGDTHLGGEDFDNRLVSHFVEEFKRKHKKDISQNKRAVRRLRTACERAKRTLSSSTQASLEIDSLFEGIDFYTSITRARFEELCSDLFRGTLEPVEKALRDAKMDKAQIHDLVLVGGSTRIPKVQKLLQDFFNGRDLNKSINPDEAVAYGAAVQAAILMGDKSENVQDLLLLDVAPLSLGLETAGGVMTALIKRNSTIPT
KQTQTFTTYSDNQPGVLIQVYEGERAMTRDNNLLGRFELSGIPPAPRGVPQIEVTFDIDANGILNVTATDKSTGKANKITITNDKGRLSKEEIERMVQEAERYKAEDEVQRDRVAAKNALESYAFNMKSAVEDEGLKGKLSEADKKKVLDKCQEVISWLDSNTLADKEEFVHKREELERVCSPIISGLYQGAGAPGAGGFGAQAPKGASGS
GPTIEEVD

Digest Matches (Score: 172.00)
Score = 172.000000, Rank = 1, Database = SwissProt, Accesskey = HS71A_MOUSE
Search Parameters: MS Tol.:100.00 ppm, MSMS Tol.: 0.600000 Da, Enz.Trypsin, Engine: Mascot Version: 2.3.01.241 , DB: NCBInr NCBInr , DB Version: NCBInr_20110715.fasta NCBInr_20110715.fasta
Modifications: Optional: Oxidation (M)

Tree hierarchy	Meas. M/z	Calc. MH+	Meas. Mr	Calc. Mr	Int.	z	Dev.(Da)	Dev.(ppm)	Score	MascotScore	Rt(min)	Range	P	Sequence
peak 3	1197.687	1197.695	1196.680	1196.688	3907.883	1+	-0.008	-6.317	-	-	-	160 - 171	0	DAGVIAGLNVLR
peak 8	1465.799	1465.812	1464.792	1464.805	1056.510	1+	-0.013	-8.803	-	-	-	329 - 342	0	AQIHDLVLVGGSTR
peak 10	1487.696	1487.701	1486.689	1486.694	2941.208	1+	-0.005	-3.551	-	-	-	37 - 49	0	TTPSYVAFTDTER
peak 18	1625.925	1625.933	1624.918	1624.926	1819.810	1+	-0.008	-5.008	-	-	-	156 - 171	1	QATKDAGVIAGLNVLR
MSMS 21	1687.908	1687.901	1686.901	1686.894	10530.833	1+	0.007	3.889	43	69	-	172 - 187	0	IINEPTAAAIAYGLDR
peak 25	1814.952	1814.951	1813.945	1813.943	3157.968	1+	0.002	0.864	-	-	-	57 - 72	1	NQVALNPQNTVFDAKR
peak 27	1855.974	1855.969	1854.967	1854.962	727.909	1+	0.004	2.422	-	-	-	326 - 342	1	MDKAQIHDLVLVGGSTR 1: Oxidation (M)
peak 28	1966.057	1966.050	1965.050	1965.043	1234.222	1+	0.007	3.440	-	-	-	452 - 469	1	DNNLLGRFELSGIPPAPR
peak 32	2156.093	2156.087	2155.086	2155.080	1829.326	1+	0.006	2.950	-	-	-	37 - 56	1	TTPSYVAFTDTERLIGDAAK
peak 36	2232.062	2232.049	2231.055	2231.042	856.783	1+	0.013	5.805	-	-	-	540 - 559	1	NALESYAFNMKSAVEDEGLK 10: Oxidation (M)
MSMS 50	2774.323	2774.327	2773.316	2773.320	13223.342	1+	-0.004	-1.300	41	11	-	424 - 447	0	QTQTFTTYSDNQPGVLIQVYEGER
peak 52	2975.454	2975.490	2974.447	2974.482	629.392	1+	-0.036	-12.031	-	-	-	129 - 155	0	EIAEAYLGHPVTNAVITVPAYFNDSQR
peak 54	3234.523	3234.625	3233.516	3233.618	293.994	1+	-0.102	-31.567	-	-	-	127 - 155	1	MKEIAEAYLGHPVTNAVITVPAYFNDSQR
peak 55	3250.568	3250.620	3249.560	3249.613	1118.240	1+	-0.052	-16.131	-	-	-	127 - 155	1	MKEIAEAYLGHPVTNAVITVPAYFNDSQR 1: Oxidation (M)

inducible heat shock protein 70 [Mus musculus] gi|118490060
MW:70373.100

MAKNTAIGIDLGTTYSCVGVFQHGKVEIIANDQGNRTTPSYVAFTDTERLIGDAAKNQVALNPQNTVFDAKRLIGRKFGDAVVQSDMKHWPFQVVNDGDKPKVQVNYKGESRSFFPEEISSMVLTKIKEIAEAYLGHPVTNAVITVPAYFNDSQRQATKDAGVIAGLNVLRIINEPTAAAIAYGLDRTGKGERNVLIFDLGGGTFDVSILT
IDDGIFEVKATAGDTHLGGEDFDNRLVSHFVEEFKRKHKKDISQNKRAVRRLRTACERAKRTLSSSTQASLEIDSLFEAIDFYTSITRARFEELCSDLFRGTLEPVEKALRDAKMDKAQIHDLVLVGGSTRIPKVQKLLQDFFNGRDLNKSINPDEAVAYGAAVQAAILMRDKSENVQDLLLLDVAPLSLGPEAAGGVMTALIKRNSTIPT
KQTQTFTTYSDNQPGVLIQVYEGERAMTRDNNLLGRFELSGIPPAPRGVPQIEVTFDIDANGILNVTATDKSTGKANKITITNDKGRLSKEEIERMVQEAERYKAEDEVQRDRVAAKNALESYAFNMKSAVEDEGLKGKLSEADKKKVLDKCQEVISWLDSNTLADKEEFVHKREELERVCSPIISGLYQGAGAPGAGGFGAQAPMGASGS
GPTIEEVD

Copyright © 1999-2008 Bruker Daltonik GmbH

Spectrum Analysis Report

Date: 07/29/2011 Time: 06:56

FileName: D:\Data\Bernardo\2011_07_27\M23_28\0_J18\1\1SRef\pdata\1\PMF_LIFT.xml

Digest Matches (Score: 160.00)
Score = 160.000000, Rank = 1, Database = NCBInr, Accesskey = gi|118490060
Search Parameters: MS Tol.:100.00 ppm, MSMS Tol.: 0.600000 Da, Enz.Trypsin, Engine: Mascot Version: 2.3.01.241 , DB: NCBInr NCBInr , DB Version: NCBInr_20110715.fasta NCBInr_20110715.fasta
Modifications: Optional: Oxidation (M)

Tree hierarchy	Meas. M/z	Calc. MH+	Meas. Mr	Calc. Mr	Int.	z	Dev.(Da)	Dev.(ppm)	Score	MascotScore	Rt(min)	Range	P	Sequence
peak 3	1197.687	1197.695	1196.680	1196.688	3907.883	1+	-0.008	-6.317	-	-	-	160 - 171	0	DAGVIAGLNVLR
peak 8	1465.799	1465.812	1464.792	1464.805	1056.510	1+	-0.013	-8.803	-	-	-	329 - 342	0	AQIHDLVLVGGSTR
peak 10	1487.696	1487.701	1486.689	1486.694	2941.208	1+	-0.005	-3.551	-	-	-	37 - 49	0	TTPSYVAFTDTER
peak 18	1625.925	1625.933	1624.918	1624.926	1819.810	1+	-0.008	-5.008	-	-	-	156 - 171	1	QATKDAGVIAGLNVLR
MSMS 21	1687.908	1687.901	1686.901	1686.894	10530.833	1+	0.007	3.889	43	69	-	172 - 187	0	IINEPTAAAIAYGLDR
peak 25	1814.952	1814.951	1813.945	1813.943	3157.968	1+	0.002	0.864	-	-	-	57 - 72	1	NQVALNPQNTVFDAKR
peak 27	1855.974	1855.969	1854.967	1854.962	727.909	1+	0.004	2.422	-	-	-	326 - 342	1	MDKAQIHDLVLVGGSTR 1: Oxidation (M)
peak 28	1966.057	1966.050	1965.050	1965.043	1234.222	1+	0.007	3.440	-	-	-	452 - 469	1	DNNLLGRFELSGIPPAPR
peak 32	2156.093	2156.087	2155.086	2155.080	1829.326	1+	0.006	2.950	-	-	-	37 - 56	1	TTPSYVAFTDTERLIGDAAK
peak 36	2232.062	2232.049	2231.055	2231.042	856.783	1+	0.013	5.805	-	-	-	540 - 559	1	NALESYAFNMKSAVEDEGLK 10: Oxidation (M)
MSMS 50	2774.323	2774.327	2773.316	2773.320	13223.342	1+	-0.004	-1.300	41	11	-	424 - 447	0	QTQTFTTYSDNQPGVLIQVYEGER
peak 52	2975.454	2975.490	2974.447	2974.482	629.392	1+	-0.036	-12.031	-	-	-	129 - 155	0	EIAEAYLGHPVTNAVITVPAYFNDSQR

Copyright © 1999-2008 Bruker Daltonik GmbH
