# Supplementary material for: Proteomic Analysis of the Action of the Mycobacterium ulcerans Toxin Mycolactone: Targeting Host Cells Cytoskeleton and Collagen
Source: PLoS Negl Trop Dis. 2014 Aug 7;8(8):e3066. doi: 10.1371/journal.pntd.0003066 (PMC4125307; doi:10.1371/journal.pntd.0003066)
Supplement: Dataset S7 — MS and MS/MS data. (ZIP) [file pntd.0003066.s010.zip › MS Data/Spot 03 - Plod1.pdf]

D:\Data\Bernardo\2011_07_30\P5_30\0_O9\1\1SRef

Comment 1
Comment 2

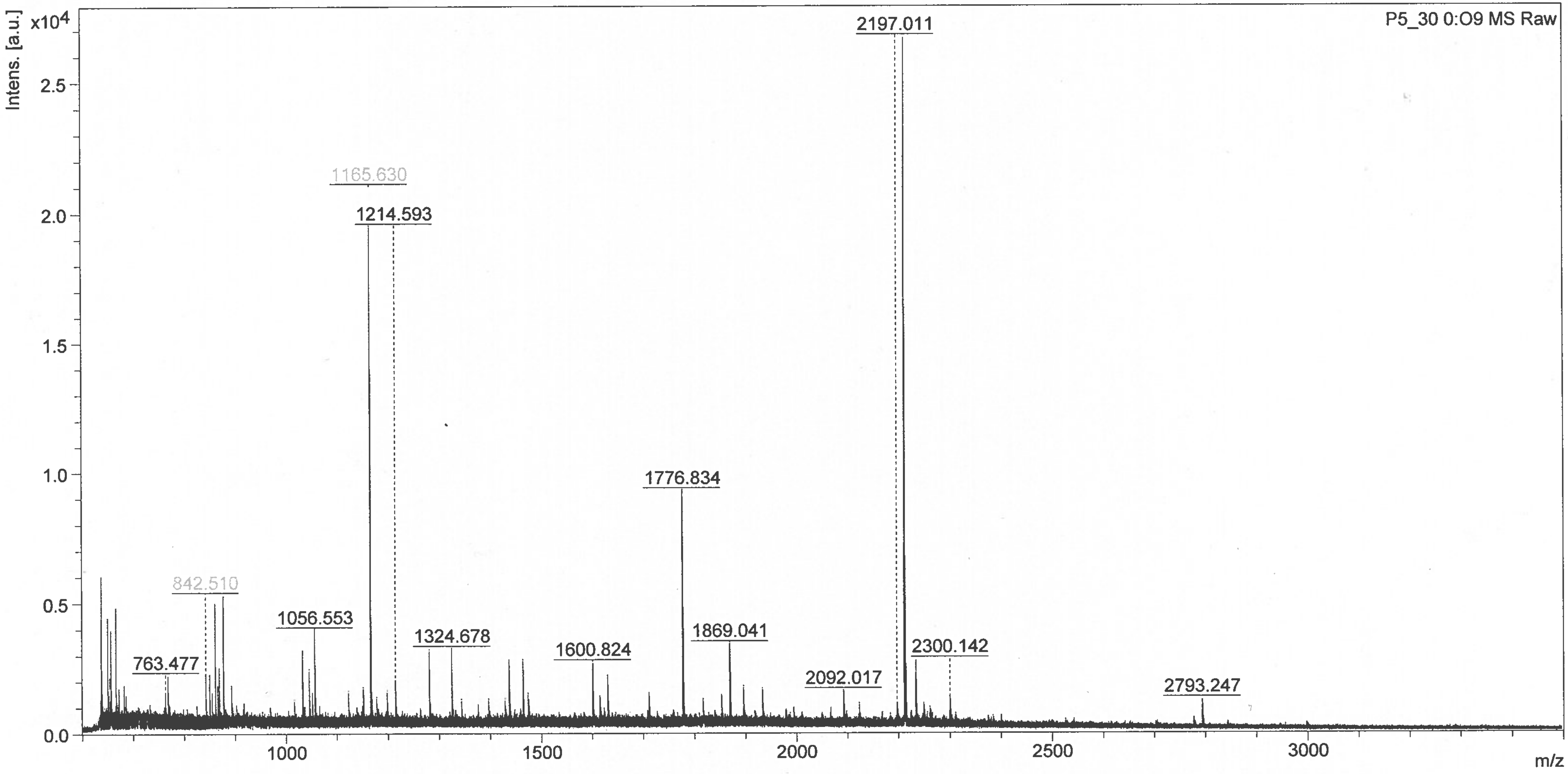

Bruker Daltonics flexAnalysis printed: 7/30/2011 12:08:17 PM

Spectrum Analysis Report

Date: 07/30/2011 Time: 12:07

FileName: D:\Data\Bernardo\2011_07_30\P5_30\0_O9\1\1SRef\pdata\1\PMF_LIFT.xml

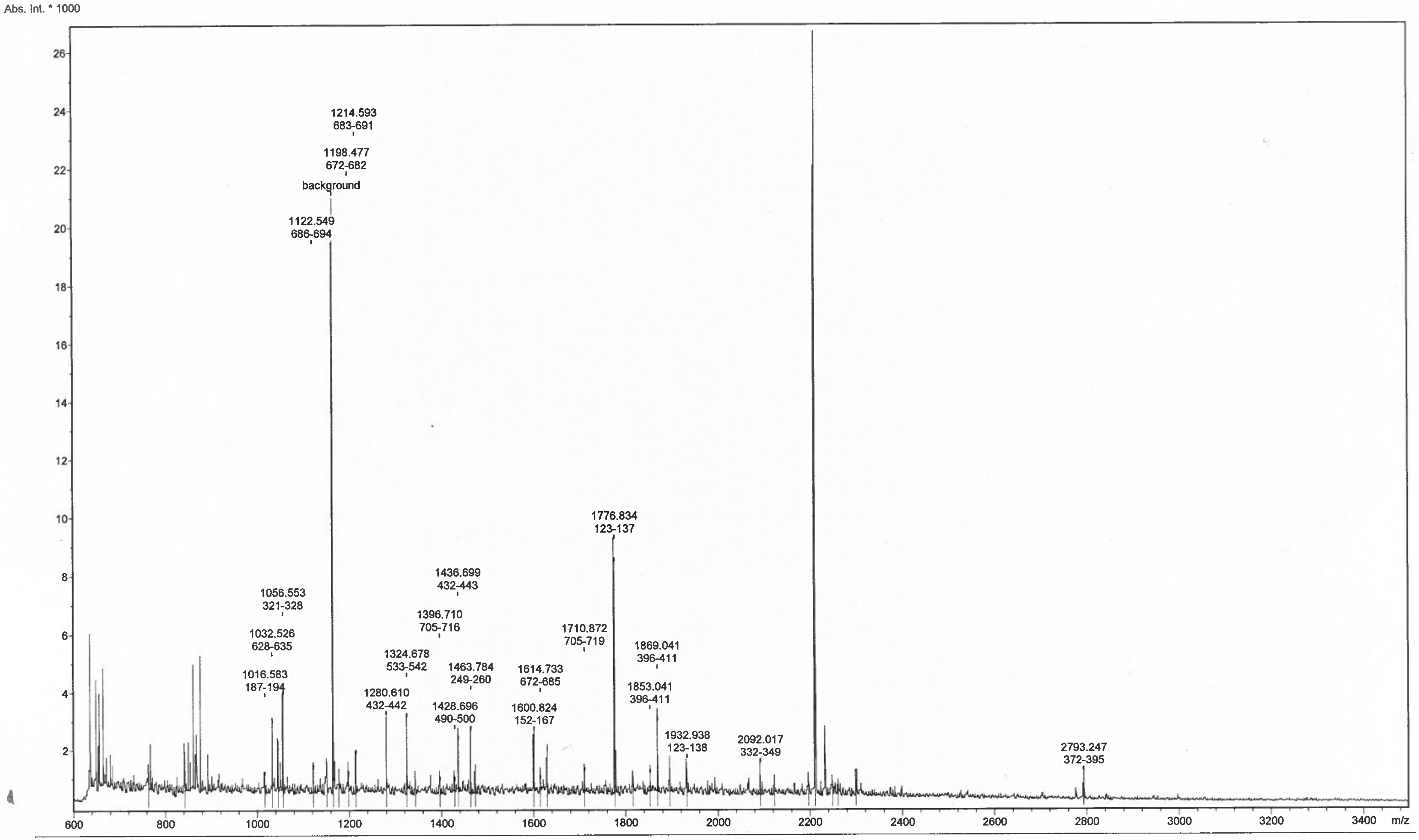

Copyright © 1999-2008 Bruker Daltonik GmbH

Spectrum Analysis Report

Date: 07/30/2011 Time: 12:07

FileName: D:\Data\Bernardo\2011_07_30\P5_30\0_O9\1\1SRef\pdata\1\PMF_LIFT.xml

Sequence data:

Procollagen-lysine,2-oxoglutarate 5-dioxygenase 1 OS=Mus musculus GN=Plod1 PE=1 SV=1 PLOD1_MOUSE

Intensity Coverage:	63.9 % (66804 cnts)	Sequence Coverage MS:	28.4%
Sequence Coverage MS/MS:	8.1%	pI (isoelectric point):	6.1

10	20	30	40	50	60	70	80	90	100	110
MRSLLLLAPL	AWLLLVQAKD	DAKLEDNLLV	LTVATKETEG	FRRFKRSAQF	FNYKIQSLGL	GEDWSVDGGP	AAAGGGQKVR	LLKKALEKHA	DKEDLVILFV	DSYDVVFASG
120	130	140	150	160	170	180	190	200	210	220
PRELLKKFQQ	AKSQVVFSAE	EHIYPDRRLE	AKYPTVPDGK	RFLGSGGFIG	YAPSLSKLVA	EWEGQDSDSD	QLFYTKIFLN	PEKREQINIS	LDHRCRIFQN	LDGALDEVVL
230	240	250	260	270	280	290	300	310	320	330
KFEMGHVRAR	NLAYDTLPVV	VHGNGPTKLQ	LNYLGNYIPR	FWTFETGCTV	CDEGLRSLKG	IGDEALPTVL	VGVFIEQPTP	FLSLFFLRLL	RLRYPQKQMR	LFIHNQERHH
340	350	360	370	380	390	400	410	420	430	440
KLQVEQFLAE	HGSEYQSVKL	VGPEVRMANA	DARNMGADLC	RQDQTCTYYF	SVDADVALTE	PNSLRLLIEQ	NKNVIAPLMT	RHGRLWSNFW	GGLSADGYYA	RSEDYVDIVQ
450	460	470	480	490	500	510	520	530	540	550
GRRVGVWNVP	YISNIYLIKG	SALRAELQNV	DLFHYSKLDS	DMSFCANVRQ	QEVFMFLTNR	HTFGHLLSLD	NYQTTHLHND	LWEVFSNPED	WKEKYIHENY	TKALAGKLVE
560	570	580	590	600	610	620	630	640	650	660
TPCPDVYWFP	IFTEAACDEL	VEEMEHYGQW	SLGDNKDNRI	QGGYENVPTI	DIHMNQITFE	REWHKFLVEY	IAPMTEKLYP	GYYTRAQFDL	AFVVRYKPDE	QPSLMPHHDA
670	680	690	700	710	720	730				
STFTVNIALN	RVGEDYEGGG	CRFLRYNCSV	RAPRKGWALL	HPGRLTHYHE	GLPTTKGTRY	IAVSFVDP				

Acquisition Parameter:

Matched Sequences:

Unmatched

Peaks/MSMS Spectra

Tree hierarchy	Meas. M/z	Calc. MH+	Meas. Mr	Calc. Mr	Int.	z	Dev.(Da)	Dev.(ppm)	Score	MascotScore	Rt(min)	Range	P	Sequence
peak 1	763.477	-	762.470	-	1233.011	1+	-	-	-	-	-		-	
peak 2	842.510	-	841.503	-	1950.483	1+	-	-	-	-	-		-	
peak 5	1045.562	-	1044.554	-	2346.540	1+	-	-	-	-	-		-	
peak 8	1151.729	-	1150.721	-	1560.833	1+	-	-	-	-	-		-	
peak 10	1177.623	-	1176.616	-	1258.217	1+	-	-	-	-	-		-	
peak 15	1343.754	-	1342.747	-	976.406	1+	-	-	-	-	-		-	
peak 20	1473.794	-	1472.787	-	1305.032	1+	-	-	-	-	-		-	
peak 23	1629.885	-	1628.878	-	1946.946	1+	-	-	-	-	-		-	
peak 26	1816.586	-	1815.578	-	835.911	1+	-	-	-	-	-		-	
peak 29	1895.967	-	1894.959	-	1321.295	1+	-	-	-	-	-		-	
peak 32	2123.098	-	2122.091	-	818.254	1+	-	-	-	-	-		-	
peak 33	2197.011	-	2196.004	-	797.209	1+	-	-	-	-	-		-	
peak 34	2211.086	-	2210.079	-	19159.029	1+	-	-	-	-	-		-	
peak 35	2249.041	-	2248.034	-	671.469	1+	-	-	-	-	-		-	
peak 36	2261.011	-	2260.004	-	656.150	1+	-	-	-	-	-		-	
peak 37	2300.142	-	2299.135	-	889.515	1+	-	-	-	-	-		-	

Global peptide results

Procollagen-lysine,2-oxoglutarate 5-dioxygenase 1 OS=Mus musculus GN=Plod1 PE=1 SV=1 PLOD1_MOUSE
MW:84112.500

MRSLLLLAPLAWLLLVQAKDDAKLEDNLLVLTVATKETEGFRRFKRSAQFFNYKIQSLGLGEDWSVDGGPAAAGGGQKVRLLKKALEKHADKEDLVILFVDSYDVVFASGPRELLKKFQQAKSQVVFSAEEHIYPDRRLEAKYPTVPDGKRFLGSGGFIGYAPSLSKLVAEWEGQDSDSDQLFYTKIFLNPEKREQINISLDHRCRIFQNL
DGALDEVVLKFEMGHVRARNLAYDTLPVVVHGNGPTKLQLNYLGNYIPRFWTFETGCTVCDEGLRSLKGIGDEALPTVLVGVFIEQPTPFLSLFFLRLLRLYPQKQMRLFIHNQERHHKLQVEQFLAEHGSEYQSVKLVGPEVRMANADARNMGADLCRQDQTCTYYFSVDADVALTEPNSLRLLIEQNKNVIAPLMTRHGRLWSNFWGG
LSADGYYARSEDYVDIVQGRRVGVWNVPYISNIYLIKGSALRAELQNVDLFHYSKLDSDMSFCANVRQQEVFMFLTNRHTFGHLLSLDNYQTTHLHNDLWEVFSNPEDWKEKYIHENYTKALAGKLVETPCPDVYWFPIFTEAACDELVEEMEHYGQWSLGDNKDNRIQGGYENVPTIDIHMNQITFEREWHKFLVEYIAPMTEKLYPGYY
TRAQFDLAFVVRYKPDEQPSLMPHHDASTFTVNIALNRVGEDYEGGGCRFLRYNCSVRAPRKGWALLHPGRLTHYHEGLPTTKGTRYIAVSFVDP

Digest Matches (Score: 256.00)
Score = 256.000000, Rank = 1, Database = SwissProt, Accesskey = PLOD1_MOUSE
Search Parameters: MS Tol.:100.00 ppm, MSMS Tol.:0.600000Da, Enz.Trypsin, Engine:Mascot Version:2.3.01.241, DB:NCBInr NCBInr, DB Version:NCBInr_20110715.fasta NCBInr_20110715.fasta
Modifications: Optional: Oxidation (M)

Copyright © 1999-2008 Bruker Daltonik GmbH

Spectrum Analysis Report

Date: 07/30/2011 Time: 12:07

FileName: D:\Data\Bernardo\2011_07_30\P5_30\0_O9\1\1SRef\pdata\1\PMF_LIFT.xml

Tree hierarchy	Meas. M/z	Calc. MH+	Meas. Mr	Calc. Mr	Int.	z	Dev.(Da)	Dev.(ppm)	Score	MascotScore	Rt(min)	Range	P	Sequence
peak 3	1016.583	1016.589	1015.576	1015.581	1135.274	1+	-0.005	-5.397	-	-	-	187 - 194	1	IFLNPEKR
peak 4	1032.526	1032.515	1031.519	1031.508	2946.047	1+	0.011	11.095	-	-	-	628 - 635	0	LYPGYYTR
MSMS 6	1056.553	1056.558	1055.546	1055.551	3933.390	1+	-0.005	-4.882	11	-	-	321 - 328	0	LFIHNQER
peak 7	1122.549	1122.547	1121.541	1121.540	1359.797	1+	0.001	1.298	-	-	-	686 - 694	1	YNCSVRAPR 3: Carbamidomethyl (C)
MSMS 9	1165.630	1165.636	1164.622	1164.629	21012.658	1+	-0.007	-5.893	94	36	-	636 - 645	0	AQFDLAFVVR
peak 11	1198.477	1198.479	1197.470	1197.472	1138.188	1+	-0.002	-2.055	-	-	-	672 - 682	0	VGEDYEGGGCR 10: Carbamidomethyl (C)
peak 12	1214.593	1214.610	1213.585	1213.603	1877.700	1+	-0.017	-14.115	-	-	-	683 - 691	1	FLRYNCSVR 6: Carbamidomethyl (C)
peak 13	1280.610	1280.612	1279.603	1279.604	2831.564	1+	-0.002	-1.421	-	-	-	432 - 442	0	SEDYVDIVQGR
MSMS 14	1324.678	1324.653	1323.670	1323.646	3152.677	1+	0.024	18.454	10	-	-	533 - 542	1	EKYIHENYTK
peak 16	1396.710	1396.722	1395.703	1395.715	933.332	1+	-0.012	-8.427	-	-	-	705 - 716	0	LTHYHEGLPTTK
peak 17	1428.696	1428.694	1427.689	1427.687	937.251	1+	0.002	1.502	-	-	-	490 - 500	0	QQEVFMFLTNR 6: Oxidation (M)
peak 18	1436.699	1436.713	1435.691	1435.706	2358.132	1+	-0.014	-9.907	-	-	-	432 - 443	1	SEDYVDIVQGRR
peak 19	1463.784	1463.800	1462.777	1462.793	2705.020	1+	-0.016	-11.262	-	-	-	249 - 260	0	LQLNYLGNYIPR
peak 21	1600.824	1600.837	1599.817	1599.830	2502.540	1+	-0.013	-7.873	-	-	-	152 - 167	0	FLGSGGFIGYAPSLSK
peak 22	1614.733	1614.733	1613.726	1613.726	992.396	1+	0.000	0.164	-	-	-	672 - 685	1	VGEDYEGGGCRFLR 10: Carbamidomethyl (C)
peak 24	1710.872	1710.892	1709.864	1709.885	1261.164	1+	-0.020	-11.953	-	-	-	705 - 719	1	LTHYHEGLPTTKGTR
MSMS 25	1776.834	1776.855	1775.827	1775.848	8472.786	1+	-0.021	-11.761	7	-	-	123 - 137	0	SQVVFSAEEHIYPDR
peak 27	1853.041	1853.068	1852.034	1852.060	1189.509	1+	-0.026	-14.217	-	-	-	396 - 411	1	LLIEQNKNVIAPLMTR
MSMS 28	1869.041	1869.063	1868.033	1868.055	3010.127	1+	-0.022	-11.786	15	-	-	396 - 411	1	LLIEQNKNVIAPLMTR 14: Oxidation (M)
peak 30	1932.938	1932.956	1931.930	1931.949	1324.848	1+	-0.019	-9.690	-	-	-	123 - 138	1	SQVVFSAEEHIYPDRR
peak 31	2092.017	2092.035	2091.009	2091.027	1069.328	1+	-0.018	-8.612	-	-	-	332 - 349	0	LQVEQFLAEHGSEYQSVK
peak 38	2793.247	2793.267	2792.240	2792.260	660.563	1+	-0.020	-7.116	-	-	-	372 - 395	0	QDQTCTYYFSVDADVALTEPNSLR 5: Carbamidomethyl (C)

Copyright © 1999-2008 Bruker Daltonik GmbH
