# Supplementary material for: Proteomic Analysis of the Action of the Mycobacterium ulcerans Toxin Mycolactone: Targeting Host Cells Cytoskeleton and Collagen
Source: PLoS Negl Trop Dis. 2014 Aug 7;8(8):e3066. doi: 10.1371/journal.pntd.0003066 (PMC4125307; doi:10.1371/journal.pntd.0003066)
Supplement: Dataset S7 — MS and MS/MS data. (ZIP) [file pntd.0003066.s010.zip › MS Data/Spot 04 - Cfl1.pdf]

D:\Data\Bernardo\2011_07_30\P23_07\0_P11\1\1SRef

Comment 1

Comment 2

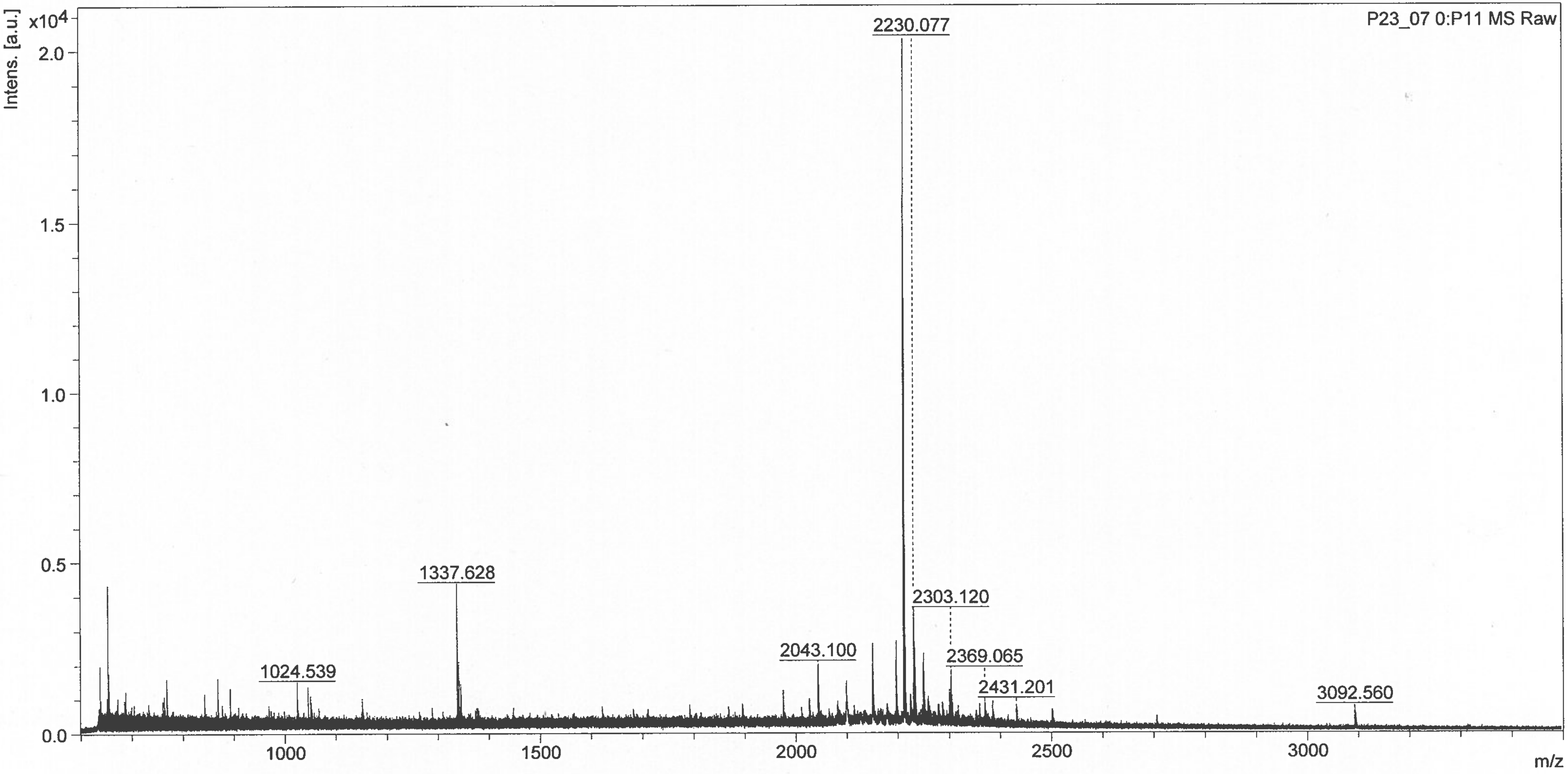

Bruker Daltonics flexAnalysis

printed: 7/30/2011 1:54:37 PM

Spectrum Analysis Report

Date: 07/30/2011 Time: 13:54

FileName: D:\Data\Bernardo\2011_07_30\P23_07\0_P11\1\1SRef\pdata\1\PMF_LIFT.xml

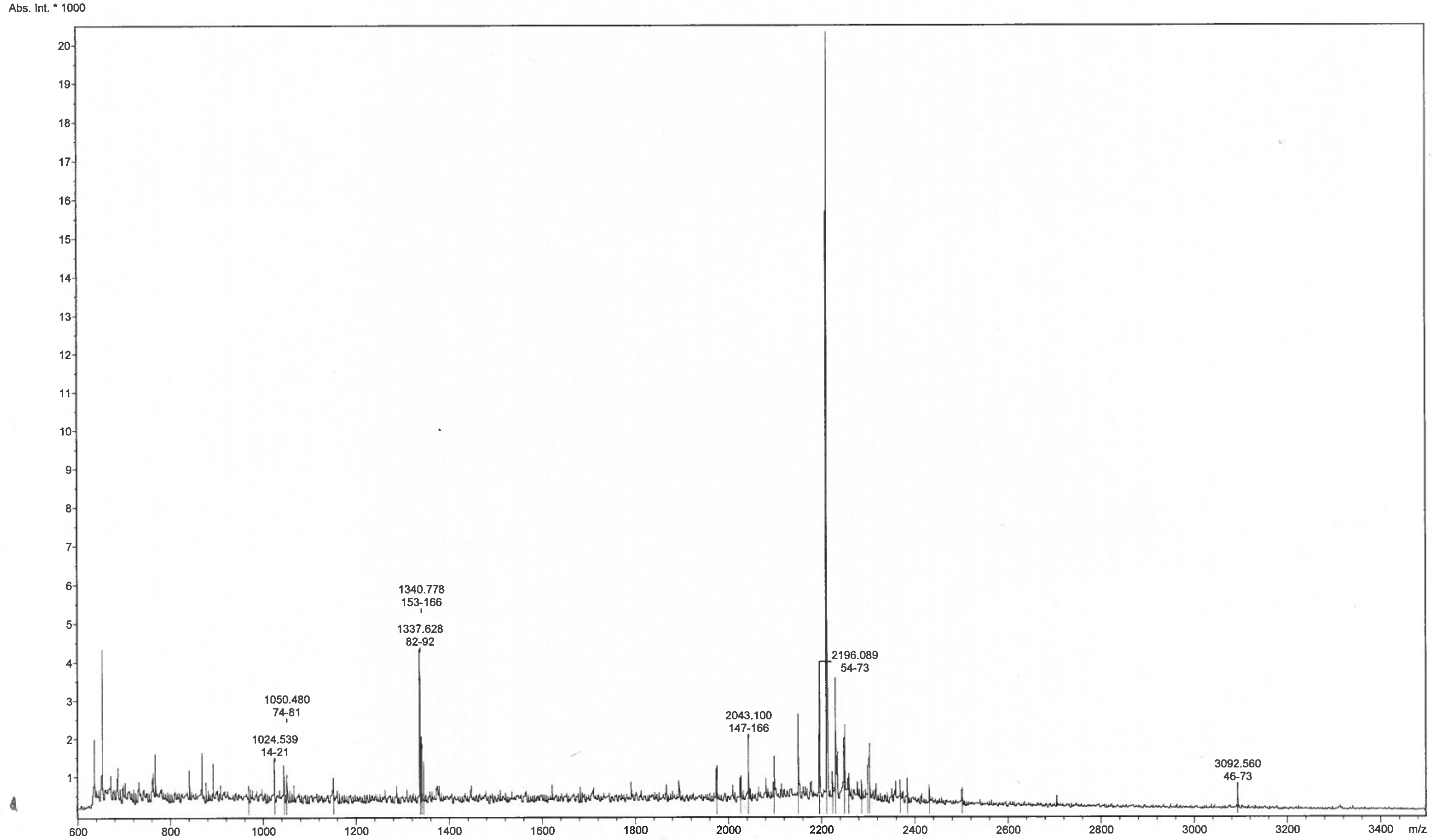

Copyright © 1999-2008 Bruker Daltonik GmbH

Spectrum Analysis Report

Date: 07/30/2011 Time: 13:54

FileName: D:\Data\Bernardo\2011_07_30\P23_07\0_P11\1\1SRef\pdata\1\PMF_LIFT.xml

Sequence data:

Cofilin-1 OS=Mus musculus GN=Cfl1 PE=1 SV=3 COF1_MOUSE

Intensity Coverage:	24.4 % (11347 cnts)	Sequence Coverage MS:	45.2%
Sequence Coverage MS/MS:	30.7%	pI (isoelectric point):	9.1

10	20	30	40	50	60	70	80	90	100	110
MASGVAVSDG	VIKVFNDMKV	RKSSTPEEVK	KRKKAVLFCL	SEDKKNIILE	EGKEILVGDV	GQTVDDPYTT	FVKMLPDKDC	RYALYDATYE	TKESKKEDLV	FIFWAPENAP
120	130	140	150	160	170					
LKSKMIYASS	KDAIKKKLTG	IKHELQANCY	EEVKDRCTLA	EKLGGSAVIS	LEGKPL					

Acquisition Parameter:

Matched Sequences:

Unmatched

Peaks/MSMS Spectra

Tree hierarchy	Meas. M/z	Calc. MH+	Meas. Mr	Calc. Mr	Int.	z	Dev.(Da)	Dev.(ppm)	Score	MascotScore	Rt(min)	Range	P	Sequence
peak 1	968.591	-	967.583	-	655.614	1+	-	-	-	-	-		-	
peak 3	1045.573	-	1044.566	-	997.672	1+	-	-	-	-	-		-	
peak 5	1151.742	-	1150.734	-	799.991	1+	-	-	-	-	-		-	
peak 8	1344.749	-	1343.742	-	1223.113	1+	-	-	-	-	-		-	
peak 9	1974.923	-	1973.916	-	938.623	1+	-	-	-	-	-		-	
peak 10	2026.076	-	2025.069	-	783.504	1+	-	-	-	-	-		-	
peak 12	2098.472	-	2097.465	-	963.871	1+	-	-	-	-	-		-	
peak 13	2149.857	-	2148.850	-	1726.018	1+	-	-	-	-	-		-	
peak 15	2211.093	-	2210.085	-	16443.814	1+	-	-	-	-	-		-	
peak 16	2224.073	-	2223.066	-	687.021	1+	-	-	-	-	-		-	
MSMS 17	2230.077	-	2229.070	-	2876.582	1+	-	-	-	-	-		-	
MSMS 18	2249.038	-	2248.031	-	1770.757	1+	-	-	-	-	-		-	
peak 19	2258.074	-	2257.066	-	648.727	1+	-	-	-	-	-		-	
peak 20	2286.067	-	2285.060	-	570.088	1+	-	-	-	-	-		-	
peak 21	2299.136	-	2298.129	-	777.946	1+	-	-	-	-	-		-	
peak 22	2303.120	-	2302.113	-	1179.763	1+	-	-	-	-	-		-	
peak 23	2369.065	-	2368.058	-	570.399	1+	-	-	-	-	-		-	
peak 24	2383.954	-	2382.946	-	517.582	1+	-	-	-	-	-		-	
peak 25	2431.201	-	2430.194	-	540.570	1+	-	-	-	-	-		-	
peak 26	2501.209	-	2500.201	-	442.116	1+	-	-	-	-	-		-	

Global peptide results

Cofilin-1 OS=Mus musculus GN=Cfl1 PE=1 SV=3 COF1_MOUSE
MW:18775.760

MASGVAVSDGVIKVFNDMKVRKSSTPEEVKKRKKAVLFCLSEDKKNIILEEGKEILVGDVGQTVDDPYTTFVKMLPDKDCRYALYDATYETKESKKEDLVFIFWAPENAPLKSKMIYASSKDAIKKKLTGIKHELQANCYEEVKDRCTLAEKLGGSAVISLEGKPL

Digest Matches (Score: 140.00)
Score = 140.000000, Rank = 1, Database = SwissProt, Accesskey = COF1_MOUSE
Search Parameters: MS Tol.:100.00 ppm, MSMS Tol.:0.600000Da, Enz.Trypsin, Engine:Mascot Version:2.3.01.241, DB:NCBInr NCBInr, DB Version:NCBInr_20110715.fasta NCBInr_20110715.fasta
Modifications: Optional: Oxidation (M)

Tree hierarchy	Meas. M/z	Calc. MH+	Meas. Mr	Calc. Mr	Int.	z	Dev.(Da)	Dev.(ppm)	Score	MascotScore	Rt(min)	Range	P	Sequence
peak 2	1024.539	1024.524	1023.532	1023.517	1182.768	1+	0.014	14.156	-	-	-	14 - 21	1	VFNDMKVR 5: Oxidation (M)
peak 4	1050.480	1050.471	1049.472	1049.463	679.965	1+	0.009	8.509	-	-	-	74 - 81	1	MLPDKDCR 1: Oxidation (M) 7: Carbamidomethyl (C)
MSMS 6	1337.628	1337.626	1336.621	1336.619	3744.843	1+	0.002	1.809	75	25	-	82 - 92	0	YALYDATYETK
peak 7	1340.778	1340.778	1339.771	1339.771	1740.382	1+	-0.000	-0.168	-	-	-	153 - 166	0	LGGSAVISLEGKPL
MSMS 11	2043.100	2043.115	2042.093	2042.108	1705.630	1+	-0.015	-7.360	3	10	-	147 - 166	1	CTLAEKLGGSAVISLEGKPL 1: Carbamidomethyl (C)
MSMS 14	2196.089	2196.107	2195.082	2195.100	1957.810	1+	-0.018	-8.163	7	14	-	54 - 73	0	EILVGDVGQTVDDPYTTFVK
peak 27	3092.560	3092.604	3091.553	3091.597	335.841	1+	-0.044	-14.195	-	-	-	46 - 73	1	NIILEEGKEILVGDVGQTVDDPYTTFVK

Copyright © 1999-2008 Bruker Daltonik GmbH
