# Supplementary material for: Proteomic Analysis of the Action of the Mycobacterium ulcerans Toxin Mycolactone: Targeting Host Cells Cytoskeleton and Collagen
Source: PLoS Negl Trop Dis. 2014 Aug 7;8(8):e3066. doi: 10.1371/journal.pntd.0003066 (PMC4125307; doi:10.1371/journal.pntd.0003066)
Supplement: Dataset S7 — MS and MS/MS data. (ZIP) [file pntd.0003066.s010.zip › MS Data/Spot 05 - Plod3.pdf]

D:\Data\Bernardo\2011_07_30\P5_31\0_P9\1\1SRef

Comment 1

Comment 2

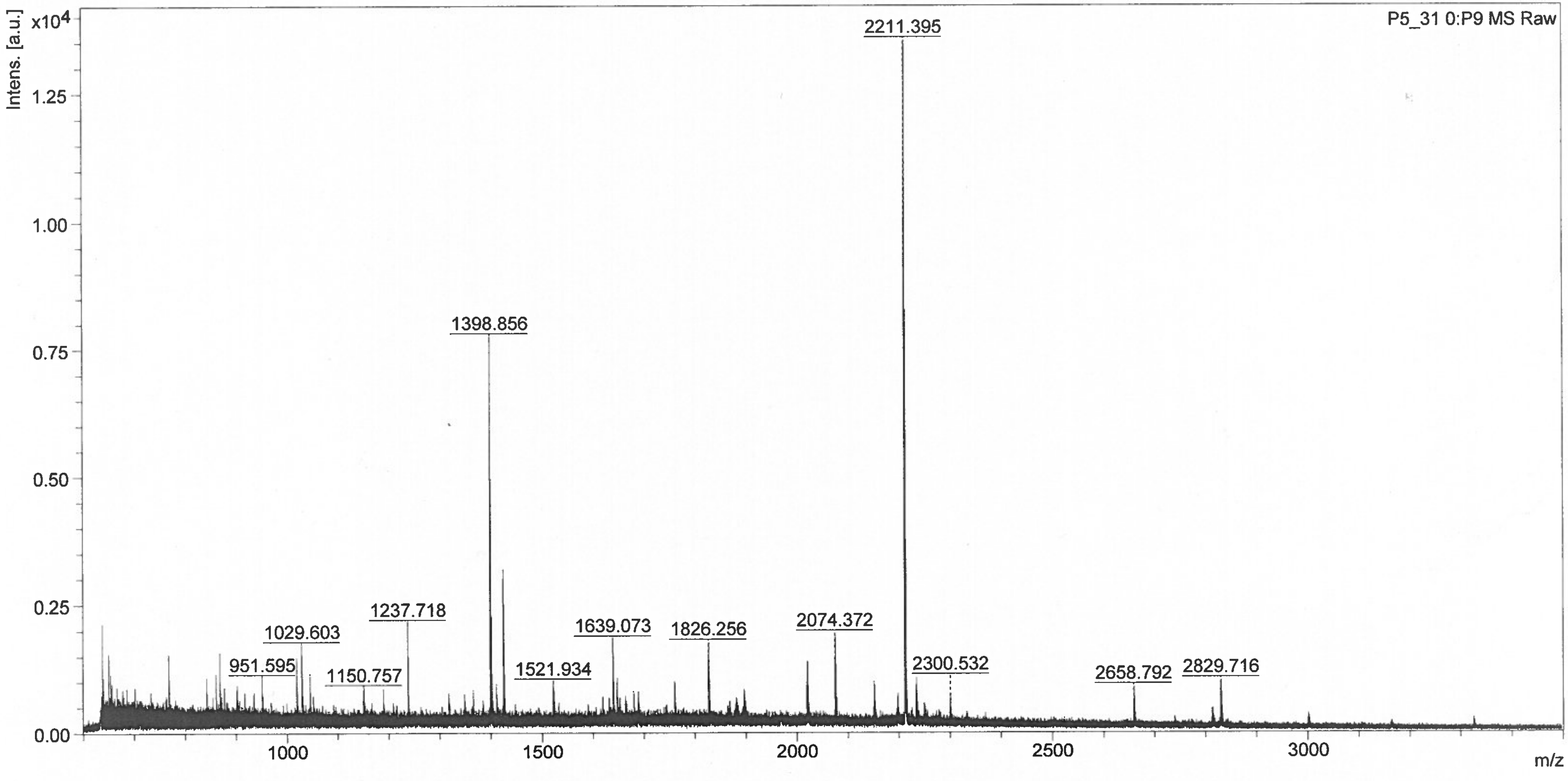

Spectrum Analysis Report

Date: 07/30/2011 Time: 13:35

FileName: D:\Data\Bernardo\2011_07_30\P5_31\0_P9\1\1SRef\pdata\1\PMF_LIFT.xml

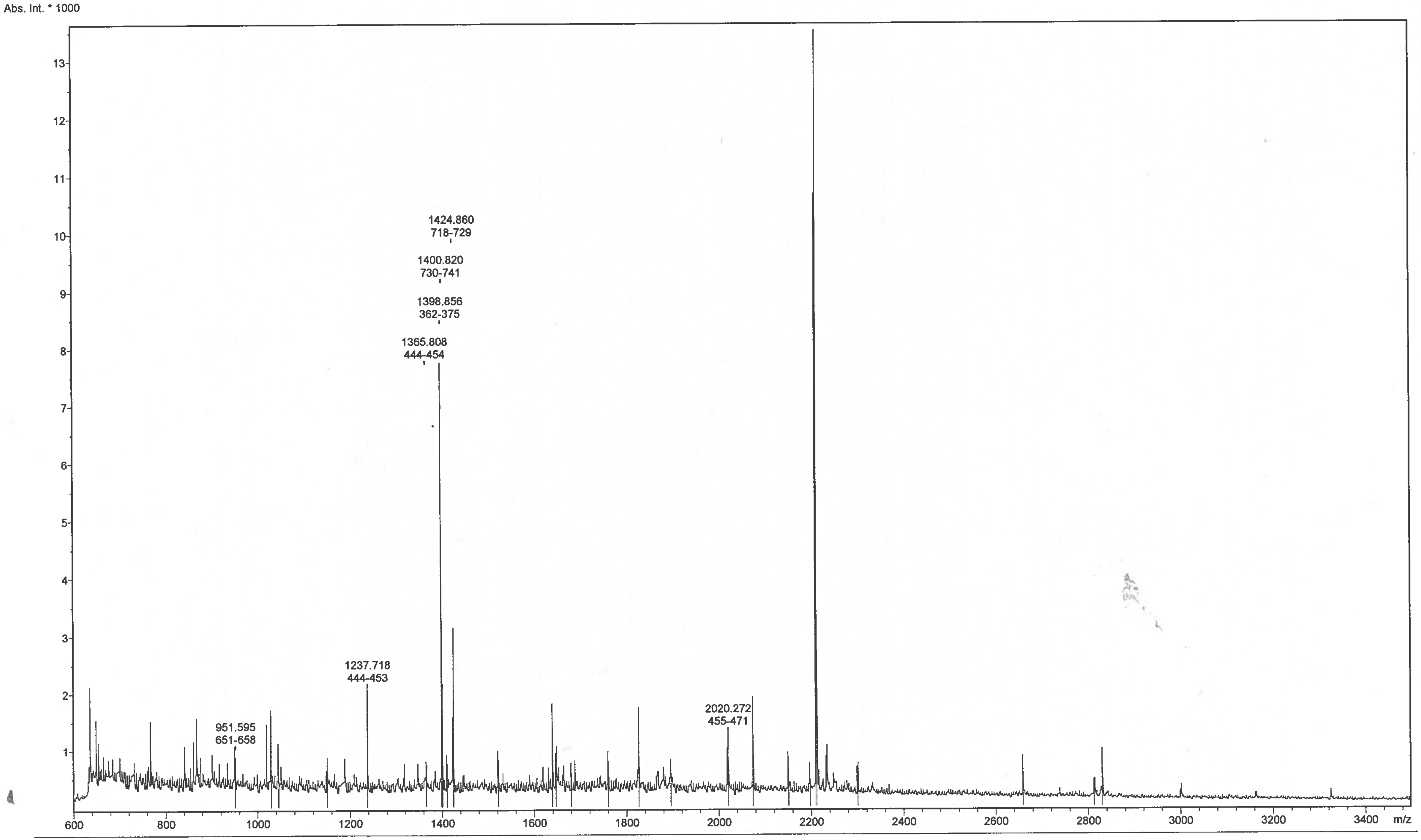

Copyright © 1999-2008 Bruker Daltonik GmbH

Sequence data:

Procollagen-lysine,2-oxoglutarate 5-dioxygenase 3 OS=Mus musculus GN=Plod3 PE=1 SV=1 PLOD3_MOUSE

Intensity Coverage:	37.1 % (14485 cnts)	Sequence Coverage MS:	10.0%
Sequence Coverage MS/MS:	5.1%	pI (isoelectric point):	5.8

10	20	30	40	50	60	70	80	90	100	110
MAAAGPEPRL	LLLLLLLLPP	LPPVTSASDR	PRGANAVNPD	KLLVITVATA	ETEGYRRFLQ	SAEFFNYTVR	TLGLGQEWRG	GDVARTVGGG	QKVRWLKKEM	EKYADQKDMI
120	130	140	150	160	170	180	190	200	210	220
IMFVDSYDVI	LASSPTELLK	KFVQSGSHLL	FSAESFCWPE	WGLAEQYPEV	GMGKRFLNSG	GFIGFAPTIH	QIVRQWNYKD	DDDDQLFYTQ	LYLDPGLREK	LKLSLDHKSR
230	240	250	260	270	280	290	300	310	320	330
IFQNLNGALD	EVILKFDQNR	VRIRNVAYDT	LPVVVHGNGP	TKLQLNYLGN	YVPNGWTPQG	GCGFCNQTLR	TLPGGQPPPR	VLLAVFVEQP	TPFLPRFLQR	LLLLDYPPDR
340	350	360	370	380	390	400	410	420	430	440
ISLFLHNSEV	YHEPHIADAW	PQLQDHFSAV	KLVGPEEALS	AGEARDMAMD	SCRQNPECEF	YFSLDADAVL	TNPETLRVLI	EQNRKVIAPM	LSRHGKLWSN	FWGALSPNEY
450	460	470	480	490	500	510	520	530	540	550
YARSEDYVEL	VQRKRVGVWN	VPYISQAYVI	RGETLRTELP	QKEVFSSSDT	DPDMAFCKSV	RDKGIFLHLS	NQHEFGRLLA	TSRYDTDHLH	PDLWQIFDNP	VDWREQYIHE
560	570	580	590	600	610	620	630	640	650	660
NYSRALDGEG	LVEQPCPDVY	WFPLLTEQMC	DELVEEMEHY	GQWSGGRHED	SRLAGGYENV	PTVDIHMKQV	GYEDQWLQLL	RTYVGPMTEY	LFPGYHTKTR	AVMNFVVRYR
670	680	690	700	710	720	730	740	750		
PDEQPSLRPH	HDSSTFTLNV	ALNHKGVDYE	GGGCRFLRYD	CRISSPRKGW	ALLHPGRLTH	YHEGLPTTRG	TRYIMVSFVD	P		

Acquisition Parameter:

Matched Sequences:

Unmatched

Peaks/MSMS Spectra

Tree hierarchy	Meas. M/z	Calc. MH+	Meas. Mr	Calc. Mr	Int.	z	Dev.(Da)	Dev.(ppm)	Score	MascotScore	Rt(min)	Range	P	Sequence
peak 2	1029.603	-	1028.596	-	1634.597	1+	-	-	-	-	-		-	
peak 3	1045.646	-	1044.639	-	860.741	1+	-	-	-	-	-		-	
peak 4	1150.757	-	1149.750	-	643.647	1+	-	-	-	-	-		-	
peak 9	1410.834	-	1409.826	-	756.678	1+	-	-	-	-	-		-	
peak 11	1521.934	-	1520.926	-	820.356	1+	-	-	-	-	-		-	
peak 12	1639.073	-	1638.065	-	1555.403	1+	-	-	-	-	-		-	
peak 13	1646.995	-	1645.988	-	785.154	1+	-	-	-	-	-		-	
peak 14	1680.037	-	1679.030	-	597.279	1+	-	-	-	-	-		-	
peak 15	1760.074	-	1759.066	-	792.550	1+	-	-	-	-	-		-	
peak 16	1826.256	-	1825.249	-	1322.964	1+	-	-	-	-	-		-	
peak 17	1896.188	-	1895.181	-	646.609	1+	-	-	-	-	-		-	
peak 19	2074.372	-	2073.364	-	1180.793	1+	-	-	-	-	-		-	
peak 20	2151.416	-	2150.409	-	666.816	1+	-	-	-	-	-		-	
peak 21	2197.275	-	2196.268	-	485.137	1+	-	-	-	-	-		-	
peak 22	2211.395	-	2210.388	-	8055.062	1+	-	-	-	-	-		-	
peak 23	2300.532	-	2299.525	-	459.653	1+	-	-	-	-	-		-	
peak 24	2658.792	-	2657.785	-	448.000	1+	-	-	-	-	-		-	
peak 25	2812.682	-	2811.675	-	270.221	1+	-	-	-	-	-		-	
peak 26	2829.716	-	2828.708	-	463.916	1+	-	-	-	-	-		-	

Global peptide results

Procollagen-lysine,2-oxoglutarate 5-dioxygenase 3 OS=Mus musculus GN=Plod3 PE=1 SV=1 PLOD3_MOUSE
MW:85438.850

MAAAGPEPRLLLLLLLLLPPLPPVTSASDRPRGANAVNPDKLLVITVATAETEGYRRFLQSAEFFNYTVRTLGLGQEWRGGDVARTVGGGQKVRWLKKEMEKYADQKDMIIMFVDSYDVILASSPTELLKKFVQSGSHLLFSAESFCWPEWGLAEQYPEVGMGKRFLNSGGFIGFAPTIHQIVRQWNYKDDDDDQLFYTQLYLDPGLREKL
KLSLDHKSRIFQNLNGALDEVILKFDQNRVRIRNVAYDTLPVVVHGNGPTKLQLNYLGNYVPNGWTPQGGCGFCNQTLRTLPGGQPPPRVLLAVFVEQPTPFLPRFLQRLLLLDYPPDRISLFLHNSEVYHEPHIADAWPQLQDHFSAVKLVGPEEALSAGEARDMAMDSCRQNPECEFYFSLDADAVLTNPETLRVLIEQNRKVIAPMLS
RHGKLWSNFWGALSPNEYYARSEDYVELVQRKRVGVWNVPYISQAYVIRGETLRTELPQKEVFSSSDTDPDMAFCKSVRDKGIFLHLSNQHEFGRLLATSRYDTDHLHPDLWQIFDNPVDWREQYIHENYSRALDGEGLVEQPCPDVYWFPLLTEQMCDELVEEMEHYGQWSGGRHEDSRLAGGYENVPTVDIHMKQVGYEDQWLQLLRTY
VGPMTEYLFPGYHTKTRAVMNFVVRYRPDEQPSLRPHHDSSTFTLNVALNHKGVDYEGGGCRFLRYDCRISSPRKGWALLHPGRLTHYHEGLPTTRGTRYIMVSFVDP

Digest Matches (Score: 78.50)
Score = 78.500000, Rank = 1, Database = SwissProt, Accesskey = PLOD3_MOUSE
Search Parameters: MS Tol.:100.00 ppm, MSMS Tol.:0.600000Da, Enz.Trypsin, Engine:Mascot Version:2.3.01.241, DB:NCBInr NCBInr, DB Version:NCBInr_20110715.fasta NCBInr_20110715.fasta
Modifications: Optional: Oxidation (M)

Copyright © 1999-2008 Bruker Daltonik GmbH

Spectrum Analysis Report

Date: 07/30/2011 Time: 13:35

FileName: D:\Data\Bernardo\2011_07_30\P5_31\0_P9\1\1SRef\pdata\1\PMF_LIFT.xml

Tree hierarchy	Meas. M/z	Calc. MH+	Meas. Mr	Calc. Mr	Int.	z	Dev.(Da)	Dev.(ppm)	Score	MascotScore	Rt(min)	Range	P	Sequence
peak 1	951.595	951.508	950.588	950.501	888.841	1+	0.087	91.847	-	-	-	651 - 658	0	AVMNFVVR 3: Oxidation (M)
peak 5	1237.718	1237.606	1236.711	1236.599	2073.528	1+	0.112	90.763	-	-	-	444 - 453	0	SEDYVELVQR
peak 6	1365.808	1365.701	1364.800	1364.694	678.660	1+	0.107	78.198	-	-	-	444 - 454	1	SEDYVELVQRK
MSMS 7	1398.856	1398.722	1397.849	1397.715	6959.712	1+	0.133	95.497	32	25	-	362 - 375	0	LVGPEEALSAGEAR
MSMS 8	1400.820	1400.688	1399.813	1399.681	2106.433	1+	0.132	94.293	6	-	-	730 - 741	1	GTRYIMVSFVDP 6: Oxidation (M)
MSMS 10	1424.860	1424.728	1423.853	1423.721	2905.369	1+	0.132	92.587	15	7	-	718 - 729	0	LTHYHEGLPTTR
peak 18	2020.272	2020.113	2019.265	2019.105	978.452	1+	0.160	79.000	-	-	-	455 - 471	1	RVGVWNVPYISQAYVIR

Copyright © 1999-2008 Bruker Daltonik GmbH
