# Supplementary material for: Proteomic Analysis of the Action of the Mycobacterium ulcerans Toxin Mycolactone: Targeting Host Cells Cytoskeleton and Collagen
Source: PLoS Negl Trop Dis. 2014 Aug 7;8(8):e3066. doi: 10.1371/journal.pntd.0003066 (PMC4125307; doi:10.1371/journal.pntd.0003066)
Supplement: Dataset S7 — MS and MS/MS data. (ZIP) [file pntd.0003066.s010.zip › MS Data/Spot 06 - Crmp2.pdf]

D:\Data\Bernardo\2011_07_30\P5_32\0_P10\1\1SRef

Comment 1
Comment 2

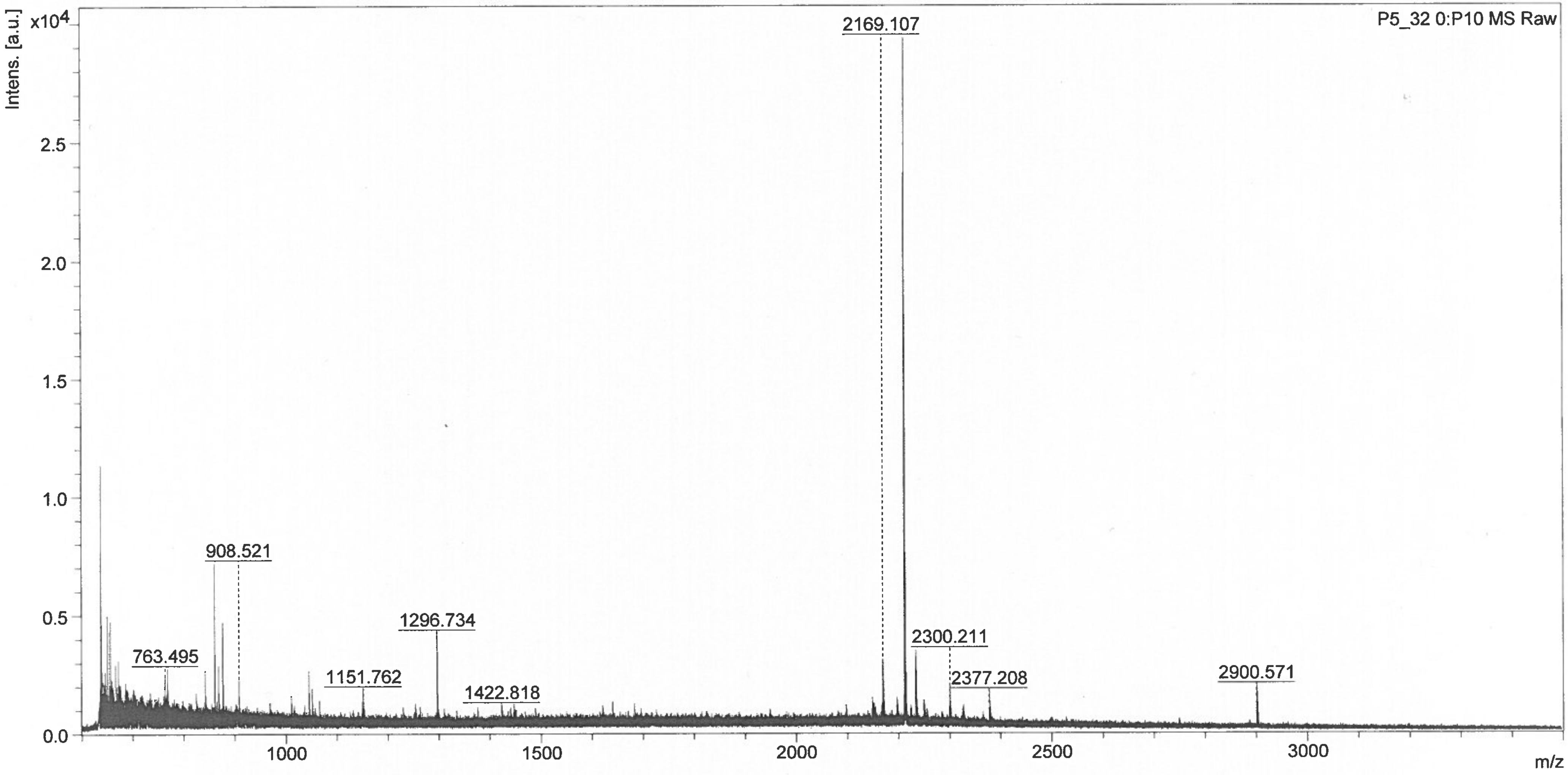

Bruker Daltonics flexAnalysis

printed: 7/30/2011 1:39:13 PM

Spectrum Analysis Report

Date: 07/30/2011 Time: 13:38

FileName: D:\Data\Bernardo\2011_07_30\P5_32\0_P10\1\1SRef\pdata\1\PMF_LIFT.xml

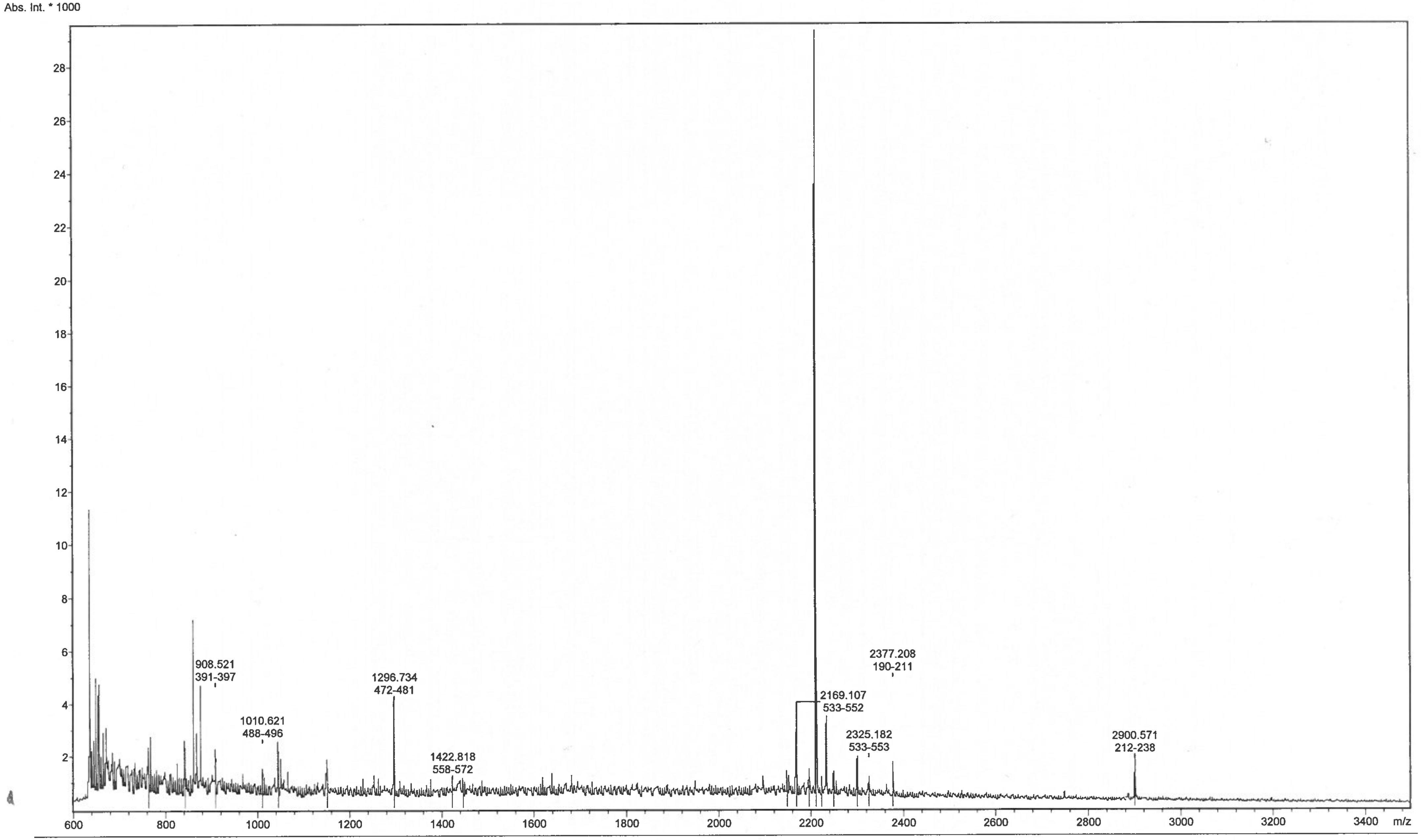

Copyright © 1999-2008 Bruker Daltonik GmbH

Spectrum Analysis Report

Date: 07/30/2011 Time: 13:38

FileName: D:\Data\Bernardo\2011_07_30\P5_32\0_P10\1\1SRef\pdata\1\PMF_LIFT.xml

Sequence data:

Dihydropyrimidinase-related protein 2 OS=Mus musculus GN=Dpysl2 PE=1 SV=2 DPYL2_MOUSE

Intensity Coverage:	27.2 % (13970 cnts)	Sequence Coverage MS:	19.4%
Sequence Coverage MS/MS:	5.2%	pI (isoelectric point):	5.9

```
10          20          30          40          50          60          70          80          90          100         110
MSYQGKKNIP  RITSDRLLIK  GGKIVNDDQS  FYADIYMEDG  LIKQIGENLI  VPGGVKTIEA  HSRMVIPGGI  DVHTRFQMPD  QGMTSADDFF  QGTKAALAGG  TTMIIDHVVP
120         130         140         150         160         170         180         190         200         210         220
EPGTSLLAAF  DQWREWADSK  SCCDYSLHVD  ITEWHKGIQE  EMEALVKDHG  VNSFLVYMAF  KDRFQLTDSQ  IYEVLSVIRD  IGAIAQVHAE  NGDIIAEEQQ  RILDLGITGP
230         240         250         260         270         280         290         300         310         320         330
EGHVLSRPEE  VEAEAVNRSI  TIANQTNCPL  YVTKVMSKSA  AEVIAQARKK  GTVVYGEPIT  ASLGTDGSHY  WSKNWAKAAA  FVTSPPLSPD  PTTPDFLNSL  LSCGDLQVTG
340         350         360         370         380         390         400         410         420         430         440
SAHCTFNTAQ  KAVGKDNFTL  IPEGTNGTEE  RMSVIWDKAV  VTGKMDENQF  VAVTSTNAAK  VFNLYPRKGR  ISVGSDADLV  IWDPDSVKTI  SAKTHNSALE  YNIFEGMECR
450         460         470         480         490         500         510         520         530         540         550
GSPLVVISQG  KIVLEDGTLH  VTEGSGRYIP  RKPFPDFVYK  RIKARSRLAE  LRGVPRGLYD  GPVCEVSVTP  KTVTPASSAK  TSPAKQQAPP  VRNLHQSGFS  LSGAQIDDNI
560         570         580
PRRTTQRIVA  PPGGRANITS  LG
```

Acquisition Parameter:

Matched Sequences:

Unmatched

Peaks/MSMS Spectra

Tree hierarchy	Meas. M/z	Calc. MH+	Meas. Mr	Calc. Mr	Int.	z	Dev.(Da)	Dev.(ppm)	Score	MascotScore	Rt(min)	Range	P	Sequence
peak 1	763.495	-	762.488	-	1877.869	1+	-	-	-	-	-		-	
peak 2	842.528	-	841.521	-	2319.505	1+	-	-	-	-	-		-	
peak 5	1045.584	-	1044.577	-	2194.817	1+	-	-	-	-	-		-	
peak 6	1151.762	-	1150.755	-	1542.595	1+	-	-	-	-	-		-	
peak 9	1446.881	-	1445.874	-	932.726	1+	-	-	-	-	-		-	
peak 10	2149.074	-	2148.067	-	1127.467	1+	-	-	-	-	-		-	
peak 12	2196.094	-	2195.087	-	875.092	1+	-	-	-	-	-		-	
peak 13	2211.151	-	2210.144	-	23192.211	1+	-	-	-	-	-		-	
peak 14	2223.136	-	2222.128	-	829.325	1+	-	-	-	-	-		-	
peak 15	2249.106	-	2248.098	-	953.366	1+	-	-	-	-	-		-	
peak 16	2300.211	-	2299.204	-	1451.775	1+	-	-	-	-	-		-	

Global peptide results

Dihydropyrimidinase-related protein 2 OS=Mus musculus GN=Dpysl2 PE=1 SV=2 DPYL2_MOUSE

MW:62637.740

MSYQGKKNIPRITSDRLLIKGGKIVNDDQSFYADIYMEDGLIKQIGENLIVPGGVKTIEAHSRMVIPGGIDVHTRFQMPDQGMTSADDFFQGTKAALAGGTTMIIDHVVPEPGTSLLAAFDQWREWADSKSCCDYSLHVDITEWHKGIQEEMEALVKDHGVNSFLVYMAFKDRFQLTDSQIYEVLSVIRDIGAIAQVHAENGDIIAEEQQR
ILDLGITGPEGHVLSRPEEVEAEAVNRSITIANQTNCPLYVTKVMSKSAAEVIAQARKKGTVVYGEPITASLGTDGSHYWSKNWAKAAAFVTSPPLSPDPTTPDFLNSLLSCGDLQVTGSAHCTFNTAQKAVGKDNFTLIPEGTNGTEERMSVIWDKAVVTGKMDENQFVAVTSTNAAKVFNLYPRKGRISVGSDADLVIWDPDSVKTISA
KTHNSALEYNIFEGMECRGSPLVVISQGKIVLEDGTLHVTEGSGRYIPRKPFPDFVYKRIKARSRLAELRGVPRGLYDGPVCEVSVTPKTVTPASSAKTSPAKQQAPPVRNLHQSGFSLSGAQIDDNIPRRTTQRIVAPPGGRANITSLG

Digest Matches (Score: 94.90)

Score = 94.900000, Rank = 1, Database = SwissProt, Accesskey = DPYL2_MOUSE

Search Parameters: MS Tol.:100.00 ppm, MSMS Tol.:0.600000Da, Enz.Trypsin, Engine:Mascot Version:2.3.01.241, DB:NCBInr NCBInr, DB Version:NCBInr_20110715.fasta NCBInr_20110715.fasta

Modifications: Optional: Oxidation (M)

Tree hierarchy	Meas. M/z	Calc. MH+	Meas. Mr	Calc. Mr	Int.	z	Dev.(Da)	Dev.(ppm)	Score	MascotScore	Rt(min)	Range	P	Sequence
peak 3	908.521	908.499	907.514	907.492	1793.437	1+	0.023	24.905	-	-	-	391 - 397	0	VFNLYPR
peak 4	1010.621	1010.611	1009.614	1009.603	1207.223	1+	0.010	10.308	-	-	-	488 - 496	1	LAELRGVPR
MSMS 7	1296.734	1296.710	1295.727	1295.703	4186.013	1+	0.024	18.581	12	-	-	472 - 481	1	KPFPDFVYKR
peak 8	1422.818	1422.806	1421.811	1421.799	1037.161	1+	0.012	8.519	-	-	-	558 - 572	1	IVAPPGGRANITSLG
MSMS 11	2169.107	2169.068	2168.100	2168.061	2453.885	1+	0.038	17.751	9	5	-	533 - 552	0	NLHQSGFSLSGAQIDDNIPR
peak 17	2325.182	2325.169	2324.174	2324.162	835.515	1+	0.012	5.314	-	-	-	533 - 553	1	NLHQSGFSLSGAQIDDNIPRR
peak 18	2377.208	2377.174	2376.201	2376.167	1223.626	1+	0.034	14.140	-	-	-	190 - 211	0	DIGAIAQVHAENGDIIAEEQQR
MSMS 19	2900.571	2900.511	2899.564	2899.504	1232.990	1+	0.060	20.768	-	-	-	212 - 238	0	ILDLGITGPEGHVLSRPEEVEAEAVNR

Copyright © 1999-2008 Bruker Daltonik GmbH
