# Supplementary material for: Proteomic Analysis of the Action of the Mycobacterium ulcerans Toxin Mycolactone: Targeting Host Cells Cytoskeleton and Collagen
Source: PLoS Negl Trop Dis. 2014 Aug 7;8(8):e3066. doi: 10.1371/journal.pntd.0003066 (PMC4125307; doi:10.1371/journal.pntd.0003066)
Supplement: Dataset S7 — MS and MS/MS data. (ZIP) [file pntd.0003066.s010.zip › MS Data/Spot 07 - Crmp2.pdf]

D:\Data\Bernardo\2011_07_30\P5_20\0_P8\1\1SRef

Comment 1

Comment 2

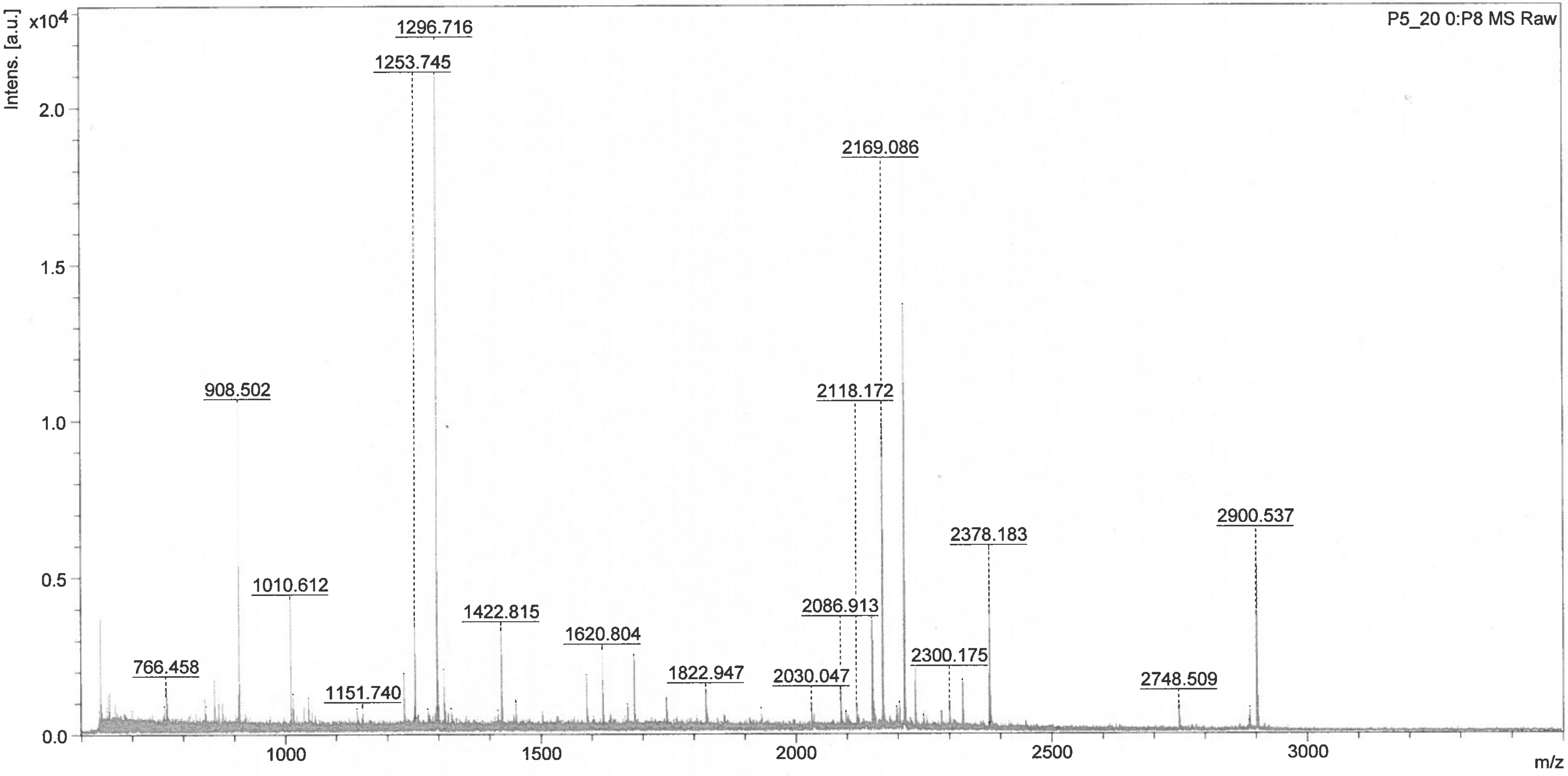

Bruker Daltonics flexAnalysis

printed: 7/30/2011 1:27:47 PM

Spectrum Analysis Report

Date: 07/30/2011 Time: 13:27

FileName: D:\Data\Bernardo\2011_07_30\P5_20\0_P8\1\1SRef\pdata\1\PMF_LIFT.xml

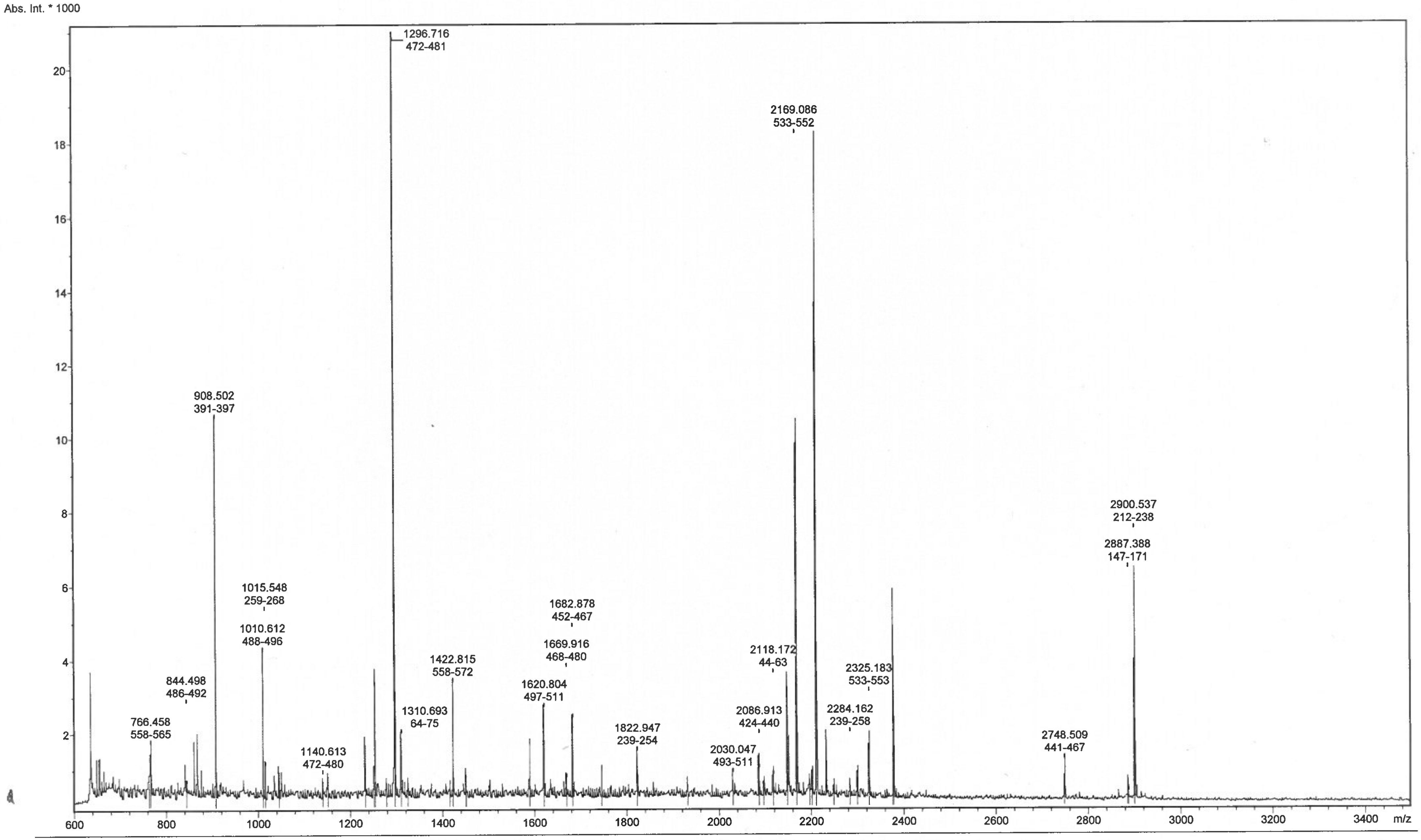

Copyright © 1999-2008 Bruker Daltonik GmbH

Spectrum Analysis Report

Date: 07/30/2011 Time: 13:27

FileName: D:\Data\Bernardo\2011_07_30\P5_20\0_P8\1\1SRef\pdata\1\PMF_LIFT.xml

Sequence data:

Dihydropyrimidinase-related protein 2 OS=Mus musculus GN=Dpysl2 PE=1 SV=2 DPYL2_MOUSE

Intensity Coverage:	63.5 % (71141 cnts)	Sequence Coverage MS:	42.1%
Sequence Coverage MS/MS:	11.2%	pI (isoelectric point):	5.9

10	20	30	40	50	60
MSYQGKKNIP	RITSDRLLIK	GGKIVNDDQS	FYADIYMEDG	LIKQIGENLI	VPGGVKTIEA
**70**	**80**	**90**	**100**	**110**	**120**
HSRMVIPGGI	DVHTRFQMPD	QGMTSADDFF	QGTKAALAGG	TTMIIDHVVP	EPGTSLLAAF
**130**	**140**	**150**	**160**	**170**	**180**
DQWREWADSK	SCCDYSLHVD	ITEWHKGIQE	EMEALVKDHG	VNSFLVYMAF	KDRFQLTDSQ
**190**	**200**	**210**	**220**	**230**	**240**
IYEVLSVIRD	IGAIAQVHAE	NGDIIAEEQQ	RILDLGITGP	EGHVLSRPEE	VEAEAVNRSI
**250**	**260**	**270**	**280**	**290**	**300**
TIANQTNCPL	YVTKVMSKSA	AEVIAQARKK	GTVVYGEPIT	ASLGTDGSHY	WSKNWAKAAA
**310**	**320**	**330**	**340**	**350**	**360**
FVTSPPLSPD	PTTPDFLNSL	LSCGDLQVTG	SAHCTFNTAQ	KAVGKDNFTL	IPEGTNGTEE
**370**	**380**	**390**	**400**	**410**	**420**
RMSVIWDKAV	VTGKMDENQF	VAVTSTNAAK	VFNLYPRKGR	ISVGSDADLV	IWDPDSVKTI
**430**	**440**	**450**	**460**	**470**	**480**
SAKTHNSALE	YNIFEGMECR	GSPLVVISQG	KIVLEDGTLH	VTEGSGRYIP	RKPFPDFVYK
**490**	**500**	**510**	**520**	**530**	**540**
RIKARSRLAE	LRGVPRGLYD	GPVCEVSVTP	KTVTPASSAK	TSPAKQQAPP	VRNLHQSGFS
**550**	**560**	**570**	**580**		
LSGAQIDDNI	PRRTTQRIVA	PPGGRANITS	LG		

Acquisition Parameter:

Matched Sequences:

Unmatched

Peaks/MSMS Spectra

Tree hierarchy	Meas. M/z	Calc. MH+	Meas. Mr	Calc. Mr	Int.	z	Dev.(Da)	Dev.(ppm)	Score	MascotScore	Rt(min)	Range	P	Sequence
peak 1	763.472	-	762.465	-	653.418	1+	-	-	-	-	-		-	
peak 7	1045.565	-	1044.557	-	949.851	1+	-	-	-	-	-		-	
peak 9	1151.740	-	1150.733	-	820.907	1+	-	-	-	-	-		-	
peak 10	1232.675	-	1231.668	-	1680.382	1+	-	-	-	-	-		-	
peak 11	1251.734	-	1250.727	-	663.207	1+	-	-	-	-	-		-	
peak 12	1253.745	-	1252.737	-	3420.413	1+	-	-	-	-	-		-	
peak 13	1278.703	-	1277.695	-	638.549	1+	-	-	-	-	-		-	
peak 16	1324.705	-	1323.698	-	646.845	1+	-	-	-	-	-		-	
peak 17	1415.868	-	1414.861	-	617.593	1+	-	-	-	-	-		-	
peak 19	1450.773	-	1449.766	-	864.557	1+	-	-	-	-	-		-	
peak 20	1451.270	-	1450.263	-	858.263	1+	-	-	-	-	-		-	
peak 21	1589.801	-	1588.793	-	1687.147	1+	-	-	-	-	-		-	
peak 25	1745.894	-	1744.886	-	991.083	1+	-	-	-	-	-		-	
peak 27	1932.008	-	1931.000	-	571.444	1+	-	-	-	-	-		-	
peak 30	2097.133	-	2096.126	-	594.889	1+	-	-	-	-	-		-	
peak 32	2149.055	-	2148.048	-	3243.343	1+	-	-	-	-	-		-	
peak 34	2197.052	-	2196.045	-	525.798	1+	-	-	-	-	-		-	
peak 35	2202.141	-	2201.134	-	737.212	1+	-	-	-	-	-		-	

Copyright © 1999-2008 Bruker Daltonik GmbH

peak 36	2211.125	-	2210.118	-	13919.148	1+	-	-	-	-	-		-	
peak 37	2249.067	-	2248.059	-	498.459	1+	-	-	-	-	-		-	
peak 39	2300.175	-	2299.167	-	726.253	1+	-	-	-	-	-		-	
peak 41	2376.198	-	2375.191	-	1427.177	1+	-	-	-	-	-		-	
MSMS 42	2378.183	-	2377.176	-	3782.408	1+	-	-	-	-	-		-	
peak 44	2885.413	-	2884.406	-	282.261	1+	-	-	-	-	-		-	

Global peptide results

Dihydropyrimidinase-related protein 2 OS=Mus musculus GN=Dpysl2 PE=1 SV=2 DPYL2_MOUSE
MW:62637.740
MSYQGKKNIPRITSDRLLIKGGKIVNDDQSFYADIYMEDGLIKQIGENLIVPGGVKTIEAHSRMVIPGGIDVHTRFQMPDQGMTSADDFFQGTKAALAGGTTMIIDHVVPEPGTSLLAAFDQWREWADSKSCCDYSLHVDITEWHKGIQEEMEALVKDHGVNSFLVYMAFKDRFQLTDSQIYEVLSVIRDIGAIAQVHAENGDIIAEEQQR
ILDLGITGPEGHVLSRPEEVEAEAVNRSITIANQTNCPLYVTKVMSKSAAEVIAQARKKGTVVYGEPITASLGTDGSHYWSKNWAKAAAFVTSPPLSPDPTTPDFLNSLLSCGDLQVTGSAHCTFNTAQKAVGKDNFTLIPEGTNGTEERMSVIWDKAVVTGKMDENQFVAVTSTNAAKVFNLYPRKGRISVGSDADLVIWDPDSVKTISA
KTHNSALEYNIFEGMECRGSPLVVISQGKIVLEDGTLHVTEGSGRYIPRKPFPDFVYKRIKARSRLAELRGVPRGLYDGPVCEVSVTPKTVTPASSAKTSPAKQQAPPVRNLHQSGFSLSGAQIDDNIPRRTTQRIVAPPGGRANITSLG

Digest Matches (Score: 357.00)
Score = 357.000000, Rank = 1, Database = SwissProt, Accesskey = DPYL2_MOUSE
Search Parameters: MS Tol.:100.00 ppm, MSMS Tol.:0.600000Da, Enz.Trypsin, Engine:Mascot Version:2.3.01.241, DB:NCBInr NCBInr, DB Version:NCBInr_20110715.fasta NCBInr_20110715.fasta
Modifications: Optional: Oxidation (M)

Tree hierarchy	Meas. M/z	Calc. MH+	Meas. Mr	Calc. Mr	Int.	z	Dev.(Da)	Dev.(ppm)	Score	MascotScore	Rt(min)	Range	P	Sequence
peak 2	766.458	766.457	765.450	765.450	1202.652	1+	0.001	0.792	-	-	-	558 - 565	0	IVAPPGGR
peak 3	844.498	844.500	843.491	843.493	643.315	1+	-0.001	-1.653	-	-	-	486 - 492	1	SRLAELR
MSMS 4	908.502	908.499	907.495	907.492	10590.225	1+	0.003	3.639	36	12	-	391 - 397	0	VFNLYPR
peak 5	1010.612	1010.611	1009.605	1009.603	4292.359	1+	0.001	1.405	-	-	-	488 - 496	1	LAELRGVPR
peak 6	1015.548	1015.553	1014.541	1014.546	1137.142	1+	-0.005	-4.714	-	-	-	259 - 268	0	SAAEVIAQAR
peak 8	1140.613	1140.609	1139.606	1139.602	686.902	1+	0.004	3.630	-	-	-	472 - 480	0	KPFPDFVYK
MSMS 14	1296.716	1296.710	1295.709	1295.703	22124.054	1+	0.006	4.663	61	32	-	472 - 481	1	KPFPDFVYKR
peak 15	1310.693	1310.689	1309.686	1309.681	1872.512	1+	0.005	3.493	-	-	-	64 - 75	0	MVIPGGIDVHTR 1: Oxidation (M)
peak 18	1422.815	1422.806	1421.808	1421.799	3277.559	1+	0.009	6.037	-	-	-	558 - 572	1	IVAPPGGRANITSLG
peak 22	1620.804	1620.794	1619.797	1619.787	2669.121	1+	0.010	6.366	-	-	-	497 - 511	0	GLYDGPVCEVSVTPK 8: Carbamidomethyl (C)
peak 23	1669.916	1669.910	1668.908	1668.903	787.045	1+	0.006	3.378	-	-	-	468 - 480	1	YIPRKPFPDFVYK
peak 24	1682.878	1682.871	1681.871	1681.864	2431.129	1+	0.008	4.512	-	-	-	452 - 467	0	IVLEDGTLHVTEGSGR
peak 26	1822.947	1822.937	1821.940	1821.929	1299.408	1+	0.011	5.797	-	-	-	239 - 254	0	SITIANQTNCPLYVTK 10: Carbamidomethyl (C)
peak 28	2030.047	2030.038	2029.040	2029.030	695.562	1+	0.010	4.827	-	-	-	493 - 511	1	GVPRGLYDGPVCEVSVTPK 12: Carbamidomethyl (C)
peak 29	2086.913	2086.896	2085.905	2085.888	1039.190	1+	0.017	8.112	-	-	-	424 - 440	0	THNSALEYNIFEGMECR 14: Oxidation (M) 16: Carbamidomethyl (C)
peak 31	2118.172	2118.167	2117.165	2117.159	855.429	1+	0.006	2.633	-	-	-	44 - 63	1	QIGENLIVPGGVKTIEAHSR
MSMS 33	2169.086	2169.068	2168.079	2168.061	8781.904	1+	0.018	8.272	86	36	-	533 - 552	0	NLHQSGFSLSGAQIDDNIPR
peak 38	2284.162	2284.168	2283.154	2283.160	437.437	1+	-0.006	-2.534	-	-	-	239 - 258	1	SITIANQTNCPLYVTKVMSK 18: Oxidation (M) 10: Carbamidomethyl (C)
peak 40	2325.183	2325.169	2324.176	2324.162	1545.635	1+	0.014	6.022	-	-	-	533 - 553	1	NLHQSGFSLSGAQIDDNIPRR
peak 43	2748.509	2748.489	2747.502	2747.482	733.741	1+	0.020	7.360	-	-	-	441 - 467	1	GSPLVVISQGKIVLEDGTLHVTEGSGR
peak 45	2887.388	2887.400	2886.381	2886.393	368.829	1+	-0.012	-4.301	-	-	-	147 - 171	1	GIQEEMEALVKDHGVNSFLVYMAFK 6: Oxidation (M) 22: Oxidation (M)
MSMS 46	2900.537	2900.511	2899.529	2899.504	3756.545	1+	0.025	8.766	33	45	-	212 - 238	0	ILDLGITGPEGHVLSRPEEVEAEAVNR

Copyright © 1999-2008 Bruker Daltonik GmbH
