# Supplementary material for: Proteomic Analysis of the Action of the Mycobacterium ulcerans Toxin Mycolactone: Targeting Host Cells Cytoskeleton and Collagen
Source: PLoS Negl Trop Dis. 2014 Aug 7;8(8):e3066. doi: 10.1371/journal.pntd.0003066 (PMC4125307; doi:10.1371/journal.pntd.0003066)
Supplement: Dataset S7 — MS and MS/MS data. (ZIP) [file pntd.0003066.s010.zip › MS Data/Spot 08 - Actg1.pdf]

D:\Data\Bernardo\2011_07_30\P5_33\0_O11\1\1SRef

Comment 1

Comment 2

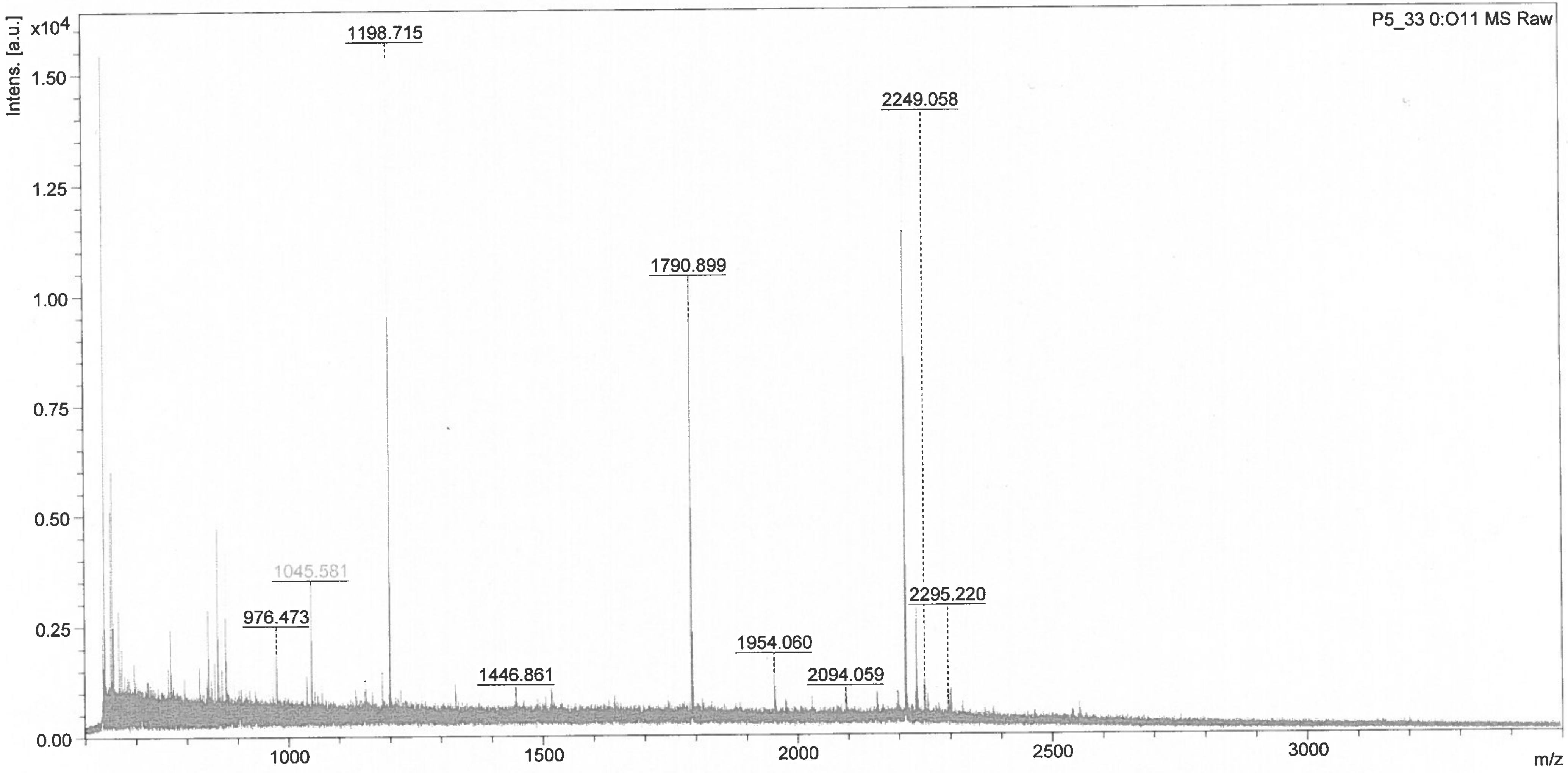

Bruker Daltonics flexAnalysis

printed: 7/30/2011 1:44:49 PM

Spectrum Analysis Report

Date: 07/30/2011 Time: 13:45

FileName: D:\Data\Bernardo\2011_07_30\P5_33\0_O11\1\1SRef\pdata\1\PMF_LIFT.xml

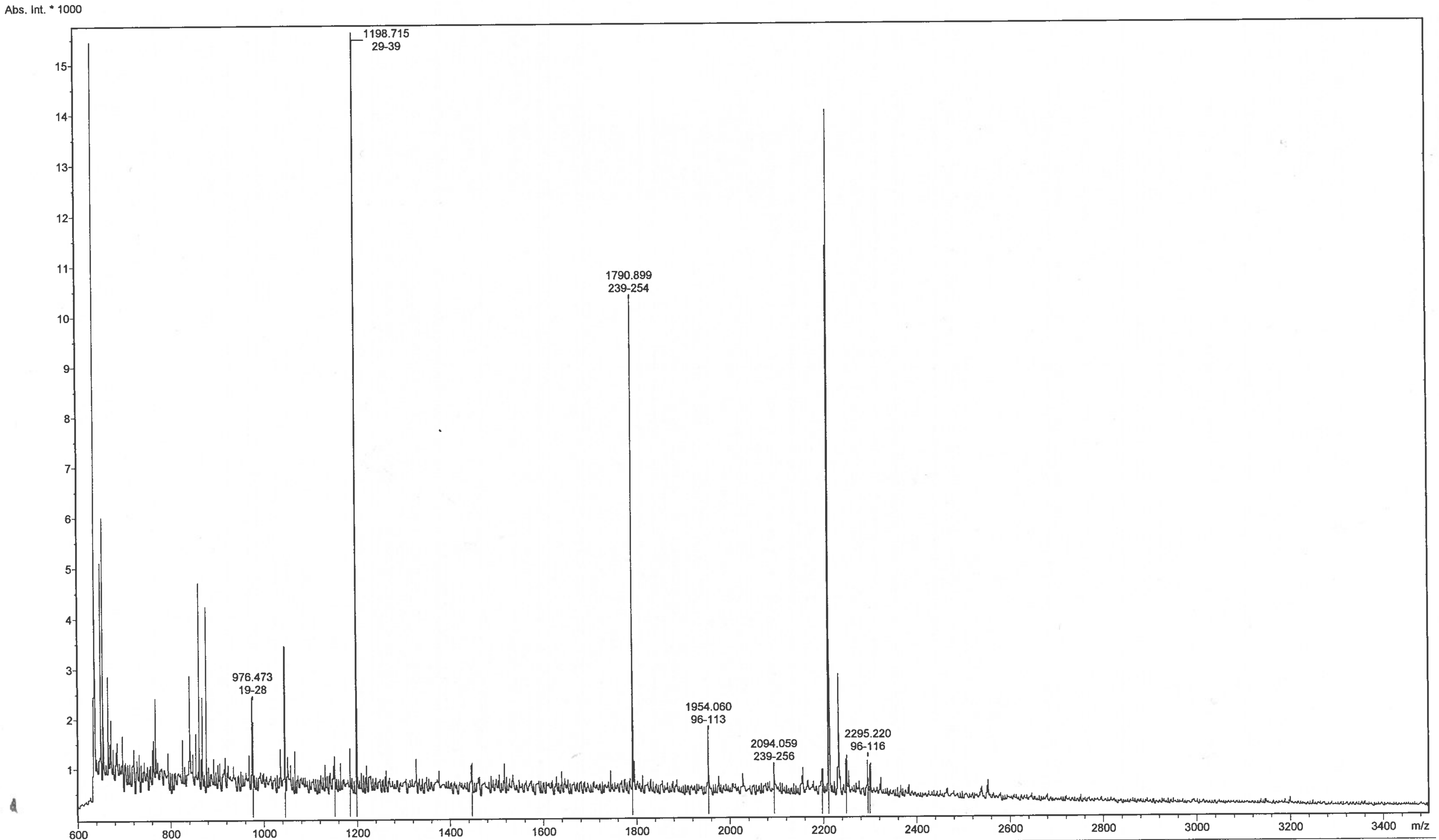

Copyright © 1999-2008 Bruker Daltonik GmbH

Spectrum Analysis Report

Date: 07/30/2011 Time: 13:45

FileName: D:\Data\Bernardo\2011_07_30\P5_33\0_O11\1\1SRef\pdata\1\PMF_LIFT.xml

Sequence data:

unnamed protein product [Mus musculus] gi|74187644

Intensity Coverage:	61.7 % (29271 cnts)	Sequence Coverage MS:	16.0%
Sequence Coverage MS/MS:	7.2%	pI (isoelectric point):	5.1

10	20	30	40	50	60	70	80	90	100	110
MEEEIAALVI	DNGSGMCKAG	FAGDDAPRAV	FPSIVGRPRH	QGVMVGMGQK	DSYVGDEAQS	KTGILTLKYP	IEHGIVTNWD	DMEKIWHHTF	YNELRVAPEE	HPVLLTEAPL
120	130	140	150	160	170	180	190	200	210	220
NPKANREKMT	QIMFETFNTP	AMYVAIQAVL	SLYASGRTTG	IVMDSGDGVT	HTVPIYEGYA	LPHAILRLDL	AGRDLTDYLM	KILTERGYSF	TTTAEREIVR	DIKEKLCYVA
230	240	250	260	270	280	290	300	310	320	330
LDFEQEMATA	ASSSSLEKSY	ELPDGQVITI	GNERFRCPEA	LFQPSFLGME	SCGIHETTFN	SIMKCDVDIR	KDLYANTVLS	GGTTMYPGIA	DRMQKEITAL	APSTMKIKII
340	350	360	370	380						
APPERKYSVW	IGGSILASLS	TFQQMWISKQ	EYDESGPSIV	HRKCF						

Acquisition Parameter:

Matched Sequences:

Unmatched

Peaks/MSMS Spectra

Tree hierarchy	Meas. M/z	Calc. MH+	Meas. Mr	Calc. Mr	Int.	z	Dev.(Da)	Dev.(ppm)	Score	MascotScore	Rt(min)	Range	P	Sequence
peak 2	1045.581	-	1044.574	-	3442.546	1+	-	-	-	-	-		-	
peak 3	1151.748	-	1150.741	-	1005.478	1+	-	-	-	-	-		-	
peak 4	1184.671	-	1183.664	-	952.344	1+	-	-	-	-	-		-	
peak 6	1446.861	-	1445.853	-	885.320	1+	-	-	-	-	-		-	
peak 10	2197.022	-	2196.015	-	676.369	1+	-	-	-	-	-		-	
peak 11	2211.101	-	2210.094	-	9797.480	1+	-	-	-	-	-		-	
peak 12	2249.058	-	2248.051	-	743.627	1+	-	-	-	-	-		-	
peak 14	2299.149	-	2298.142	-	643.606	1+	-	-	-	-	-		-	

Global peptide results

unnamed protein product [Mus musculus] gi|74187644
MW:42052.870

MEEEIAALVIDNGSGMCKAGFAGDDAPRAVFPSIVGRPRHQGVMVGMGQKDSYVGDEAQSKTGILTLKYPIEHGIVTNWDDMEKIWHHTFYNELRVAPEEHPVLLTEAPLNPKANREKMTQIMFETFNTPAMYVAIQAVLSLYASGRTTGIVMDSGDGVTHTVPIYEGYALPHAILRLDLAGRDLTDYLMKILTERGYSFTTTAEREIVRD
IKEKLCYVALDFEQEMATAASSSSLEKSYELPDGQVITIGNERFRCPEALFQPSFLGMESCGIHETTFNSIMKCDVDIRKDLYANTVLSGGTTMYPGIADRMQKEITALAPSTMKIKIIAPPERKYSVWIGGSILASLSTFQQMWISKQEYDESGPSIVHRKCF

Digest Matches (Score: 85.00)
Score = 85.000000, Rank = 1, Database = NCBInr, Accesskey = gi|74187644
Search Parameters: MS Tol.:100.00 ppm, MSMS Tol.:0.600000Da, Enz.Trypsin, Engine:Mascot Version:2.3.01.241, DB:NCBInr NCBInr, DB Version:NCBInr_20110715.fasta NCBInr_20110715.fasta
Modifications: Optional: Oxidation (M)

Tree hierarchy	Meas. M/z	Calc. MH+	Meas. Mr	Calc. Mr	Int.	z	Dev.(Da)	Dev.(ppm)	Score	MascotScore	Rt(min)	Range	P	Sequence
peak 1	976.473	976.448	975.466	975.441	1865.460	1+	0.025	25.823	-	-	-	19 - 28	0	AGFAGDDAPR
MSMS 5	✓1198.715	1198.706	1197.707	1197.698	15339.090	1+	0.009	7.598	23	8	-	29 - 39	0	AVFPSIVGRPR
MSMS 7	✓1790.899	1790.892	1789.892	1789.885	9328.836	1+	0.007	4.042	7	-	-	239 - 254	0	SYELPDGQVITIGNER
peak 8	✓1954.060	1954.064	1953.053	1953.057	1391.028	1+	-0.004	-2.238	-	-	-	96 - 113	0	VAPEEHPVLLTEAPLNPK
peak 9	✓2094.059	2094.061	2093.051	2093.054	695.574	1+	-0.003	-1.362	-	-	-	239 - 256	1	SYELPDGQVITIGNERFR
peak 13	✓2295.220	2295.246	2294.212	2294.238	651.450	1+	-0.026	-11.244	-	-	-	96 - 116	1	VAPEEHPVLLTEAPLNPKANR

Copyright © 1999-2008 Bruker Daltonik GmbH
