# Supplementary material for: Proteomic Analysis of the Action of the Mycobacterium ulcerans Toxin Mycolactone: Targeting Host Cells Cytoskeleton and Collagen
Source: PLoS Negl Trop Dis. 2014 Aug 7;8(8):e3066. doi: 10.1371/journal.pntd.0003066 (PMC4125307; doi:10.1371/journal.pntd.0003066)
Supplement: Dataset S7 — MS and MS/MS data. (ZIP) [file pntd.0003066.s010.zip › MS Data/Spot 09 - Prdx4.pdf]

Spectrum Analysis Report

Date: 07/29/2011 Time: 07:39

FileName: D:\Data\Bernardo\2011_07_28\M5_34\0_K15\1\1SRef\pdata\1\PMF_LIFT.xml

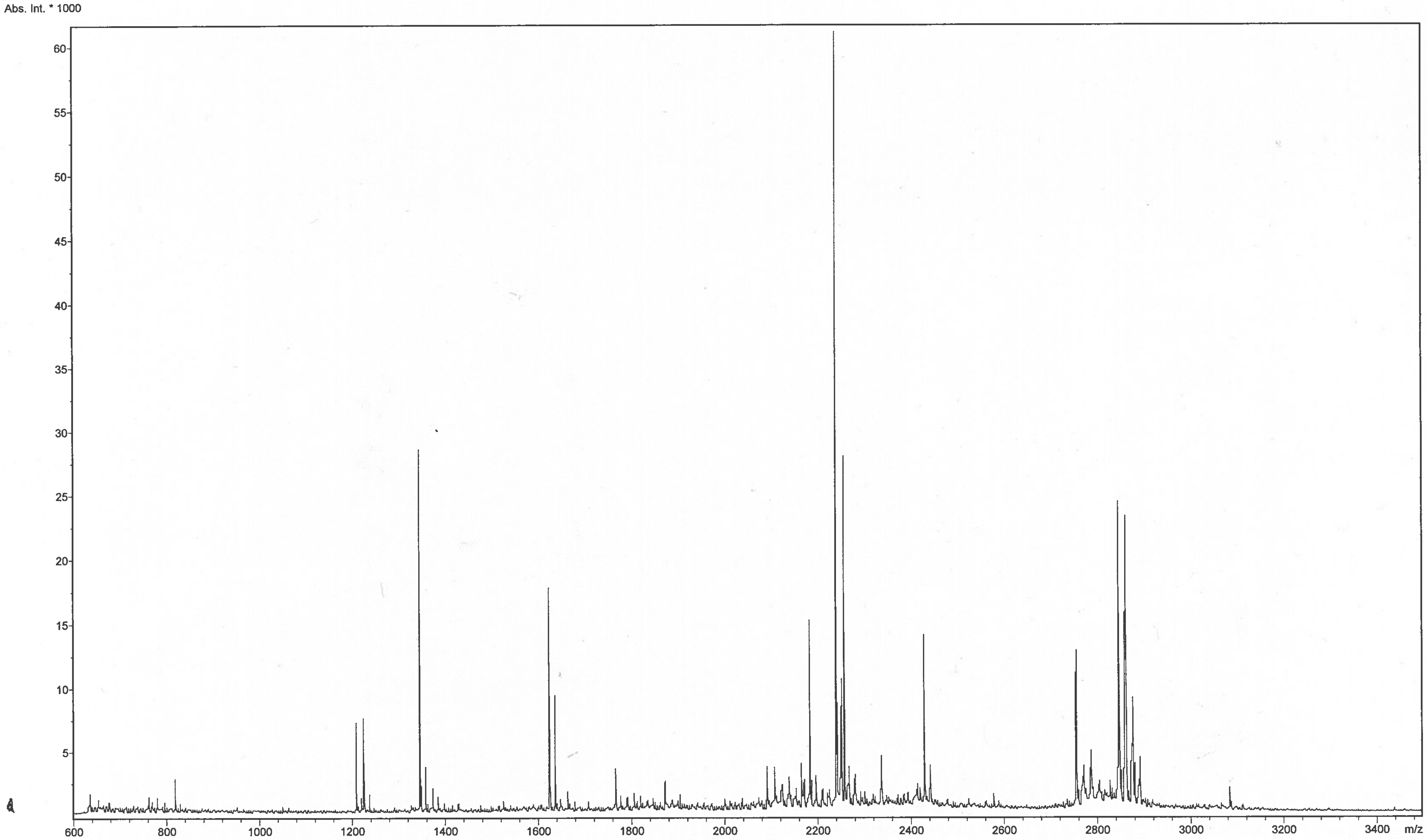

Copyright © 1999-2008 Bruker Daltonik GmbH

Spectrum Analysis Report

Date: 07/29/2011 Time: 07:39

FileName: D:\Data\Bernardo\2011_07_28\M5_34\0_K15\1\1SRef\pdata\1\PMF_LIFT.xml

Sequence data:

Intensity Coverage:	32.8 % (0 cnts)	Sequence Coverage MS:	40.5%
Sequence Coverage MS/MS:	26.3%	pI (isoelectric point):	6.8

10	20	30	40	50	60	70	80	90	100	110
MEARSKLLDG	TTASRRWTRK	LVLLLPPLLL	FLLRTESLQG	LESDERFRTR	ENECHFYAGG	QVYPGEASRV	SVADHSLHLS	KAKISKPAPY	WEGTAVINGE	FKELKLTDYR
**120**	**130**	**140**	**150**	**160**	**170**	**180**	**190**	**200**	**210**	**220**
GKYLVFFFYP	LDFTFVCPTE	IIAFGDRIEE	FKSINTEVVA	CSVDSQFTHL	AWINTPRRQG	GLGPIRIPLL	SDLNHQISKD	YGVYLEDSGH	TLRGLFIIDD	KGVLRQITLN
**230**	**240**	**250**	**260**	**270**	**280**					
DLPVGRSVDE	TLRLVQAFQY	TDKHGEVCPA	GWKPGSETII	PDPAGKLKYF	DKLN					

Acquisition Parameter:

Matched Sequences:

Unmatched

Peaks/MSMS Spectra

Tree hierarchy	Meas. M/z	Calc. MH+	Meas. Mr	Calc. Mr	Int.	z	Dev.(Da)	Dev.(ppm)	Score	MascotScore	Rt(min)	Range	P	Sequence
peak 1	763.491	-	762.484	-	1228.884	1+	-	-	-	-	-		-	
peak 2	780.452	-	779.444	-	1047.857	1+	-	-	-	-	-		-	
peak 5	1208.687	-	1207.679	-	6728.795	1+	-	-	-	-	-		-	
peak 6	1220.681	-	1219.674	-	1160.417	1+	-	-	-	-	-		-	
peak 8	1237.713	-	1236.706	-	1258.698	1+	-	-	-	-	-		-	
peak 9	1327.779	-	1326.772	-	751.752	1+	-	-	-	-	-		-	
peak 11	1357.811	-	1356.803	-	3445.862	1+	-	-	-	-	-		-	
peak 12	1361.722	-	1360.714	-	966.528	1+	-	-	-	-	-		-	
peak 13	1373.804	-	1372.796	-	1779.496	1+	-	-	-	-	-		-	
peak 14	1385.800	-	1384.793	-	1365.908	1+	-	-	-	-	-		-	
peak 15	1399.809	-	1398.802	-	831.792	1+	-	-	-	-	-		-	
peak 17	1526.918	-	1525.911	-	1042.801	1+	-	-	-	-	-		-	
peak 19	1636.798	-	1635.791	-	8369.745	1+	-	-	-	-	-		-	
peak 20	1648.803	-	1647.796	-	936.248	1+	-	-	-	-	-		-	
peak 21	1664.795	-	1663.788	-	1456.045	1+	-	-	-	-	-		-	
peak 22	1708.815	-	1707.808	-	850.360	1+	-	-	-	-	-		-	
peak 23	1766.814	-	1765.807	-	3172.074	1+	-	-	-	-	-		-	
peak 24	1777.913	-	1776.906	-	1229.558	1+	-	-	-	-	-		-	
peak 25	1791.908	-	1790.901	-	1110.368	1+	-	-	-	-	-		-	
peak 26	1806.912	-	1805.905	-	1385.400	1+	-	-	-	-	-		-	
peak 27	1819.937	-	1818.930	-	1156.335	1+	-	-	-	-	-		-	
peak 28	1833.941	-	1832.934	-	933.805	1+	-	-	-	-	-		-	
peak 29	1847.953	-	1846.945	-	956.955	1+	-	-	-	-	-		-	
peak 30	1871.978	-	1870.971	-	2193.413	1+	-	-	-	-	-		-	
peak 31	1903.976	-	1902.969	-	1225.441	1+	-	-	-	-	-		-	
peak 32	1999.994	-	1998.987	-	928.896	1+	-	-	-	-	-		-	
peak 33	2091.979	-	2090.971	-	2648.752	1+	-	-	-	-	-		-	
peak 34	2108.131	-	2107.124	-	3164.485	1+	-	-	-	-	-		-	
peak 35	2120.140	-	2119.133	-	1493.691	1+	-	-	-	-	-		-	
peak 36	2123.118	-	2122.111	-	1317.027	1+	-	-	-	-	-		-	
peak 37	2140.124	-	2139.117	-	2030.477	1+	-	-	-	-	-		-	
peak 38	2154.012	-	2153.004	-	1198.963	1+	-	-	-	-	-		-	
peak 39	2164.973	-	2163.966	-	2713.381	1+	-	-	-	-	-		-	
peak 41	2182.978	-	2181.970	-	11581.084	1+	-	-	-	-	-		-	
peak 42	2196.990	-	2195.983	-	1943.348	1+	-	-	-	-	-		-	
peak 43	2211.120	-	2210.113	-	1172.346	1+	-	-	-	-	-		-	
peak 44	2221.273	-	2220.266	-	1237.406	1+	-	-	-	-	-		-	
peak 45	2225.202	-	2224.195	-	1222.062	1+	-	-	-	-	-		-	
MSMS 46	2239.311	-	2238.303	-	43717.600	1+	-	-	-	-	-		-	
peak 47	2247.130	-	2246.123	-	1317.656	1+	-	-	-	-	-		-	
peak 48	2248.165	-	2247.157	-	1672.372	1+	-	-	-	-	-		-	
peak 49	2251.302	-	2250.295	-	7159.923	1+	-	-	-	-	-		-	
peak 51	2265.150	-	2264.142	-	1187.768	1+	-	-	-	-	-		-	
peak 52	2268.335	-	2267.327	-	2413.414	1+	-	-	-	-	-		-	

Copyright © 1999-2008 Bruker Daltonik GmbH

peak 53	2280.179	-	2279.171	-	1470.709	1+	-	-	-	-	-	-
peak 54	2292.170	-	2291.163	-	1227.908	1+	-	-	-	-	-	-
peak 55	2337.151	-	2336.144	-	2869.719	1+	-	-	-	-	-	-
peak 56	2393.375	-	2392.368	-	1122.926	1+	-	-	-	-	-	-
peak 57	2414.079	-	2413.072	-	1272.461	1+	-	-	-	-	-	-
peak 59	2441.165	-	2440.158	-	2136.028	1+	-	-	-	-	-	-
peak 60	2443.161	-	2442.154	-	1644.368	1+	-	-	-	-	-	-
peak 61	2577.383	-	2576.376	-	982.289	1+	-	-	-	-	-	-
peak 62	2754.453	-	2753.446	-	7743.561	1+	-	-	-	-	-	-
peak 63	2766.451	-	2765.443	-	1720.097	1+	-	-	-	-	-	-
peak 64	2770.459	-	2769.452	-	2160.095	1+	-	-	-	-	-	-
peak 65	2773.526	-	2772.519	-	1307.214	1+	-	-	-	-	-	-
peak 66	2776.138	-	2775.131	-	1138.587	1+	-	-	-	-	-	-
peak 67	2786.448	-	2785.441	-	3068.214	1+	-	-	-	-	-	-
peak 68	2801.434	-	2800.426	-	1096.422	1+	-	-	-	-	-	-
peak 69	2804.791	-	2803.783	-	1023.404	1+	-	-	-	-	-	-
peak 70	2809.139	-	2808.132	-	1075.391	1+	-	-	-	-	-	-
peak 71	2827.458	-	2826.450	-	1453.051	1+	-	-	-	-	-	-
peak 73	2856.463	-	2855.456	-	2132.434	1+	-	-	-	-	-	-
peak 74	2859.484	-	2858.477	-	12737.116	1+	-	-	-	-	-	-
peak 75	2862.480	-	2861.473	-	3713.928	1+	-	-	-	-	-	-
peak 76	2870.469	-	2869.462	-	1744.161	1+	-	-	-	-	-	-
peak 77	2873.487	-	2872.480	-	2803.139	1+	-	-	-	-	-	-
peak 78	2876.476	-	2875.468	-	5375.616	1+	-	-	-	-	-	-
peak 79	2879.458	-	2878.451	-	1795.005	1+	-	-	-	-	-	-
peak 80	2887.480	-	2886.473	-	1548.696	1+	-	-	-	-	-	-
peak 81	2891.480	-	2890.473	-	2926.876	1+	-	-	-	-	-	-

Global peptide results

Peroxiredoxin-4 OS=Mus musculus GN=Prdx4 PE=1 SV=1 PRDX4_MOUSE
MW:31261.230

MEARSKLLDGTTASRRWTRKLVLLLPPLLLFLLRTESLQGLESDERFRTRENECHFYAGGQVYPGEASRVSVADHSLHLSKAKISKPAPYWEGTAVINGEFKELKLTDYRGKYLVFFFYPLDFTFVCPTEIIAFGDRIEEFKSINTEVVACSVDSQFTHLAWINTPRRQGGLGPIRIPLLSDLNHQISKDYGVYLEDSGHTLRGLFIIDDK
GVLRQITLNDLPVGRSVDETLRLVQAFQYTDKHGEVCPAGWKPGSETIIPDPAGKLKYFDKLN

Digest Matches (Score: 254.00)
Score = 254.000000, Rank = 1, Database = SwissProt, Accesskey = PRDX4_MOUSE
Search Parameters: MS Tol.:100.00 ppm, MSMS Tol.: 0.600000 Da, Enz.Trypsin, Engine: Mascot Version: 2.3.01.241 , DB: NCBInr NCBInr , DB Version: NCBInr_20110715.fasta NCBInr_20110715.fasta
Modifications: Optional: Oxidation (M)

Tree hierarchy	Meas. M/z	Calc. MH+	Meas. Mr	Calc. Mr	Int.	z	Dev.(Da)	Dev.(ppm)	Score	MascotScore	Rt(min)	Range	P	Sequence
peak 3	797.471	797.463	796.464	796.456	840.212	1+	0.008	10.514	-	-	-	169 - 176	0	QGGLGPIR
peak 4	819.446	819.421	818.438	818.413	2593.104	1+	0.025	30.416	-	-	-	227 - 233	0	SVDETLR
peak 7	1225.714	1225.690	1224.706	1224.683	7257.028	1+	0.024	19.451	-	-	-	216 - 226	0	QITLNDLPVGR
MSMS 10	1345.816	1345.784	1344.808	1344.777	28002.945	1+	0.032	23.724	90	49	-	204 - 215	1	GLFIIDDKGVLR
peak 16	1477.880	1477.837	1476.873	1476.830	692.375	1+	0.043	28.869	-	-	-	177 - 189	0	IPLLSDLNHQISK
MSMS 18	1624.800	1624.760	1623.792	1623.753	16268.732	1+	0.039	24.235	150	54	-	190 - 203	0	DYGVYLEDSGHTLR
peak 40	2170.996	2170.925	2169.988	2169.917	1590.407	1+	0.071	32.715	-	-	-	51 - 69	0	ENECHFYAGGQVYPGEASR 4: Carbamidomethyl (C)
MSMS 50	2256.339	2256.282	2255.331	2255.275	19818.762	1+	0.056	24.974	164	30	-	169 - 189	1	QGGLGPIRIPLLSDLNHQISK
peak 58	2428.140	2428.073	2427.133	2427.066	8970.903	1+	0.067	27.465	-	-	-	49 - 69	1	TRENECHFYAGGQVYPGEASR 6: Carbamidomethyl (C)
MSMS 72	2845.470	2845.394	2844.463	2844.386	13407.881	1+	0.076	26.850	107	35	-	143 - 167	0	SINTEVVACSVDSQFTHLAWINTPR 9: Carbamidomethyl (C)
peak 82	3083.657	3083.580	3082.650	3082.572	1100.439	1+	0.077	25.064	-	-	-	177 - 203	1	IPLLSDLNHQISKDYGVYLEDSGHTLR

Copyright © 1999-2008 Bruker Daltonik GmbH
