# Supplementary material for: Proteomic Analysis of the Action of the Mycobacterium ulcerans Toxin Mycolactone: Targeting Host Cells Cytoskeleton and Collagen
Source: PLoS Negl Trop Dis. 2014 Aug 7;8(8):e3066. doi: 10.1371/journal.pntd.0003066 (PMC4125307; doi:10.1371/journal.pntd.0003066)
Supplement: Dataset S7 — MS and MS/MS data. (ZIP) [file pntd.0003066.s010.zip › MS Data/Spot 10 - Ubiquitin.pdf]

D:\Data\Bernardo\2011_07_26\M23_03\0_I13\1\1SRef

Comment 1

Comment 2

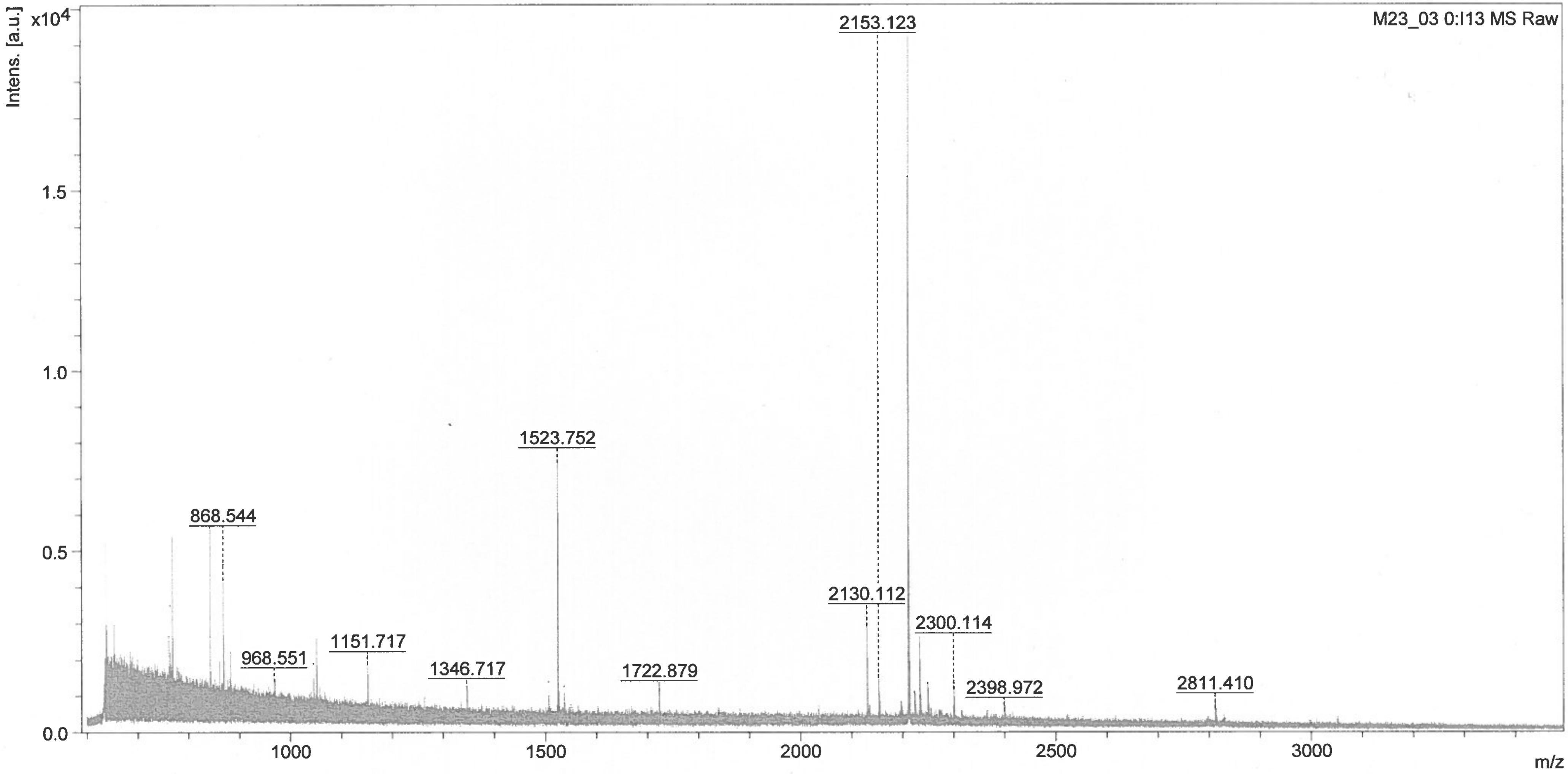

Spectrum Analysis Report

Date: 07/29/2011 Time: 06:47

FileName: D:\Data\Bernardo\2011_07_26\M23_03\0_I13\1\1SRef\pdata\1\PMF_LIFT.xml

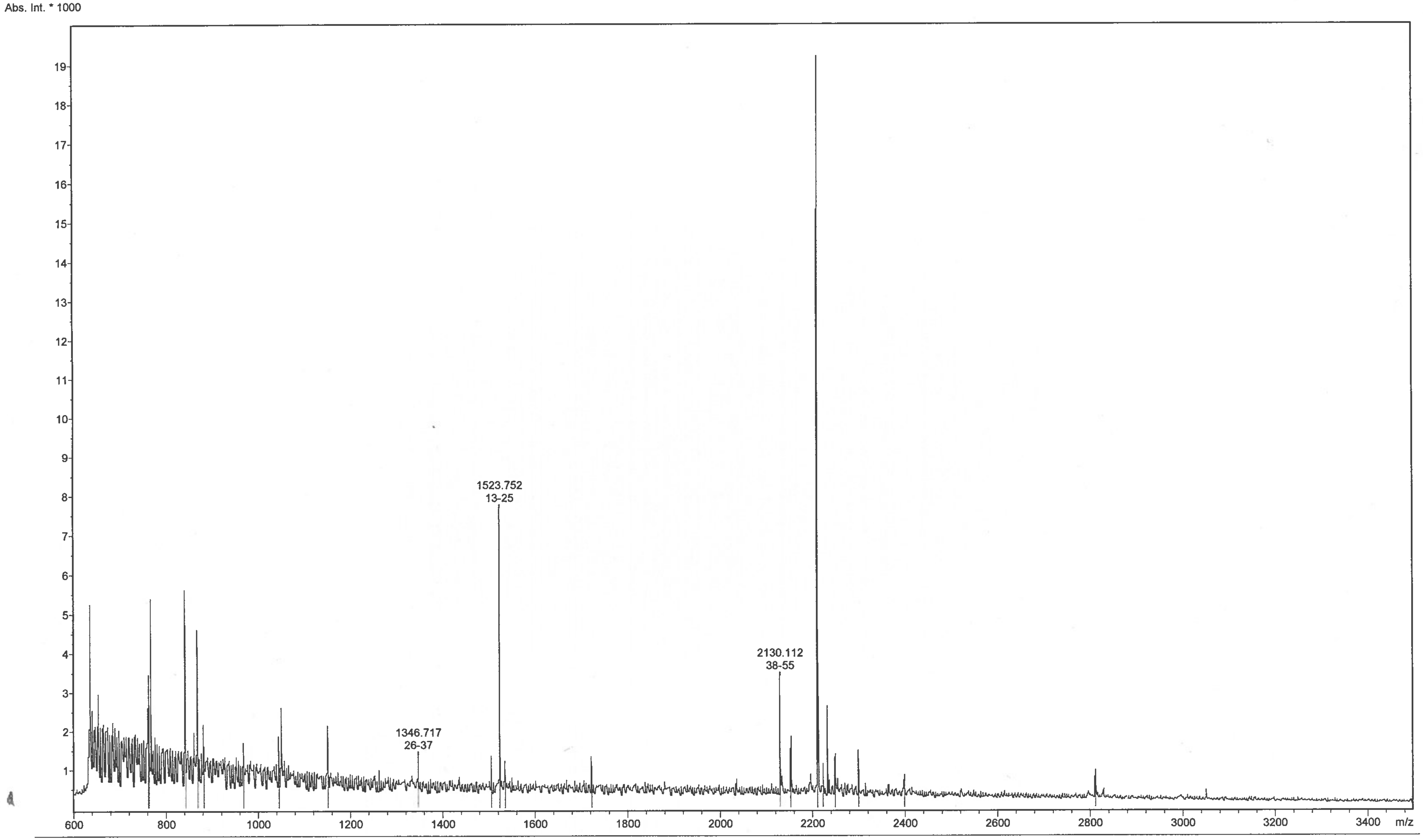

Copyright © 1999-2008 Bruker Daltonik GmbH

Spectrum Analysis Report

Date: 07/29/2011 Time: 06:47

FileName: D:\Data\Bernardo\2011_07_26\M23_03\0_I13\1\1SRef\pdata\1\PMF_LIFT.xml

Sequence data:

ubiquitin A-52 residue ribosomal protein fusion product 1 [Mus musculus] gi|154550710

Intensity Coverage:	21.1 % (11494 cnts)	Sequence Coverage MS:	60.6%
Sequence Coverage MS/MS:	18.3%	pI (isoelectric point):	7.7

10	20	30	40	50	60	70	80
EPSDTIENVK	AKIQDKEGIP	PDQQRLIFAG	KQLEDGRTLS	DYNIQKESTL	HLVLRLRGGI	IEPSLRQLAQ	K

Acquisition Parameter:

Matched Sequences:

Unmatched

Peaks/MSMS Spectra

Tree hierarchy	Meas. M/z	Calc. MH+	Meas. Mr	Calc. Mr	Int.	z	Dev.(Da)	Dev.(ppm)	Score	MascotScore	Rt(min)	Range	P	Sequence
peak 2	763.456	-	762.449	-	2690.754	1+	-	-	-	-	-		-	
peak 3	842.491	-	841.484	-	4728.937	1+	-	-	-	-	-		-	
peak 5	882.546	-	881.539	-	1604.507	1+	-	-	-	-	-		-	
peak 6	968.551	-	967.544	-	1381.159	1+	-	-	-	-	-		-	
peak 7	1045.539	-	1044.532	-	1497.914	1+	-	-	-	-	-		-	
peak 8	1151.717	-	1150.709	-	1855.293	1+	-	-	-	-	-		-	
peak 10	1505.741	-	1504.733	-	1036.041	1+	-	-	-	-	-		-	
peak 13	1722.879	-	1721.872	-	1103.475	1+	-	-	-	-	-		-	
peak 15	2153.123	-	2152.116	-	1483.880	1+	-	-	-	-	-		-	
peak 16	2211.059	-	2210.052	-	14216.674	1+	-	-	-	-	-		-	
peak 17	2223.053	-	2222.045	-	986.755	1+	-	-	-	-	-		-	
peak 18	2249.013	-	2248.005	-	1125.692	1+	-	-	-	-	-		-	

Global peptide results

ubiquitin A-52 residue ribosomal protein fusion product 1 [Mus musculus] gi|154550710
MW:8033.350

EPSDTIENVKAKIQDKEGIPPDQQRLIFAGKQLEDGRTLSDYNIQKESTLHLVLRLRGGIIEPSLRQLAQK

Digest Matches (Score: 90.40)
Score = 90.400000, Rank = 1, Database = NCBInr, Accesskey = gi|154550710
Search Parameters: MS Tol.:100.00 ppm, MSMS Tol.: 0.600000 Da, Enz.Trypsin, Engine: Mascot Version: 2.3.01.241 , DB: NCBInr NCBInr , DB Version: NCBInr_20110715.fasta NCBInr_20110715.fasta
Modifications: Optional: Oxidation (M)

Tree hierarchy	Meas. M/z	Calc. MH+	Meas. Mr	Calc. Mr	Int.	z	Dev.(Da)	Dev.(ppm)	Score	MascotScore	Rt(min)	Range	P	Sequence
peak 9	1346.717	1346.743	1345.710	1345.735	1174.147	1+	-0.026	-19.057	-	-	-	26 - 37	1	LIFAGKQLEDGR
MSMS 11	1523.752	1523.781	1522.745	1522.774	7407.782	1+	-0.029	-18.872	18	45	-	13 - 25	1	IQDKEGIPPDQQR
peak 14	2130.112	2130.155	2129.104	2129.148	2912.258	1+	-0.044	-20.451	-	-	-	38 - 55	1	TLSDYNIQKESTLHLVLR

ataxin-7 [Mus musculus] gi|159032018
MW:93836.420

MSERAADDVRGEPRRAAGGAAAARQQQQPQPLQPQRQHPPLRRPRAEDGGTGDTTTSAAAMATVGERRPLPSPEAMLGQSWNLWVEASKLPGKDGTELDESFKEFGKNREVMGLCREDMPIFGLCPAHDDFYLVVCNDCNQVVKPQAFQSHYERRHSSSSKPALAVPHTSVFSLLPSLSKSKGSGAGGSSRPPSGGVLCASSSSKLLRLP
KEKLPLRGNMKPMHPVQQIKVPHGRVMTPSVKVEKMHPKMDGTLLKSTVGPACPATMSSAVKPGLNCPSIPKPTLPSPGQILNGKGLPAMPTLEKKSEDSSNNRKFLNKRLSEREFDPDIHCGVIDLDTKKPCTRSLTCKTHSLTQRRAVQGRRKRFDVLLAEHKNKAREKELIRHDSQQVPHPLRDPHPTPPRTPQEPQLPAESKPFLAS
KPKPQTPSLPRPPGCPAQQGGSTPIDPPPGQESPHPPLPATEPASRLSSEEGEGDDREESVEKLDCHYSGRHPQPASFCTFGSRQIGRGYYVFDSRWNRLRCALNLMVEKHLNAQLWKKIPPVPCTTSPVSARVPHRTNSVPTSQGGISYLAATTVSAPPVLLSSTCISPNSKSVPAHGTTLNAQPAGSGAMDPVCSVQSRQVSASSSPPS
TPSGLSSVPSSPLSRKPQKWKPSKSIRPKESSALSTNCHNASSSTSGGSGKKRKNSSPLLVPSSSSSSSSSSSSHSVNSFRKNCVAHSGTPYLSTAPSSHSIGLNCVTNKTHSVSLRHEQAGRGPAGVSSAEPIKRMSVMVNSSDSTLSLGPFIHQASELPVNPHSHTPLDKLIGKKRKCSPGSSTVGNSGSKPTKVAKLPAMNNVHMKH
TGNISGAQGLTNNSLLHQPKARP

Digest Matches (Score: 71.00)
Score = 71.000000, Rank = 1, Database = NCBInr, Accesskey = gi|159032018
Search Parameters: MS Tol.:100.00 ppm, MSMS Tol.: 0.600000 Da, Enz.Trypsin, Engine: Mascot Version: 2.3.01.241 , DB: NCBInr , DB Version: NCBInr_20110715.fasta
Modifications: Optional: Oxidation (M)

Tree hierarchy	Meas. M/z	Calc. MH+	Meas. Mr	Calc. Mr	Int.	z	Dev.(Da)	Dev.(ppm)	Score	MascotScore	Rt(min)	Range	P	Sequence
peak 1	761.439	761.423	760.432	760.415	1791.507	1+	0.016	21.494	-	-	-	237 - 243	0	VMTPSVK
peak 4	868.544	868.471	867.537	867.464	4176.663	1+	0.073	84.206	-	-	-	244 - 250	1	VEKMHPK
peak 9	1346.717	1346.728	1345.710	1345.721	1174.147	1+	-0.011	-8.354	-	-	-	522 - 532	1	LRCALNLMVEK 3: Carbamidomethyl (C)
MSMS 11	1523.752	1523.782	1522.745	1522.775	7407.782	1+	-0.030	-19.443	1	-	-	219 - 231	0	GNMKPMHPVQQIK 3: Oxidation (M)
peak 12	1535.741	1535.759	1534.733	1534.751	950.078	1+	-0.018	-11.874	-	-	-	91 - 104	1	LPGKDGTELDESFK
peak 19	2300.114	2300.197	2299.107	2299.189	1166.076	1+	-0.082	-35.794	-	-	-	16 - 37	1	AAGGAAAARQQQQPQPLQPQR
peak 20	2398.972	2399.205	2397.965	2398.198	548.933	1+	-0.233	-97.054	-	-	-	624 - 648	0	QVSASSSPPSTPSGLSSVPSSPLSR
peak 21	2811.410	2811.315	2810.403	2810.308	624.977	1+	0.095	33.951	-	-	-	596 - 623	0	SVPAHGTTLNAQPAGSGAMDPVCSVQSR 19: Oxidation (M) 23: Carbamidomethyl (C)

Copyright © 1999-2008 Bruker Daltonik GmbH
