# Supplementary material for: Proteomic Analysis of the Action of the Mycobacterium ulcerans Toxin Mycolactone: Targeting Host Cells Cytoskeleton and Collagen
Source: PLoS Negl Trop Dis. 2014 Aug 7;8(8):e3066. doi: 10.1371/journal.pntd.0003066 (PMC4125307; doi:10.1371/journal.pntd.0003066)
Supplement: Dataset S7 — MS and MS/MS data. (ZIP) [file pntd.0003066.s010.zip › MS Data/Spot 11 - BSA.pdf]

D:\Data\Bernardo\2011_07_30\P23_37\0_P14\1\1SRef

Comment 1

Comment 2

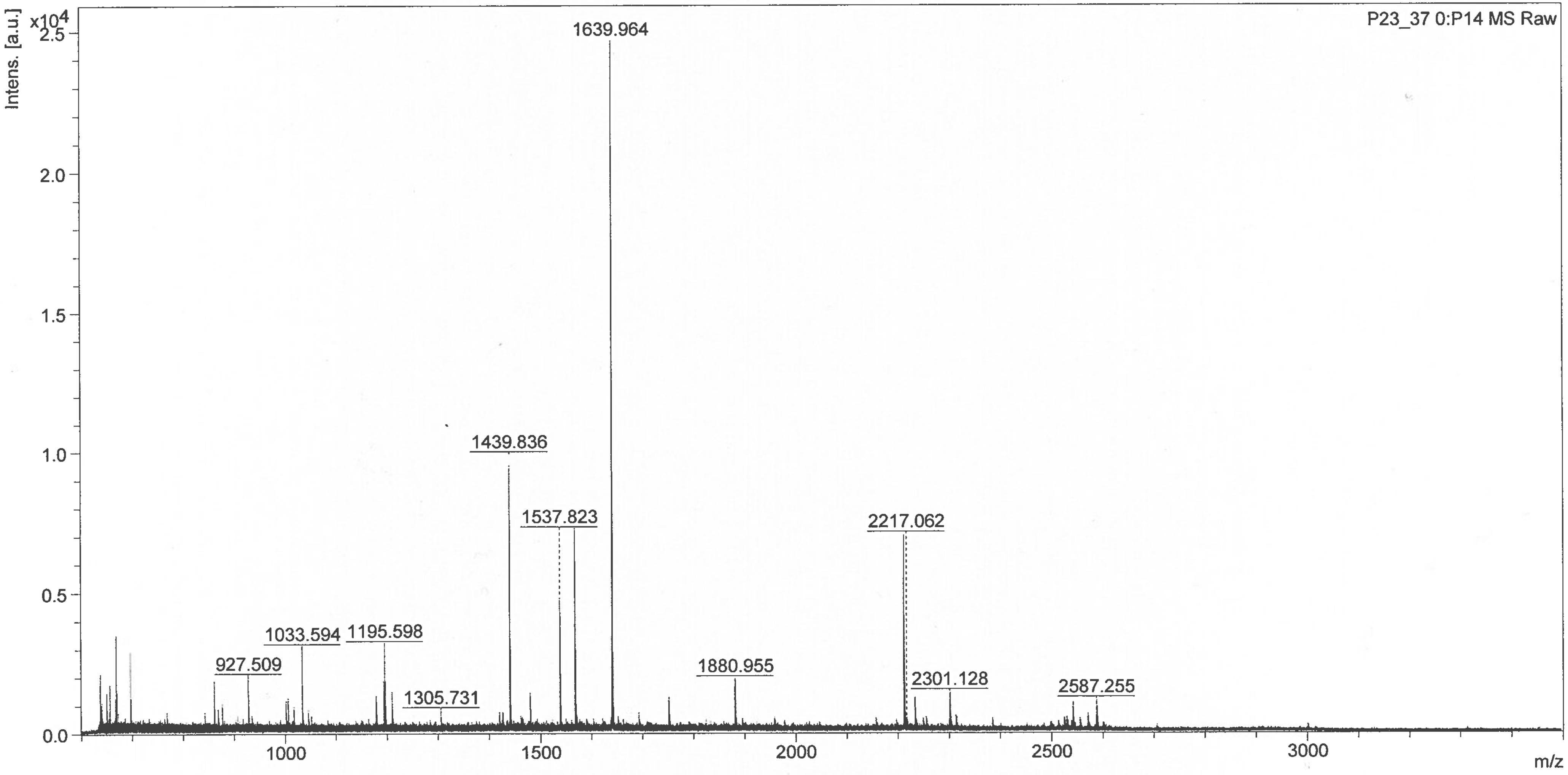

Bruker Daltonics flexAnalysis printed: 7/30/2011 1:59:55 PM

Spectrum Analysis Report

Date: 07/30/2011 Time: 13:59

FileName: D:\Data\Bernardo\2011_07_30\P23_37\0_P14\1\1SRef\pdata\1\PMF_LIFT.xml

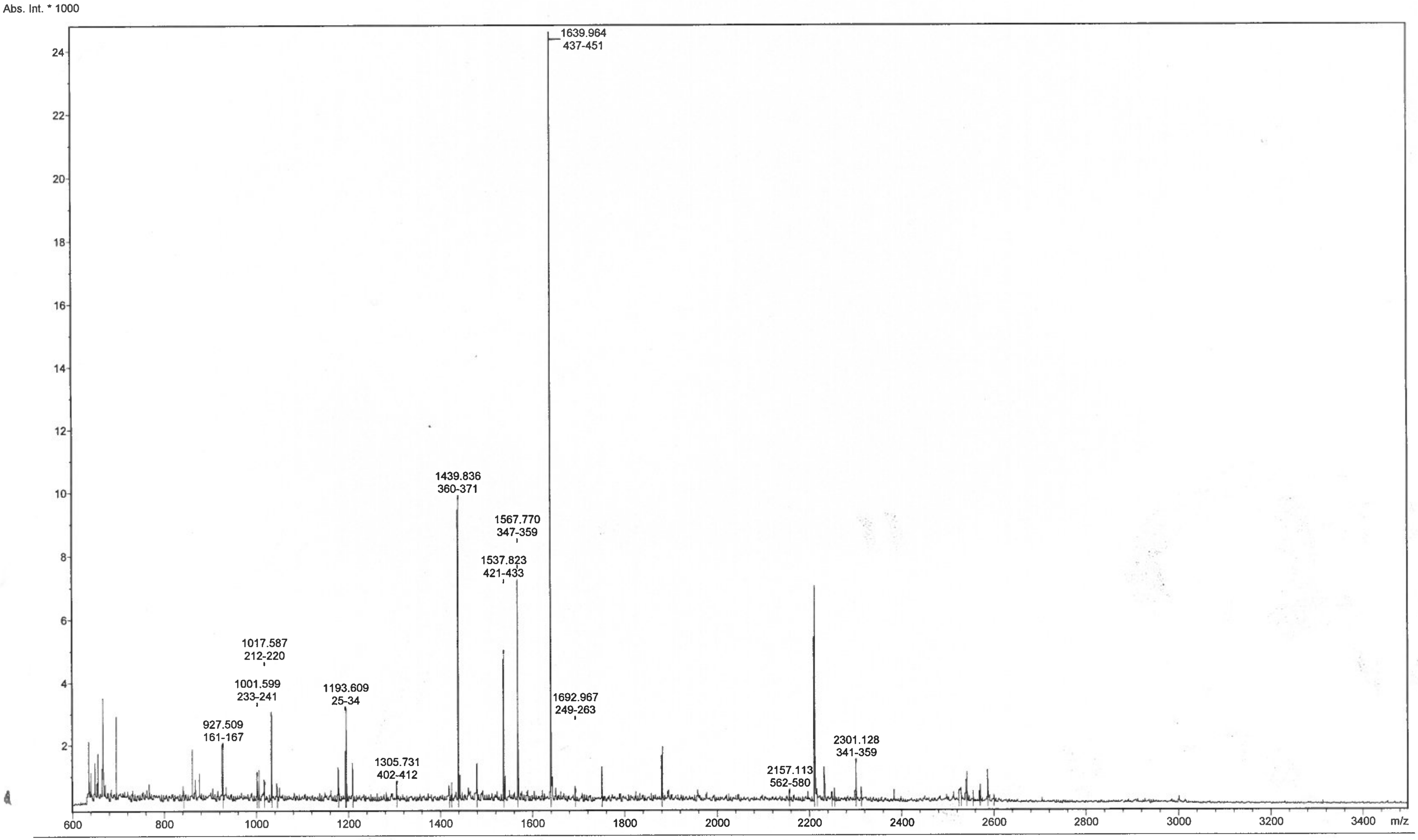

Copyright © 1999-2008 Bruker Daltonik GmbH

Spectrum Analysis Report

Date: 07/30/2011 Time: 13:59

FileName: D:\Data\Bernardo\2011_07_30\P23_37\0_P14\1\1SRef\pdata\1\PMF_LIFT.xml

Sequence data:

ALB protein [Bos taurus] gi|74267962

Intensity Coverage:	68.5 % (55016 cnts)	Sequence Coverage MS:	22.9%
Sequence Coverage MS/MS:	8.7%	pI (isoelectric point):	5.9

10	20	30	40	50	60	70	80	90	100	110
MKWVTFISLL	LLFSSAYSRG	VFRRDTHKSE	IAHRFKDLGE	EHFKGLVLIA	FSQYLQQCPF	DEHVKLVNEL	TEFAKTCVAD	ESHAGCEKSL	HTLFGDELCK	VASLRETYGD
120	130	140	150	160	170	180	190	200	210	220
MADCCAKQEP	ERNECFLSHK	DDSPDLPKLK	PDPNTLCDEF	KADEKKFWGK	YLYEIARRHP	YFYAPELLYY	ANKYNGVFQE	CCQAEDKGAC	LLPKIETMRE	KVLTSSARQR
230	240	250	260	270	280	290	300	310	320	330
LRCASIQKFG	ERALKAWSVA	RLSQKFPKAE	FVEVTKLVTD	LTKVHKECCH	GDLLECADDR	ADLAKYICDN	QDTISSKLKE	CCDKPLLEKS	HCIAEVEKDA	IPENLPPLTA
340	350	360	370	380	390	400	410	420	430	440
DFAEDKDVCK	NYQEAKDAFL	GSFLYEYSRR	HPEYAVSVLL	RLAKEYEATL	EECCAKDDPH	ACYSTVFDKL	KHLVDEPQNL	IKQNCDQFEK	LGEYGFQNEL	IVRYTRKVPQ
450	460	470	480	490	500	510	520	530	540	550
VSTPTLVEVS	RSLGKVGTRC	CTKPESERMP	CAEDYLSLIL	NRLCVLHEKT	PVSEKVTKCC	TESLVNRRPC	FSALTPDETY	VPKAFDEKLF	TFHADICTLP	DTEKQIKKQT
560	570	580	590	600	610					
ALVELLKHKP	KATEEQLKTV	MENFVAFVGK	CCAADDKEAC	FAVEGPKLVV	STQTALA					

Acquisition Parameter:

Matched Sequences:

Unmatched

Peaks/MSMS Spectra

Tree hierarchy	Meas. M/z	Calc. MH+	Meas. Mr	Calc. Mr	Int.	z	Dev.(Da)	Dev.(ppm)	Score	MascotScore	Rt(min)	Range	P	Sequence
peak 1	842.524	-	841.516	-	547.030	1+	-	-	-	-	-		-	
peak 4	1005.600	-	1004.593	-	1106.270	1+	-	-	-	-	-		-	
peak 6	1033.594	-	1032.586	-	2953.895	1+	-	-	-	-	-		-	
peak 7	1045.579	-	1044.572	-	664.681	1+	-	-	-	-	-		-	
peak 8	1178.574	-	1177.567	-	1194.245	1+	-	-	-	-	-		-	
peak 10	1195.598	-	1194.591	-	2469.208	1+	-	-	-	-	-		-	
peak 11	1209.619	-	1208.612	-	1216.349	1+	-	-	-	-	-		-	
peak 13	1419.712	-	1418.705	-	564.685	1+	-	-	-	-	-		-	
peak 14	1424.768	-	1423.761	-	477.332	1+	-	-	-	-	-		-	
peak 16	1479.811	-	1478.804	-	1403.200	1+	-	-	-	-	-		-	
peak 21	1750.999	-	1749.991	-	1096.119	1+	-	-	-	-	-		-	
peak 22	1880.955	-	1879.948	-	1614.274	1+	-	-	-	-	-		-	
peak 24	2211.142	-	2210.135	-	5375.731	1+	-	-	-	-	-		-	
peak 25	2217.062	-	2216.054	-	377.287	1+	-	-	-	-	-		-	
peak 26	2249.089	-	2248.082	-	348.126	1+	-	-	-	-	-		-	
peak 27	2255.104	-	2254.097	-	356.866	1+	-	-	-	-	-		-	
peak 29	2313.129	-	2312.122	-	471.528	1+	-	-	-	-	-		-	
peak 30	2524.182	-	2523.174	-	324.761	1+	-	-	-	-	-		-	
peak 31	2529.240	-	2528.232	-	280.730	1+	-	-	-	-	-		-	
peak 32	2541.199	-	2540.192	-	614.024	1+	-	-	-	-	-		-	
peak 33	2555.211	-	2554.204	-	284.519	1+	-	-	-	-	-		-	
peak 34	2570.230	-	2569.223	-	473.687	1+	-	-	-	-	-		-	
peak 35	2587.255	-	2586.247	-	760.618	1+	-	-	-	-	-		-	
peak 36	2600.269	-	2599.262	-	266.100	1+	-	-	-	-	-		-	

Global peptide results

ALB protein [Bos taurus] gi|74267962
MW:69190.430

MKWVTFISLLLLFSSAYSRGVFRRDTHKSEIAHRFKDLGEEHFKGLVLIAFSQYLQQCPFDEHVKLVNELTEFAKTCVADESHAGCEKSLHTLFGDELCKVASLRETYGDMADCCAKQEPERNECFLSHKDDSPDLPKLKPDPNTLCDEFKADEKKFWGKYLYEIARRHPYFYAPELLYYANKYNGVFQECCQAEDKGACLLPKIETMREK
VLTSSARQRLRCASIQKFGERALKAWSVARLSQKFPKAEFVEVTKLVTDLTKVHKECCHGDLLECADDRADLAKYICDNQDTISSKLKECCDKPLLEKSHCIAEVEKDAIPENLPPLTADFAEDKDVCKNYQEAKDAFLGSFLYEYSRRHPEYAVSVLLRLAKEYEATLEECCAKDDPHACYSTVFDKLKHLVDEPQNLIKQNCDQFEKLG
EYGFQNELIVRYTRKVPQVSTPTLVEVSRSLGKVGTRCCTKPESERMPCAEDYLSLILNRLCVLHEKTPVSEKVTKCCTESLVNRRPCFSALTPDETYVPKAFDEKLFTFHADICTLPDTEKQIKKQTALVELLKHKPKATEEQLKTVMENFVAFVGKCCAADDKEACFAVEGPKLVVSTQTALA

Digest Matches (Score: 235.00)
Score = 235.000000, Rank = 1, Database = NCBInr, Accesskey = gi|74267962
Search Parameters: MS Tol.:100.00 ppm, MSMS Tol.:0.600000Da, Enz.Trypsin, Engine:Mascot Version:2.3.01.241, DB:NCBInr NCBInr, DB Version:NCBInr_20110715.fasta NCBInr_20110715.fasta

Tree hierarchy	Meas. M/z	Calc. MH+	Meas. Mr	Calc. Mr	Int.	z	Dev.(Da)	Dev.(ppm)	Score	MascotScore	Rt(min)	Range	P	Sequence

Copyright © 1999-2008 Bruker Daltonik GmbH

peak 2	927.509	927.493	926.502	926.486	1973.275	1+	0.016	16.753	-	-	-	161 - 167	0	YLYEIAR
peak 3	1001.599	1001.589	1000.592	1000.582	1021.466	1+	0.010	9.911	-	-	-	233 - 241	1	ALKAWSVAR
peak 5	1017.587	1017.580	1016.580	1016.573	721.203	1+	0.007	6.842	-	-	-	212 - 220	1	VLTSSARQR
peak 9	1193.609	1193.602	1192.601	1192.595	1639.532	1+	0.007	5.459	-	-	-	25 - 34	1	DTHKSEIAHR
peak 12	1305.731	1305.716	1304.724	1304.709	658.775	1+	0.015	11.264	-	-	-	402 - 412	0	HLVDEPQNLIK
MSMS 15	1439.836	1439.812	1438.828	1438.804	9910.840	1+	0.024	16.588	59	22	-	360 - 371	1	RHPEYAVSVLLR
MSMS 17	1537.823	1537.801	1536.816	1536.794	4912.140	1+	0.022	14.235	30	11	-	421 - 433	0	LGEYGFQNELIVR
MSMS 18	1567.770	1567.743	1566.763	1566.735	7600.496	1+	0.028	17.575	54	30	-	347 - 359	0	DAFLGSFLYEYSR
MSMS 19	1639.964	1639.938	1638.956	1638.930	24703.884	1+	0.026	15.768	117	79	-	437 - 451	1	KVPQVSTPTLVEVSR
peak 20	1692.967	1692.942	1691.960	1691.935	468.923	1+	0.025	14.977	-	-	-	249 - 263	1	AEFVEVTKLVTDLTK
peak 23	2157.113	2157.090	2156.105	2156.082	361.218	1+	0.023	10.661	-	-	-	562 - 580	1	ATEEQLKTVMENFVAFVGK 10: Oxidation (M)
peak 28	2301.128	2301.082	2300.121	2300.075	1043.809	1+	0.046	19.949	-	-	-	341 - 359	1	NYQEAKDAFLGSFLYEYSR

Copyright © 1999-2008 Bruker Daltonik GmbH
