# Supplementary material for: Proteomic Analysis of the Action of the Mycobacterium ulcerans Toxin Mycolactone: Targeting Host Cells Cytoskeleton and Collagen
Source: PLoS Negl Trop Dis. 2014 Aug 7;8(8):e3066. doi: 10.1371/journal.pntd.0003066 (PMC4125307; doi:10.1371/journal.pntd.0003066)
Supplement: Dataset S7 — MS and MS/MS data. (ZIP) [file pntd.0003066.s010.zip › MS Data/Spot 12 - P4ha1.pdf]

D:\Data\Bernardo\2011_07_30\P5_18\0_O8\1\1SRef

Comment 1
Comment 2

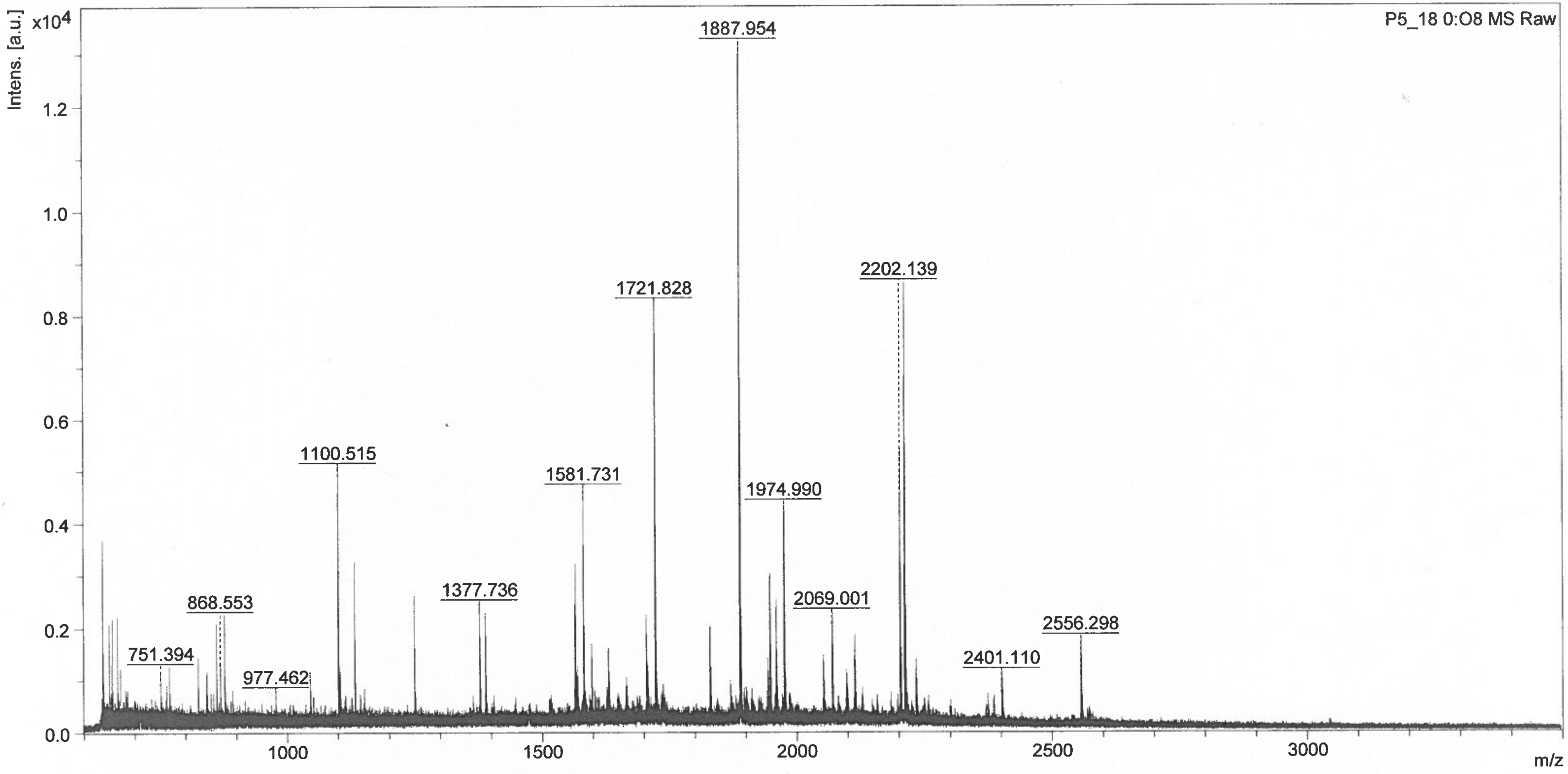

Bruker Daltonics flexAnalysis   printed: 7/30/2011 12:59:52 PM

Spectrum Analysis Report

Date: 07/30/2011 Time: 12:59

FileName: D:\Data\Bernardo\2011_07_30\P5_18\0_O8\1\1SRef\pdata\1\PMF_LIFT.xml

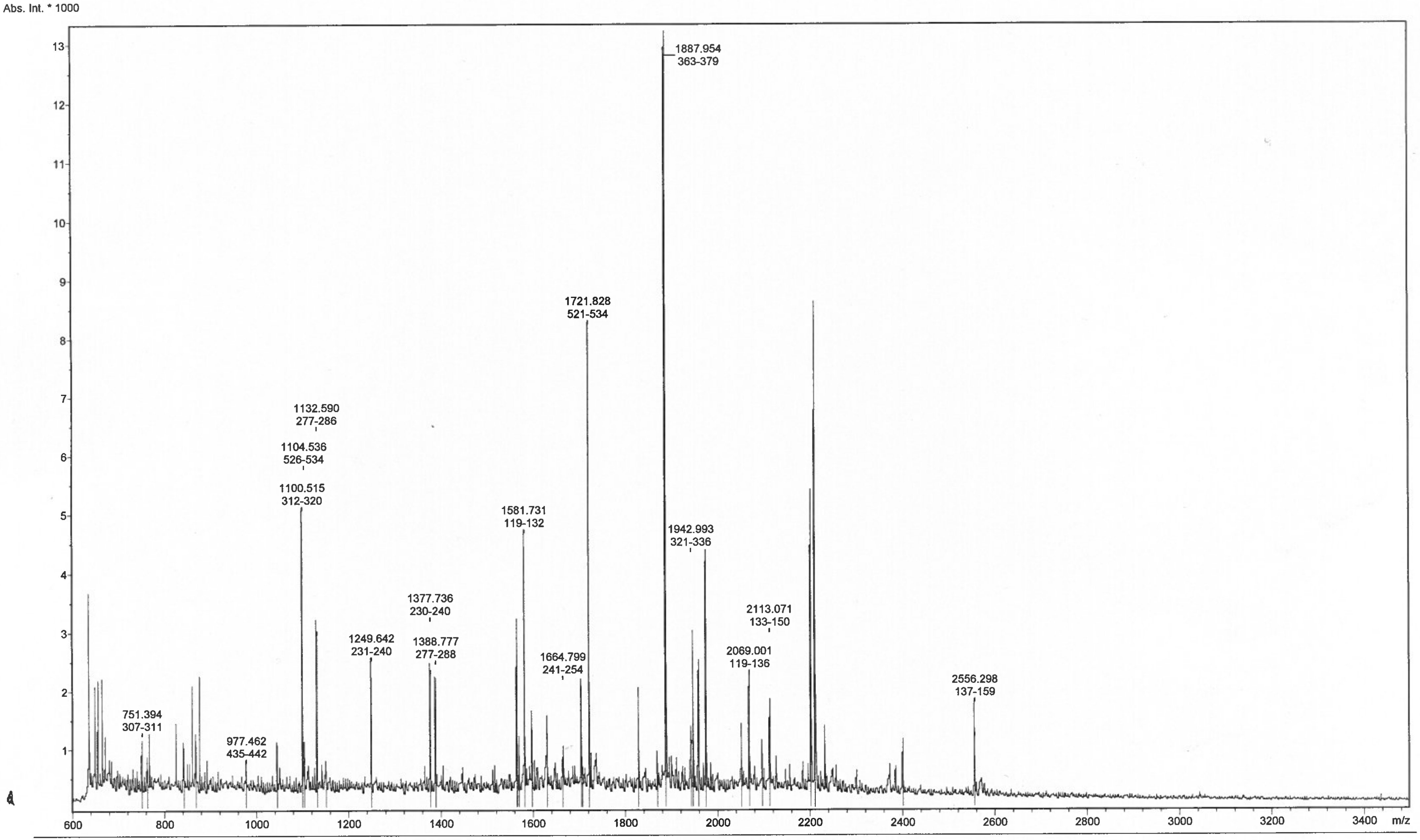

Copyright © 1999-2008 Bruker Daltonik GmbH

Spectrum Analysis Report

Date: 07/30/2011 Time: 12:59

FileName: D:\Data\Bernardo\2011_07_30\P5_18\0_O8\1\1SRef\pdata\1\PMF_LIFT.xml

Sequence data:

unnamed protein product [Mus musculus] gi|26336999

Intensity Coverage:	55.4 % (48728 cnts)	Sequence Coverage MS:	27.5%
Sequence Coverage MS/MS:	10.9%	pI (isoelectric point):	5.5

10	20	30	40	50	60	70	80	90	100	110
MIWVVLMMAI	LLPQSLAHPG	FFTSIGQMTD	LIHNEKDLVT	SLKDYIKAEE	DKLEQIKKWA	EKLDRLTSTA	TKDPEGFVGH	PVNAFKLMKR	LNTEWSELEN	LILKDMSDGF
120	130	140	150	160	170	180	190	200	210	220
ISNLTIQRQY	FPNDEDQVGA	AKALFRLQDT	YNLDTNTISK	GNLPGVQHKS	FLTAEDCFEL	GKVAYTEADY	YHTELWMEQA	LTQLEEGELS	TVDKVSVLDY	LSYAVYQQGD
230	240	250	260	270	280	290	300	310	320	330
LDKALLLTKK	LLELDPEHQR	ANGNLVYFEY	IMSKEKDANK	SASGDQSDQK	TAPKKKGIAV	DYLPERQKYE	MLCRGEGIKM	TPRRQKRLFC	RYHDGNRNPK	FILAPAKQED
340	350	360	370	380	390	400	410	420	430	440
EWDKPRIIRF	HDIISDAEIE	IVKDLAKPRL	SRATVHDPET	GKLTTAQYRV	SKSAWLSGYE	DPVVSRINMR	IQDLTGLDVS	TAEELQVANY	GVGGQYEPHF	DFARKDEPDA
450	460	470	480	490	500	510	520	530	540	
FRELGTGNRI	ATWLFYMSDV	SAGGATVFPE	VGASVWPKKG	TAVFWYNLFA	SGEGDYSTRH	AACPVLVGNK	WVSNKWLHER	GQEFRRPCTL	SELE	

Acquisition Parameter:

Matched Sequences:

Unmatched

Peaks/MSMS Spectra

Tree hierarchy	Meas. M/z	Calc. MH+	Meas. Mr	Calc. Mr	Int.	z	Dev.(Da)	Dev.(ppm)	Score	MascotScore	Rt(min)	Range	P	Sequence
peak 2	763.460	-	762.453	-	653.955	1+	-	-	-	-	-		-	
peak 3	842.503	-	841.496	-	1027.050	1+	-	-	-	-	-		-	
peak 4	868.553	-	867.546	-	1066.258	1+	-	-	-	-	-		-	
peak 6	1045.559	-	1044.552	-	1076.909	1+	-	-	-	-	-		-	
peak 10	1151.729	-	1150.721	-	624.772	1+	-	-	-	-	-		-	
peak 14	1564.705	-	1563.697	-	2537.650	1+	-	-	-	-	-		-	
peak 15	1566.773	-	1565.766	-	896.395	1+	-	-	-	-	-		-	
peak 16	1569.739	-	1568.732	-	876.337	1+	-	-	-	-	-		-	
peak 18	1597.740	-	1596.732	-	1324.020	1+	-	-	-	-	-		-	
peak 19	1630.809	-	1629.802	-	1271.043	1+	-	-	-	-	-		-	
peak 21	1704.827	-	1703.820	-	2083.102	1+	-	-	-	-	-		-	
peak 22	1707.761	-	1706.754	-	689.193	1+	-	-	-	-	-		-	
peak 24	1828.941	-	1827.934	-	1900.547	1+	-	-	-	-	-		-	
peak 25	1869.925	-	1868.917	-	692.789	1+	-	-	-	-	-		-	
peak 28	1946.992	-	1945.985	-	2675.678	1+	-	-	-	-	-		-	
peak 29	1958.996	-	1957.989	-	2306.803	1+	-	-	-	-	-		-	
peak 30	1974.990	-	1973.983	-	3792.318	1+	-	-	-	-	-		-	
peak 31	2051.984	-	2050.977	-	1178.753	1+	-	-	-	-	-		-	
peak 33	2097.010	-	2096.002	-	909.939	1+	-	-	-	-	-		-	
peak 35	2202.139	-	2201.131	-	4169.993	1+	-	-	-	-	-		-	
peak 36	2211.099	-	2210.092	-	6762.257	1+	-	-	-	-	-		-	
peak 37	2401.110	-	2400.103	-	727.211	1+	-	-	-	-	-		-	

Global peptide results

unnamed protein product [Mus musculus] gi|26336999
MW:61132.820

MIWVVLMMAILLPQSLAHPGFFTSIGQMTDLIHNEKDLVTSLKDYIKAEEDKLEQIKKWAEKLDRLTSTATKDPEGFVGHPVNAFKLMKRLNTEWSELENLILKDMSDGFISNLTIQRQYFPNDEDQVGAAKALFRLQDTYNLDTNTISKGNLPGVQHKSFLTAEDCFELGKVAYTEADYYHTELWMEQALTQLEEGELSTVDKVSVLDYL
SYAVYQQGDLDKALLLTKKLLELDPEHQRANGNLVYFEYIMSKEKDANKSASGDQSDQKTAPKKKGIAVDYLPERQKYEMLCRGEGIKMTPRRQKRLFCRYHDGNRNPKFILAPAKQEDEWDKPRIIRFHDIISDAEIEIVKDLAKPRLSRATVHDPETGKLTTAQYRVSKSAWLSGYEDPVVSRINMRIQDLTGLDVSTAEELQVANYGV
GGQYEPHFDFARKDEPDAFRELGTGNRIATWLFYMSDVSAGGATVFPEVGASVWPKKGTAVFWYNLFASGEGDYSTRHAACPVLVGNKWVSNKWLHERGQEFRRPCTLSELE

Digest Matches (Score: 284.00)
Score = 284.000000, Rank = 1, Database = NCBInr, Accesskey = gi|26336999
Search Parameters: MS Tol.:100.00 ppm, MSMS Tol.:0.600000Da, Enz.Trypsin, Engine:Mascot Version:2.3.01.241, DB:NCBInr NCBInr, DB Version:NCBInr_20110715.fasta NCBInr_20110715.fasta
Modifications: Optional: Oxidation (M)

Tree hierarchy	Meas. M/z	Calc. MH+	Meas. Mr	Calc. Mr	Int.	z	Dev.(Da)	Dev.(ppm)	Score	MascotScore	Rt(min)	Range	P	Sequence
peak 1	751.394	751.403	750.387	750.396	894.269	1+	-0.009	-11.807	-	-	-	307 - 311	1	RLFCR 4: Carbamidomethyl (C)
peak 5	977.462	977.469	976.454	976.461	626.800	1+	-0.007	-7.247	-	-	-	435 - 442	1	KDEPDAFR

Copyright © 1999-2008 Bruker Daltonik GmbH

Spectrum Analysis Report

Date: 07/30/2011 Time: 12:59

FileName: D:\Data\Bernardo\2011_07_30\P5_18\0_O8\1\1SRef\pdata\1\PMF_LIFT.xml

peak 7	1100.515	1100.523	1099.508	1099.516	4879.268	1+	-0.008	-7.552	-	-	-	312 - 320	1	YHDGNRNPK
peak 8	1104.536	1104.535	1103.529	1103.528	787.263	1+	0.001	0.472	-	-	-	526 - 534	0	RPCTLSELE 3: Carbamidomethyl (C)
peak 9	1132.590	1132.600	1131.583	1131.592	2961.054	1+	-0.010	-8.524	-	-	-	277 - 286	0	GIAVDYLPER
peak 11	1249.642	1249.654	1248.634	1248.646	2228.572	1+	-0.012	-9.531	-	-	-	231 - 240	0	LLELDPEHQR
peak 12	1377.736	1377.748	1376.729	1376.741	2300.462	1+	-0.012	-8.858	-	-	-	230 - 240	1	KLLELDPEHQR
peak 13	1388.777	1388.753	1387.770	1387.746	2225.107	1+	0.024	17.363	-	-	-	277 - 288	1	GIAVDYLPERQK
peak 17	1581.731	1581.718	1580.723	1580.711	4440.154	1+	0.013	7.939	-	-	-	119 - 132	0	QYFPNDEDQVGAAK
peak 20	1664.799	1664.799	1663.791	1663.792	797.719	1+	-0.000	-0.201	-	-	-	241 - 254	0	ANGNLVYFEYIMSK 12: Oxidation (M)
MSMS 23	1721.828	1721.828	1720.821	1720.820	8262.058	1+	0.001	0.296	26	12	-	521 - 534	1	GQEFRRPCTLSELE 8: Carbamidomethyl (C)
MSMS 26	1887.954	1887.956	1886.946	1886.949	12588.922	1+	-0.002	-1.175	85	64	-	363 - 379	1	ATVHDPETGKLTTAQYR
peak 27	1942.993	1943.002	1941.986	1941.995	1085.158	1+	-0.009	-4.449	-	-	-	321 - 336	1	FILAPAKQEDEWDKPR
peak 32	2069.001	2069.009	2067.994	2068.001	2052.130	1+	-0.007	-3.564	-	-	-	119 - 136	1	QYFPNDEDQVGAAKALFR
MSMS 34	2113.071	2113.092	2112.064	2112.085	1395.447	1+	-0.021	-10.141	4	-	-	133 - 150	1	ALFRLQDTYNLDTNTISK
MSMS 38	2556.298	2556.305	2555.291	2555.298	1203.817	1+	-0.007	-2.816	12	53	-	137 - 159	1	LQDTYNLDTNTISKGNLPGVQHK

Copyright © 1999-2008 Bruker Daltonik GmbH
