# Supplementary material for: Proteomic Analysis of the Action of the Mycobacterium ulcerans Toxin Mycolactone: Targeting Host Cells Cytoskeleton and Collagen
Source: PLoS Negl Trop Dis. 2014 Aug 7;8(8):e3066. doi: 10.1371/journal.pntd.0003066 (PMC4125307; doi:10.1371/journal.pntd.0003066)
Supplement: Dataset S7 — MS and MS/MS data. (ZIP) [file pntd.0003066.s010.zip › MS Data/Spot 13 - P4ha1.pdf]

D:\Data\Bernardo\2011_07_30\P5_19\0_P7\1\1SRef

Comment 1

Comment 2

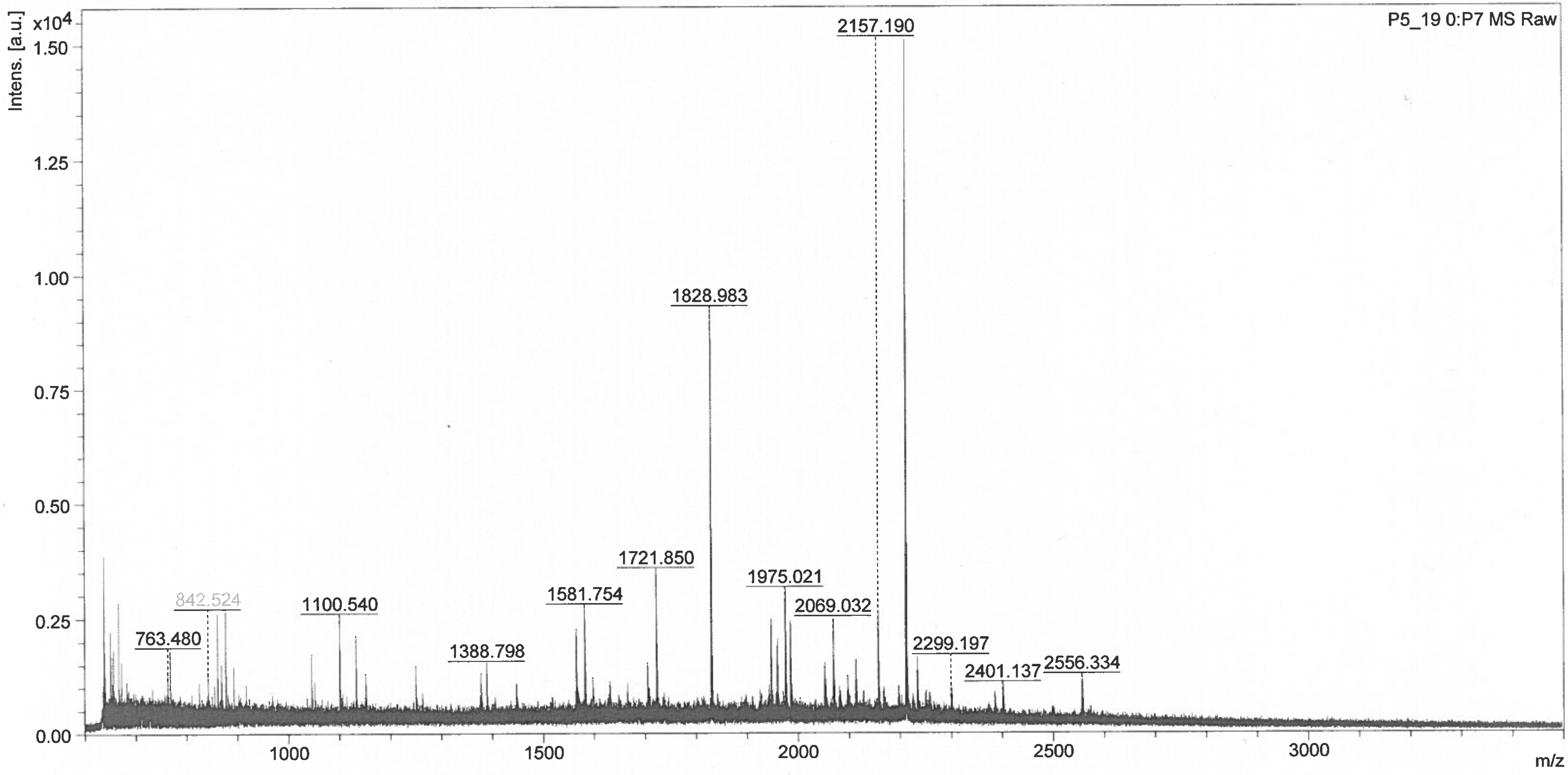

Bruker Daltonics flexAnalysis

printed: 7/30/2011 1:23:16 PM

Spectrum Analysis Report

Date: 07/30/2011 Time: 13:22

FileName: D:\Data\Bernardo\2011_07_30\P5_19\0_P7\1\1SRef\pdata\1\PMF_LIFT.xml

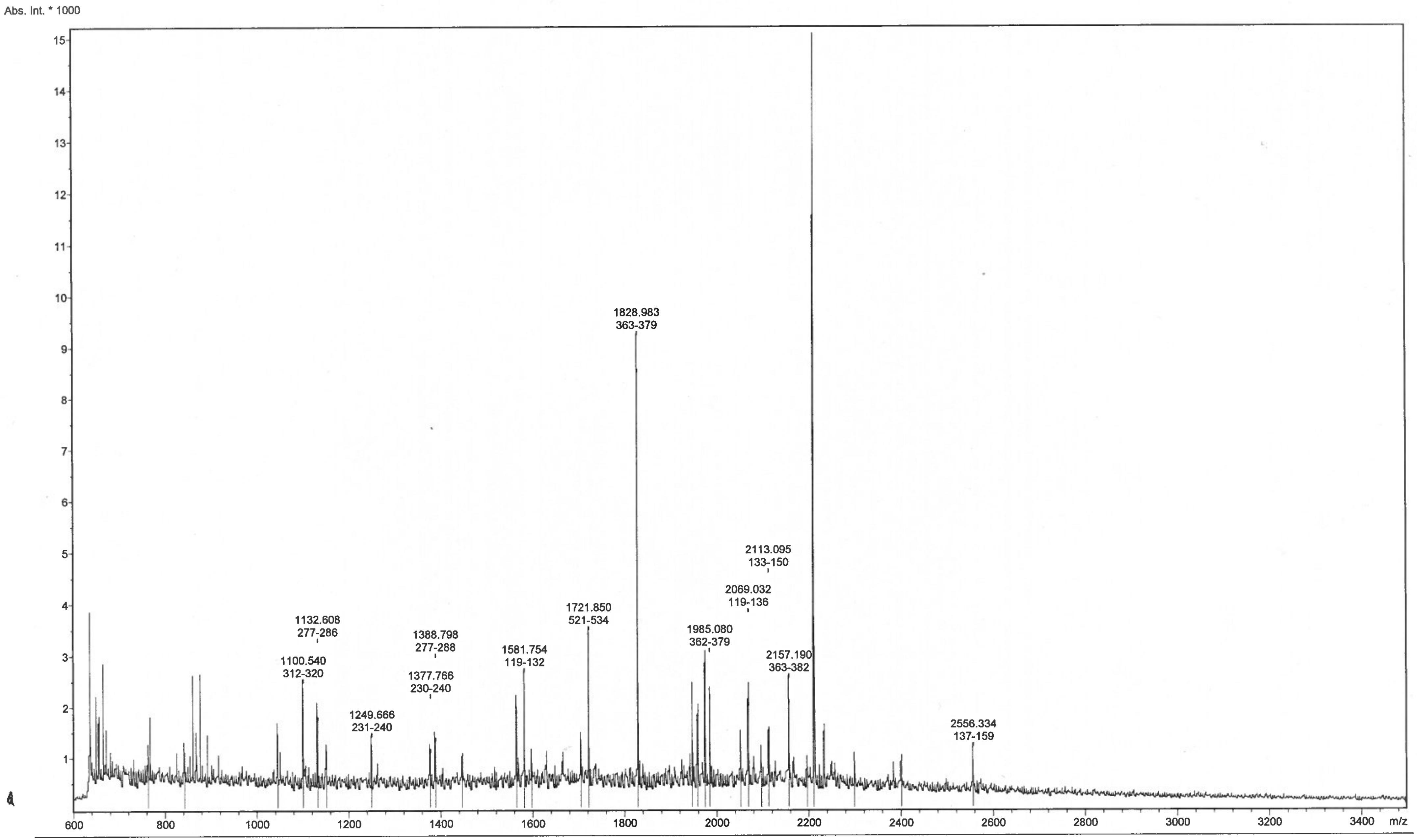

Copyright © 1999-2008 Bruker Daltonik GmbH

Spectrum Analysis Report

Date: 07/30/2011 Time: 13:22

FileName: D:\Data\Bernardo\2011_07_30\P5_19\0_P7\1\1SRef\pdata\1\PMF_LIFT.xml

Sequence data:

Prolyl 4-hydroxylase subunit alpha-1 OS=Mus musculus GN=P4ha1 PE=2 SV=2 P4HA1_MOUSE

Intensity Coverage:	48.3 % (30624 cnts)	Sequence Coverage MS:	20.2%
Sequence Coverage MS/MS:	5.8%	pI (isoelectric point):	5.5

10	20	30	40	50	60	70	80	90	100	110
MIWVVLMMAI	LLPQSLAHPG	FFTSIGQMTD	LIHNEKDLVT	SLKDYIKAEE	DKLEQIKKWA	EKLDRLTSTA	TKDPEGFVGH	PVNAFKLMKR	LNTEWSELEN	LILKDMSDGF
120	130	140	150	160	170	180	190	200	210	220
ISNLTIQRQY	FPNDEDQVGA	AKALFRLQDT	YNLDTNTISK	GNLPGVQHKS	FLTAEDCFEL	GKVAYTEADY	YHTELWMEQA	LTQLEEGELS	TVDKVSVLDY	LSYAVYQQGD
230	240	250	260	270	280	290	300	310	320	330
LDKALLLTKK	LLELDPEHQR	ANGNLVYFEY	IMSKEKDANK	SASGDQSDQK	TAPKKKGIAV	DYLPERQKYE	MLCRGEGIKM	TPRRQKRLFC	RYHDGNRNPK	FILAPAKQED
340	350	360	370	380	390	400	410	420	430	440
EWDKPRIIRF	HDIISDAEIE	IVKDLAKPRL	RRATISNPVT	GALETVHYRI	SKSAWLSGYE	DPVVSRINMR	IQDLTGLDVS	TAEELQVANY	GVGGQYEPHF	DFARKDEPDA
450	460	470	480	490	500	510	520	530	540	
FRELGTGNRI	ATWLFYMSDV	SAGGATVFPE	VGASVWPKKG	TAVFWYNLFA	SGEGDYSTRH	AACPVLVGNK	WVSNKWLHER	GQEFRRPCTL	SELE	

Acquisition Parameter:

Matched Sequences:

Unmatched

Peaks/MSMS Spectra

Tree hierarchy	Meas. M/z	Calc. MH+	Meas. Mr	Calc. Mr	Int.	z	Dev.(Da)	Dev.(ppm)	Score	MascotScore	Rt(min)	Range	P	Sequence
peak 1	763.480	-	762.473	-	1029.005	1+	-	-	-	-	-		-	
peak 2	842.524	-	841.517	-	1124.264	1+	-	-	-	-	-		-	
peak 3	1045.580	-	1044.572	-	1460.601	1+	-	-	-	-	-		-	
peak 6	1151.748	-	1150.740	-	1137.607	1+	-	-	-	-	-		-	
peak 10	1446.866	-	1445.859	-	839.212	1+	-	-	-	-	-		-	
peak 11	1564.725	-	1563.718	-	2007.894	1+	-	-	-	-	-		-	
peak 13	1597.769	-	1596.762	-	884.130	1+	-	-	-	-	-		-	
peak 14	1704.850	-	1703.843	-	1257.636	1+	-	-	-	-	-		-	
peak 17	1947.017	-	1946.010	-	2112.861	1+	-	-	-	-	-		-	
peak 18	1959.025	-	1958.018	-	1710.389	1+	-	-	-	-	-		-	
MSMS 19	1975.021	-	1974.014	-	2587.862	1+	-	-	-	-	-		-	
peak 21	2052.009	-	2051.002	-	1274.950	1+	-	-	-	-	-		-	
peak 23	2097.037	-	2096.030	-	889.638	1+	-	-	-	-	-		-	
peak 26	2197.061	-	2196.054	-	678.261	1+	-	-	-	-	-		-	
peak 27	2211.136	-	2210.129	-	12330.711	1+	-	-	-	-	-		-	
peak 28	2299.197	-	2298.190	-	754.533	1+	-	-	-	-	-		-	
peak 29	2401.137	-	2400.130	-	638.545	1+	-	-	-	-	-		-	

Global peptide results

Prolyl 4-hydroxylase subunit alpha-1 OS=Mus musculus GN=P4ha1 PE=2 SV=2 P4HA1_MOUSE
MW:61156.900

MIWVVLMMAILLPQSLAHPGFFTSIGQMTDLIHNEKDLVTSLKDYIKAEEDKLEQIKKWAEKLDRLTSTATKDPEGFVGHPVNAFKLMKRLNTEWSELENLILKDMSDGFISNLTIQRQYFPNDEDQVGAAKALFRLQDTYNLDTNTISKGNLPGVQHKSFLTAEDCFELGKVAYTEADYYHTELWMEQALTQLEEGELSTVDKVSVLDYL
SYAVYQQGDLDKALLLTKKLLELDPEHQRANGNLVYFEYIMSKEKDANKSASGDQSDQKTAPKKKGIAVDYLPERQKYEMLCRGEGIKMTPRRQKRLFCRYHDGNRNPKFILAPAKQEDEWDKPRIIRFHDIISDAEIEIVKDLAKPRLRRATISNPVTGALETVHYRISKSAWLSGYEDPVVSRINMRIQDLTGLDVSTAEELQVANYGV
GGQYEPHFDFARKDEPDAFRELGTGNRIATWLFYMSDVSAGGATVFPEVGASVWPKKGTAVFWYNLFASGEGDYSTRHAACPVLVGNKWVSNKWLHERGQEFRRPCTLSELE

Digest Matches (Score: 160.00)
Score = 160.000000, Rank = 1, Database = SwissProt, Accesskey = P4HA1_MOUSE
Search Parameters: MS Tol.:100.00 ppm, MSMS Tol.:0.600000Da, Enz.Trypsin, Engine:Mascot Version:2.3.01.241, DB:NCBInr NCBInr, DB Version:NCBInr_20110715.fasta NCBInr_20110715.fasta
Modifications: Optional: Oxidation (M)

Tree hierarchy	Meas. M/z	Calc. MH+	Meas. Mr	Calc. Mr	Int.	z	Dev.(Da)	Dev.(ppm)	Score	MascotScore	Rt(min)	Range	P	Sequence
peak 4	1100.540	1100.523	1099.532	1099.516	2379.653	1+	0.017	15.084	-	-	-	312 - 320	1	YHDGNRNPK
peak 5	1132.608	1132.600	1131.601	1131.592	1735.077	1+	0.008	7.260	-	-	-	277 - 286	0	GIAVDYLPER
peak 7	1249.666	1249.654	1248.659	1248.646	1256.687	1+	0.013	10.402	-	-	-	231 - 240	0	LLELDPEHQR
peak 8	1377.766	1377.748	1376.759	1376.741	1099.180	1+	0.018	12.959	-	-	-	230 - 240	1	KLLELDPEHQR
peak 9	1388.798	1388.753	1387.790	1387.746	1323.698	1+	0.044	32.034	-	-	-	277 - 288	1	GIAVDYLPERQK
peak 12	1581.754	1581.718	1580.747	1580.711	2468.673	1+	0.036	22.949	-	-	-	119 - 132	0	QYFPNDEDQVGAAK

Copyright © 1999-2008 Bruker Daltonik GmbH

MSMS 15	1721.850	1721.828	1720.843	1720.820	3451.870	1+	0.023	13.317	8	8	-	521	-	534	1	GQEFRRPCTLSELE 8: Carbamidomethyl (C)
MSMS 16	1828.983	1828.955	1827.975	1827.948	8503.726	1+	0.027	15.014	27	17	-	363	-	379	0	ATISNPVTGALETVHYR
peak 20	1985.080	1985.056	1984.072	1984.049	2165.334	1+	0.023	11.767	-	-	-	362	-	379	1	RATISNPVTGALETVHYR
peak 22	2069.032	2069.009	2068.024	2068.001	2012.944	1+	0.023	11.044	-	-	-	119	-	136	1	QYFPNDEDQVGAAKALFR
peak 24	2113.095	2113.092	2112.087	2112.085	1229.637	1+	0.002	1.014	-	-	-	133	-	150	1	ALFRLQDTYNLDTNTISK
peak 25	2157.190	2157.166	2156.183	2156.159	2066.825	1+	0.024	11.092	-	-	-	363	-	382	1	ATISNPVTGALETVHYRISK
peak 30	2556.334	2556.305	2555.327	2555.298	930.445	1+	0.029	11.372	-	-	-	137	-	159	1	LQDTYNLDTNTISKGNLPGVQHK

Copyright © 1999-2008 Bruker Daltonik GmbH
