# Supplementary material for: Proteomic Analysis of the Action of the Mycobacterium ulcerans Toxin Mycolactone: Targeting Host Cells Cytoskeleton and Collagen
Source: PLoS Negl Trop Dis. 2014 Aug 7;8(8):e3066. doi: 10.1371/journal.pntd.0003066 (PMC4125307; doi:10.1371/journal.pntd.0003066)
Supplement: Dataset S7 — MS and MS/MS data. (ZIP) [file pntd.0003066.s010.zip › MS Data/Spot 14 - Fdps.pdf]

Spectrum Analysis Report

Date: 07/29/2011 Time: 07:27

FileName: D:\Data\Bernardo\2011_07_28\M5_23\0_K9\1\1SRef\pdata\1\PMF_LIFT.xml

Sequence data:

Farnesyl pyrophosphate synthase OS=Mus musculus GN=Fdps PE=2 SV=1 FPPS_MOUSE

Intensity Coverage:	21.1 % (24173 cnts)	Sequence Coverage MS:	44.5%
Sequence Coverage MS/MS:	5.1%	pI (isoelectric point):	5.4

```
        10         20         30         40         50         60         70         80         90        100        110
MNGNQKLDAY NQEKQNFIQH FSQIVKVLTE KELGHPEIGD AIARLKEVLE YNALGGKYNR GLTVVQAFQE LVEPKKQDAE SLQRALTVGW CVELLQAFFL VSDDIMDSSL
       120        130        140        150        160        170        180        190        200        210        220
TRRGQICWYQ KPGIGLDAIN DALLLEASIY RLLKFYCREQ PYYLNLLELF LQSSYQTEIG QTLDLMTAPQ GHVDLGRYTE KRYKSIVKYK TAFYSFYLPI AAAMYMAGID
       230        240        250        260        270        280        290        300        310        320        330
GEKEHANALK ILMEMGEFFQ VQDDYLDLFG DPSVTGKVGT DIQDNKCSWL VVQCLLRASP QQRQILEENY GQKDPEKVAR VKALYEALDL QSAFFKYEED SYNRLKSLIE
       340        350        360
QCSAPLPPSI FMELANKIYK RRK
```

Acquisition Parameter:

Matched Sequences:

Unmatched

Peaks/MSMS Spectra

Tree hierarchy	Meas. M/z	Calc. MH+	Meas. Mr	Calc. Mr	Int.	z	Dev.(Da)	Dev.(ppm)	Score	MascotScore	Rt(min)	Range	P	Sequence
peak 1	763.490	-	762.483	-	2496.807	1+	-	-	-	-	-		-	
peak 2	842.520	-	841.513	-	1374.076	1+	-	-	-	-	-		-	
peak 3	1262.966	-	1261.959	-	837.991	1+	-	-	-	-	-		-	
peak 5	1471.783	-	1470.775	-	975.543	1+	-	-	-	-	-		-	
peak 6	1483.792	-	1482.785	-	2098.013	1+	-	-	-	-	-		-	
peak 8	1771.899	-	1770.891	-	1220.656	1+	-	-	-	-	-		-	
peak 9	1775.941	-	1774.933	-	1168.161	1+	-	-	-	-	-		-	
peak 10	1787.932	-	1786.925	-	1018.516	1+	-	-	-	-	-		-	
peak 11	1799.891	-	1798.883	-	1098.445	1+	-	-	-	-	-		-	
peak 12	1803.892	-	1802.884	-	973.650	1+	-	-	-	-	-		-	
peak 13	1812.893	-	1811.886	-	983.307	1+	-	-	-	-	-		-	
peak 14	1867.024	-	1866.017	-	2638.686	1+	-	-	-	-	-		-	
peak 15	1879.028	-	1878.021	-	2883.428	1+	-	-	-	-	-		-	
peak 16	1891.019	-	1890.011	-	971.893	1+	-	-	-	-	-		-	
peak 17	1895.015	-	1894.007	-	1427.132	1+	-	-	-	-	-		-	
peak 18	1907.012	-	1906.005	-	1825.156	1+	-	-	-	-	-		-	
peak 19	1935.010	-	1934.003	-	988.258	1+	-	-	-	-	-		-	
peak 21	1960.058	-	1959.050	-	951.212	1+	-	-	-	-	-		-	
peak 22	1982.022	-	1981.015	-	945.165	1+	-	-	-	-	-		-	
peak 23	2000.039	-	1999.032	-	2825.194	1+	-	-	-	-	-		-	
peak 24	2011.022	-	2010.015	-	1108.749	1+	-	-	-	-	-		-	
peak 25	2017.061	-	2016.053	-	1767.453	1+	-	-	-	-	-		-	
peak 26	2028.041	-	2027.034	-	1571.118	1+	-	-	-	-	-		-	
peak 27	2040.048	-	2039.041	-	957.359	1+	-	-	-	-	-		-	
peak 29	2211.139	-	2210.132	-	2444.009	1+	-	-	-	-	-		-	
peak 30	2223.138	-	2222.131	-	1032.947	1+	-	-	-	-	-		-	
peak 31	2288.178	-	2287.171	-	3300.528	1+	-	-	-	-	-		-	
peak 32	2299.185	-	2298.177	-	1058.252	1+	-	-	-	-	-		-	
peak 33	2302.176	-	2301.168	-	1434.452	1+	-	-	-	-	-		-	
peak 34	2316.187	-	2315.180	-	1179.956	1+	-	-	-	-	-		-	
peak 35	2320.168	-	2319.161	-	3412.029	1+	-	-	-	-	-		-	
peak 36	2327.221	-	2326.214	-	1055.039	1+	-	-	-	-	-		-	
peak 37	2346.199	-	2345.192	-	1039.918	1+	-	-	-	-	-		-	
peak 38	2352.154	-	2351.147	-	1055.032	1+	-	-	-	-	-		-	
peak 39	2360.223	-	2359.215	-	1413.767	1+	-	-	-	-	-		-	
peak 41	2414.242	-	2413.234	-	979.733	1+	-	-	-	-	-		-	
peak 42	2417.242	-	2416.234	-	1416.308	1+	-	-	-	-	-		-	
peak 43	2420.245	-	2419.238	-	983.494	1+	-	-	-	-	-		-	

Copyright © 1999-2008 Bruker Daltonik GmbH

peak 44	2432.250	-	2431.243	-	1955.940	1+	-	-	-	-	-		-	
peak 45	2436.229	-	2435.221	-	961.416	1+	-	-	-	-	-		-	
peak 46	2444.256	-	2443.249	-	1132.638	1+	-	-	-	-	-		-	
peak 48	2462.279	-	2461.271	-	2712.668	1+	-	-	-	-	-		-	
peak 49	2474.270	-	2473.262	-	961.476	1+	-	-	-	-	-		-	
peak 51	2654.282	-	2653.274	-	883.270	1+	-	-	-	-	-		-	
peak 53	2684.290	-	2683.283	-	1076.437	1+	-	-	-	-	-		-	
peak 54	2695.469	-	2694.461	-	1813.958	1+	-	-	-	-	-		-	
MSMS 55	2713.484	-	2712.477	-	6870.832	1+	-	-	-	-	-		-	
peak 56	2723.456	-	2722.448	-	794.770	1+	-	-	-	-	-		-	
peak 57	2725.461	-	2724.454	-	908.450	1+	-	-	-	-	-		-	
peak 58	2741.479	-	2740.471	-	3067.017	1+	-	-	-	-	-		-	
peak 59	2753.473	-	2752.466	-	857.322	1+	-	-	-	-	-		-	
peak 60	2769.472	-	2768.465	-	1844.550	1+	-	-	-	-	-		-	
peak 61	2785.437	-	2784.430	-	3063.953	1+	-	-	-	-	-		-	
peak 63	2899.449	-	2898.442	-	2249.167	1+	-	-	-	-	-		-	
peak 64	2912.465	-	2911.458	-	1012.375	1+	-	-	-	-	-		-	
peak 65	2921.551	-	2920.544	-	775.829	1+	-	-	-	-	-		-	
peak 67	3181.598	-	3180.590	-	700.596	1+	-	-	-	-	-		-	

Global peptide results

Farnesyl pyrophosphate synthase OS=Mus musculus GN=Fdps PE=2 SV=1 FPPS_MOUSE
MW:40897.820

MNGNQKLDAYNQEKQNFIQHFSQIVKVLTEKELGHPEIGDAIARLKEVLEYNALGGKYNRGLTVVQAFQELVEPKKQDAESLQRALTVGWCVELLQAFFLVSDDIMDSSLTRRGQICWYQKPGIGLDAINDALLLEASIYRLLKFYCREQPYYLNLLELFLQSSYQTEIGQTLDLMTAPQGHVDLGRYTEKRYKSIVKYKTAFYSFYLPIA
AAMYMAGIDGEKEHANALKILMEMGEFFQVQDDYLDLFGDPSVTGKVGTDIQDNKCSWLVVQCLLRASPQQRQILEENYGQKDPEKVARVKALYEALDLQSAFFKYEEDSYNRLKSLIEQCSAPLPPSIFMELANKIYKRRK

Digest Matches (Score: 112.00)
Score = 112.000000, Rank = 1, Database = SwissProt, Accesskey = FPPS_MOUSE
Search Parameters: MS Tol.:100.00 ppm, MSMS Tol.: 0.600000 Da, Enz.Trypsin, Engine: Mascot Version: 2.3.01.241 , DB: NCBInr NCBInr , DB Version: NCBInr_20110715.fasta NCBInr_20110715.fasta
Modifications: Optional: Oxidation (M)

Tree hierarchy	Meas. M/z	Calc. MH+	Meas. Mr	Calc. Mr	Int.	z	Dev.(Da)	Dev.(ppm)	Score	MascotScore	Rt(min)	Range	P	Sequence
peak 4	1377.720	1377.712	1376.713	1376.705	1003.118	1+	0.008	5.594	-	-	-	32 - 44	0	ELGHPEIGDAIAR
peak 7	1657.948	1657.916	1656.941	1656.909	3457.194	1+	0.032	19.237	-	-	-	61 - 75	0	GLTVVQAFQELVEPK
MSMS 20	1948.075	1948.050	1947.068	1947.043	7137.543	1+	0.026	13.135	15	21	-	27 - 44	1	VLTEKELGHPEIGDAIAR
peak 28	2091.139	2091.123	2090.131	2090.116	1809.733	1+	0.015	7.370	-	-	-	58 - 75	1	YNRGLTVVQAFQELVEPK
peak 40	2404.234	2404.211	2403.227	2403.204	1777.776	1+	0.023	9.627	-	-	-	258 - 277	1	VGTDIQDNKCSWLVVQCLLR 10: Carbamidomethyl (C) 17: Carbamidomethyl (C)
peak 47	2450.272	2450.246	2449.264	2449.239	3667.395	1+	0.025	10.365	-	-	-	7 - 26	1	LDAYNQEKQNFIQHFSQIVK
peak 50	2562.260	2562.193	2561.253	2561.186	841.178	1+	0.067	26.130	-	-	-	201 - 223	0	TAFYSFYLPIAAAMYMAGIDGEK 14: Oxidation (M) 16: Oxidation (M)
peak 52	2672.283	2672.251	2671.276	2671.244	2340.058	1+	0.032	11.964	-	-	-	303 - 324	1	ALYEALDLQSAFFKYEEDSYNR
peak 62	2853.479	2853.351	2852.471	2852.344	927.022	1+	0.127	44.640	-	-	-	199 - 223	1	YKTAFYSFYLPIAAAMYMAGIDGEK 16: Oxidation (M) 18: Oxidation (M)
peak 66	3126.549	3126.432	3125.542	3125.425	699.548	1+	0.117	37.338	-	-	-	231 - 257	0	ILMEMGEFFQVQDDYLDLFGDPSVTGK 3: Oxidation (M) 5: Oxidation (M)
peak 68	3325.667	3325.591	3324.660	3324.583	512.485	1+	0.077	23.071	-	-	-	201 - 230	1	TAFYSFYLPIAAAMYMAGIDGEKEHANALK 14: Oxidation (M) 16: Oxidation (M)

mCG17543, isoform CRA_a [Mus musculus] gi|148683291
MW:48906.800

MPLSCWLRSLGVFLLPAPCWAPRERWLGFLQRPSLAYGCPVLGAWHSARWWCQVWTEEPRAFSSSVRMNGNQKLDAYNQEKQNFIQHFSQIVKVLTEKELGHPEIGDAIARLKEVLEYNALGGKYNRGLTVVQAFQELVEPKKQDAESLQRALTVGWCVELLQAFFLVSDDIMDSSLTRRGQICWYQKPGIGLDAINDALLLEASIYRLLK
FYCREQPYYLNLLELFLQSSYQTEIGQTLDLMTAPQGHVDLGRYTEKRYKSIVKYKTAFYSFYLPIAAAMYMAGIDGEKEHANALKILMEMGEFFQVQDDYLDLFGDPSVTGKVGTDIQDNKCSWLVVQCLLRASPQQRQILEENYGQKDPEKVARVKALYEALDLQSAFFKYEEDSYNRLKSLIEQCSAPLPPSIFMELANKIYKRRK

Digest Matches (Score: 105.00)
Score = 105.000000, Rank = 1, Database = NCBInr, Accesskey = gi|148683291
Search Parameters: MS Tol.:100.00 ppm, MSMS Tol.: 0.600000 Da, Enz.Trypsin, Engine: Mascot Version: 2.3.01.241 , DB: NCBInr NCBInr , DB Version: NCBInr_20110715.fasta NCBInr_20110715.fasta
Modifications: Optional: Oxidation (M)

Tree hierarchy	Meas. M/z	Calc. MH+	Meas. Mr	Calc. Mr	Int.	z	Dev.(Da)	Dev.(ppm)	Score	MascotScore	Rt(min)	Range	P	Sequence
peak 4	1377.720	1377.712	1376.713	1376.705	1003.118	1+	0.008	5.594	-	-	-	99 - 111	0	ELGHPEIGDAIAR
peak 7	1657.948	1657.916	1656.941	1656.909	3457.194	1+	0.032	19.237	-	-	-	128 - 142	0	GLTVVQAFQELVEPK
MSMS 20	1948.075	1948.050	1947.068	1947.043	7137.543	1+	0.026	13.135	15	21	-	94 - 111	1	VLTEKELGHPEIGDAIAR
peak 28	2091.139	2091.123	2090.131	2090.116	1809.733	1+	0.015	7.370	-	-	-	125 - 142	1	YNRGLTVVQAFQELVEPK
peak 40	2404.234	2404.211	2403.227	2403.204	1777.776	1+	0.023	9.627	-	-	-	325 - 344	1	VGTDIQDNKCSWLVVQCLLR 10: Carbamidomethyl (C) 17: Carbamidomethyl (C)
peak 47	2450.272	2450.246	2449.264	2449.239	3667.395	1+	0.025	10.365	-	-	-	74 - 93	1	LDAYNQEKQNFIQHFSQIVK
peak 50	2562.260	2562.193	2561.253	2561.186	841.178	1+	0.067	26.130	-	-	-	268 - 290	0	TAFYSFYLPIAAAMYMAGIDGEK 14: Oxidation (M) 16: Oxidation (M)
peak 52	2672.283	2672.251	2671.276	2671.244	2340.058	1+	0.032	11.964	-	-	-	370 - 391	1	ALYEALDLQSAFFKYEEDSYNR
peak 62	2853.479	2853.351	2852.471	2852.344	927.022	1+	0.127	44.640	-	-	-	266 - 290	1	YKTAFYSFYLPIAAAMYMAGIDGEK 16: Oxidation (M) 18: Oxidation (M)
peak 66	3126.549	3126.432	3125.542	3125.425	699.548	1+	0.117	37.338	-	-	-	298 - 324	0	ILMEMGEFFQVQDDYLDLFGDPSVTGK 3: Oxidation (M) 5: Oxidation (M)
peak 68	3325.667	3325.591	3324.660	3324.583	512.485	1+	0.077	23.071	-	-	-	268 - 297	1	TAFYSFYLPIAAAMYMAGIDGEKEHANALK 14: Oxidation (M) 16: Oxidation (M)

Copyright © 1999-2008 Bruker Daltonik GmbH
