# Supplementary material for: Proteomic Analysis of the Action of the Mycobacterium ulcerans Toxin Mycolactone: Targeting Host Cells Cytoskeleton and Collagen
Source: PLoS Negl Trop Dis. 2014 Aug 7;8(8):e3066. doi: 10.1371/journal.pntd.0003066 (PMC4125307; doi:10.1371/journal.pntd.0003066)
Supplement: Dataset S7 — MS and MS/MS data. (ZIP) [file pntd.0003066.s010.zip › MS Data/Spot 15 - Unc119b.pdf]

D:\Data\Bernardo\2011_07_29\M5_48\0_K22\1\1SRef

Comment 1
Comment 2

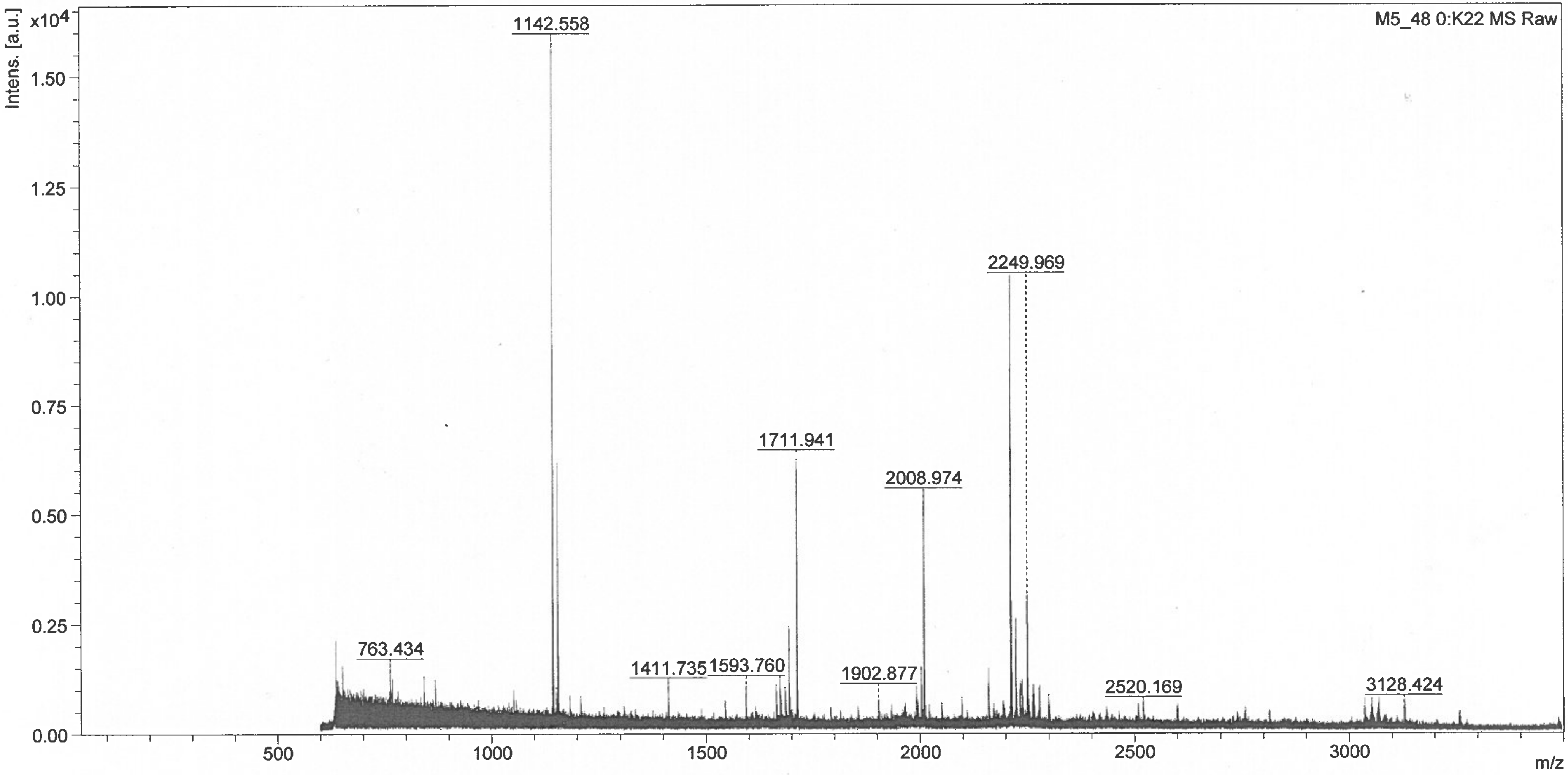

Spectrum Analysis Report

Date: 07/29/2011 Time: 08:33

FileName: D:\Data\Bernardo\2011_07_29\M5_48\0_K22\1\1SRef\pdata\1\PMF_LIFT.xml

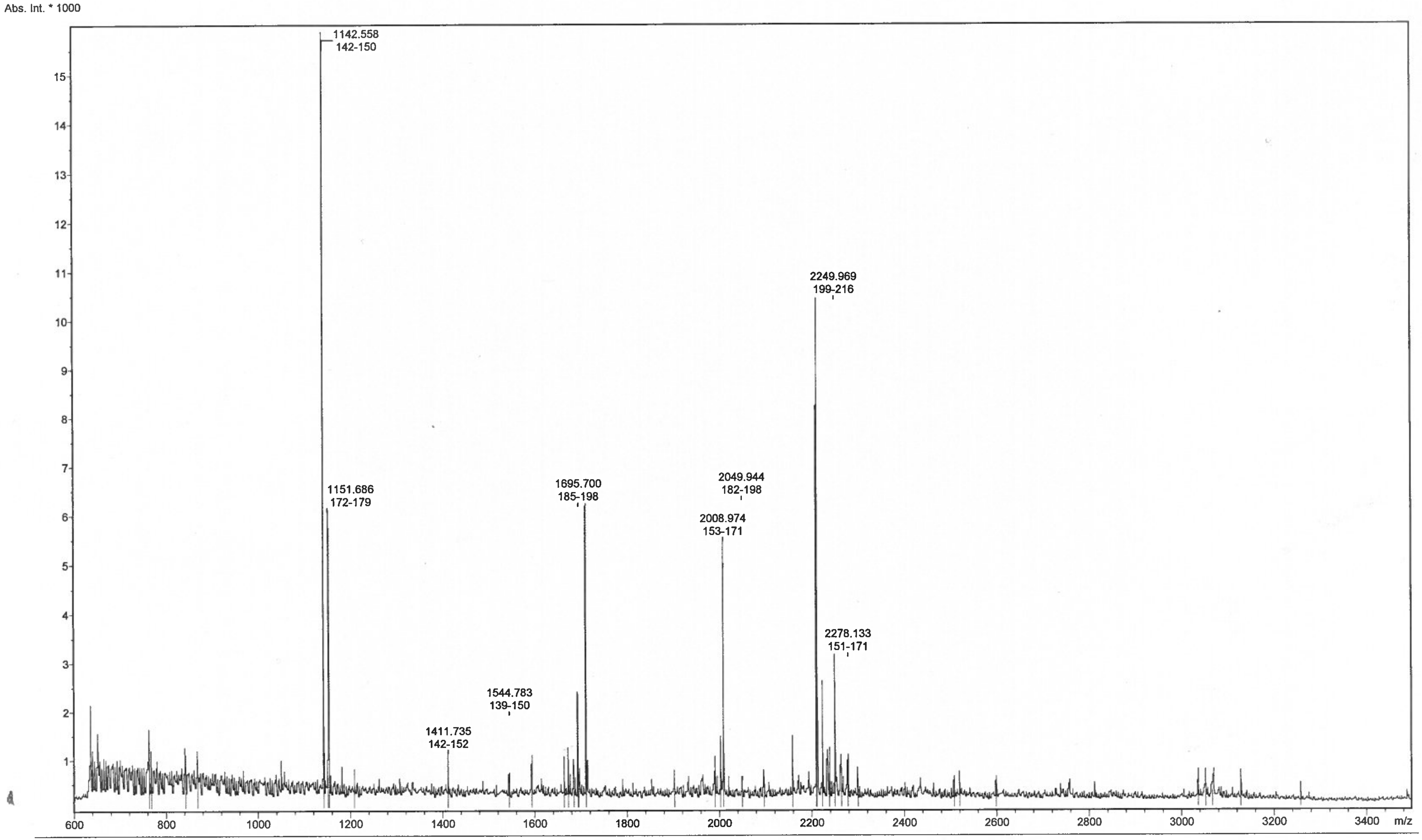

Copyright © 1999-2008 Bruker Daltonik GmbH

Spectrum Analysis Report

Date: 07/29/2011 Time: 08:33

FileName: D:\Data\Bernardo\2011_07_29\M5_48\0_K22\1\1SRef\pdata\1\PMF_LIFT.xml

Sequence data:

Protein unc-119 homolog B OS=Mus musculus GN=Unc119b PE=2 SV=1 U119B_MOUSE

Intensity Coverage: 40.5 % (28828 cnts)
Sequence Coverage MS/MS: 11.2%
Sequence Coverage MS: 30.3%
pI (isoelectric point): 5.4

10	20	30	40	50	60	70	80	90	100	110
MSGSNPKAAT	AGSQAGPGGL	VAGKEEKKKA	GGGVLNRLKA	RRQGPPHTPD	DGSGAAVTEQ	ELLALDTIRP	EHVLRLNRVT	ENYLCKPEDN	VYSIDFTRFK	IRDLETGTVL
120	130	140	150	160	170	180	190	200	210	220
FEIAKPCISD	QDQDAEEESV	DVDISVGRFV	RYQFTPAFLR	LRTVGATVEF	TVGDRPVTGF	RMIERHYFRE	RLLKTFDFDF	GFCIPSSRNT	CEHIYEFPQL	SEDVIRLMIE
230	240	250	260							
NPYETRSDSF	YFVDNKLVMH	NKADYAYNGG	Q							

Acquisition Parameter:

Matched Sequences:

Unmatched

Peaks/MSMS Spectra

Tree hierarchy	Meas. M/z	Calc. MH+	Meas. Mr	Calc. Mr	Int.	z	Dev.(Da)	Dev.(ppm)	Score	MascotScore	Rt(min)	Range	P	Sequence
peak 1	763.434	-	762.427	-	1372.264	1+	-	-	-	-	-		-	
peak 2	768.493	-	767.485	-	862.418	1+	-	-	-	-	-		-	
peak 3	842.469	-	841.462	-	1107.718	1+	-	-	-	-	-		-	
peak 4	868.515	-	867.508	-	1019.154	1+	-	-	-	-	-		-	
peak 7	1154.554	-	1153.547	-	5744.932	1+	-	-	-	-	-		-	
peak 8	1208.574	-	1207.567	-	626.936	1+	-	-	-	-	-		-	
peak 11	1593.760	-	1592.753	-	836.376	1+	-	-	-	-	-		-	
peak 12	1663.732	-	1662.724	-	873.638	1+	-	-	-	-	-		-	
peak 13	1672.761	-	1671.754	-	996.157	1+	-	-	-	-	-		-	
peak 14	1684.768	-	1683.761	-	888.558	1+	-	-	-	-	-		-	
peak 15	1693.929	-	1692.921	-	2360.064	1+	-	-	-	-	-		-	
MSMS 17	1711.941	-	1710.933	-	6384.177	1+	-	-	-	-	-		-	
peak 18	1902.877	-	1901.869	-	528.281	1+	-	-	-	-	-		-	
peak 19	1990.954	-	1989.946	-	819.858	1+	-	-	-	-	-		-	
peak 20	2002.864	-	2001.856	-	1374.598	1+	-	-	-	-	-		-	
peak 23	2097.053	-	2096.046	-	648.344	1+	-	-	-	-	-		-	
peak 24	2158.965	-	2157.958	-	1000.090	1+	-	-	-	-	-		-	
peak 25	2209.929	-	2208.922	-	949.774	1+	-	-	-	-	-		-	
peak 26	2211.030	-	2210.023	-	8374.437	1+	-	-	-	-	-		-	
peak 27	2223.028	-	2222.020	-	2036.529	1+	-	-	-	-	-		-	
peak 28	2238.087	-	2237.079	-	922.178	1+	-	-	-	-	-		-	
peak 30	2263.981	-	2262.974	-	807.379	1+	-	-	-	-	-		-	
peak 32	2300.079	-	2299.071	-	548.908	1+	-	-	-	-	-		-	
peak 33	2508.229	-	2507.221	-	391.336	1+	-	-	-	-	-		-	
peak 34	2520.169	-	2519.162	-	479.677	1+	-	-	-	-	-		-	
peak 35	2599.244	-	2598.237	-	438.518	1+	-	-	-	-	-		-	
peak 36	3036.345	-	3035.338	-	337.359	1+	-	-	-	-	-		-	
peak 37	3052.340	-	3051.333	-	353.715	1+	-	-	-	-	-		-	
peak 38	3067.334	-	3066.327	-	353.560	1+	-	-	-	-	-		-	
peak 39	3128.424	-	3127.417	-	396.923	1+	-	-	-	-	-		-	
peak 40	3257.514	-	3256.506	-	257.378	1+	-	-	-	-	-		-	

Global peptide results

expressed sequence AA407659, isoform CRA_b [Mus musculus] gi|148687934
MW:19397.400

RKQGCRVGTSVICQSVACWVGTWVLTAQLCALADQDQDAEEESVDVDISVGRFVRYQFTPAFLRLRTVGATVEFTVGDRPVTGFRMIERHYFRERLLKTFDFDFGFCIPSSRNTCEHIYEFPQLSEDVIRLMIENPYETRSDSFYFVDNKLVMHNKADYAYNGGQ

Digest Matches (Score: 130.00)
Score = 130.000000, Rank = 1, Database = NCBInr, Accesskey = gi|148687934
Search Parameters: MS Tol.:100.00 ppm, MSMS Tol.:0.600000Da, Enz.Trypsin, Engine:Mascot Version:2.3.01.241, DB:NCBInr NCBInr, DB Version:NCBInr_20110715.fasta NCBInr_20110715.fasta
Modifications: Optional: Oxidation (M)

Tree hierarchy	Meas. M/z	Calc. MH+	Meas. Mr	Calc. Mr	Int.	z	Dev.(Da)	Dev.(ppm)	Score	MascotScore	Rt(min)	Range	P	Sequence
MSMS 5	1142.558	1142.599	1141.551	1141.592	15512.646	1+	-0.041	-36.211	20	13	-	56 - 64	0	YQFTPAFLR
peak 6	1151.686	1151.578	1150.679	1150.571	644.261	1+	0.108	94.080	-	-	-	86 - 93	1	MIERHYFR

Copyright © 1999-2008 Bruker Daltonik GmbH

peak 9	1411.735	1411.784	1410.728	1410.777	972.304	1+	-0.050	-35.183	-	-	-	56 - 66	1	YQFTPAFLRLR
peak 10	1544.783	1544.837	1543.775	1543.830	550.932	1+	-0.055	-35.323	-	-	-	53 - 64	1	FVRYQFTPAFLR
peak 16	1695.700	1695.747	1694.693	1694.740	628.741	1+	-0.047	-27.923	-	-	-	99 - 112	0	TFDFDFGFCIPSSR 9: Carbamidomethyl (C)
MSMS 21	2008.974	2009.045	2007.967	2008.038	4940.920	1+	-0.071	-35.197	21	8	-	67 - 85	0	TVGATVEFTVGDRPVTGFR
peak 22	2049.944	2050.010	2048.936	2049.003	572.693	1+	-0.067	-32.516	-	-	-	96 - 112	1	LLKTFDFDFGFCIPSSR 12: Carbamidomethyl (C)
peak 29	2249.969	2250.050	2248.961	2249.042	2501.304	1+	-0.081	-35.998	-	-	-	113 - 130	0	NTCEHIYEFPQLSEDVIR 3: Carbamidomethyl (C)
peak 31	2278.133	2278.230	2277.126	2277.223	772.681	1+	-0.097	-42.765	-	-	-	65 - 85	1	LRTVGATVEFTVGDRPVTGFR

Protein unc-119 homolog B OS=Mus musculus GN=Unc119b PE=2 SV=1 U119B_MOUSE
MW:28513.210

MSGSNPKAATAGSQAGPGGLVAGKEEKKKAGGGVLNRLKARRQGPPHTPDDGSGAAVTEQELLALDTIRPEHVLRLNRVTENYLCKPEDNVYSIDFTRFKIRDLETGTVLFEIAKPCISDQDQDAEEESVDVDISVGRFVRYQFTPAFLRLRTVGATVEFTVGDRPVTGFRMIERHYFRERLLKTFDFDFGFCIPSSRNTCEHIYEFPQLS
EDVIRLMIENPYETRSDSFYFVDNKLVMHNKADYAYNGGQ

Digest Matches (Score: 114.00)
Score = 114.000000, Rank = 1, Database = SwissProt, Accesskey = U119B_MOUSE
Search Parameters: MS Tol.:100.00 ppm, MSMS Tol.:0.600000Da, Enz.Trypsin, Engine:Mascot Version:2.3.01.241, DB:NCBInr NCBInr, DB Version:NCBInr_20110715.fasta NCBInr_20110715.fasta
Modifications: Optional: Oxidation (M)

Tree hierarchy	Meas. M/z	Calc. MH+	Meas. Mr	Calc. Mr	Int.	z	Dev.(Da)	Dev.(ppm)	Score	MascotScore	Rt(min)	Range	P	Sequence
MSMS 5	1142.558	1142.599	1141.551	1141.592	15512.646	1+	-0.041	-36.211	20	13	-	142 - 150	0	YQFTPAFLR
peak 6	1151.686	1151.578	1150.679	1150.571	644.261	1+	0.108	94.080	-	-	-	172 - 179	1	MIERHYFR
peak 9	1411.735	1411.784	1410.728	1410.777	972.304	1+	-0.050	-35.183	-	-	-	142 - 152	1	YQFTPAFLRLR
peak 10	1544.783	1544.837	1543.775	1543.830	550.932	1+	-0.055	-35.323	-	-	-	139 - 150	1	FVRYQFTPAFLR
peak 16	1695.700	1695.747	1694.693	1694.740	628.741	1+	-0.047	-27.923	-	-	-	185 - 198	0	TFDFDFGFCIPSSR 9: Carbamidomethyl (C)
MSMS 21	2008.974	2009.045	2007.967	2008.038	4940.920	1+	-0.071	-35.197	21	8	-	153 - 171	0	TVGATVEFTVGDRPVTGFR
peak 22	2049.944	2050.010	2048.936	2049.003	572.693	1+	-0.067	-32.516	-	-	-	182 - 198	1	LLKTFDFDFGFCIPSSR 12: Carbamidomethyl (C)
peak 29	2249.969	2250.050	2248.961	2249.042	2501.304	1+	-0.081	-35.998	-	-	-	199 - 216	0	NTCEHIYEFPQLSEDVIR 3: Carbamidomethyl (C)
peak 31	2278.133	2278.230	2277.126	2277.223	772.681	1+	-0.097	-42.765	-	-	-	151 - 171	1	LRTVGATVEFTVGDRPVTGFR

Copyright © 1999-2008 Bruker Daltonik GmbH
