# Supplementary material for: Proteomic Analysis of the Action of the Mycobacterium ulcerans Toxin Mycolactone: Targeting Host Cells Cytoskeleton and Collagen
Source: PLoS Negl Trop Dis. 2014 Aug 7;8(8):e3066. doi: 10.1371/journal.pntd.0003066 (PMC4125307; doi:10.1371/journal.pntd.0003066)
Supplement: Dataset S7 — MS and MS/MS data. (ZIP) [file pntd.0003066.s010.zip › MS Data/Spot 16 - Psme3.pdf]

D:\Data\Bernardo\2011_07_29\M5_47\0_K21\1\1SRef

Comment 1

Comment 2

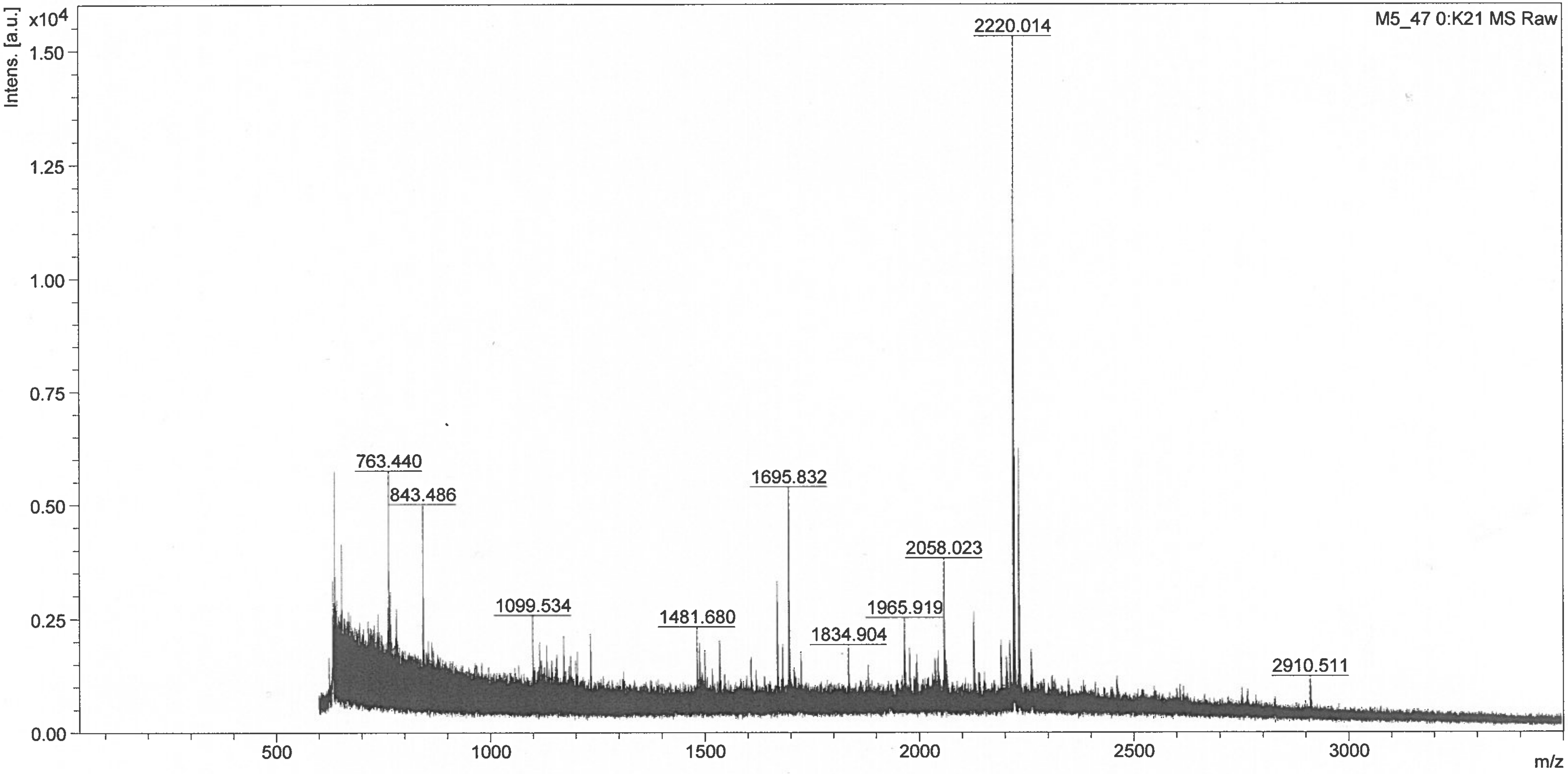

Bruker Daltonics flexAnalysis printed: 7/29/2011 8:28:37 AM

Spectrum Analysis Report

Date: 07/29/2011 Time: 08:29

FileName: D:\Data\Bernardo\2011_07_29\M5_47\0_K21\1\1SRef\pdata\1\PMF_LIFT.xml

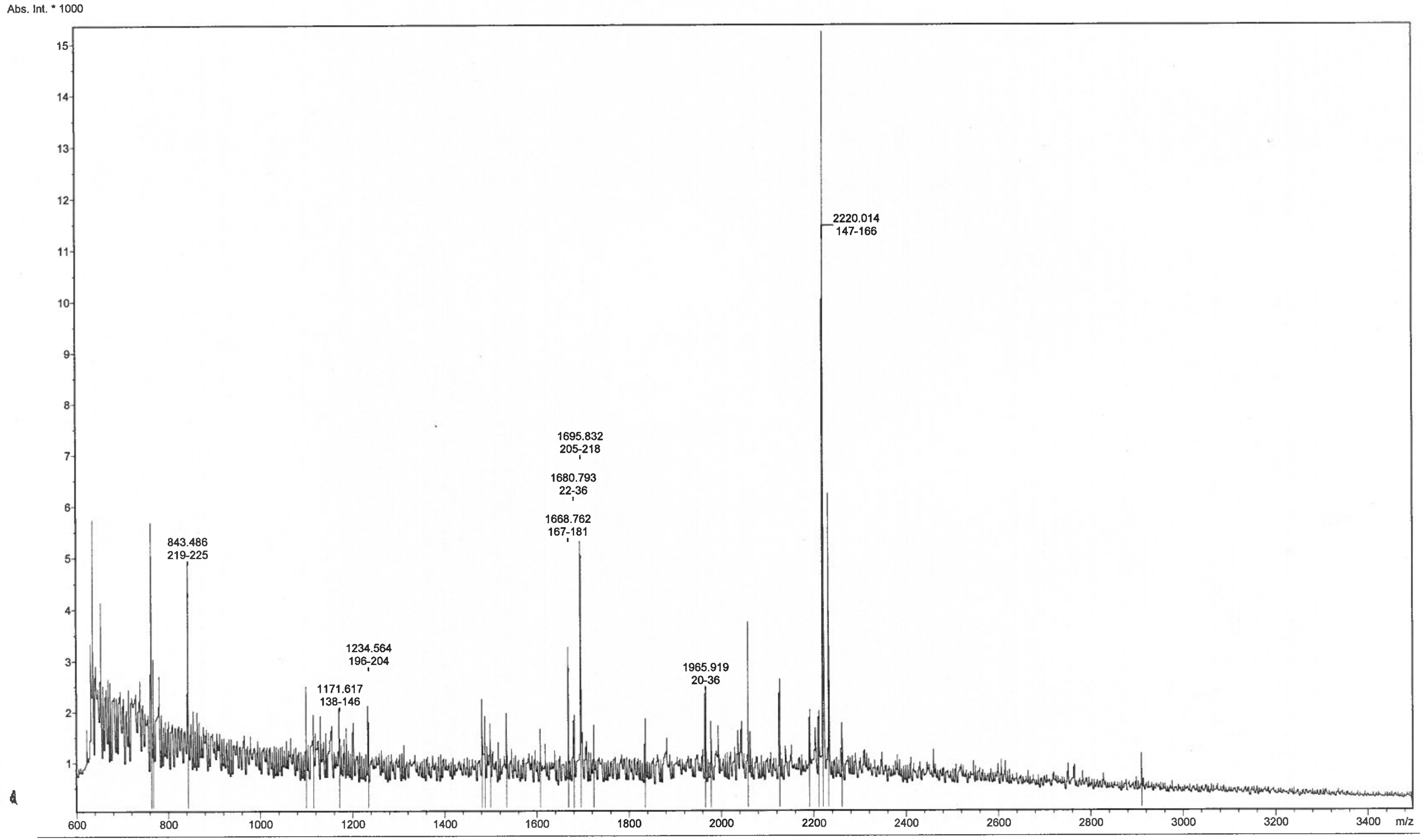

Copyright © 1999-2008 Bruker Daltonik GmbH

Spectrum Analysis Report

Date: 07/29/2011 Time: 08:29

FileName: D:\Data\Bernardo\2011_07_29\M5_47\0_K21\1\1SRef\pdata\1\PMF_LIFT.xml

Sequence data:

Proteasome activator complex subunit 3 OS=Mus musculus GN=Psme3 PE=1 SV=1 PSME3_MOUSE

Intensity Coverage:	45.1 % (29755 cnts)	Sequence Coverage MS:	35.8%
Sequence Coverage MS/MS:	13.4%	pI (isoelectric point):	5.6

10	20	30	40	50	60	70	80	90	100	110
MASLLKVDQE	VKLKVDSFRE	RITSEAEDLV	ANFFPKKLLE	LDSFLKEPIL	NIHDLTQIHS	DMNLPVPDPI	LLTNSHDGLD	GPTYKKRRLD	ECEEAFQGTK	VFVMPNGMLK
**120**	**130**	**140**	**150**	**160**	**170**	**180**	**190**	**200**	**210**	**220**
SNQQLVDIIE	KVKPEIRLLI	EKCNTVKMWV	QLLIPRIEDG	NNFGVSIQEE	TVAELRTVES	EAASYLDQIS	RYYITRAKLV	SKIAKYPHVE	DYRRTVTEID	EKEYISLRLI
**230**	**240**	**250**	**260**							
ISELRNQYVT	LHDMILKNIE	KIKRPRSSNA	ETLY							

Acquisition Parameter:

Matched Sequences:

Unmatched

Peaks/MSMS Spectra

Tree hierarchy	Meas. M/z	Calc. MH+	Meas. Mr	Calc. Mr	Int.	z	Dev.(Da)	Dev.(ppm)	Score	MascotScore	Rt(min)	Range	P	Sequence
peak 1	763.440	-	762.433	-	5035.287	1+	-	-	-	-	-		-	
peak 2	767.455	-	766.448	-	2157.537	1+	-	-	-	-	-		-	
peak 4	1099.534	-	1098.527	-	1976.112	1+	-	-	-	-	-		-	
peak 5	1115.538	-	1114.531	-	1524.947	1+	-	-	-	-	-		-	
peak 8	1481.680	-	1480.673	-	1761.291	1+	-	-	-	-	-		-	
peak 9	1487.703	-	1486.696	-	1392.380	1+	-	-	-	-	-		-	
peak 10	1499.715	-	1498.708	-	1260.625	1+	-	-	-	-	-		-	
peak 11	1534.705	-	1533.698	-	1626.415	1+	-	-	-	-	-		-	
peak 12	1607.791	-	1606.783	-	1374.967	1+	-	-	-	-	-		-	
peak 16	1723.824	-	1722.816	-	1241.895	1+	-	-	-	-	-		-	
peak 17	1834.904	-	1833.897	-	1243.989	1+	-	-	-	-	-		-	
peak 19	1977.936	-	1976.929	-	1251.019	1+	-	-	-	-	-		-	
MSMS 20	2058.023	-	2057.015	-	2991.753	1+	-	-	-	-	-		-	
peak 21	2126.916	-	2125.909	-	1822.604	1+	-	-	-	-	-		-	
peak 22	2191.046	-	2190.039	-	1491.354	1+	-	-	-	-	-		-	
peak 23	2211.032	-	2210.025	-	1434.867	1+	-	-	-	-	-		-	
MSMS 25	2232.010	-	2231.003	-	4740.729	1+	-	-	-	-	-		-	
peak 26	2260.997	-	2259.990	-	1178.061	1+	-	-	-	-	-		-	
peak 27	2910.511	-	2909.504	-	674.774	1+	-	-	-	-	-		-	

Global peptide results

Proteasome activator complex subunit 3 OS=Mus musculus GN=Psme3 PE=1 SV=1 PSME3_MOUSE

MW:29601.600

MASLLKVDQEVKLKVDSFRERITSEAEDLVANFFPKKLLELDSFLKEPILNIHDLTQIHSDMNLPVPDPILLTNSHDGLDGPTYKKRRLDECEEAFQGTKVFVMPNGMLKSNQQLVDIIEKVKPEIRLLIEKCNTVKMWVQLLIPRIEDGNNFGVSIQEETVAELRTVESEAASYLDQISRYYITRAKLVSKIAKYPHVEDYRRTVTEIDE
KEYISLRLIISELRNQYVTLHDMILKNIEKIKRPRSSNAETLY

Digest Matches (Score: 126.00)

Score = 126.000000, Rank = 1, Database = SwissProt, Accesskey = PSME3_MOUSE

Search Parameters: MS Tol.:100.00 ppm, MSMS Tol.:0.600000Da, Enz.Trypsin, Engine:Mascot Version:2.3.01.241, DB:NCBInr NCBInr, DB Version:NCBInr_20110715.fasta NCBInr_20110715.fasta

Modifications: Optional: Oxidation (M)

Tree hierarchy	Meas. M/z	Calc. MH+	Meas. Mr	Calc. Mr	Int.	z	Dev.(Da)	Dev.(ppm)	Score	MascotScore	Rt(min)	Range	P	Sequence
peak 3	843.486	843.530	842.479	842.523	4249.707	1+	-0.044	-51.860	-	-	-	219 - 225	0	LIISELR
peak 6	1171.617	1171.666	1170.610	1170.658	1397.756	1+	-0.048	-41.365	-	-	-	138 - 146	0	MWVQLLIPR 1: Oxidation (M)
peak 7	1234.564	1234.596	1233.557	1233.589	1722.163	1+	-0.032	-25.804	-	-	-	196 - 204	1	YPHVEDYRR
peak 13	1668.762	1668.807	1667.755	1667.800	2781.089	1+	-0.046	-27.317	-	-	-	167 - 181	0	TVESEAASYLDQISR
peak 14	1680.793	1680.848	1679.786	1679.841	1391.007	1+	-0.055	-32.604	-	-	-	22 - 36	0	ITSEAEDLVANFFPK
MSMS 15	1695.832	1695.880	1694.825	1694.873	4966.147	1+	-0.048	-28.209	10	3	-	205 - 218	1	TVTEIDEKEYISLR
peak 18	1965.919	1965.992	1964.912	1964.984	2026.300	1+	-0.073	-36.911	-	-	-	20 - 36	1	ERITSEAEDLVANFFPK
MSMS 24	2220.014	2220.078	2219.007	2219.071	11221.218	1+	-0.064	-28.862	21	38	-	147 - 166	0	IEDGNNFGVSIQEETVAELR

P61290

Copyright © 1999-2008 Bruker Daltonik GmbH
