# Supplementary material for: Proteomic Analysis of the Action of the Mycobacterium ulcerans Toxin Mycolactone: Targeting Host Cells Cytoskeleton and Collagen
Source: PLoS Negl Trop Dis. 2014 Aug 7;8(8):e3066. doi: 10.1371/journal.pntd.0003066 (PMC4125307; doi:10.1371/journal.pntd.0003066)
Supplement: Dataset S7 — MS and MS/MS data. (ZIP) [file pntd.0003066.s010.zip › MS Data/Spot 17 - Ftl1.pdf]

D:\Data\Bernardo\2011_07_27\M23_15\0_I17\1\1SRef

Comment 1

Comment 2

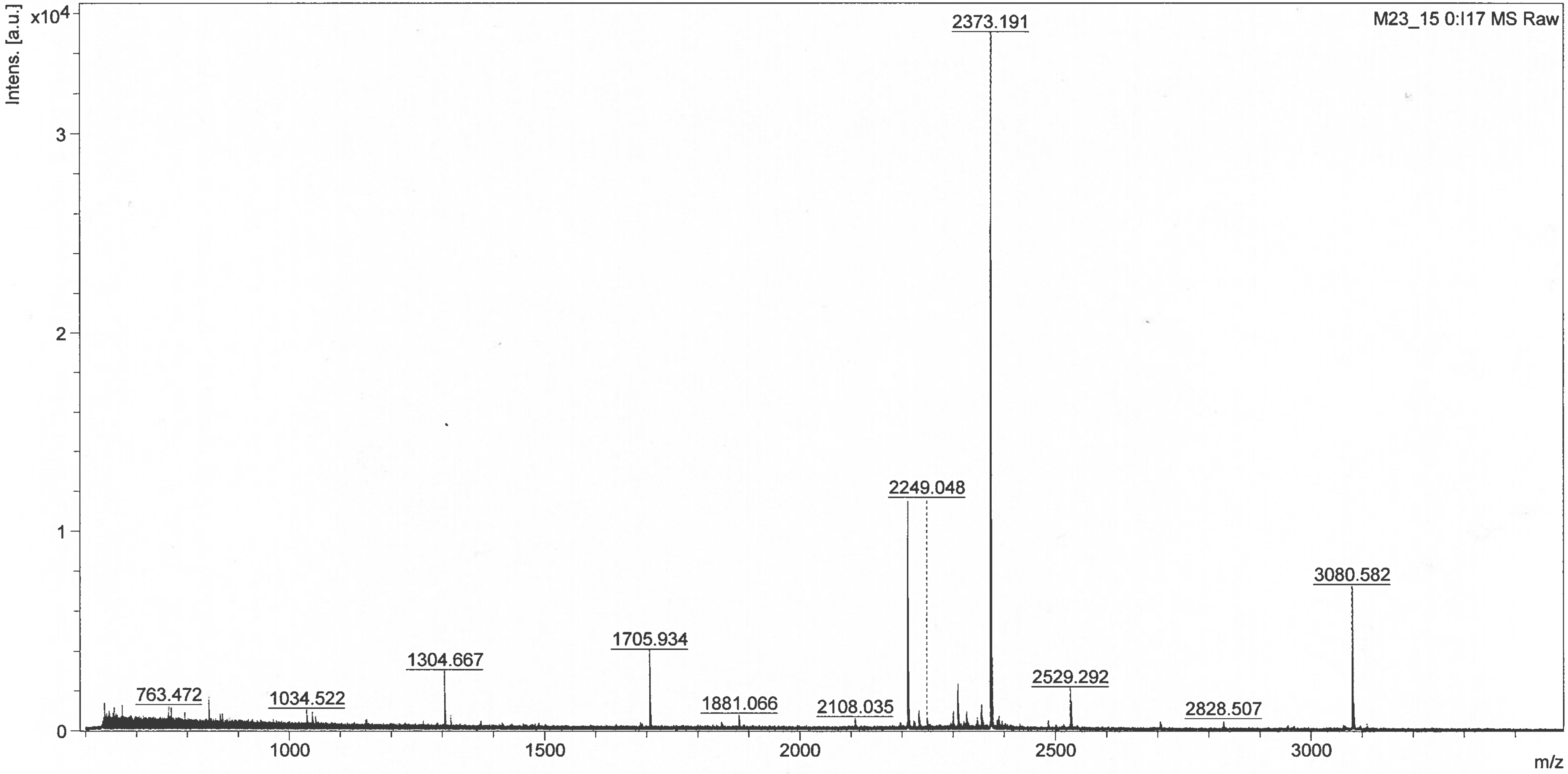

Bruker Daltonics flexAnalysis printed: 7/29/2011 6:55:26 AM

Spectrum Analysis Report

Date: 07/29/2011 Time: 06:54

FileName: D:\Data\Bernardo\2011_07_27\M23_15\0_I17\1\1SRef\pdata\1\PMF_LIFT.xml

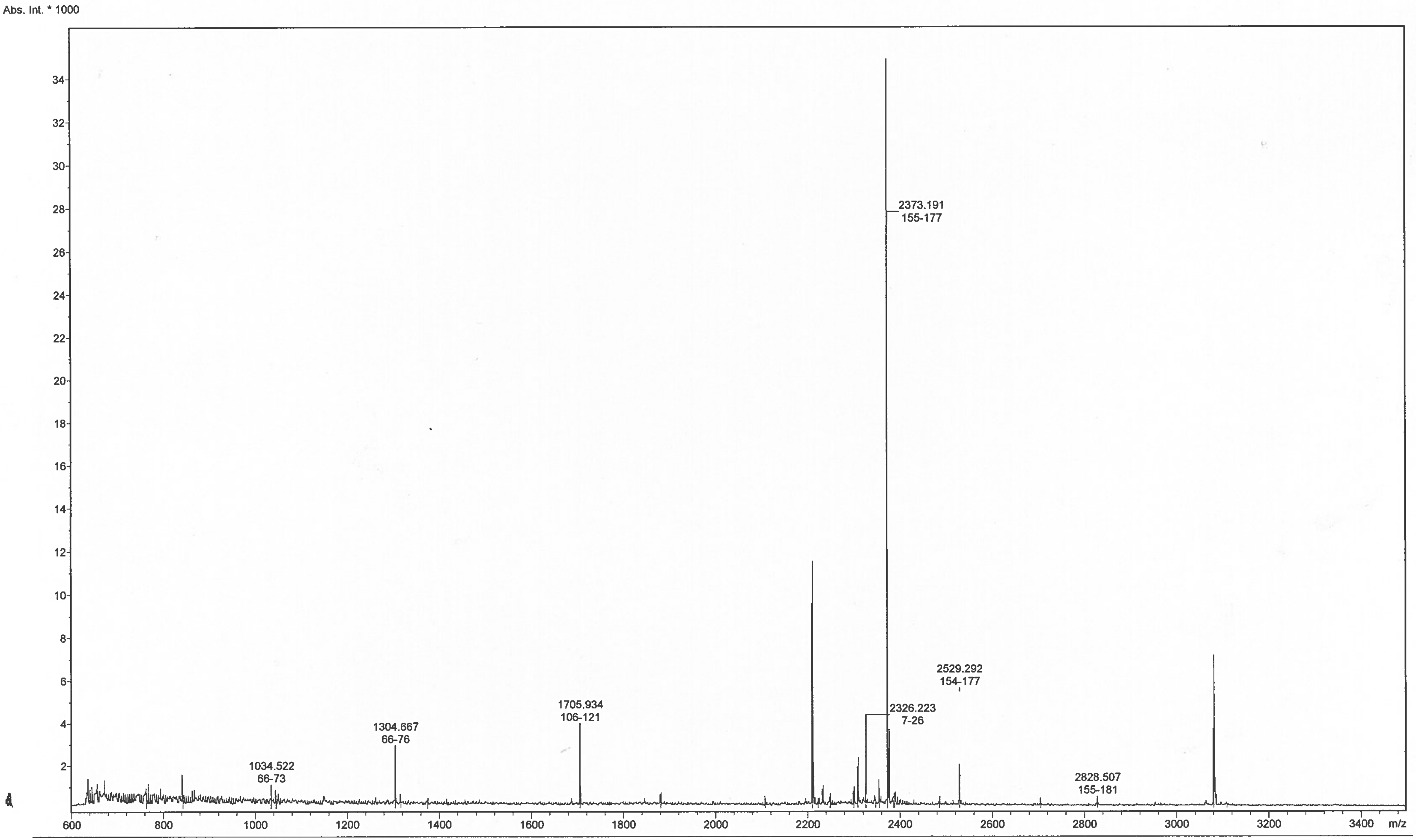

Copyright © 1999-2008 Bruker Daltonik GmbH

Sequence data:

Ferritin light chain 1 OS=Mus musculus GN=Ftl1 PE=1 SV=2 FRIL1_MOUSE

Intensity Coverage:	61.2 % (37224 cnts)	Sequence Coverage MS:	41.0%
Sequence Coverage MS/MS:	12.6%	pI (isoelectric point):	5.6

```
        10          20          30          40          50          60
MTSQIRQNYS  TEVEAAVNRL  VNLHLRASYT  YLSLGFFFDR  DDVALEGVGH  FFRELAEEKR
        70          80          90         100         110         120
EGAERLLEFQ  NDRGGRALFQ  DVQKPSQDEW  GKTQEAMEAA  LAMEKNLNQA  LLDLHALGSA
       130         140         150         160         170         180
RTDPHLCDFL  ESHYLDKEVK  LIKKMGNHLT  NLRRVAGPQP  AQTGAPQGSL  GEYLFERLTL
       190
KHD
```

Acquisition Parameter:

Matched Sequences:

Unmatched

Peaks/MSMS Spectra

Tree hierarchy	Meas. M/z	Calc. MH+	Meas. Mr	Calc. Mr	Int.	z	Dev.(Da)	Dev.(ppm)	Score	MascotScore	Rt(min)	Range	P	Sequence
peak 1	763.472	-	762.465	-	727.019	1+	-	-	-	-	-		-	
peak 2	842.500	-	841.492	-	1361.323	1+	-	-	-	-	-		-	
peak 4	1045.563	-	1044.556	-	613.142	1+	-	-	-	-	-		-	
peak 7	1375.038	-	1374.031	-	413.002	1+	-	-	-	-	-		-	
peak 9	1881.066	-	1880.059	-	610.660	1+	-	-	-	-	-		-	
peak 10	2108.035	-	2107.028	-	455.591	1+	-	-	-	-	-		-	
peak 11	2211.117	-	2210.110	-	8950.566	1+	-	-	-	-	-		-	
peak 12	2223.091	-	2222.084	-	368.850	1+	-	-	-	-	-		-	
peak 13	2249.048	-	2248.041	-	463.328	1+	-	-	-	-	-		-	
peak 15	2309.213	-	2308.205	-	1760.526	1+	-	-	-	-	-		-	
peak 17	2347.286	-	2346.279	-	393.546	1+	-	-	-	-	-		-	
peak 18	2355.176	-	2354.168	-	915.641	1+	-	-	-	-	-		-	
peak 20	2385.188	-	2384.181	-	349.603	1+	-	-	-	-	-		-	
peak 21	2388.175	-	2387.168	-	490.503	1+	-	-	-	-	-		-	
peak 22	2485.280	-	2484.272	-	316.281	1+	-	-	-	-	-		-	
peak 24	2705.157	-	2704.150	-	261.131	1+	-	-	-	-	-		-	
peak 26	3080.582	-	3079.574	-	3852.804	1+	-	-	-	-	-		-	
peak 27	3109.569	-	3108.562	-	126.152	1+	-	-	-	-	-		-	

Global peptide results

mCG118431 [Mus musculus] gi|148701888
MW:10004.190
NPGGMEAALALEKNLNQALLDLHALGSARADPHLCDFLESHFLDKEVKLIKKMGNHLTNLRRVAGPQPAQTGAPQGSLGEYLFERLTLKHD

Digest Matches (Score: 228.00)
Score = 228.000000, Rank = 1, Database = NCBInr, Accesskey = gi|148701888
Search Parameters: MS Tol.:100.00 ppm, MSMS Tol.: 0.600000 Da, Enz.Trypsin, Engine: Mascot Version: 2.3.01.241 , DB: NCBInr NCBInr , DB Version: NCBInr_20110715.fasta NCBInr_20110715.fasta
Modifications: Optional: Oxidation (M)

Tree hierarchy	Meas. M/z	Calc. MH+	Meas. Mr	Calc. Mr	Int.	z	Dev.(Da)	Dev.(ppm)	Score	MascotScore	Rt(min)	Range	P	Sequence
peak 6	1316.657	1316.651	1315.650	1315.644	489.141	1+	0.006	4.411	-	-	-	1 - 13	0	NPGGMEAALALEK 5: Oxidation (M)
peak 8	1705.934	1705.934	1704.926	1704.927	3999.878	1+	-0.001	-0.435	-	-	-	14 - 29	0	NLNQALLDLHALGSAR
peak 14	2300.175	2300.102	2299.167	2299.094	689.201	1+	0.073	31.746	-	-	-	30 - 48	1	ADPHLCDFLESHFLDKEVK 6: Carbamidomethyl (C)
MSMS 19	2373.191	2373.183	2372.184	2372.176	27244.818	1+	0.008	3.268	1063	127	-	63 - 85	0	VAGPQPAQTGAPQGSLGEYLFER
peak 23	2529.292	2529.284	2528.284	2528.277	1391.715	1+	0.007	2.895	-	-	-	62 - 85	1	RVAGPQPAQTGAPQGSLGEYLFER
peak 25	2828.507	2828.494	2827.499	2827.487	275.252	1+	0.012	4.407	-	-	-	63 - 89	1	VAGPQPAQTGAPQGSLGEYLFERLTLK

Ferritin light chain 1 OS=Mus musculus GN=Ftl1 PE=1 SV=2 FRIL1_MOUSE
MW:20846.520
MTSQIRQNYSTEVEAAVNRLVNLHLRASYTYLSLGFFFDRDDVALEGVGHFFRELAEEKREGAERLLEFQNDRGGRALFQDVQKPSQDEWGKTQEAMEAALAMEKNLNQALLDLHALGSARTDPHLCDFLESHYLDKEVKLIKKMGNHLTNLRRVAGPQPAQTGAPQGSLGEYLFERLTLKHD

Digest Matches (Score: 226.00)

Copyright © 1999-2008 Bruker Daltonik GmbH

Spectrum Analysis Report

Date: 07/29/2011 Time: 06:54

FileName: D:\Data\Bernardo\2011_07_27\M23_15\0_I17\1\1SRef\pdata\1\PMF_LIFT.xml

Score = 226.000000, Rank = 1, Database = SwissProt, Accesskey = FRIL1_MOUSE
Search Parameters: MS Tol.:100.00 ppm, MSMS Tol.: 0.600000 Da, Enz.Trypsin, Engine: Mascot Version: 2.3.01.241 , DB: NCBInr NCBInr , DB Version: NCBInr_20110715.fasta NCBInr_20110715.fasta
Modifications: Optional: Oxidation (M)

Tree hierarchy	Meas. M/z	Calc. MH+	Meas. Mr	Calc. Mr	Int.	z	Dev.(Da)	Dev.(ppm)	Score	MascotScore	Rt(min)	Range	P	Sequence
peak 3	1034.522	1034.527	1033.515	1033.519	844.266	1+	-0.005	-4.535	-	-	-	66 - 73	0	LLEFQNDR
peak 5	1304.667	1304.671	1303.660	1303.663	2825.023	1+	-0.003	-2.471	-	-	-	66 - 76	1	LLEFQNDRGGR
peak 8	1705.934	1705.934	1704.926	1704.927	3999.878	1+	-0.001	-0.435	-	-	-	106 - 121	0	NLNQALLDLHALGSAR
peak 16	2326.223	2326.226	2325.216	2325.219	642.822	1+	-0.003	-1.429	-	-	-	7 - 26	1	QNYSTEVEAAVNRLVNLHLR
MSMS 19	2373.191	2373.183	2372.184	2372.176	27244.818	1+	0.008	3.268	1063	127	-	155 - 177	0	VAGPQPAQTGAPQGSLGEYLFER
peak 23	2529.292	2529.284	2528.284	2528.277	1391.715	1+	0.007	2.895	-	-	-	154 - 177	1	RVAGPQPAQTGAPQGSLGEYLFER
peak 25	2828.507	2828.494	2827.499	2827.487	275.252	1+	0.012	4.407	-	-	-	155 - 181	1	VAGPQPAQTGAPQGSLGEYLFERLTLK

Copyright © 1999-2008 Bruker Daltonik GmbH
