# Supplementary material for: Proteomic Analysis of the Action of the Mycobacterium ulcerans Toxin Mycolactone: Targeting Host Cells Cytoskeleton and Collagen
Source: PLoS Negl Trop Dis. 2014 Aug 7;8(8):e3066. doi: 10.1371/journal.pntd.0003066 (PMC4125307; doi:10.1371/journal.pntd.0003066)
Supplement: Dataset S7 — MS and MS/MS data. (ZIP) [file pntd.0003066.s010.zip › MS Data/Spot 18 - Stmn1.pdf]

D:\Data\Bernardo\2011_07_30\P5_09\0_O7\1\1SRef

Comment 1

Comment 2

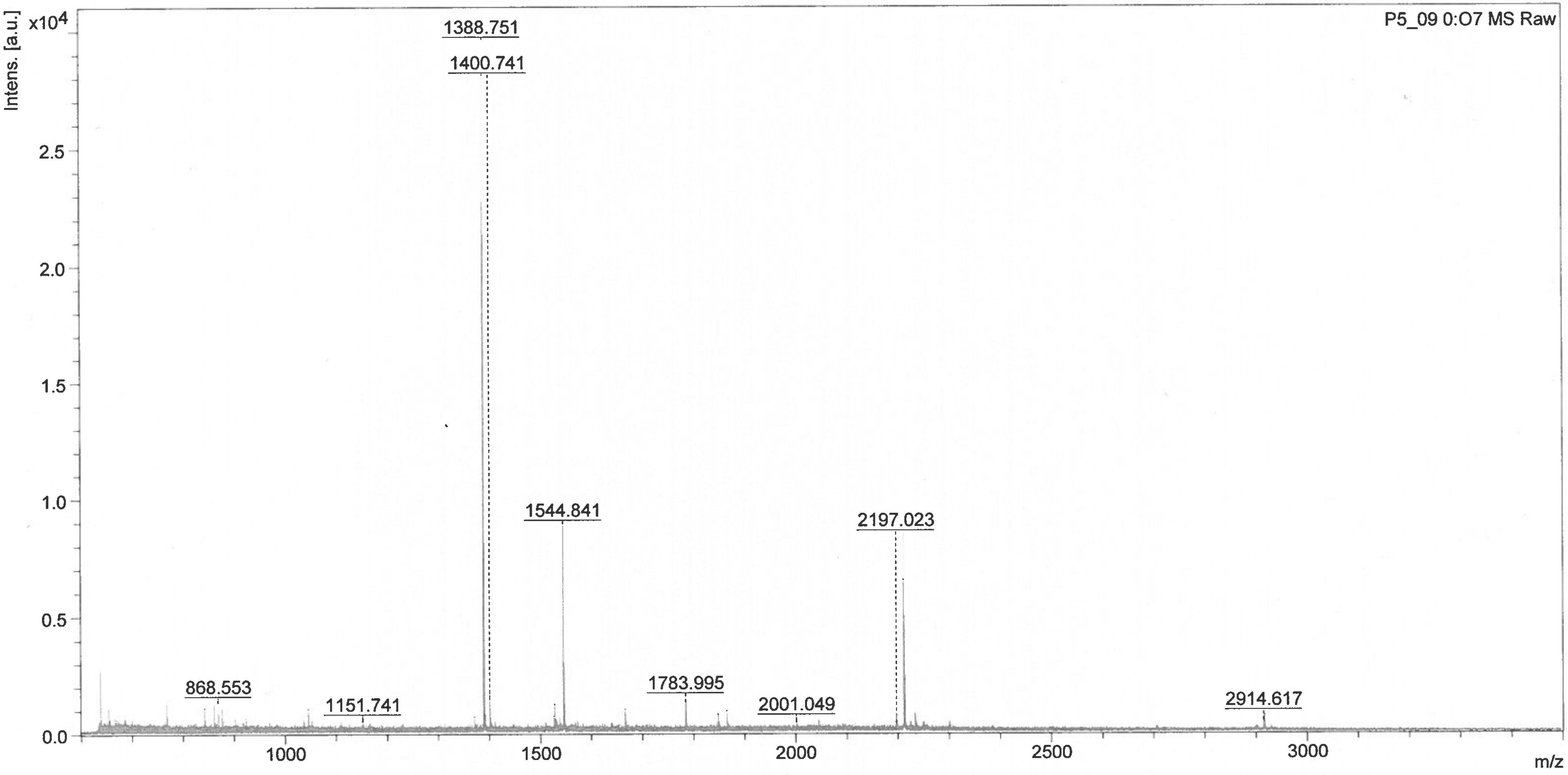

Bruker Daltonics flexAnalysis printed: 7/30/2011 12:48:42 PM

Spectrum Analysis Report

Date: 07/30/2011 Time: 12:48

FileName: D:\Data\Bernardo\2011_07_30\P5_09\0_O7\1\1SRef\pdata\1\PMF_LIFT.xml

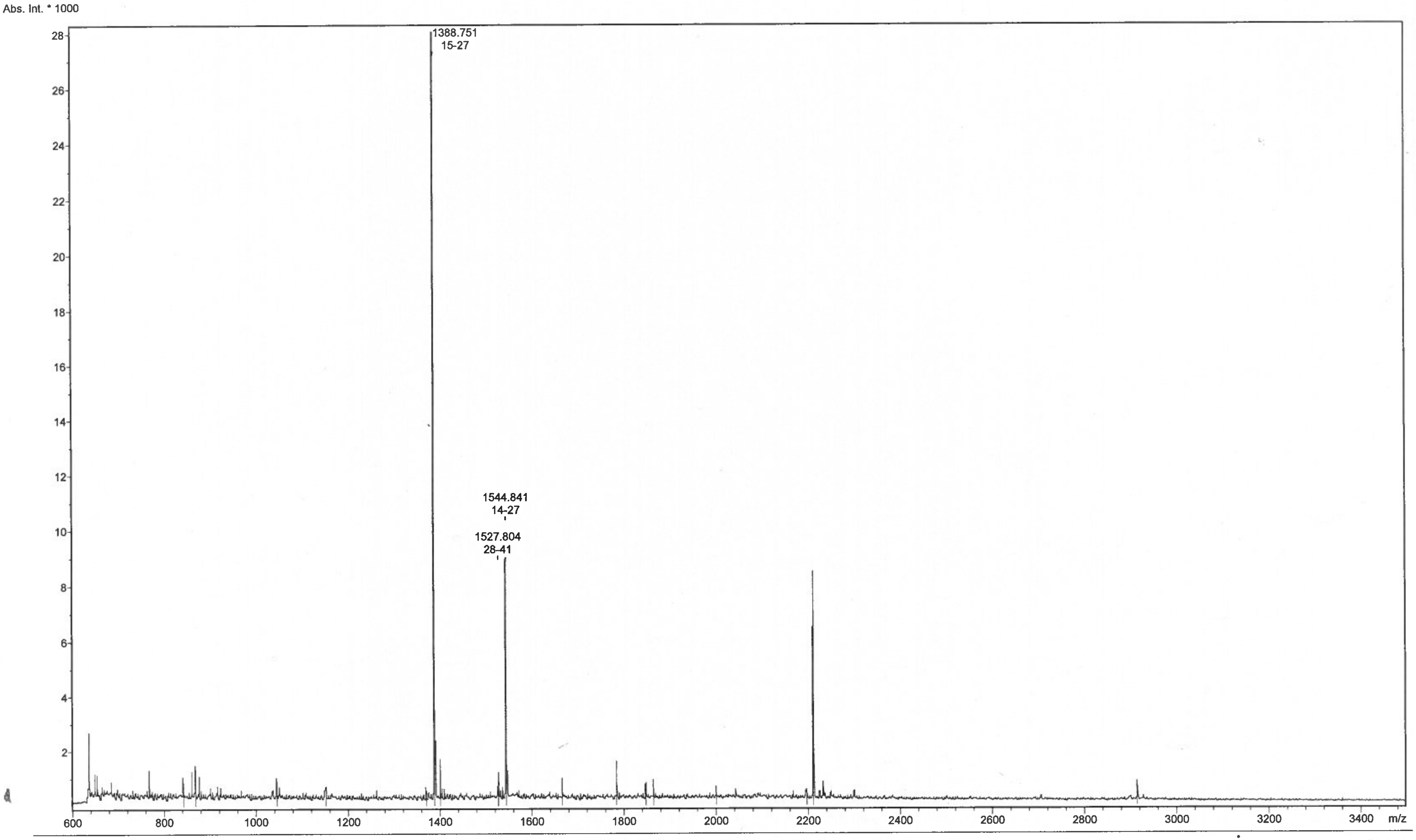

Copyright © 1999-2008 Bruker Daltonik GmbH

Spectrum Analysis Report

Date: 07/30/2011 Time: 12:48

FileName: D:\Data\Bernardo\2011_07_30\P5_09\0_O7\1\1SRef\pdata\1\PMF_LIFT.xml

Sequence data:

Stathmin OS=Mus musculus GN=Stmn1 PE=1 SV=2 STMN1_MOUSE

Intensity Coverage:	68.7 % (38987 cnts)	Sequence Coverage MS:	18.8%
Sequence Coverage MS/MS:	9.4%	pI (isoelectric point):	5.7

10	20	30	40	50	60	70	80	90	100	110
MASSDIQVKE	LEKRASGQAF	ELILSPRSKE	SVPDFPLSPP	KKKDLSLEEI	QKKLEAAEER	RKSHEAEVLK	QLAEKREHEK	EVLQKAIEEN	NNFSKMAEEK	LTHKMEANKE
120	130	140	150							
NREAQMAAKL	ERLREKDKHV	EEVRKNKESK	DPADETEAD							

Acquisition Parameter:

Matched Sequences:

Unmatched

Peaks/MSMS Spectra

Tree hierarchy	Meas. M/z	Calc. MH+	Meas. Mr	Calc. Mr	Int.	z	Dev.(Da)	Dev.(ppm)	Score	MascotScore	Rt(min)	Range	P	Sequence
peak 1	842.500	-	841.493	-	846.471	1+	-	-	-	-	-		-	
peak 2	868.553	-	867.546	-	1319.377	1+	-	-	-	-	-		-	
peak 3	1045.559	-	1044.552	-	883.681	1+	-	-	-	-	-		-	
peak 4	1151.741	-	1150.734	-	504.402	1+	-	-	-	-	-		-	
peak 5	1370.726	-	1369.719	-	538.954	1+	-	-	-	-	-		-	
peak 7	1400.741	-	1399.734	-	1486.945	1+	-	-	-	-	-		-	
peak 8	1526.802	-	1525.795	-	499.262	1+	-	-	-	-	-		-	
peak 11	1665.924	-	1664.917	-	809.372	1+	-	-	-	-	-		-	
peak 12	1783.995	-	1782.988	-	1323.692	1+	-	-	-	-	-		-	
peak 13	1847.986	-	1846.979	-	571.853	1+	-	-	-	-	-		-	
peak 14	1865.002	-	1863.994	-	626.024	1+	-	-	-	-	-		-	
peak 15	2001.049	-	2000.042	-	458.428	1+	-	-	-	-	-		-	
peak 16	2197.023	-	2196.016	-	421.214	1+	-	-	-	-	-		-	
peak 17	2211.126	-	2210.119	-	6733.331	1+	-	-	-	-	-		-	
peak 18	2914.617	-	2913.610	-	502.781	1+	-	-	-	-	-		-	

Global peptide results

mCG12955, isoform CRA_b [Mus musculus] gi|148698063

MW:13376.990

MASSDIQVKELEKRASGQAFELILSPRSKESVPDFPLSPPKKKDLSLEEIQKKLEAAEERRKSHEAEVLKQLAEKREHEKELTHKMERLREKDKHVEEVRKNKESKDPADETEAD

Digest Matches (Score: 135.00)

Score = 135.000000, Rank = 1, Database = NCBInr, Accesskey = gi|148698063

Search Parameters: MS Tol.:100.00 ppm, MSMS Tol.:0.600000Da, Enz.Trypsin, Engine:Mascot Version:2.3.01.241, DB:NCBInr NCBInr, DB Version:NCBInr_20110715.fasta NCBInr_20110715.fasta

Modifications: Optional: Oxidation (M)

Tree hierarchy	Meas. M/z	Calc. MH+	Meas. Mr	Calc. Mr	Int.	z	Dev.(Da)	Dev.(ppm)	Score	MascotScore	Rt(min)	Range	P	Sequence
MSMS 6	1388.751	1388.753	1387.744	1387.746	29591.207	1+	-0.002	-1.545	150	75	-	15 - 27	0	ASGQAFELILSPR
peak 9	1527.804	1527.805	1526.797	1526.798	734.949	1+	-0.001	-0.819	-	-	-	28 - 41	1	SKESVPDFPLSPPK
MSMS 10	1544.841	1544.854	1543.834	1543.847	8896.038	1+	-0.013	-8.731	38	21	-	14 - 27	1	RASGQAFELILSPR

Stathmin OS=Mus musculus GN=Stmn1 PE=1 SV=2 STMN1_MOUSE

MW:17263.920

MASSDIQVKELEKRASGQAFELILSPRSKESVPDFPLSPPKKKDLSLEEIQKKLEAAEERRKSHEAEVLKQLAEKREHEKEVLQKAIEENNNFSKMAEEKLTHKMEANKENREAQMAAKLERLREKDKHVEEVRKNKESKDPADETEAD

Digest Matches (Score: 130.00)

Score = 130.000000, Rank = 1, Database = SwissProt, Accesskey = STMN1_MOUSE

Search Parameters: MS Tol.:100.00 ppm, MSMS Tol.:0.600000Da, Enz.Trypsin, Engine:Mascot Version:2.3.01.241, DB:NCBInr NCBInr, DB Version:NCBInr_20110715.fasta NCBInr_20110715.fasta

Modifications: Optional: Oxidation (M)

Tree hierarchy	Meas. M/z	Calc. MH+	Meas. Mr	Calc. Mr	Int.	z	Dev.(Da)	Dev.(ppm)	Score	MascotScore	Rt(min)	Range	P	Sequence
MSMS 6	1388.751	1388.753	1387.744	1387.746	29591.207	1+	-0.002	-1.545	150	75	-	15 - 27	0	ASGQAFELILSPR
peak 9	1527.804	1527.805	1526.797	1526.798	734.949	1+	-0.001	-0.819	-	-	-	28 - 41	1	SKESVPDFPLSPPK
MSMS 10	1544.841	1544.854	1543.834	1543.847	8896.038	1+	-0.013	-8.731	38	21	-	14 - 27	1	RASGQAFELILSPR

Copyright © 1999-2008 Bruker Daltonik GmbH
