# Supplementary material for: Proteomic Analysis of the Action of the Mycobacterium ulcerans Toxin Mycolactone: Targeting Host Cells Cytoskeleton and Collagen
Source: PLoS Negl Trop Dis. 2014 Aug 7;8(8):e3066. doi: 10.1371/journal.pntd.0003066 (PMC4125307; doi:10.1371/journal.pntd.0003066)
Supplement: Dataset S7 — MS and MS/MS data. (ZIP) [file pntd.0003066.s010.zip › MS Data/Spot 19 - Ftl1.pdf]

D:\Data\Bernardo\2011_07_26\M23_08\0_J14\1\1SRef

Comment 1

Comment 2

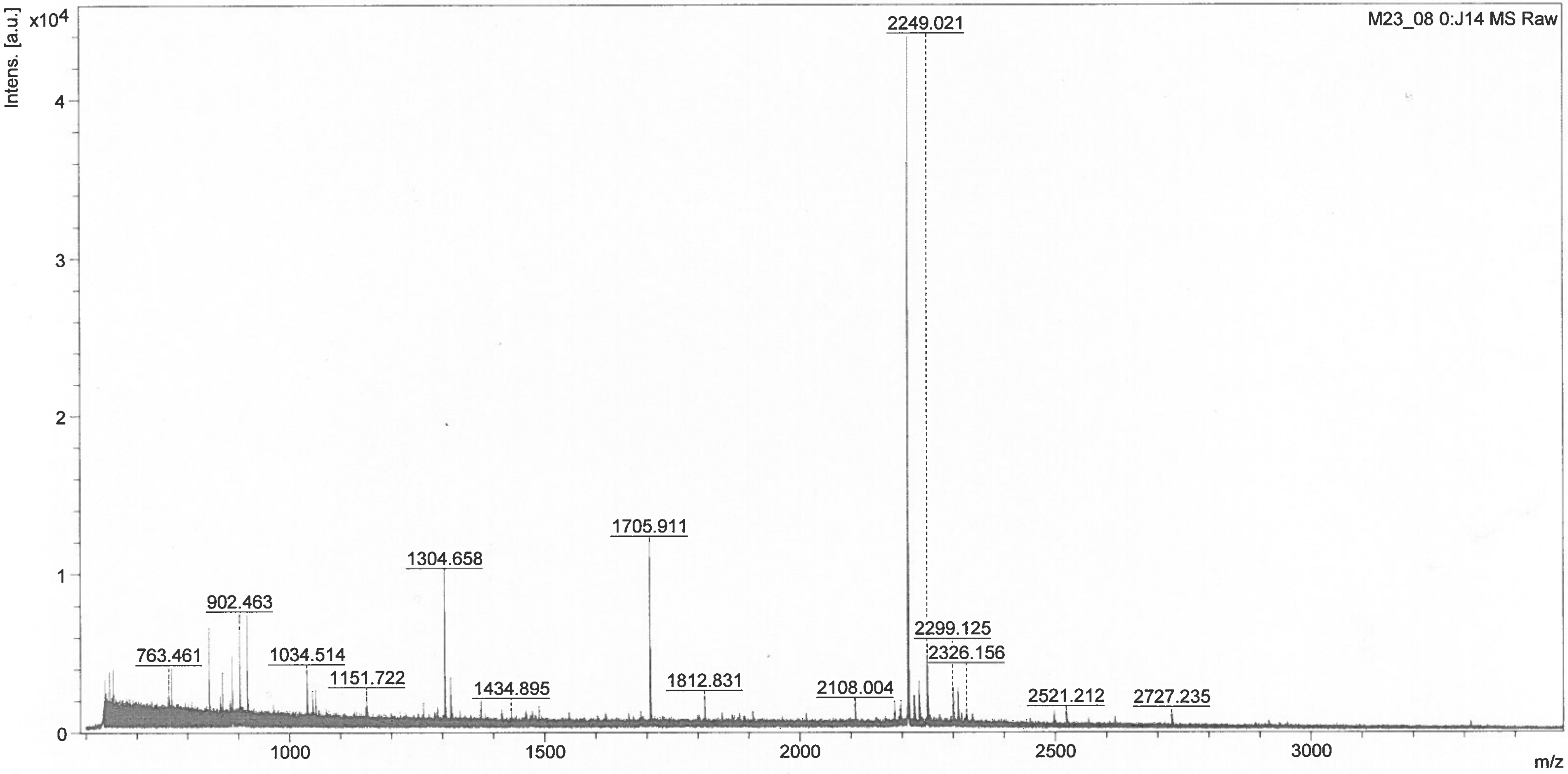

Bruker Daltonics flexAnalysis printed: 7/29/2011 6:49:50 AM

Spectrum Analysis Report

Date: 07/29/2011 Time: 06:53

FileName: D:\Data\Bernardo\2011_07_26\M23_08\0_J14\1\1SRef\pdata\1\PMF_LIFT.xml

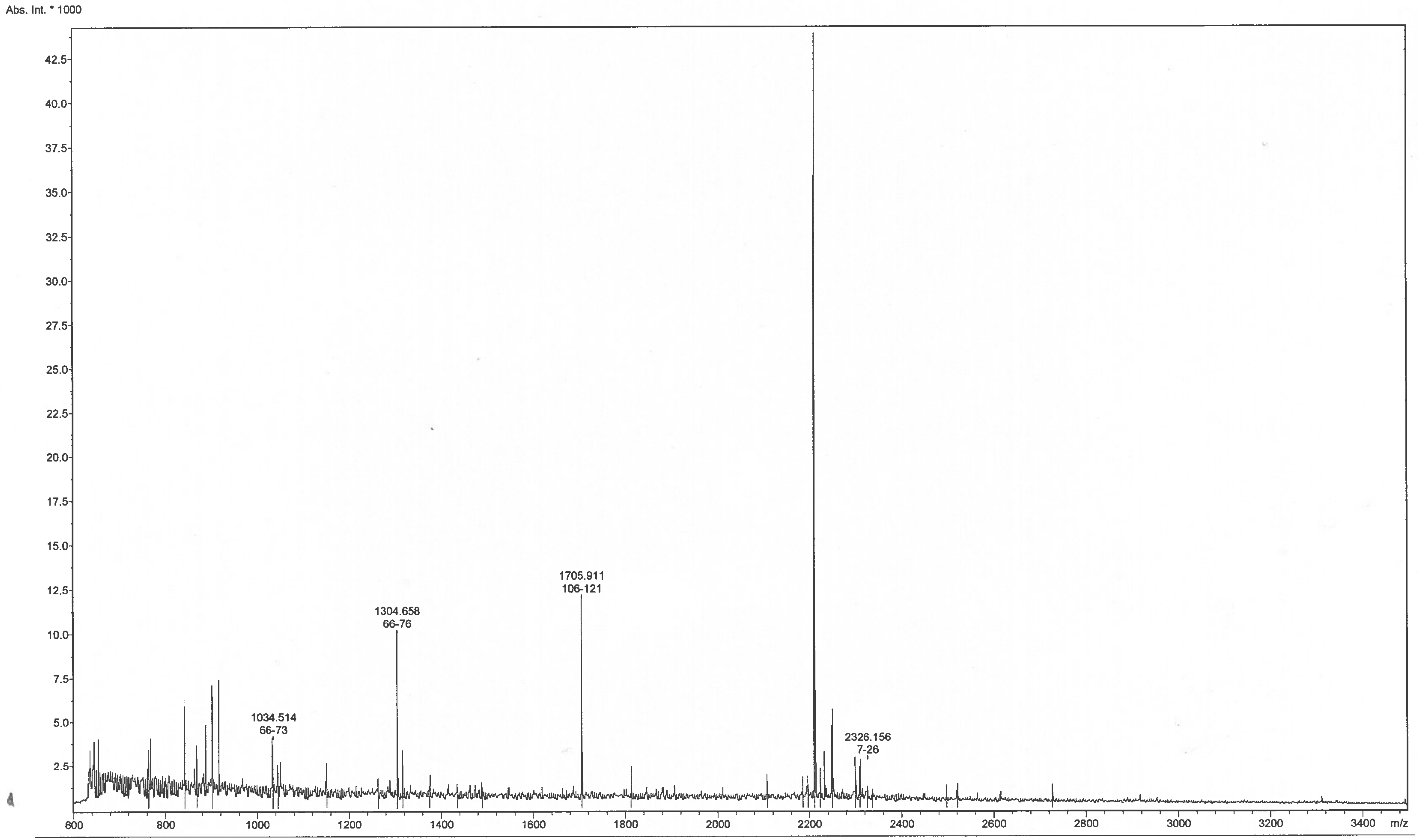

BioTools Version 3.2

Copyright © 1999-2008 Bruker Daltonik GmbH

Spectrum Analysis Report

Date: 07/29/2011 Time: 06:53

FileName: D:\Data\Bernardo\2011_07_26\M23_08\0_J14\1\1SRef\pdata\1\PMF_LIFT.xml

Sequence data:

Ferritin light chain 1 OS=Mus musculus GN=Ftl1 PE=1 SV=2 FRIL1_MOUSE

Intensity Coverage:	23.4 % (26788 cnts)	Sequence Coverage MS:	25.7%
Sequence Coverage MS/MS:	14.8%	pI (isoelectric point):	5.6

10	20	30	40	50	60	70	80	90	100	110
MTSQIRQNYS	TEVEAAVNRL	VNLHLRASYT	YLSLGFFFDR	DDVALEGVGH	FFRELAEEKR	EGAERLLEFQ	NDRGGRALFQ	DVQKPSQDEW	GKTQEAMEAA	LAMEKNLNQA
120	130	140	150	160	170	180	190			
LLDLHALGSA	RTDPHLCDFL	ESHYLDKEVK	LIKKMGNHLT	NLRRVAGPQP	AQTGAPQGSL	GEYLFERLTL	KHD			

Acquisition Parameter:

Matched Sequences:

Unmatched

Peaks/MSMS Spectra

Tree hierarchy	Meas. M/z	Calc. MH+	Meas. Mr	Calc. Mr	Int.	z	Dev.(Da)	Dev.(ppm)	Score	MascotScore	Rt(min)	Range	P	Sequence
peak 1	763.461	-	762.454	-	2701.167	1+	-	-	-	-	-		-	
peak 2	842.497	-	841.489	-	5905.307	1+	-	-	-	-	-		-	
peak 3	868.547	-	867.539	-	2925.533	1+	-	-	-	-	-		-	
peak 4	902.463	-	901.455	-	6484.208	1+	-	-	-	-	-		-	
peak 6	1045.541	-	1044.534	-	2092.995	1+	-	-	-	-	-		-	
peak 7	1151.722	-	1150.715	-	1992.846	1+	-	-	-	-	-		-	
peak 8	1262.929	-	1261.921	-	1441.008	1+	-	-	-	-	-		-	
peak 10	1316.573	-	1315.565	-	2526.227	1+	-	-	-	-	-		-	
peak 11	1375.009	-	1374.002	-	1391.383	1+	-	-	-	-	-		-	
peak 12	1434.895	-	1433.887	-	1289.364	1+	-	-	-	-	-		-	
peak 13	1489.090	-	1488.083	-	1255.774	1+	-	-	-	-	-		-	
peak 15	1812.831	-	1811.824	-	2041.851	1+	-	-	-	-	-		-	
peak 16	2108.004	-	2106.996	-	1555.113	1+	-	-	-	-	-		-	
peak 17	2185.045	-	2184.037	-	1108.215	1+	-	-	-	-	-		-	
peak 18	2185.547	-	2184.540	-	1102.343	1+	-	-	-	-	-		-	
peak 19	2195.998	-	2194.991	-	919.440	1+	-	-	-	-	-		-	
peak 20	2197.940	-	2196.933	-	1038.101	1+	-	-	-	-	-		-	
peak 21	2211.075	-	2210.068	-	35376.538	1+	-	-	-	-	-		-	
peak 22	2223.048	-	2222.041	-	1960.510	1+	-	-	-	-	-		-	
peak 23	2249.021	-	2248.014	-	4619.500	1+	-	-	-	-	-		-	
peak 24	2299.125	-	2298.117	-	2180.702	1+	-	-	-	-	-		-	
peak 25	2309.141	-	2308.134	-	2130.714	1+	-	-	-	-	-		-	
peak 27	2337.067	-	2336.059	-	822.043	1+	-	-	-	-	-		-	
peak 28	2497.271	-	2496.263	-	876.444	1+	-	-	-	-	-		-	
peak 29	2521.212	-	2520.205	-	1039.249	1+	-	-	-	-	-		-	
peak 30	2727.235	-	2726.228	-	795.421	1+	-	-	-	-	-		-	

Global peptide results

Ferritin light chain 1 OS=Mus musculus GN=Ftl1 PE=1 SV=2 FRIL1_MOUSE

MW:20846.520

MTSQIRQNYSTEVEAAVNRLVNLHLRASYTYLSLGFFFDRDDVALEGVGHFFRELAEEKREGAERLLEFQNDRGGRALFQDVQKPSQDEWGKTQEAMEAALAMEKNLNQALLDLHALGSARTDPHLCDFLESHYLDKEVKLIKKMGNHLTNLRRVAGPQPAQTGAPQGSLGEYLFERLTLKHD

Digest Matches (Score: 92.10)

Score = 92.100000, Rank = 1, Database = SwissProt, Accesskey = FRIL1_MOUSE

Search Parameters: MS Tol.:100.00 ppm, MSMS Tol.: 0.600000 Da, Enz.Trypsin, Engine: Mascot Version: 2.3.01.241 , DB: NCBInr NCBInr , DB Version: NCBInr_20110715.fasta NCBInr_20110715.fasta

Modifications: Optional: Oxidation (M)

Tree hierarchy	Meas. M/z	Calc. MH+	Meas. Mr	Calc. Mr	Int.	z	Dev.(Da)	Dev.(ppm)	Score	MascotScore	Rt(min)	Range	P	Sequence
peak 5	1034.514	1034.527	1033.506	1033.519	3692.019	1+	-0.013	-12.393	-	-	-	66 - 73	0	LLEFQNDR
MSMS 9	1304.658	1304.671	1303.651	1303.663	10217.038	1+	-0.012	-9.458	17	9	-	66 - 76	1	LLEFQNDRGGR
MSMS 14	1705.911	1705.934	1704.904	1704.927	12027.821	1+	-0.023	-13.453	46	42	-	106 - 121	0	NLNQALLDLHALGSAR
peak 26	2326.156	2326.226	2325.148	2325.219	850.797	1+	-0.070	-30.316	-	-	-	7 - 26	1	QNYSTEVEAAVNRLVNLHLR

Copyright © 1999-2008 Bruker Daltonik GmbH
