# Supplementary material for: Proteomic Analysis of the Action of the Mycobacterium ulcerans Toxin Mycolactone: Targeting Host Cells Cytoskeleton and Collagen
Source: PLoS Negl Trop Dis. 2014 Aug 7;8(8):e3066. doi: 10.1371/journal.pntd.0003066 (PMC4125307; doi:10.1371/journal.pntd.0003066)
Supplement: Dataset S7 — MS and MS/MS data. (ZIP) [file pntd.0003066.s010.zip › MS Data/Spot 20 - Btf3.pdf]

D:\Data\Bernardo\2011_07_27\M5_02\0_L4\1\1SRef

Comment 1

Comment 2

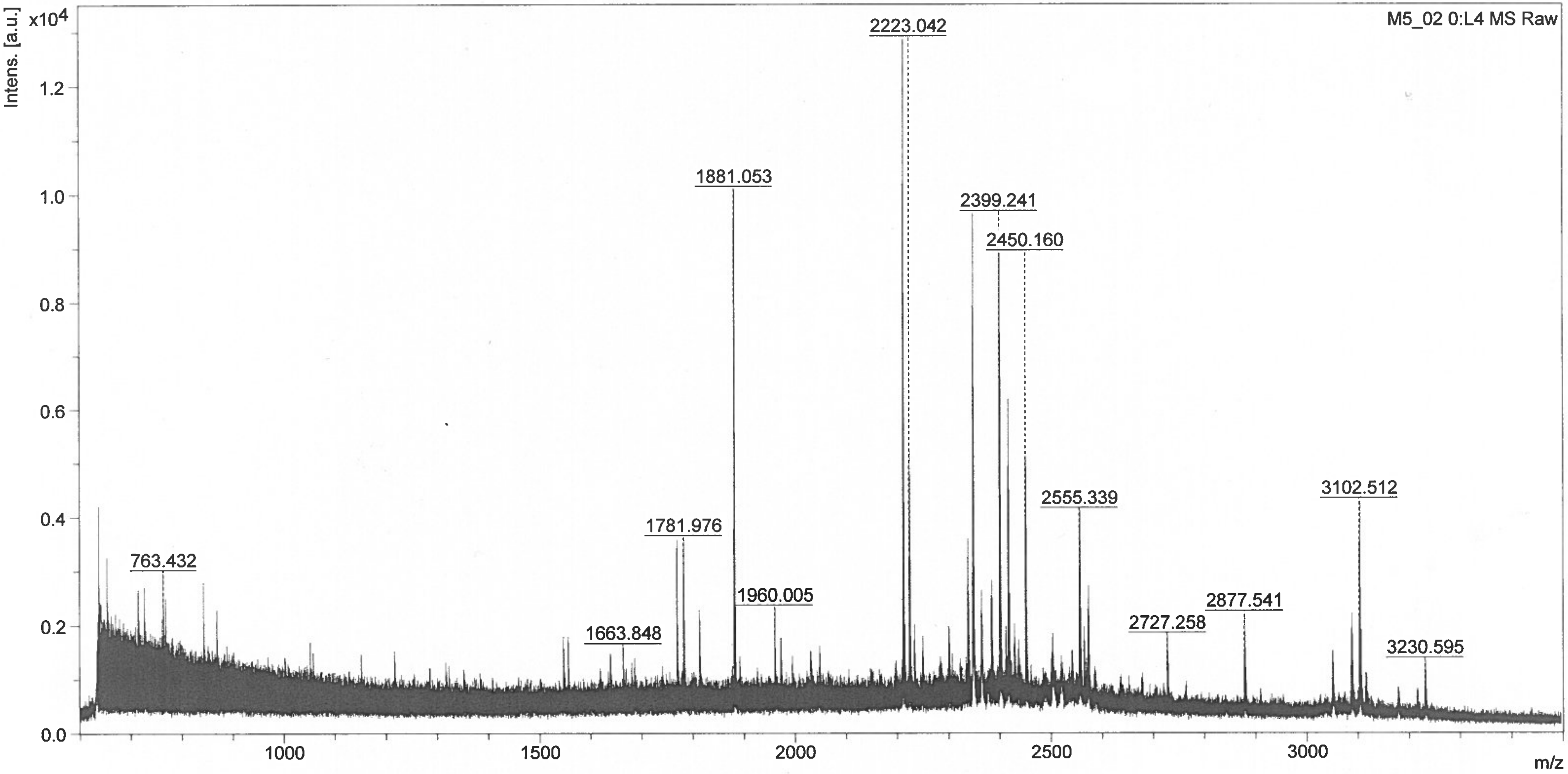

Bruker Daltonics flexAnalysis printed: 7/29/2011 7:11:59 AM

Spectrum Analysis Report

Date: 07/29/2011 Time: 07:11

FileName: D:\Data\Bernardo\2011_07_27\M5_02\0_L4\1\1SRef\pdata\1\PMF_LIFT.xml

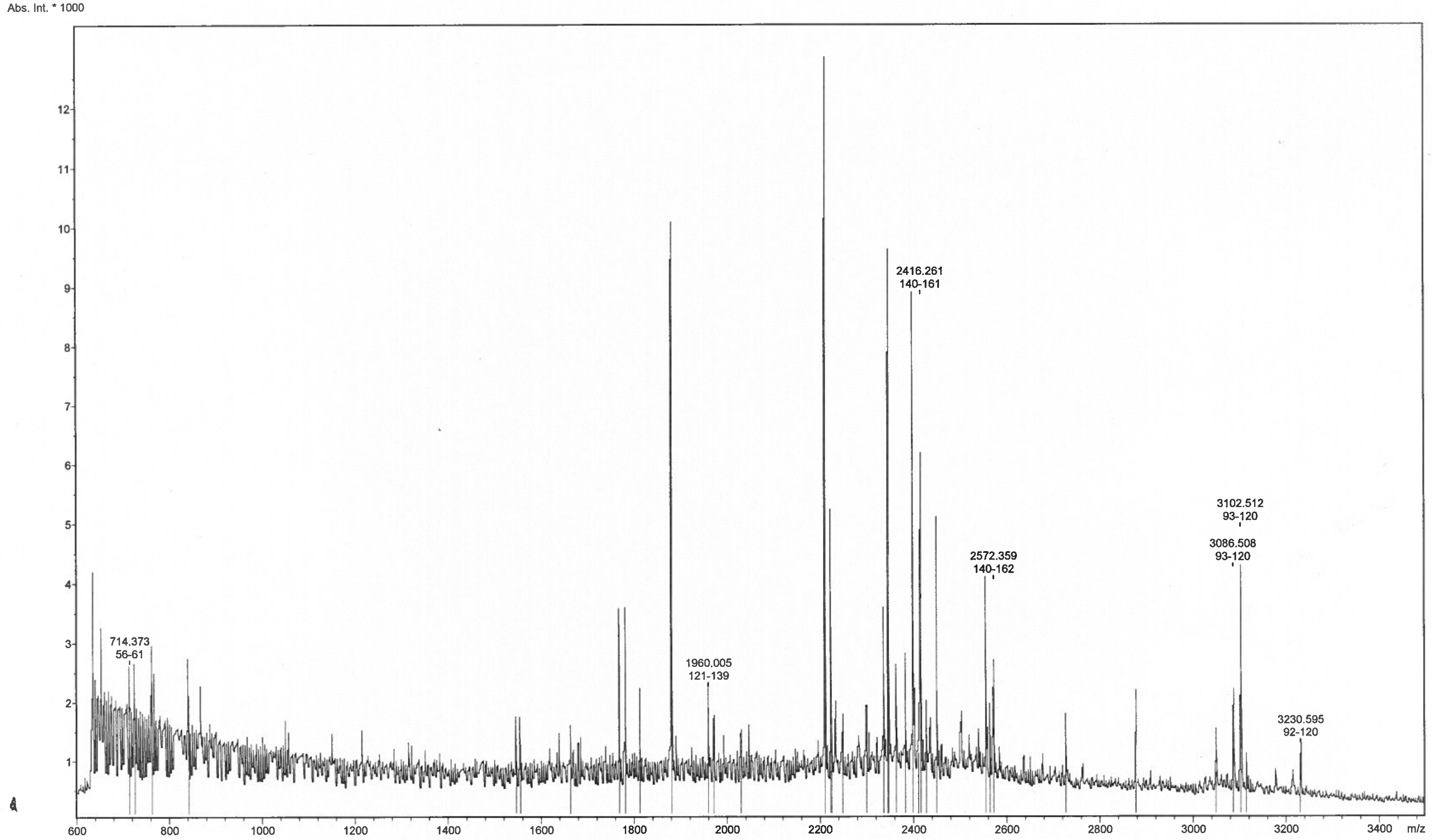

Copyright © 1999-2008 Bruker Daltonik GmbH

Spectrum Analysis Report

Date: 07/29/2011 Time: 07:11

FileName: D:\Data\Bernardo\2011_07_27\M5_02\0_L4\1\1SRef\pdata\1\PMF_LIFT.xml

Sequence data:

transcription factor BTF3 isoform 1 [Mus musculus] gi|56605979

Intensity Coverage:	13.5 % (12717 cnts)	Sequence Coverage MS:	37.7%
Sequence Coverage MS/MS:	10.8%	pI (isoelectric point):	10.0

```
        10         20         30         40         50         60         70         80         90        100        110
MRRTGAPTQA DSRGRGRARG GWPGAEATPS LPLGGSRGRE SQMKETIMNQ EKLAKLQAQV RIGGKGTARR KKKVVHRTAT ADDKKLQFSL KKLGVNNISG IEEVNMFTNQ
       120        130        140        150        160        170        180        190        200        210
GTVIHFNNPK VQASLAANTF TITGHAETKQ LTEMLPSILN QLGADSLTSL RRLAEALPKQ SVDGKAPLAT GEDDDDEVPD LVENFDEASK NEAN
```

Acquisition Parameter:

Matched Sequences:

Unmatched

Peaks/MSMS Spectra

Tree hierarchy	Meas. M/z	Calc. MH+	Meas. Mr	Calc. Mr	Int.	z	Dev.(Da)	Dev.(ppm)	Score	MascotScore	Rt(min)	Range	P	Sequence
peak 2	726.368	-	725.361	-	1749.488	1+	-	-	-	-	-		-	
peak 3	763.432	-	762.425	-	2323.577	1+	-	-	-	-	-		-	
peak 4	842.470	-	841.463	-	2118.935	1+	-	-	-	-	-		-	
peak 5	1546.692	-	1545.685	-	1235.593	1+	-	-	-	-	-		-	
peak 6	1555.819	-	1554.812	-	1274.742	1+	-	-	-	-	-		-	
peak 7	1663.848	-	1662.840	-	1417.993	1+	-	-	-	-	-		-	
peak 8	1768.951	-	1767.944	-	3295.185	1+	-	-	-	-	-		-	
peak 9	1781.976	-	1780.969	-	3120.524	1+	-	-	-	-	-		-	
peak 10	1812.837	-	1811.829	-	1786.588	1+	-	-	-	-	-		-	
peak 11	1881.053	-	1880.046	-	7970.506	1+	-	-	-	-	-		-	
peak 13	1971.973	-	1970.965	-	1392.055	1+	-	-	-	-	-		-	
peak 14	2030.033	-	2029.026	-	1127.439	1+	-	-	-	-	-		-	
peak 15	2211.086	-	2210.079	-	9735.324	1+	-	-	-	-	-		-	
peak 16	2223.042	-	2222.035	-	3350.335	1+	-	-	-	-	-		-	
peak 17	2224.998	-	2223.990	-	1728.120	1+	-	-	-	-	-		-	
peak 18	2249.028	-	2248.020	-	1104.776	1+	-	-	-	-	-		-	
peak 19	2300.139	-	2299.132	-	1336.831	1+	-	-	-	-	-		-	
peak 20	2337.077	-	2336.070	-	2393.035	1+	-	-	-	-	-		-	
peak 21	2345.677	-	2344.670	-	1574.373	1+	-	-	-	-	-		-	
peak 22	2346.262	-	2345.255	-	3923.999	1+	-	-	-	-	-		-	
peak 23	2347.304	-	2346.297	-	4468.835	1+	-	-	-	-	-		-	
peak 24	2363.541	-	2362.533	-	1502.304	1+	-	-	-	-	-		-	
peak 25	2383.231	-	2382.224	-	2044.576	1+	-	-	-	-	-		-	
peak 26	2399.241	-	2398.234	-	5908.145	1+	-	-	-	-	-		-	
peak 27	2411.203	-	2410.196	-	1121.254	1+	-	-	-	-	-		-	
peak 29	2428.250	-	2427.243	-	1123.118	1+	-	-	-	-	-		-	
peak 30	2450.160	-	2449.153	-	3300.782	1+	-	-	-	-	-		-	
peak 31	2555.339	-	2554.332	-	2474.933	1+	-	-	-	-	-		-	
peak 32	2564.204	-	2563.197	-	1262.660	1+	-	-	-	-	-		-	
peak 34	2727.258	-	2726.251	-	1132.956	1+	-	-	-	-	-		-	
peak 35	2877.541	-	2876.534	-	1365.320	1+	-	-	-	-	-		-	
peak 36	3049.615	-	3048.608	-	787.723	1+	-	-	-	-	-		-	
peak 39	3114.506	-	3113.498	-	698.528	1+	-	-	-	-	-		-	

Global peptide results

transcription factor BTF3 isoform B [Homo sapiens] gi|20070130

MW:17688.210

MKETIMNQEKLAKLQAQVRIGGKGTARRKKKVVHRTATADDKKLQFSLKKLGVNNISGIEEVNMFTNQGTVIHFNNPKVQASLAANTFTITGHAETKQLTEMLPSILNQLGADSLTSLRRLAEALPKQSVDGKAPLATGEDDDDEVPDLVENFDEASKNEAN

Digest Matches (Score: 195.00)

Score = 195.000000, Rank = 1, Database = NCBInr, Accesskey = gi|20070130

Search Parameters: MS Tol.:100.00 ppm, MSMS Tol.: 0.600000 Da, Enz.Trypsin, Engine: Mascot Version: 2.3.01.241 , DB: NCBInr NCBInr , DB Version: NCBInr_20110715.fasta NCBInr_20110715.fasta

Modifications: Optional: Oxidation (M)

Tree hierarchy	Meas. M/z	Calc. MH+	Meas. Mr	Calc. Mr	Int.	z	Dev.(Da)	Dev.(ppm)	Score	MascotScore	Rt(min)	Range	P	Sequence
peak 1	714.373	714.426	713.365	713.418	1861.213	1+	-0.053	-74.517	-	-	-	14 - 19	0	LQAQVR
peak 12	1960.005	1960.013	1958.998	1959.006	1835.012	1+	-0.008	-4.157	-	-	-	79 - 97	0	VQASLAANTFTITGHAETK

Copyright © 1999-2008 Bruker Daltonik GmbH

MSMS 28	2416.261	2416.275	2415.254	2415.268	3755.591	1+	-0.014	-5.858	16410	122	-	98 - 119	0	QLTEMLPSILNQLGADSLTSLR 5: Oxidation (M)
peak 33	2572.359	2572.376	2571.352	2571.369	1460.068	1+	-0.017	-6.645	-	-	-	98 - 120	1	QLTEMLPSILNQLGADSLTSLRR 5: Oxidation (M)
peak 37	3086.508	3086.536	3085.501	3085.529	1113.528	1+	-0.029	-9.262	-	-	-	51 - 78	0	LGVNNISGIEEVNMFTNQGTVIHFNNPK
peak 38	3102.512	3102.531	3101.505	3101.524	2031.960	1+	-0.019	-6.160	-	-	-	51 - 78	0	LGVNNISGIEEVNMFTNQGTVIHFNNPK 14: Oxidation (M)
peak 40	3230.595	3230.626	3229.587	3229.619	659.179	1+	-0.032	-9.788	-	-	-	50 - 78	1	KLGVNNISGIEEVNMFTNQGTVIHFNNPK 15: Oxidation (M)

transcription factor BTF3 isoform 1 [Mus musculus] gi|56605979
MW:22017.390

MRRTGAPTQADSRGRGRARGGWPGAEATPSLPLGGSRGRESQMKETIMNQEKLAKLQAQVRIGGKGTARRKKKVVHRTATADDKKLQFSLKKLGVNNISGIEEVNMFTNQGTVIHFNNPKVQASLAANTFTITGHAETKQLTEMLPSILNQLGADSLTSLRRLAEALPKQSVDGKAPLATGEDDDDEVPDLVENFDEASKNEAN

Digest Matches (Score: 189.00)
Score = 189.000000, Rank = 1, Database = NCBInr, Accesskey = gi|56605979
Search Parameters: MS Tol.:100.00 ppm, MSMS Tol.: 0.600000 Da, Enz.Trypsin, Engine: Mascot Version: 2.3.01.241 , DB: NCBInr NCBInr , DB Version: NCBInr_20110715.fasta NCBInr_20110715.fasta
Modifications: Optional: Oxidation (M)

Tree hierarchy	Meas. M/z	Calc. MH+	Meas. Mr	Calc. Mr	Int.	z	Dev.(Da)	Dev.(ppm)	Score	MascotScore	Rt(min)	Range	P	Sequence
peak 1	714.373	714.426	713.365	713.418	1861.213	1+	-0.053	-74.517	-	-	-	56 - 61	0	LQAQVR
peak 12	1960.005	1960.013	1958.998	1959.006	1835.012	1+	-0.008	-4.157	-	-	-	121 - 139	0	VQASLAANTFTITGHAETK
MSMS 28	2416.261	2416.275	2415.254	2415.268	3755.591	1+	-0.014	-5.858	16410	122	-	140 - 161	0	QLTEMLPSILNQLGADSLTSLR 5: Oxidation (M)
peak 33	2572.359	2572.376	2571.352	2571.369	1460.068	1+	-0.017	-6.645	-	-	-	140 - 162	1	QLTEMLPSILNQLGADSLTSLRR 5: Oxidation (M)
peak 37	3086.508	3086.536	3085.501	3085.529	1113.528	1+	-0.029	-9.262	-	-	-	93 - 120	0	LGVNNISGIEEVNMFTNQGTVIHFNNPK
peak 38	3102.512	3102.531	3101.505	3101.524	2031.960	1+	-0.019	-6.160	-	-	-	93 - 120	0	LGVNNISGIEEVNMFTNQGTVIHFNNPK 14: Oxidation (M)
peak 40	3230.595	3230.626	3229.587	3229.619	659.179	1+	-0.032	-9.788	-	-	-	92 - 120	1	KLGVNNISGIEEVNMFTNQGTVIHFNNPK 15: Oxidation (M)

Copyright © 1999-2008 Bruker Daltonik GmbH
